# Harnessing Methylselenol (CH_3_SeH): A Highly Effective Platform for Stimuli‐Responsive Delivery With Therapeutic Potential

**DOI:** 10.1002/advs.78356

**Published:** 2026-10-18

**Authors:** Wenfang Liang, Liyue Wang, Xuan Wang, Xinran Li, Hao Xing, Lu Sun, Zhen Xi, Long Yi

**Affiliations:** ^1^ College of Chemical Engineering and Beijing Key Laboratory of Bioprocess Beijing University of Chemical Technology Beijing China; ^2^ Tianjin Key Laboratory On Technologies Enabling Development of Clinical Therapeutics and Diagnostics (Theranostics) School of Pharmacy Tianjin Medical University Tianjin China; ^3^ National Pesticide Engineering Research Center (Tianjin) Department of Chemical Biology State Key Laboratory of Elemento‐Organic Chemistry College of Chemistry Frontiers Science Center For New Organic Matter Nankai University Tianjin China

**Keywords:** cellular delivery, methylselenol, prodrug, reactive selenium species, responsive release

## Abstract

Methylselenol (CH_3_SeH) is not only a key selenium (Se) metabolite but also a potent therapeutic molecule. Chemical tools for stimulus‐responsive delivery of CH_3_SeH should be crucial for exploring Se‐related biomedicine. Nevertheless, the classical 1,6‐self‐immolation is inefficient in releasing the poorly leaving selenol, and donation platforms for triggerable release of the CH_3_SeH remain lacking. Herein, we report the first general responsive platform (Triggered Donation for MethylSelenol, termed TD‐MSe) via fast intramolecular amino‐activated methylselenocarbamates (*t*_1/2_ = 5 s at pH 7.4), enabling the efficient cyclization release of CH_3_SeH without electrophilic byproducts. We leverage the caged TD‐MSe motifs to successfully create triggered donors that are stable before activation but can liberate CH_3_SeH under different stimulus conditions in vitro and in live cells. Additionally, we demonstrate that the tumor‐associated oxidoreductase (hNQO1)‐triggered CH_3_SeH donor was activated in hNQO1‐positive HT‐29 cells but not in hNQO1‐negative cells. Moreover, we can use the CH_3_SeH prodrug to selectively kill metastatic cells of hNQO1‐overexpressing cancer types, providing a promising therapeutic strategy for difficult‐to‐treat diseases. We anticipate that the new‐release chemistry will be widely applied for developing advanced tools toward smart selenol delivery and therapy.

## Graphical abstract

Tools for controlled selenol delivery are crucial for exploring Se‐related biology and medicine, but compound platforms for triggerable release of CH_3_SeH remain lacking. Herein, we report the first general triggered platform (TD‐MSe) for efficient cyclization release of CH_3_SeH in live cells. The new well‐defined release chemistry should significantly promote the development of stimulus‐responsive systems with therapeutic potential.

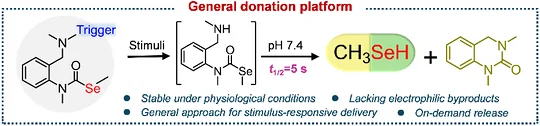

## 1 Introduction

Selenium (Se) is an essential trace element for chemoprevention of cancer, and various Se compounds have been assessed in clinical trials for multiple malignancies, including prostate, colon, and lung cancers [1, 2, 3, 4, 5, 6, 7, 8]. In the complex metabolism network, these Se compounds are metabolized into different redox‐active products that mainly belong to two pools (Figure 1A): a hydrogen selenide (H_2_Se) pool (e.g., from selenite) and a methylselenol (CH_3_SeH) pool (e.g., from methylseleninic acid (MSA), methylselenocysteine (MeSec)) [8, 9, 10, 11, 12, 13]. Although H_2_Se pools exert certain antitumor activity, excess H_2_Se that surpasses the physiological threshold for selenoprotein biosynthesis may induce DNA single‐strand cleavage [14, 15, 16, 17], which markedly compresses the therapeutic safety window and raises metastatic risks of tumors. In contrast, CH_3_SeH is relatively nongenotoxic and acts independently of selenoprotein saturation levels [18, 19, 20]. It targets tumors via multiple mechanisms, including regulating the expression of NKG2D ligands [21], inducing caspase‐mediated apoptosis [22], modulating oxidative stress [23], and inhibiting the expression of the angiogenic factor VEGF to block angiogenesis [20, 24]. Furthermore, it can also synergistically enhance the effects of chemoradiotherapy and immunotherapy and reduce drug side effects, possessing numerous desirable antitumorigenic properties [25, 26, 27, 28, 29, 30]. Accordingly, the development of CH_3_SeH precursors and donors should be of great significance for cancer prevention and treatment.

**FIGURE 1:**
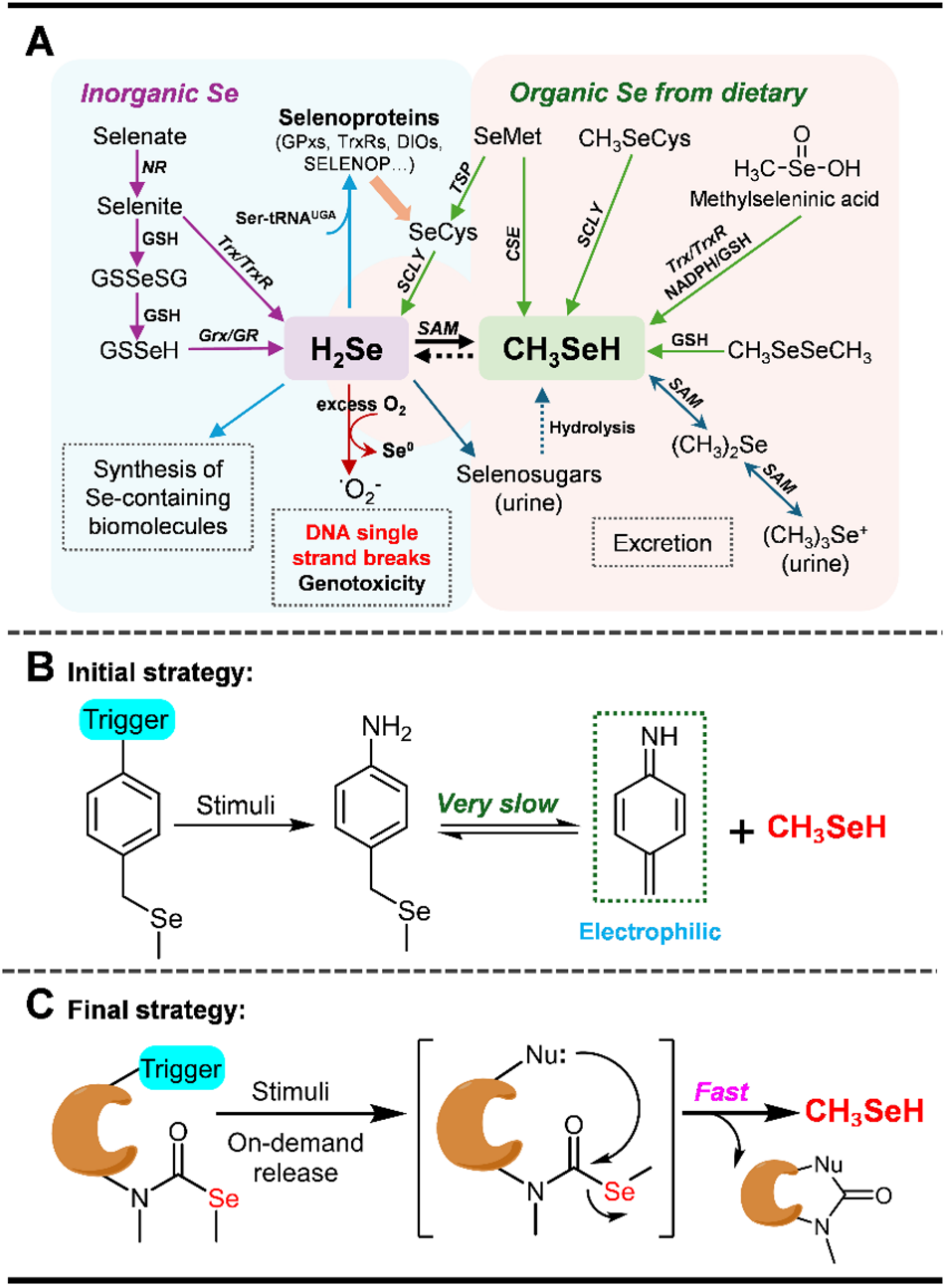
Strategies for biogenesis or controlled release of CH_3_SeH. (A) Overview of the Se metabolic pathways to generate the H_2_Se and CH_3_SeH pools. GSSeSG, selenodiglutathione; GSSeH, glutathioselenol; SeCys, selenocysteine; NR, nitroreductase; Trx, thioredoxin; TrxR, thioredoxin reductase; GR, glutathione reductase; Grx, glutaredoxin; GSH, glutathione; TSP, transsulfuration pathway; NADPH, nicotinamide adenine dinucleotide phosphate; CSE, cystathionine γ‐lyase; SCLY, selenocysteine β‐lyase; SAM, *S*‐adenosylmethionine‐dependent methylation or demethylation. (B) Initial design of triggered CH_3_SeH donors via a very slow self‐immolative process. (C) Final design via a triggerable intramolecular cyclization for fast‐releasing CH_3_SeH without electrophilic byproducts.

Natural MeSec can be solely metabolized to release CH_3_SeH [8, 12, 21], and this metabolic process is highly dependent on the expression level of endogenous β‐lyase, which should be enhanced to improve the release efficiency and therapeutic efficacy [31]. Selenomethionine (SeMet), a clinically prevalent Se dietary supplement, also serves as a CH_3_SeH precursor in the presence of γ‐lyase [7, 11], but the main metabolic pathway of SeMet is the conversion to H_2_Se [8, 12]. Although excess H_2_Se can also be metabolized into CH_3_SeH, SeMet at low micromolar concentrations cannot produce significant amounts of CH_3_SeH to exert anti‐tumor effects [10, 12, 32]. MSA needs to be converted by endogenous reduction systems to produce CH_3_SeH [6, 19, 33], and hydrolysis of methylselenocarbamates [34, 35, 36] and methylselenoesters [37, 38] can generate CH_3_SeH directly in physiological buffers. These donors and precursors have demonstrated impressive pharmacological activities due to their ability to liberate CH_3_SeH. Despite these successes, there are still problems such as uncontrollable release and low conversion efficiency, making it difficult to meet the needs of precise therapy. Moreover, selectively releasing CH_3_SeH in cancer cells without affecting normal cells is challenging. Developing stimulus‐responsive delivery platforms should be an effective strategy to address the dilemma [39, 40, 41, 42, 43, 44, 45], but this has not yet been reported for CH_3_SeH. An ideal stimulus‐responsive system can remain stable under physiological conditions and only convert to CH_3_SeH in response to a specific stimulus, enabling spatiotemporally controlled or “on‐demand” delivery in live biosystems. Motivated by these needs, herein we report a highly effective and general platform for the development of triggered CH_3_SeH donors.

To achieve this goal, we initially developed CH_3_SeH donors based on classical self‐immolative chemistry [45, 46, 47], but the release rate for CH_3_SeH was very slow (Figure 1B). On the other hand, distinct from the good stability of C = Se moieties in H_2_Se donors [44], these C‒Se scaffolds [34, 35, 36, 37, 38] lack sufficient water stability, restricting their applications for constructing triggered CH_3_SeH donors. Through extensive optimization, we identified a water‐stable methylselenocarbamate motif and then engineered an adjacent nucleophilic group to intramolecularly activate the motif, achieving rapid cyclization release of the CH_3_SeH (Figure 1C). Importantly, we created triggered donors that can be activated under specific stimuli and demonstrated their proof‐of‐concept applications for intracellular CH_3_SeH delivery.

## 2 Results and Discussion

### 2.1 1,6‐Self‐Immolation Is Very Slow for CH_3_SeH Donation

We first assessed the quinone methide self‐immolation [45, 46, 47] for developing CH_3_SeH donors and prepared compound **1** with an azide as the trigger (Figure 2A). We then examined the Staudinger‐mediated reduction of **1** to generate **1a**, which may undergo a self‐immolative process to liberate CH_3_SeH and **1b**. The reaction between **1** and tris‐(2‐carboxyethyl)‐phosphine (TCEP) in PBS (pH 7.4) at 25 °C was monitored by UV−vis experiments, permitting kinetic analysis of the Staudinger reduction. Time‐dependent spectra exhibited an isosbestic point at 247 nm within the initial 15 min (Figure S1), and the reaction kinetics was determined by monitoring the decrease at 257 nm. Pseudo‐first‐order rate constants (*k*_obs_) obtained at varying TCEP concentrations gave a linear relationship to yield a bimolecular rate constant *k*_2_ = 1.43 M^−1^s^−1^ (Figure 2B). This rate should be due to the conversion from **1** to **1a**, and HRMS analysis confirmed that **1a** is the main product (Figure S2). We also observed the mass peaks of **1b** and its hydrate, implying that the self‐immolation occurred very slowly. In time‐dependent ^77^Se NMR experiments (Figure S2), the results further support a fast Staudinger reaction followed by a very slow self‐decomposition of **1a**. We further conducted time‐dependent ^1^H NMR experiments of **1** with TCEP and found that all resonances of **1** disappeared within the initial 0.5 h (Figure S3), and the ensuing changes from 0.5 to 266.5 h reflected the slow decomposition of **1a**. Kinetic analysis of the decreasing resonances of **1a** gave a self‐immolative rate of 2.63 × 10^−6^ s^−1^ (Figure 2B), supporting a very slow CH_3_SeH release (Figure 2A). The half‐life (*t*_1/2_) of the self‐immolation is about 73 h, making such self‐immolative chemistry inefficient in the selenol donation.

**FIGURE 2:**
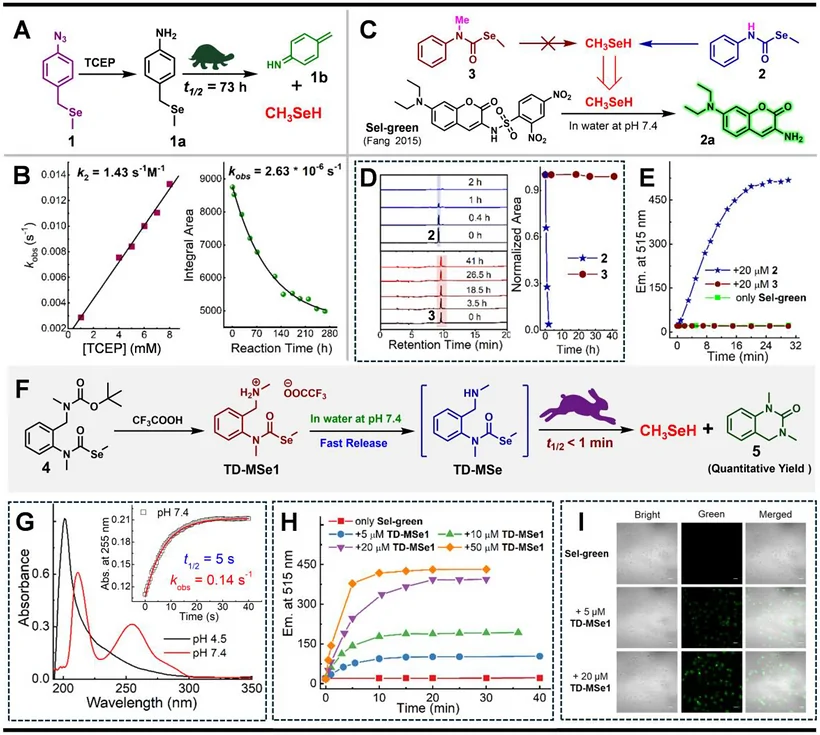
Developing a highly efficient releasing platform for CH_3_SeH delivery. (A) Reaction pathways of the self‐immolative donor to release CH_3_SeH. (B) Kinetic studies of the bimolecular rate by UV−vis analyses (left) and the self‐immolation rate by time‐dependent integral area of aromatic protons (at 7.35–7.31 ppm) obtained from ^1^H NMR spectra (right). (C) Illustration of **2** or **3** to generate CH_3_SeH and its fluorescence detection. (D) Time‐dependent HPLC analyses of **2** or **3** in PBS (pH 7.4) and the normalized time‐dependent peak areas from the HPLC analyses. (E) Time‐dependent emissions at 515 nm of **Sel‐green** (10 µM) without or with **2** or **3** (20 µM) in PBS (pH 7.4). Excitation: 370 nm. (F) Synthesis of **TD‐MSe1** and its pH‐triggered reaction pathways to generate CH_3_SeH and **5**. (G) UV−vis spectra of **TD‐MSe1** in PBS (pH 4.5 or 7.4). Inset: Time‐dependent absorbance intensities at 255 nm of **TD‐MSe1** in PBS (pH 7.4) at 25°C; the red solid line represents the best fit using a single exponential function. (H) Time‐dependent emissions of **Sel‐green** with different concentrations of **TD‐MSe1** in PBS (pH 7.4). Excitation: 370 nm. (I) Confocal fluorescence images of HeLa cells stained with **Sel‐green** (10 µM) for 20 min, and then incubated with **TD‐MSe1** (5 or 20 µM) for 30 min. Scale bar, 20 µm.

### 2.2 Rational Design of a Water‐Stable Methylselenocarbamate Scaffold

To develop more efficient releasing tools, we envisioned that intramolecular nucleophilic attack toward a water‐stable methylselenocarbamate would lead to the formation of a cyclization product [48, 49, 50], providing access to rapid liberation of the poor leaving selenol. We first assessed the water‐stability of *Se*‐methyl *N*‐phenylcarbamoselenoate (**2**) (Figure 2C) and found that it decomposed quickly in PBS (pH 7.4) with *t*_1/2_ of 9.4 min (Figure 2D and Figure S4). In addition, HRMS analysis revealed that **2** hydrolyzed to generate aniline. One explanation for this decomposition is that the selenocarbamate N–H would be deprotonated in PBS (pH 7.4) [51], resulting in isocyanate formation and CH_3_SeH release (Figure S4). This isocyanate would further hydrolyze to aniline. To investigate this hypothesis as well as to develop a water‐stable motif, we prepared *Se*‐methyl *N*‐phenyl‐*N*‐methylcarbamoselenoate (**3**) and assessed its stability in PBS (pH 7.4). HPLC analyses and absorbance spectra suggested that compound **3** was much more stable in buffers (Figure 2D and Figure S4E), implying that the *N*‐methylation should significantly inhibit the hydrolysis of **2**. We also employed a selenol‐selective fluorescence probe, **Sel‐green** [52], to monitor possible CH_3_SeH release from the selenocarbamates (Figure 2C). Time‐dependent emissions confirmed the CH_3_SeH release from **2** but not from **3** (Figure 2E and Figure S5). Moreover, **3** showed remarkable stability even in the presence of physiologically relevant nucleophiles including thiols and amines (Figure S6). Therefore, the selenocarbamate motif in **3** should be suitable for further donor design.

### 2.3 Engineering of a Highly Efficient Donation Platform

We next installed a Boc‐protected amino group adjacent to the *Se*‐methyl *N*‐phenyl‐*N*‐methylselenocarbamate (**4**) and further prepared **TD‐MSe1** with a protonated amine (Figure 2F). The deprotonation at pH 7.4 yielded a transient intermediate (TD‐MSe) that quickly underwent an intramolecular cyclization to form a non‐electrophilic byproduct **5** (Figure S7). In addition, the isolated yield of **5** from the spontaneous decomposition of **TD‐MSe1** in PBS was quantitative, supporting a highly efficient cyclization process. Subsequently, methyl(phenyl)carbamic chloride was employed to trap the released CH_3_SeH from **TD‐MSe1** in one pot, and the resultant product **3** was determined via HPLC analysis (Figure S8). The results demonstrated a CH_3_SeH release efficiency of up to 94.2 ± 2.7%, thereby validating TD‐MSe as a highly efficient platform for CH_3_SeH donation.

We further evaluated the cyclization kinetics using UV−vis experiments. The absorbance bands of **TD‐MSe1** at pH 4.5 and 7.4 are significantly different (Figure 2G), and the result at pH 7.4 is consistent with that of compound **5** (Figure S9), suggesting a very fast conversion from **TD‐MSe1** to **5** with *k*_obs_ of 0.14 s^–^^1^ (Figure 2G inset). In addition, we fitted the absorbance intensity curves to measure *k*_obs_ for each pH value to determine whether the amine or its protonated form was the active nucleophile (Figure S10 and Table 1). If the amino group is the active nucleophile in donor activation, we would expect to observe a positively correlated relationship between log(*k*_obs_) and pH value (Figure S11) [53]. As expected, the plot of log(*k*_obs_) versus pH value was linear, with more acidic conditions resulting in slower conversion, which is consistent with the deprotonated amino group being the active nucleophile. HPLC analyses further confirmed the pH‐dependent rapid conversion from **TD‐MSe1** to **5** (Figure S12). Altogether, the intramolecular amine can quickly attack the water‐stable selenocarbamate to result in rapid extrusion of CH_3_SeH under physiological pH.

**TABLE 1:** The *k*_obs_ and *t*_1/2_ for the conversion from **TD‐MSe1** to **5** under various pH values.

pH value	*k*_obs_ (1/s)	*t*_1/2_ (s)
4.5	2.61 × 10^−4^	2654
5.0	8.78 × 10^−4^	789
5.5	2.58 × 10^−3^	269
6.0	7.98 × 10^−3^	87
6.5	0.02	35
7.0	0.06	12
7.4	0.14	5

We then employed an electrophilic reagent (iodoacetamide) to successfully trap the released CH_3_SeH from **TD‐MSe1** in PBS buffers in one pot (Figure S13). In addition, the probe **Sel‐green** could be used to monitor the CH_3_SeH released from **TD‐MSe1** by fluorescence spectra as well as by naked eye under a 365 nm UV lamp (Figure S14). The emissions at 515 nm increased with higher concentrations of **TD‐MSe1** under physiological buffers (Figure 2H), supporting the CH_3_SeH‐releasing ability of the donor. HRMS analysis of a solution of **TD‐MSe1** and **Sel‐green** identified the expected **2a**, **5**, and CH_3_SeH‐trapped products (Figure S14). Taken together, the TD‐MSe motif should be highly efficient for the CH_3_SeH release under physiological conditions.

Encouraged by the above results, we further investigated whether **TD‐MSe1** could be used for intracellular CH_3_SeH delivery. Briefly, HeLa cells were pre‐treated with 10 µM probe **Sel‐green** for 20 min and then incubated with donor **TD‐MSe1** (0–20 µM) for 30 min. As expected, negligible fluorescence was observed in the probe‐only treated cells, and a concentration‐dependent intracellular fluorescence increase was observed in the donor‐treated cells (Figure 2I). In addition, time‐dependent bioimaging results of the cells treated with **TD‐MSe1** further supported the intracellular CH_3_SeH delivery (Figure S15). In contrast, using **3** instead of **TD‐MSe1** could not trigger the intracellular fluorescence from **Sel‐green**. Therefore, the TD‐MSe platform should be useful for controlled CH_3_SeH donation in live cells.

### 2.4 Validation of the Caged TD‐MSe Platform for Light‐Triggered CH_3_SeH Release

To create stable CH_3_SeH donors under physiological conditions for triggerable delivery, the TD‐MSe platform was caged by an *o*‐nitrobenzyl moiety via a carbamate linkage to prepare **TD‐MSe2** (Figure 3A). HPLC analysis revealed that **TD‐MSe2** remained stable in PBS (pH 7.4) (Figure 3B and Figure S16), even in the presence of physiologically relevant concentrations of thiols (Figure S17), supporting the remarkable stability of the caged TD‐MSe motif before activation.

**FIGURE 3:**
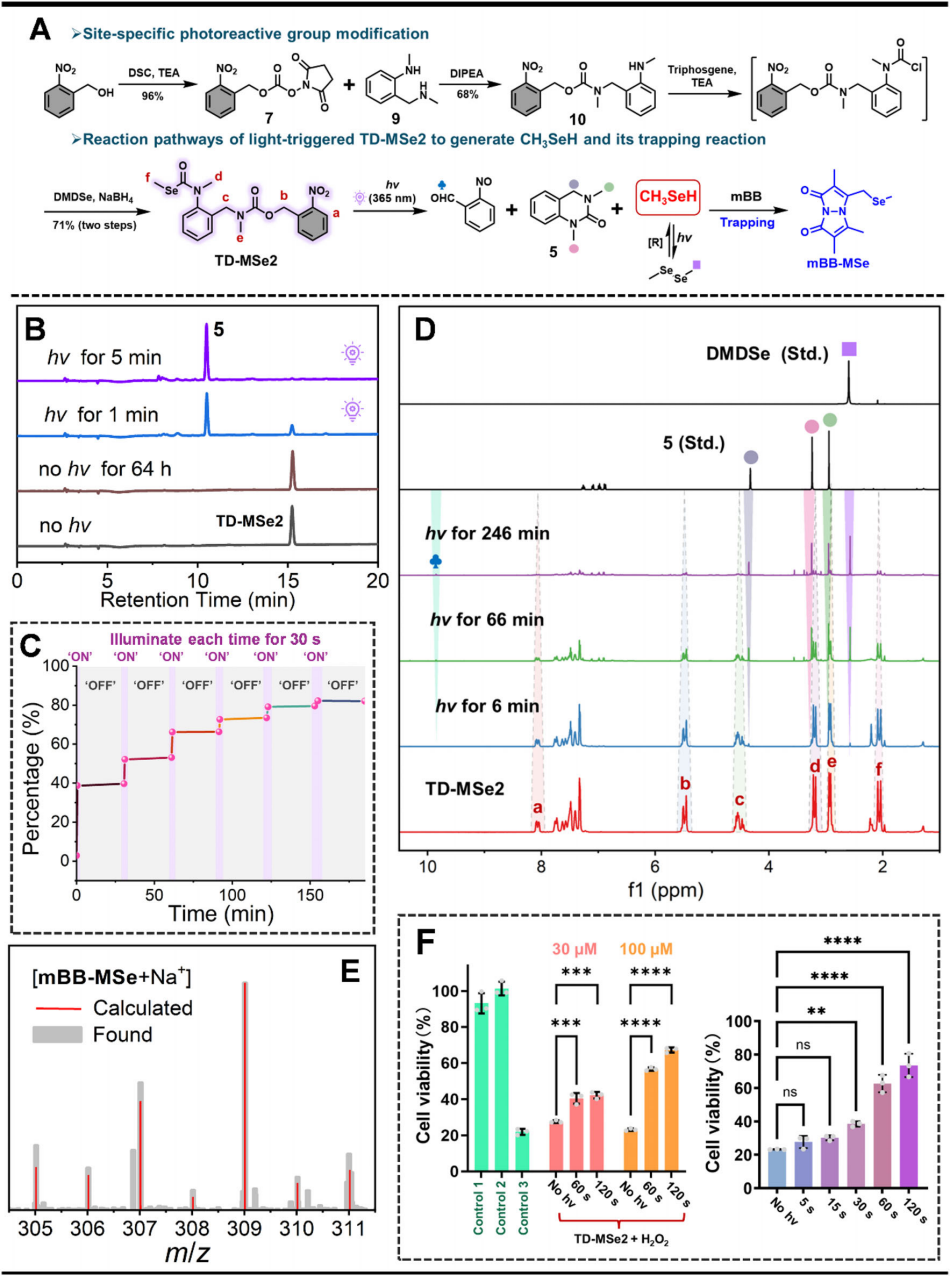
Developing a light‐triggered donor for CH_3_SeH delivery and cellular protection under oxidative stress. (A) Synthesis of **TD‐MSe2** and its light‐triggered reaction pathways to generate **5** and CH_3_SeH, as well as the trapped reaction to generate mBB‐MSe. (B) Time‐dependent HPLC analysis of **TD‐MSe2** in PBS (pH 7.4) without or with 365 nm irradiation. (C) The release efficiency of **5** from **TD‐MSe2** under “light” and “dark” conditions in PBS (pH 7.4). (D) Time‐dependent ^1^H NMR of **TD‐MSe2** in CD_3_CN under different irradiation times. (E) HRMS for the mBB‐trapped CH_3_SeH released from light‐triggered **TD‐MSe2**. Calculated for C_11_H_14_N_2_NaO_2_Se^+^: 309.0113; found: 309.0108. (F) Effects of different treatments on HeLa cell viability. Left panel: The three control groups include cells treated with 150 µM **TD‐MSe2** (control 1), cells pretreated with 150 µM **TD‐MSe2** followed by 120 s light irradiation (control 2), and cells treated with 400 µM H_2_O_2_ (control 3). The pink and orange groups correspond to cells pretreated with 30 µM and 100 µM **TD‐MSe2**, exposed to 365 nm light for various durations, and subsequently treated with 400 µM H_2_O_2_. Right panel: Cells pretreated with 150 µM **TD‐MSe2** at different light exposure times followed by 400 µM H_2_O_2_ treatment. Data are shown as mean ± SD (n = 3; ns: not significant; ^**^ *p* < 0.01; ^***^ *p* < 0.001; ^****^ *p* < 0.0001).

After irradiation activation, the donor **TD‐MSe2** is expected to generate **5** and to release CH_3_SeH, and both time‐dependent HPLC and UV−vis experiments suggested light‐triggered decomposition of **TD‐MSe2** in PBS buffers (Figure 3B and Figure S18). In addition, the expected byproducts **5** and 2‐nitrosobenzaldehyde from the light‐triggered **TD‐MSe2** were identified by HRMS analysis. We also used excess 4‐bromomethylbenzyl alcohol to trap and confirm the CH_3_SeH released from light‐activated **TD‐MSe2** in one pot (Figure S18). These results imply that **TD‐MSe2** should be a light‐triggered CH_3_SeH donor.

To demonstrate the spatiotemporal control of the release, we further carried out the light on‐off experiments [54] with **TD‐MSe2** under alternating “light” and “dark” conditions (Figure S19). The light‐triggered release of product **5** could be quantified by HPLC analysis (Figure S19). As shown in Figure 3C, under the “dark” condition, the photo‐uncaging process completely ceased, while the photolysis only proceeded under the “light on” condition. Therefore, the donation reaction is selectively controlled by light, and moreover, the release efficiency of **5** could reach over 80% after the photolysis (Figure 3C).

To provide mechanistic insights, we also used NMR spectroscopy to monitor the irradiation‐dependent activation of **TD‐MSe2** in CD_3_CN solution. We could identify the characteristic peaks in irradiation‐dependent ^1^H NMR spectra for the generation of **5**, 2‐nitrosobenzaldehyde and dimethyldiselenide (DMDSe) via comparison with the standard compounds (Figure 3D). Prolonging the irradiation time resulted in gradual disappearance of the **TD‐MSe2** peaks as well as the appearance of the products’ peaks. Because selenols can be rapidly oxidized to diselenides under irradiation conditions [54], ^13^C{^1^H} and ^77^Se{^1^H} NMR spectra further demonstrated a clear irradiation‐dependent increase of DMDSe (Figures S20 and S21). Moreover, monobromobimane (mBB), a trapping reagent for selenol [55], was used to successfully trap the CH_3_SeH released from the light‐activated donor in the presence of 1,4‐dithiothreitol, and the characteristic Se isotopic patterns are consistent with the calculated mass distribution (Figure 3E). Building on these studies, **TD‐MSe2** is highly efficient for light‐triggered CH_3_SeH/DMDSe release with a well‐defined releasing mechanism.

Inspired by Xian's preeminent work on Se‐based catalytic scavengers to eliminate ROS [56], we envisioned that the light‐triggered CH_3_SeH donor might alleviate oxidative stress. We then evaluated the antioxidant activity of **TD‐MSe2** in live cells. A Cell Counting Kit‐8 (CCK‐8) assay suggested that **TD‐MSe2** without or with 120 s UV (365 nm) irradiation following 2 h of incubation with HeLa cells exhibited negligible cytotoxicity (Figure 3F). In antioxidant studies, incubating HeLa cells with 400 µM H_2_O_2_ for 2 h significantly reduced the cell viability to near 22%. When HeLa cells were first treated with 30 or 100 µM **TD‐MSe2** for 2 h and then irradiated for 120 s, prior to the incubation with H_2_O_2_, the cell viability increased to near 42 and 67 percent, respectively. In contrast, the negative control without irradiation did not provide cellular protection against the H_2_O_2_‐induced damage. We further found that longer photoactivation of the donor provided more obvious cellular protection against H_2_O_2_‐induced oxidative stress (Figure 3F). These results support that the CH_3_SeH/DMDSe released from **TD‐MSe2** should be responsible for the cytoprotective abilities. It is noteworthy that HeLa cells contain millimolar endogenous GSH [57], which can reduce DMDSe to CH_3_SeH easily. In living cells, CH_3_SeH can react with ROS to generate CH_3_SeOH, and a catalytic cycle may be achieved by the reaction of GSH and CH_3_SeOH to regenerate CH_3_SeH [58], promoting efficient ROS elimination. To support the ROS‐scavenging activity of CH_3_SeH, we further used a commercially available ROS probe (2′,7′‐dichlorofluorescin diacetate) to detect the intracellular ROS levels, and as expected, cells treated with **TD‐MSe2** with or without irradiation showed negligible or significant fluorescence, respectively (Figure S22). To the best of our knowledge, **TD‐MSe2** represents the first light‐triggered CH_3_SeH donor, which should be capable of mimicking the GR activity to enhance ROS scavenging in live cells [56, 58].

### 2.5 Development of a Biomarker‐Triggered CH_3_SeH Donor for Cell‐Selective Therapeutic Delivery

To demonstrate the general applicability of the caged TD‐MSe platform, we chose a “trimethyl‐locked” quinone trigger that can be removed by a tumor‐associated oxidoreductase (hNQO1). hNQO1 is over‐expressed in some types of metastatic cells, including the human colorectal adenocarcinoma cell line (HT‐29) [59, 60, 61]. We created the first example of hNQO1‐triggered CH_3_SeH donor **TD‐MSe3** for cell‐selective delivery (Figures 4 and 5). UV−vis experiments suggested that **TD‐MSe3** was stable in PBS (pH 7.4) over 70 h (Figure S23). Upon treatment with hNQO1 in the presence of nicotinamide adenine dinucleotide (NADH), the donor was activated to generate the expected products **5** and **11**, which were identified by HRMS analysis (Figure S24). In addition, time‐dependent HPLC analysis suggested the fast enzyme‐triggered reaction of **TD‐MSe3** to generate **11** followed by **5** (Figure 4B and Figure S24), supporting the uncaging of the trigger and the formation of **5** in tandem. Furthermore, the release efficiency of **11** from **TD‐MSe3** after hNQO1 reduction exceeded 90% (Figure S25), implying a highly efficient enzyme‐activated process.

**FIGURE 4:**
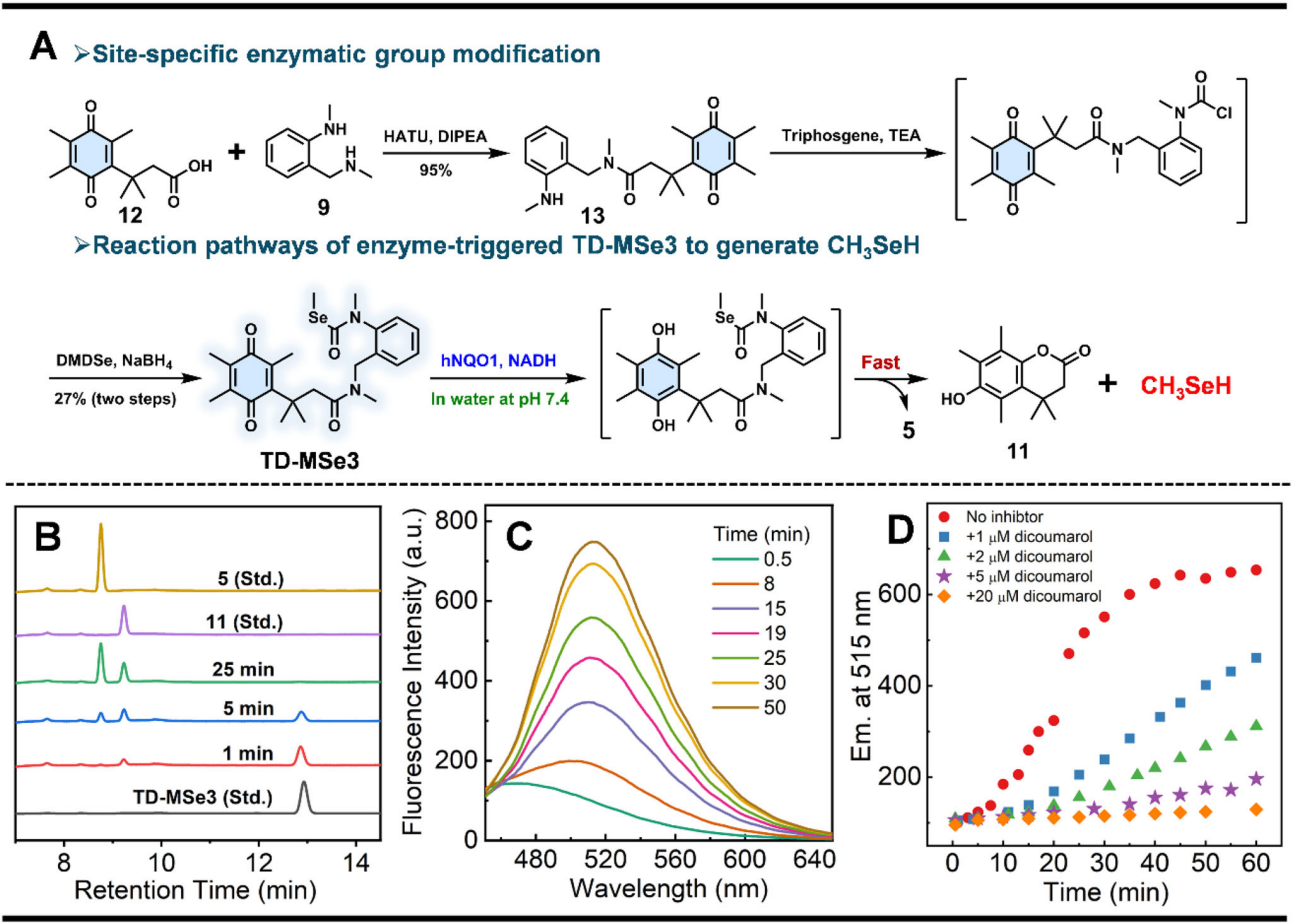
Developing an enzyme‐triggered donor for controlled CH_3_SeH release. (A) Synthesis of **TD‐MSe3** and its reaction pathways with hNQO1 to generate CH_3_SeH. (B) Time‐dependent HPLC traces for the enzymatic reaction of **TD‐MSe3** in PBS buffer. (C) Fluorescence spectra of **Sel‐green** (10 µM) with the hNQO1‐activated **TD‐MSe3** (50 µM). Excitation: 370 nm. (D) Time‐dependent emission at 515 nm of **Sel‐green** (10 µM) with the enzymatic reaction of **TD‐MSe3** (50 µM) in the presence of different concentrations of dicoumarol.

**FIGURE 5:**
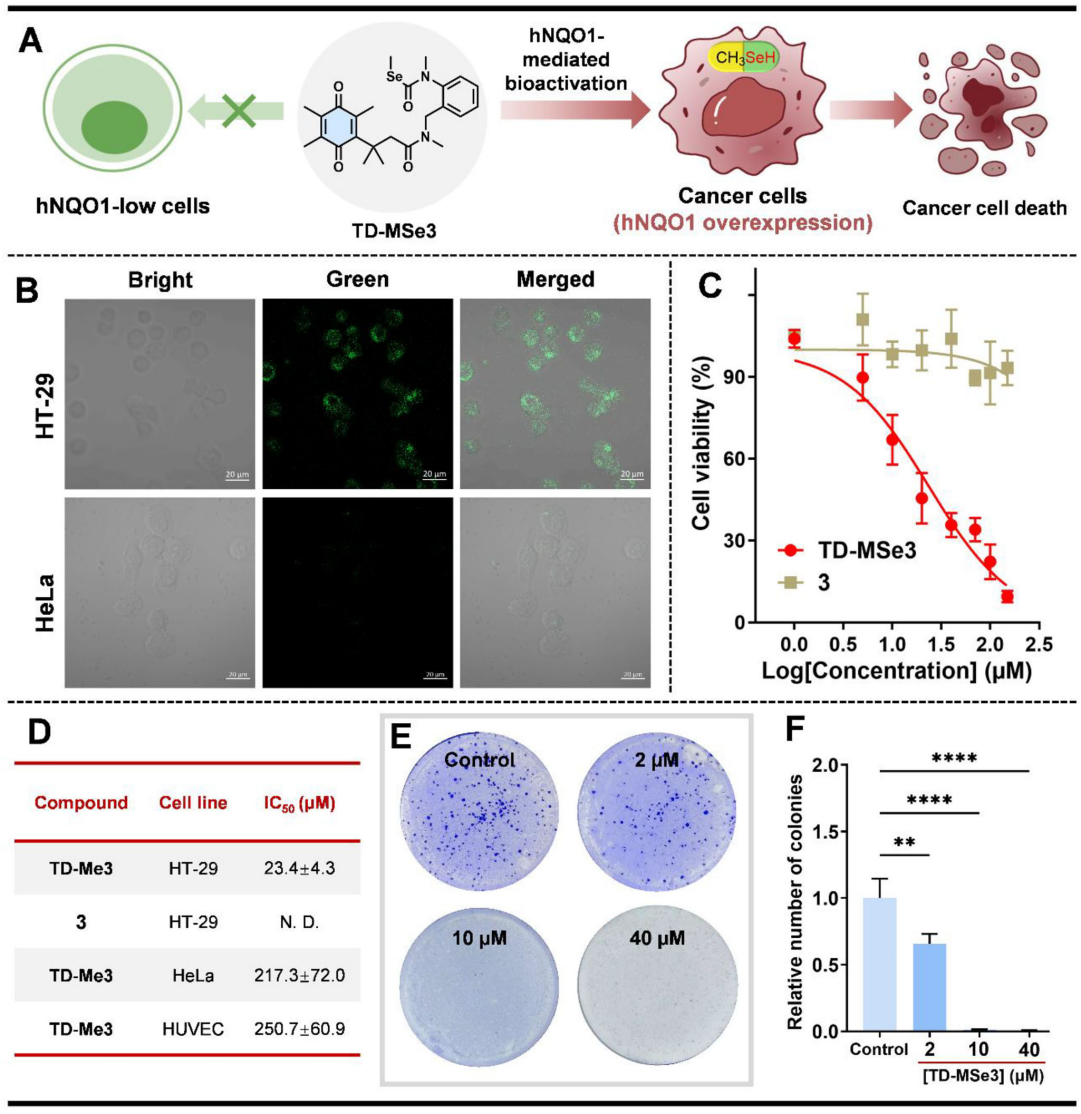
Cancer cell‐selective activation of **TD‐MSe3** for CH_3_SeH‐mediated anticancer activity. (A) Schematic illustration of hNQO1‐selective bioactivation of **TD‐MSe3** for CH_3_SeH‐mediated cancer cell killing. (B) Confocal fluorescence images of HT‐29 and HeLa cells stained with **Sel‐green** (10 µM) and **TD‐MSe3** (20 µM). Scale bar, 20 µm. (C) Relative viability of HT‐29 cells after chemical treatments with different concentrations of **TD‐MSe3** or **3**. The data were expressed as mean ± SD (n = 3). Solid lines represent the nonlinear regression fits for *IC*_50_ determination. (D) The *IC*_50_ data of **TD‐MSe3** for different cell lines. (E) Representative images of colonies of HT‐29 cells with different concentrations of **TD‐MSe3**. (F) Relative number of colony formation results in (E). Data are presented as mean ± SD (n = 3, ^**^ *p* < 0.01, ^****^ *p* < 0.0001).

The released CH_3_SeH from hNQO1‐triggered **TD‐MSe3** was successfully trapped by mBB in one pot (Figure S26). We also confirmed the CH_3_SeH release from the enzyme‐triggered **TD‐MSe3** by measuring the time‐dependent fluorescence response of **Sel‐green** (Figure 4C and Figure S27). Such enzyme‐triggered CH_3_SeH release can be inhibited by the hNQO1 inhibitor dicoumarol [62] in a concentration‐dependent manner (Figure 4D and Figure S27). These results confirm the enzyme‐triggered **TD‐MSe3** for controlled CH_3_SeH donation in physiological buffers.

We then investigated the intracellular CH_3_SeH release from **TD‐MSe3** mediated by endogenous hNQO1 (Figure 5A). First of all, we evaluated the hNQO1 levels in three different cell lines using an hNQO1 fluorescent probe [61] and found that HT‐29 cells had high hNQO1 expression while HeLa and HUVEC cells had low hNQO1 expression (Figure S28). Accordingly, HT‐29 cells were selected for subsequent imaging experiments: upon co‐treatment with both probe **Sel‐green** and donor **TD‐MSe3**, the HT‐29 cells displayed pronounced intracellular fluorescence (Figure 5B), implying that endogenous hNQO1 effectively activates **TD‐MSe3** to release CH_3_SeH intracellularly. When cells were pre‐incubated with dicoumarol, the intracellular fluorescence was substantially diminished, further confirming that the intracellular release of CH_3_SeH from **TD‐MSe3** is hNQO1‐dependent (Figure S29). In contrast, HeLa cells, which possess low basal hNQO1 expression [61], exhibited negligible fluorescence compared with HT‐29 cells (Figure 5B). The results suggest that the intracellular activation of **TD‐MSe3** is governed by endogenous hNQO1 levels and thus displays intrinsic cell selectivity.

To assess anticancer effects of the enzyme‐triggered CH_3_SeH donor, we examined the cytotoxicity of **TD‐MSe3** in HT‐29 cells (Figure 5C). After 72 h of incubation, **TD‐MSe3** exhibited a potent effect on cell viability (*IC*_50_ = 23.4 µM), whereas control **3** showed no appreciable cytotoxicity at concentrations up to 150 µM. In contrast, **TD‐MSe3** displayed substantially higher *IC*_50_ values (> 200 µM) in hNQO1‐low cell lines of both HeLa and HUVEC cells (Figure 5D and Figure S30). Our preliminary data suggest that the CH_3_SeH release from the donor is crucial for the anticancer effect, and the therapeutic potential of **TD‐MSe3** should have cellular selectivity toward hNQO1‐overexpressing cancer types, matching the above bioimaging results.

Subsequently, we further evaluated the long‐term anti‐proliferative effect of **TD‐MSe3** using a colony formation assay (Figure 5E). Notably, **TD‐MSe3** inhibited the HT‐29 cell proliferation in a concentration‐dependent manner (Figure 5F). At 2 µM, the number of tumor colonies was reduced by more than 35% relative to the untreated control, and the colony formation was almost completely abolished at 10 µM. These findings collectively demonstrate that **TD‐MSe3** effectively suppresses the clonogenic survival of hNQO1‐overexpressing cancer cells in vitro, thereby underscoring its potential as a promising hNQO1‐activated antitumor candidate.

## 3 Conclusion

In summary, we have reported the first general responsive platform for harnessing CH_3_SeH in vitro and in live cells. We found that the classical self‐immolative chemistry is too slow to release CH_3_SeH (*t*_1/2_ = 73 h) and then identified *Se*‐methyl *N*‐phenyl‐*N*‐methylcarbamoselenoate as a stable motif under physiological conditions. We further rationally designed an adjacent amino (nucleophilic) group for fast intramolecular activation of the water‐stable methylcarbamoselenoate, developing a highly efficient platform (TD‐MSe) for controlled release of CH_3_SeH. Moreover, the caged TD‐MSe motifs were successfully used to create the first examples of light‐ and hNQO1‐triggered CH_3_SeH donors, and these donors were successfully applied for intracellular CH_3_SeH delivery (Table S1). Importantly, the hNQO1‐triggered donor could be activated by an endogenous biomarker for killing metastatic cancer cells selectively. We anticipate that the new donation chemistry will significantly promote the development of advanced selenol donors, further advancing Se‐related biomedical research [63, 64, 65].

## 4 Experimental Section

The synthesis methods of all new compounds as well as compound characterizations are described in detail in the Supporting Information. We used HPLC analysis, HRMS/NMR spectra, and UV absorption/fluorescence experiments to evaluate the release performance of the synthesized donors. For cell imaging, HeLa cells were first incubated with the probe (10 µM) for 20 min, and then the probe‐treated cells were further incubated with the donor (0, 5, 20 µM) for 30 min. Images were captured using a confocal microscope (Olympus FV1000). To verify the intracellular CH_3_SeH release from the enzyme‐triggered donor, both HT‐29 and HeLa cells were treated with **TD‐MSe3** (20 µM) and the CH_3_SeH‐responsive probe **Sel‐green** (10 µM) for 2 h, and fluorescence imaging was subsequently acquired using Axio‐Imager_LSM‐800. The cytotoxicity of the donors and control molecules was evaluated using the CCK‐8 assay. HeLa cells were used to evaluate the irradiation time‐dependent antioxidant activity of the light‐triggered donor **TD‐MSe2**. HT‐29, HeLa, and HUVEC cells were incubated with different concentrations of **TD‐MSe3** for 72 h to evaluate the anticancer effects. For the colony formation assay, HT‐29 cells (3 × 10^3^ cells per well) were incubated with culture medium containing the enzyme‐triggered donor at different concentrations (0, 2, 10, or 40 µM), and the medium was refreshed every three days. After 19 days of culture, colonies were fixed with 4% paraformaldehyde at 4°C for 15 min and then stained with 0.1% crystal violet for 5 min at room temperature. The plates were then imaged, and colony numbers were quantified using ImageJ software.

## Author Contributions

**Wenfang Liang**: methodology, Writing – review and editing, Writing – original draft, data curation, software, validation, investigation. **Liyue Wang**: methodology, writing – review and editing, software. **Xuan Wang**: methodology, software, writing – review and editing. **Xinran Li**: methodology, software, writing – review and editing. **Hao Xing**: methodology, software, writing – review and editing. **Lu Sun**: methodology, writing – original draft, writing – review and editing, funding acquisition, supervision, software. **Zhen Xi**: writing – review and editing, project administration, funding acquisition, supervision. **Long Yi**: writing – original draft, writing – review and editing, conceptualization, project administration, funding acquisition, supervision, methodology, data curation, software, investigation, validation, formal analysis.

## Funding

This work is supported by the National Natural Science Foundation of China (22377007), the Science & Technology Development Fund of Tianjin Education Commission for Higher Education (2023ZD019), Beijing Municipal Natural Science Foundation and the Frontiers Science Center for New Organic Matter, Nankai University (63181206).

## Conflicts of Interest

The authors declare no conflicts of interest.

## Supporting information




**Supporting File 1**: advs78356‐sup‐0001‐SuppMat1.pdf.

## Data Availability

The data that supports the findings of this study are available in the supplementary material of this article.
