# Supplementary material for: Harnessing Methylselenol (CH3SeH): A Highly Effective Platform for Stimuli‐Responsive Delivery With Therapeutic Potential
Source: Adv Sci (Weinh). 2026 Oct 18:e78356. Online ahead of print. doi: 10.1002/advs.78356 (PMC13677048; doi:10.1002/advs.78356)
Supplement: Supplementary file 1 [file ADVS-9999-e78356-s001.pdf]

Supporting Information

Harnessing Methylselenol (CH_3SeH): A Highly Effective Platform for Stimuli-Responsive Delivery with Therapeutic Potential

Wenfang Liang[1], Liyue Wang[2], Xuan Wang[2], Xinran Li[2], Hao Xing[2], Lu Sun[2], Zhen Xi[3], and Long Yi[1]**

[1]College of Chemical Engineering and Beijing Key Laboratory of Bioprocess, Beijing University of Chemical Technology, Beijing 100029, China

[2]Tianjin Key Laboratory on Technologies Enabling Development of Clinical Therapeutics and Diagnostics (Theranostics), School of Pharmacy, Tianjin Medical University, Tianjin, 300070, China.

[3]National Pesticide Engineering Research Center (Tianjin), Department of Chemical Biology, State Key Laboratory of Elemento-Organic Chemistry, College of Chemistry, Frontiers Science Center for New Organic Matter, Nankai University, Tianjin 300071, China.

*Corresponding Author(s): Long Yi; Lu Sun; Email Address: yilong@buct.edu.cn; sunlu@tmu.edu.cn

Table of contents

1. Supporting Figures and Table 2

2. General Chemicals and Instruments 26

3. Experimental Methods 27

4. Detailed Synthetic Procedures and Characterization Data for All Compounds .. 35

5. Supporting NMR, IR and HRMS Spectra 42

6. SI References 71

1. Supporting Figures

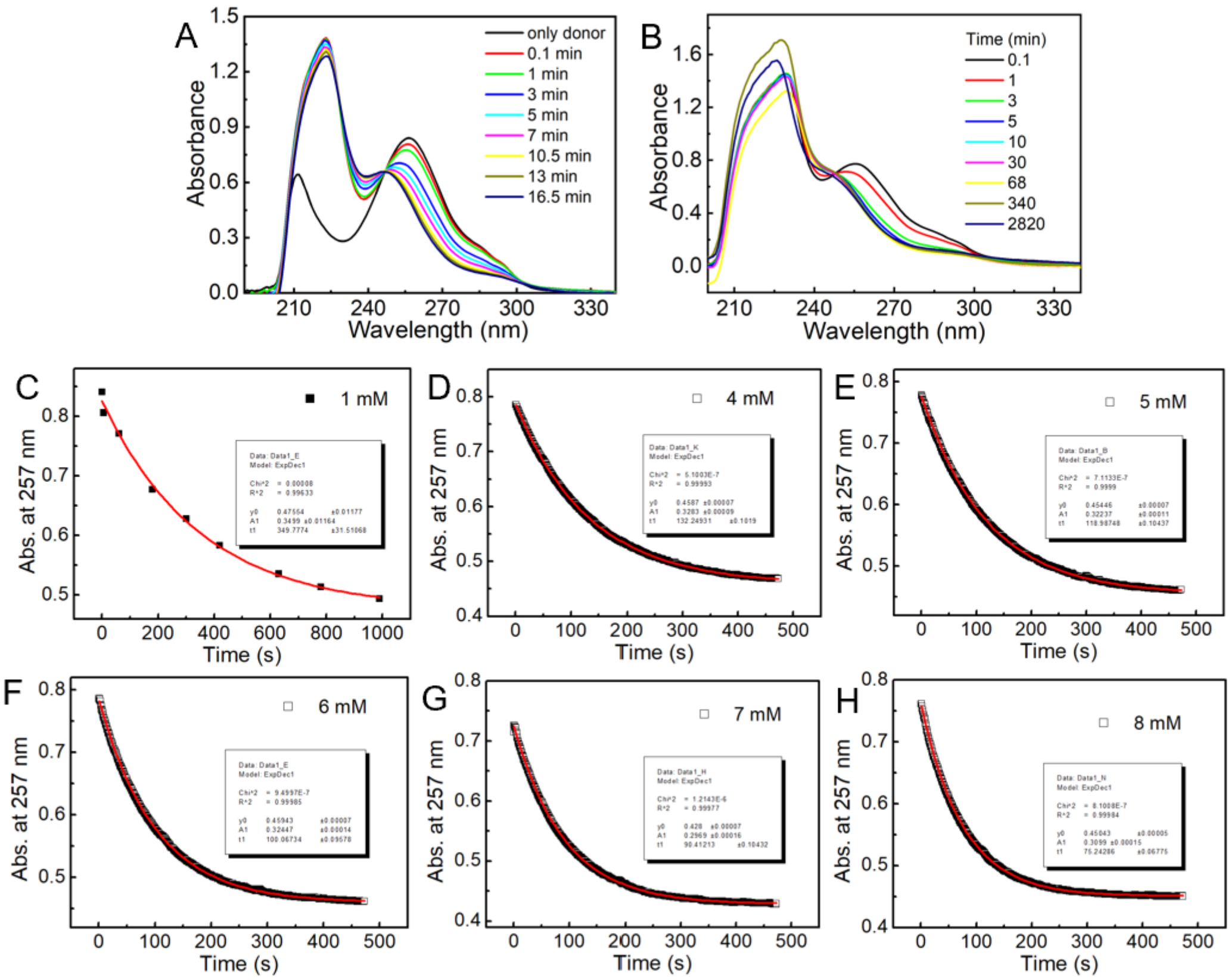

Figure S1. (A, B) Time-dependent UV–vis spectra of **1** (50 μM) with 1 mM (A) or 5 mM (B) TCEP in PBS (pH 7.4, containing 30% CH_3CN). (C) Time-dependent intensities at 257 nm from (A). (D–H) Time-dependent intensities at 257 nm of **1** (50 μM) in PBS buffers with 4 mM (D), 5 mM (E), 6 mM (F), 7 mM (G) or 8 mM (H) TCEP. Each red solid red line in (C–H) represents the best fit using a single-exponential function to give k_{obs}.

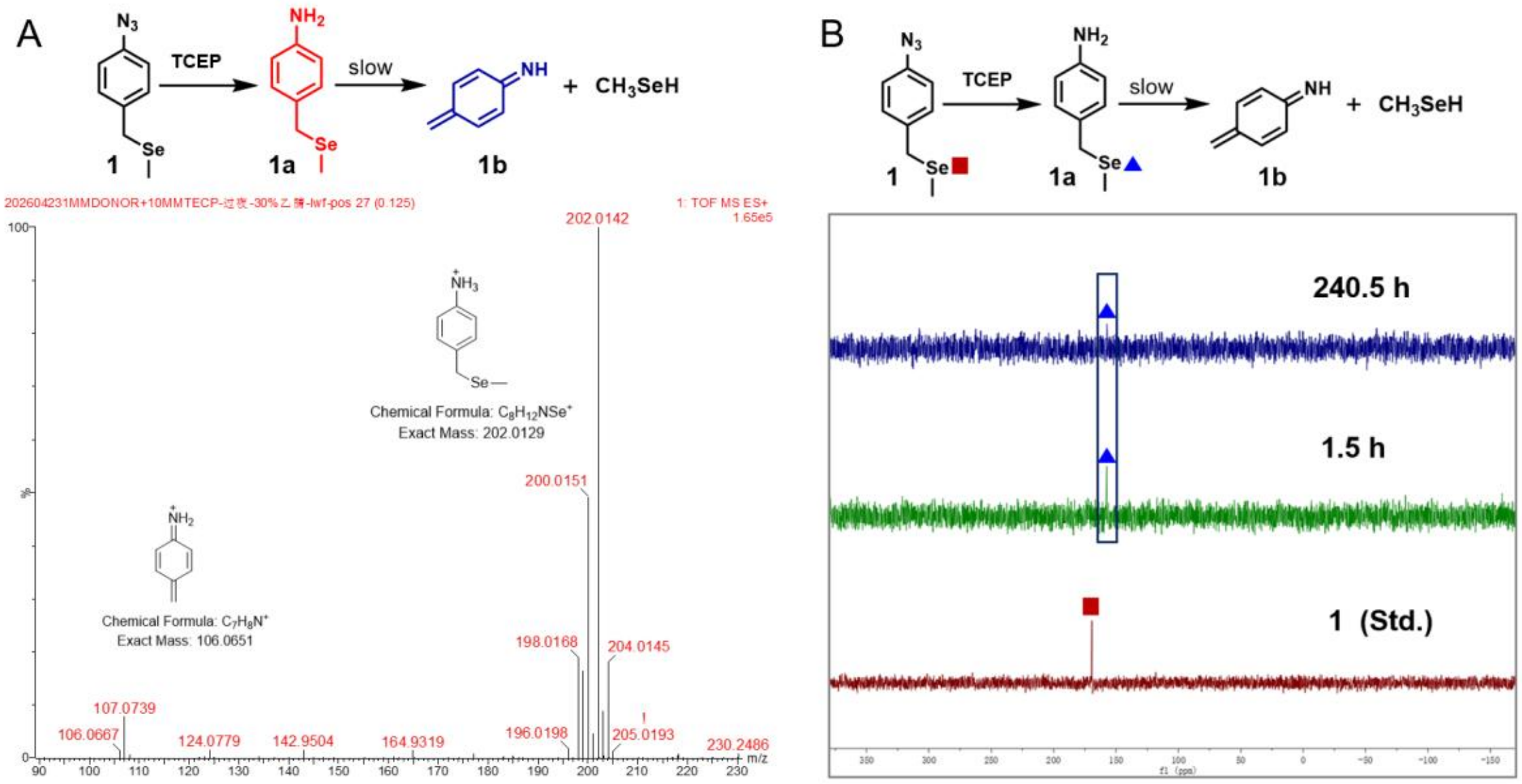

Figure S2. (A) HRMS analysis of the reaction solution of **1** (1 mM) incubated with TCEP (10 mM) overnight in PBS buffer (50 mM, pH 7.4, containing 30% CH_3CN). The possible reaction pathway is given. (B) Representative time-dependent $^{77}Se\{^1H\}$ NMR for the reaction of **1** (50 mM) and TCEP (100 mM). The key ^{77}Se peaks are marked by colored symbols.

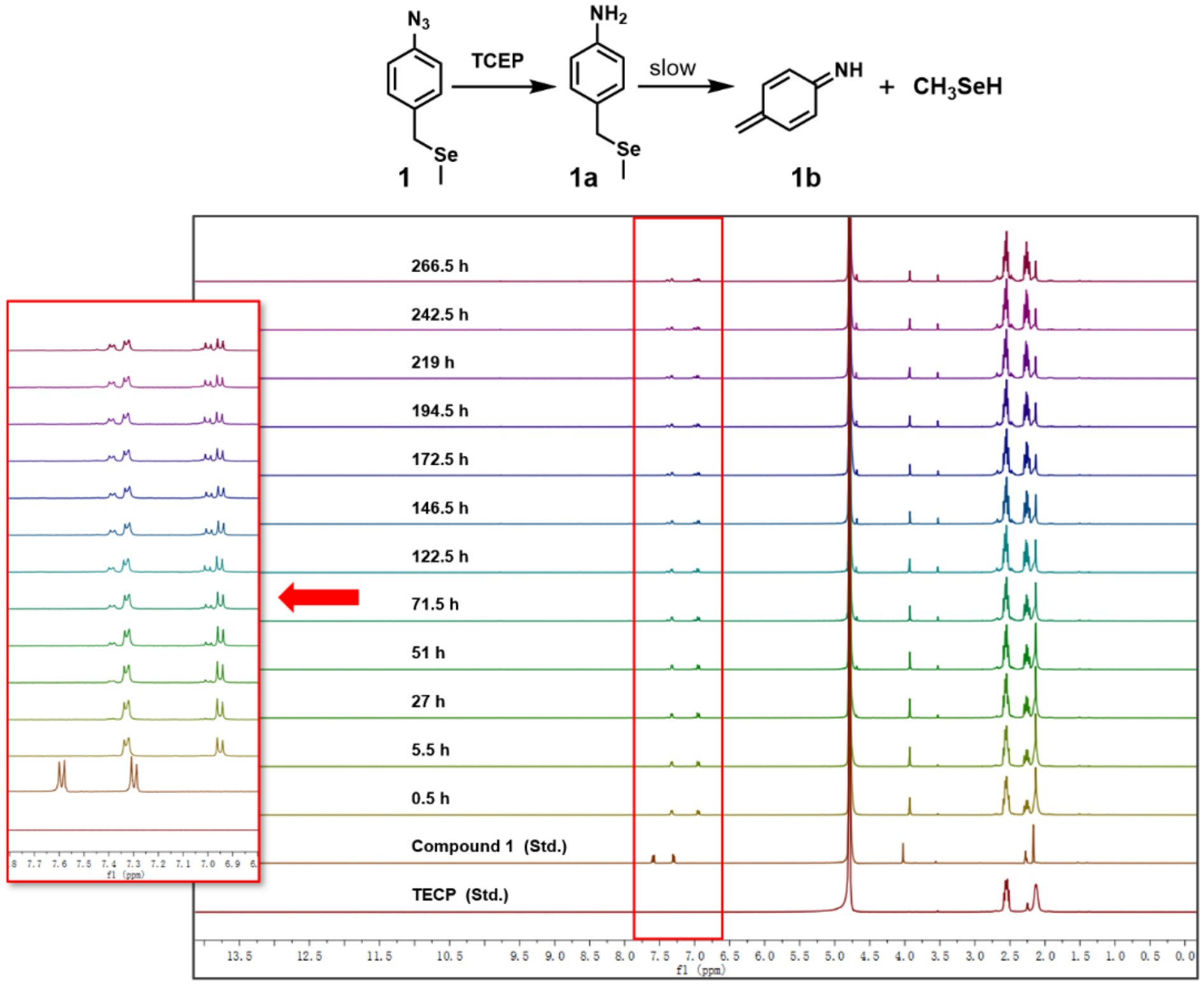

Figure S3. Representative time-dependent ^{1}H NMR for the reaction of **1** (5 mM) and TCEP (25 mM). Inset: partial magnified view of ^{1}H NMR spectra. The results suggest that compound **1** is rapidly reduced to **1a** within initial 0.5 h, and the subsequent self-immolative process is very slow.

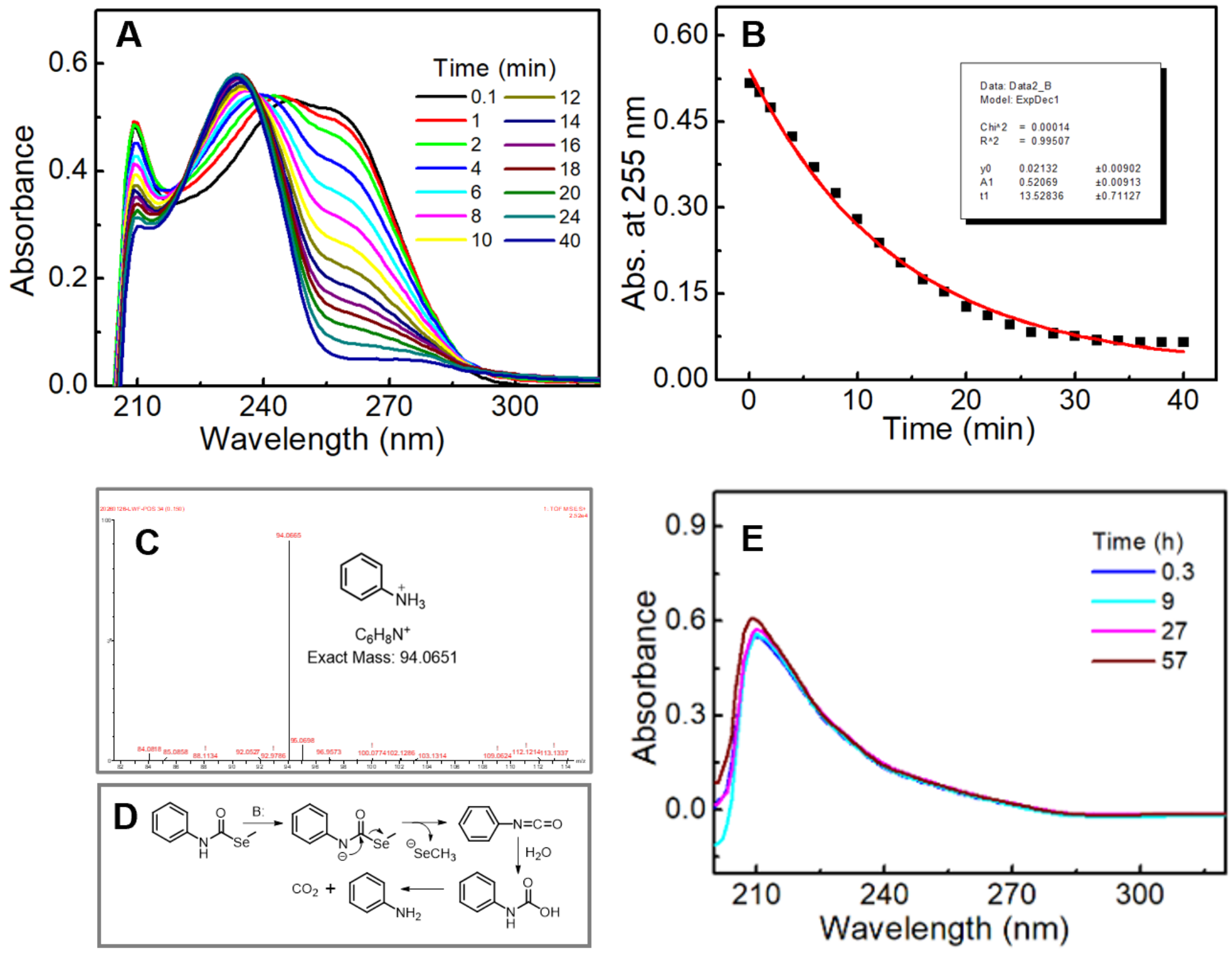

Figure S4. (A) Time-dependent UV–vis spectra of **2** (50 μM) in PBS buffer (pH 7.4, containing 10% CH_3CN) at 25 °C. (B) Time-dependent intensities at 255 nm from (A) were fitted by a single-exponential function to give k_{obs}. (C) HRMS analysis for the reaction solution of **2** (5 mM) in PBS buffer (50 mM, pH 7.4, containing 50% CH_3CN) after 1.5 h of incubation. (D) Proposed hydrolysis pathway for **2**. (E) Time-dependent absorbance spectra of **3** (50 μM) in PBS at room temperature. The results imply that compound **3** should be stable in PBS for over 57 h.

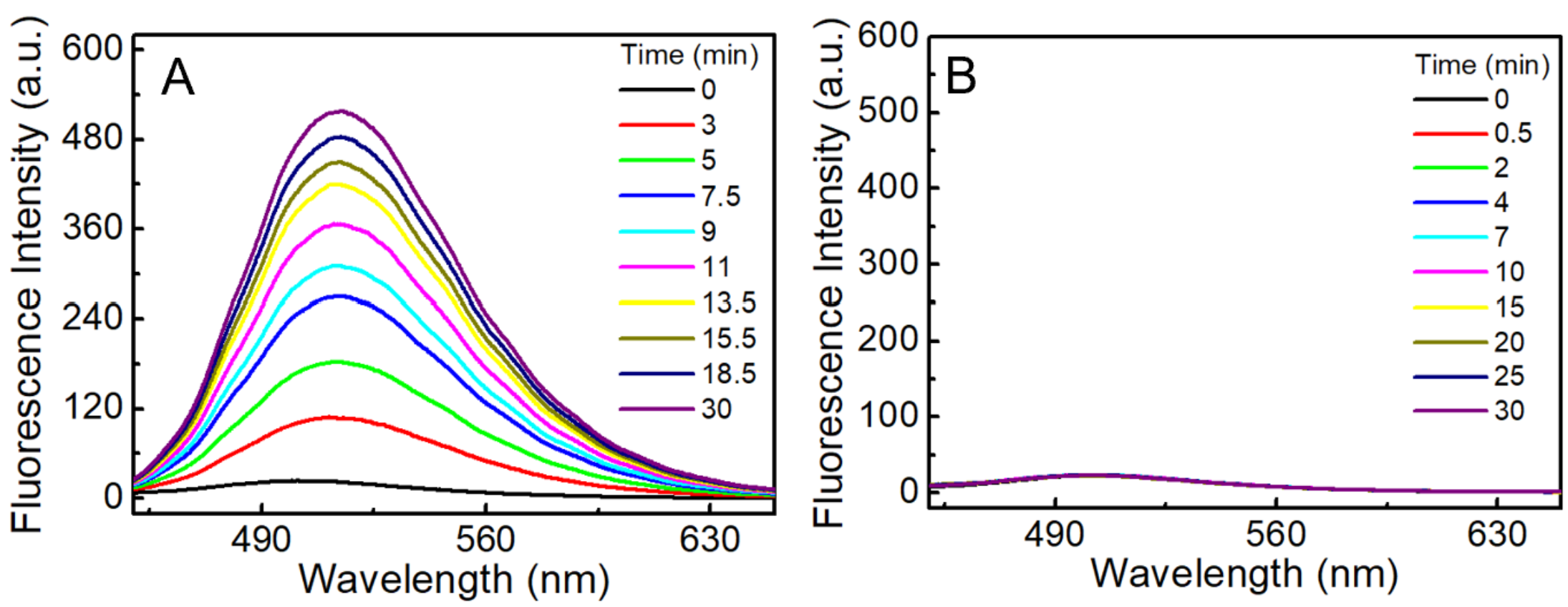

Figure S5. Time-dependent fluorescence spectra of **Sel-green** (10 μM) with 20 μM **2** (A) or **3** (B) in PBS (pH 7.4, containing 10% CH_3CN). Excitation at 370 nm. The results imply that compound **2** can release CH_3SeH but compound **3** should be stable in PBS.

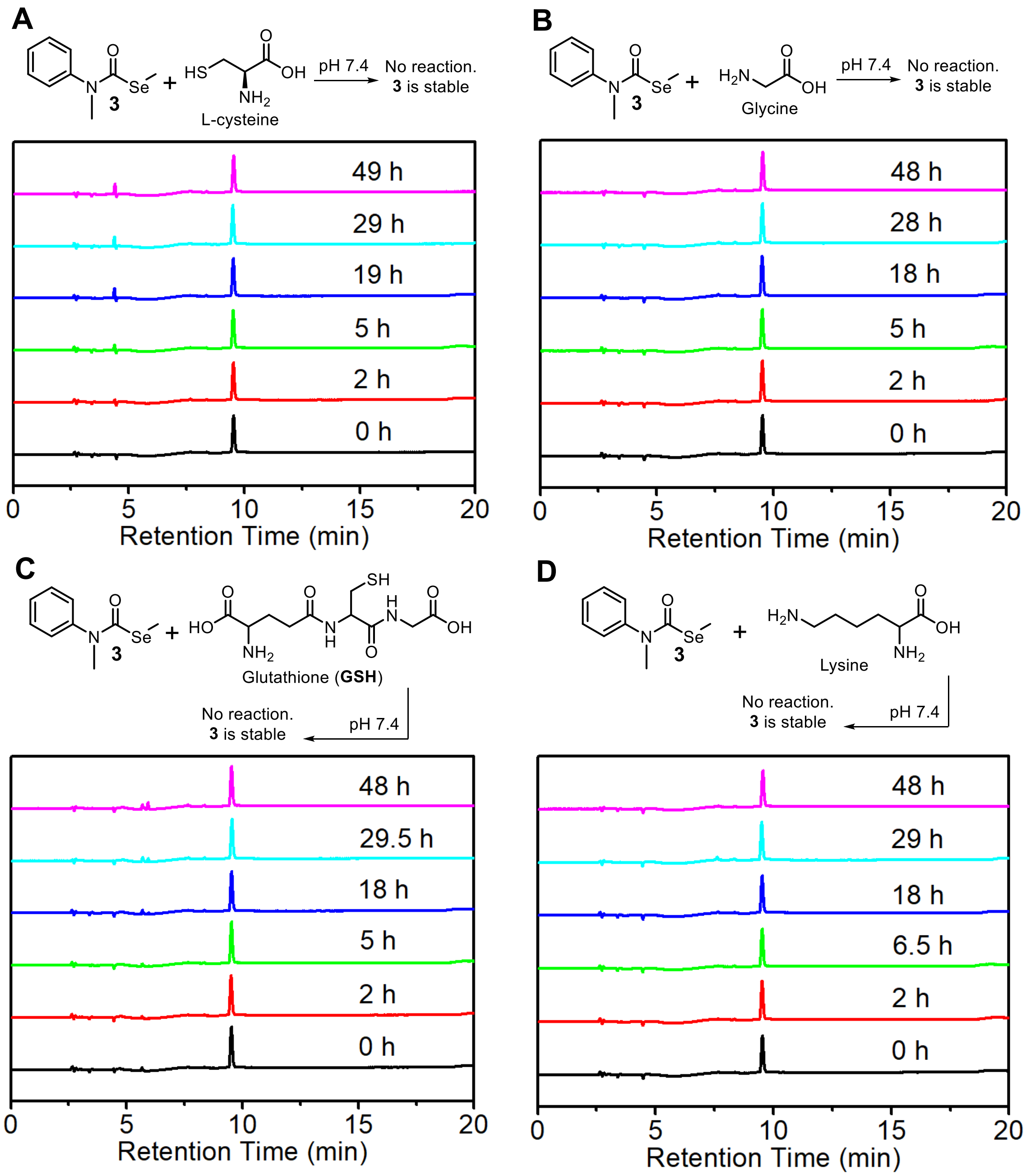

Figure S6. Time-dependent HPLC traces of **3** (0.5 mM) in PBS buffer (pH 7.4, containing 30% CH_3CN) with 0.2 mM Cys (A), 1 mM Gly (B), 5 mM GSH (C), or 1 mM Lys (D) at room temperature. Detection wavelength: 254 nm. The results showed that >95% of **3** was stable after 48 h of incubation in the presence of Lys, and >99% of **3** was stable after 48 h of incubation in the presence Cys, Gly, or GSH.

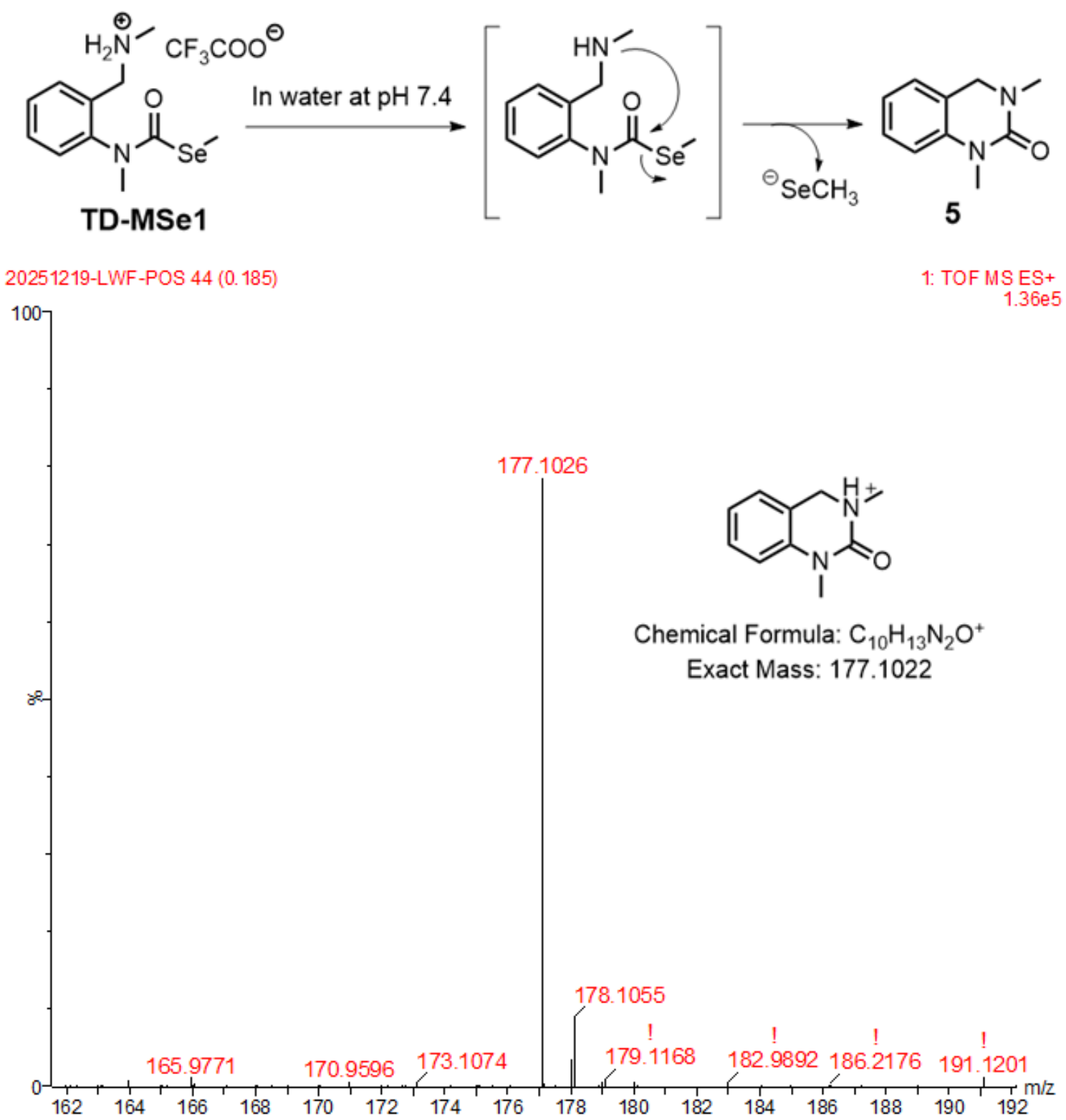

Figure S7. HRMS analysis for the reaction solution of **TD-MSe1** (5 mM) in PBS buffer (50 mM, pH 7.4, containing 50% CH_3CN).

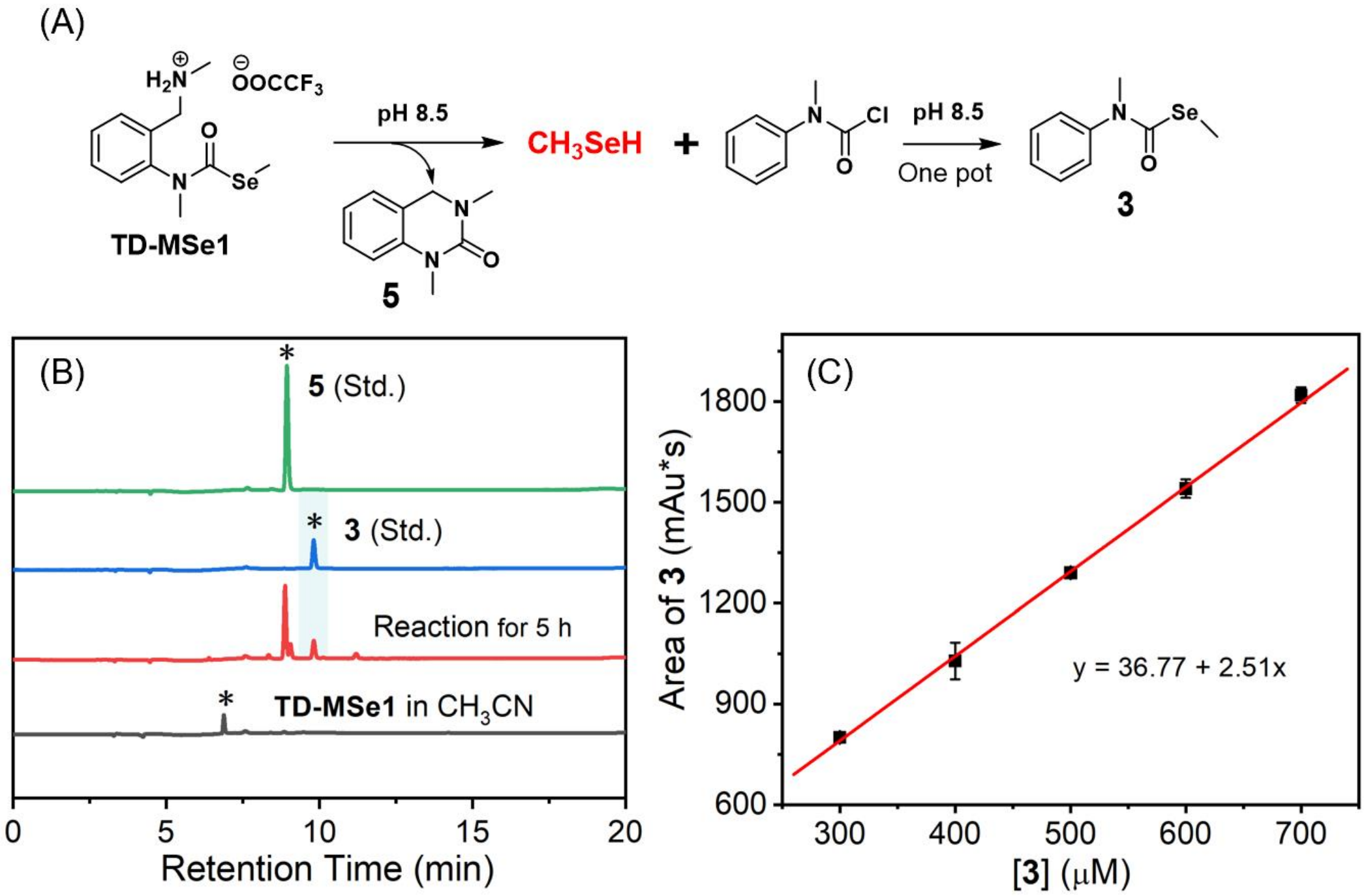

Figure S8. (A) Reaction pathways of **TD-MSe1** to release CH_3SeH and then to trap it via methyl(phenyl)carbamoyl chloride in one pot. (B) HPLC trace of the one-pot reaction mixture of **TD-MSe1** (330 μM) and methyl(phenyl)carbamoyl chloride (2 mM) in PBS (pH 8.5, containing 50% CH_3CN and 1 mM dithiothreitol) for 5 h. The control traces of pure **TD-MSe1** in CH_3CN, **5** or **3** in PBS buffer (pH 8.5, containing 50% CH_3CN) are also shown. Detection wavelength: 254 nm. (C) HPLC calibration curve of the peak areas of **3** versus its concentrations. The results are expressed as mean ± SD (n = 3).

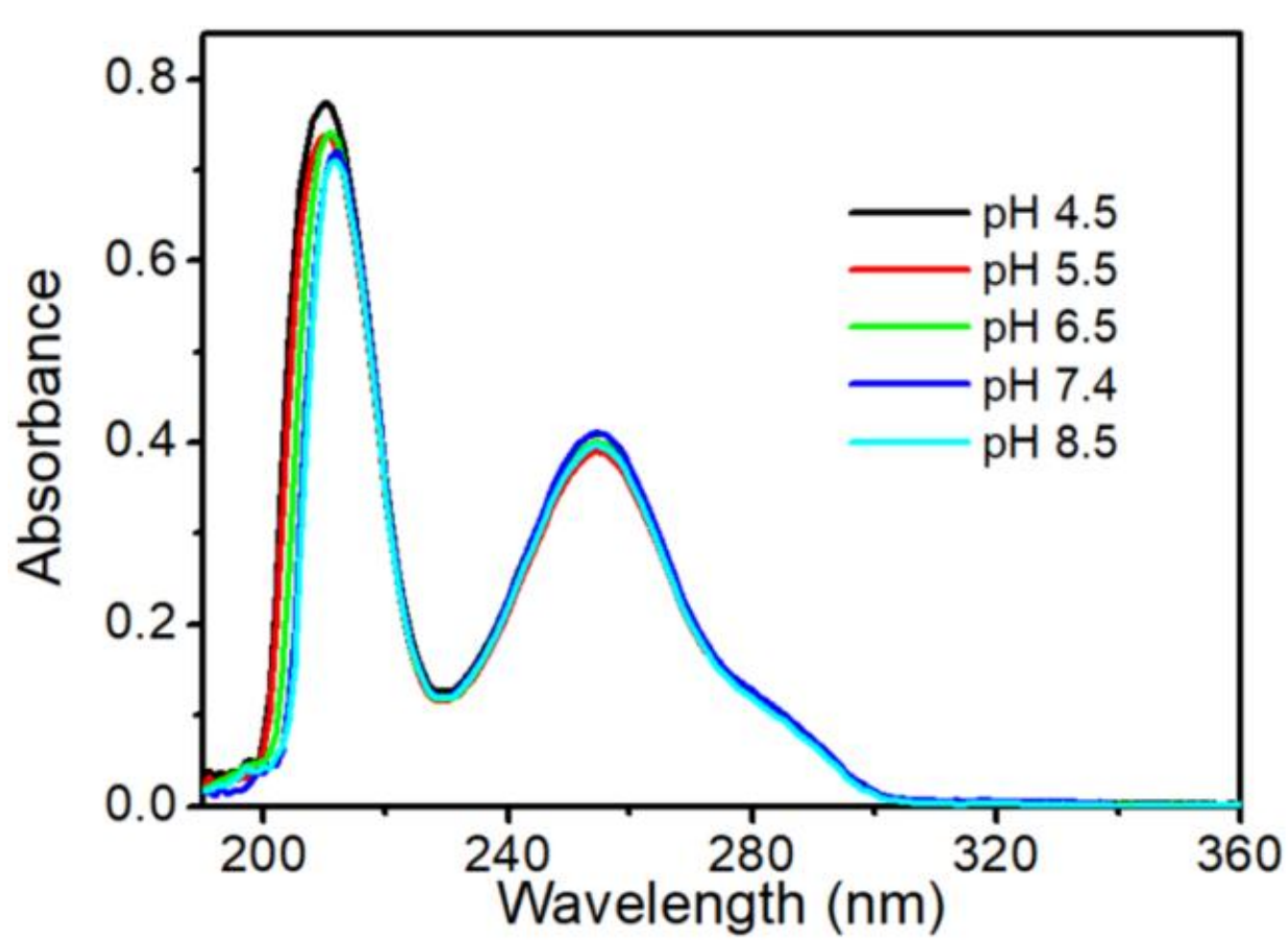

Figure S9. UV–vis spectra of **5** (50 μM) in PBS buffer (containing 10% CH_3CN) at different pH values. The results imply that the tested pH values do not influence the absorbance of **5**.

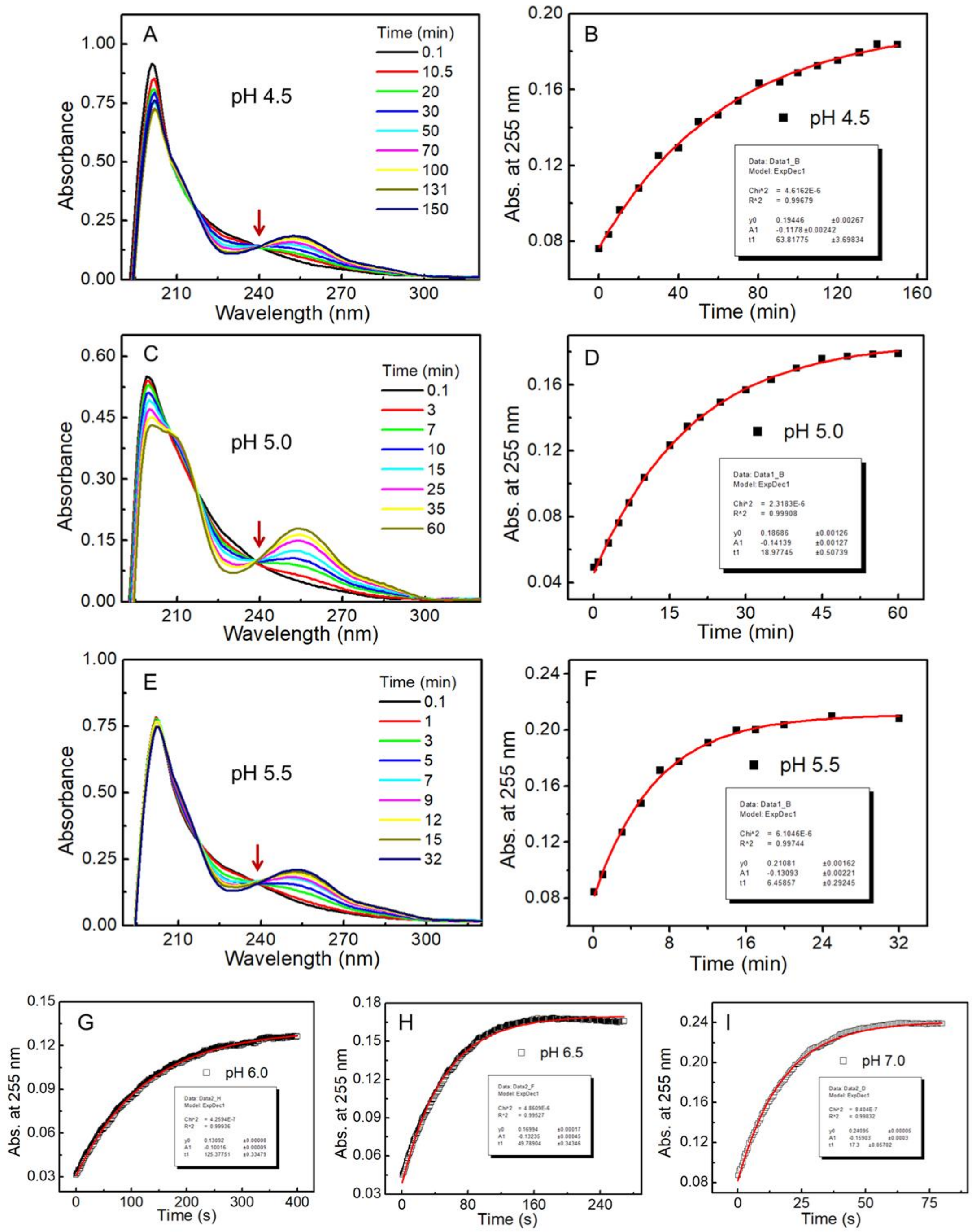

Figure S10. (A) Time-dependent UV–vis spectra of **TD-MSe1** (30 μM) in PBS (pH 4.5, containing 10% CH_3CN). (B) Time-dependent intensities at 255 nm from (A). (C) Time-dependent UV–vis spectra of **TD-MSe1** (30 μM) in PBS (pH 5.0). (D) Time-dependent intensities at 255 nm from (C). (E) Time-dependent UV–vis spectra of **TD-MSe1** (30 μM) in PBS (pH 5.5). (F) Time-dependent intensities at 255 nm from (E). (G–I) Time-dependent intensities at 255 nm of **TD-MSe1** (30 μM) in PBS buffers at different pH values (6.0 (G), 6.5 (H), 7.0 (I)). Each red solid line in (B, D, F, G–I) represents the best fit using a single-exponential function to give k_{obs}.

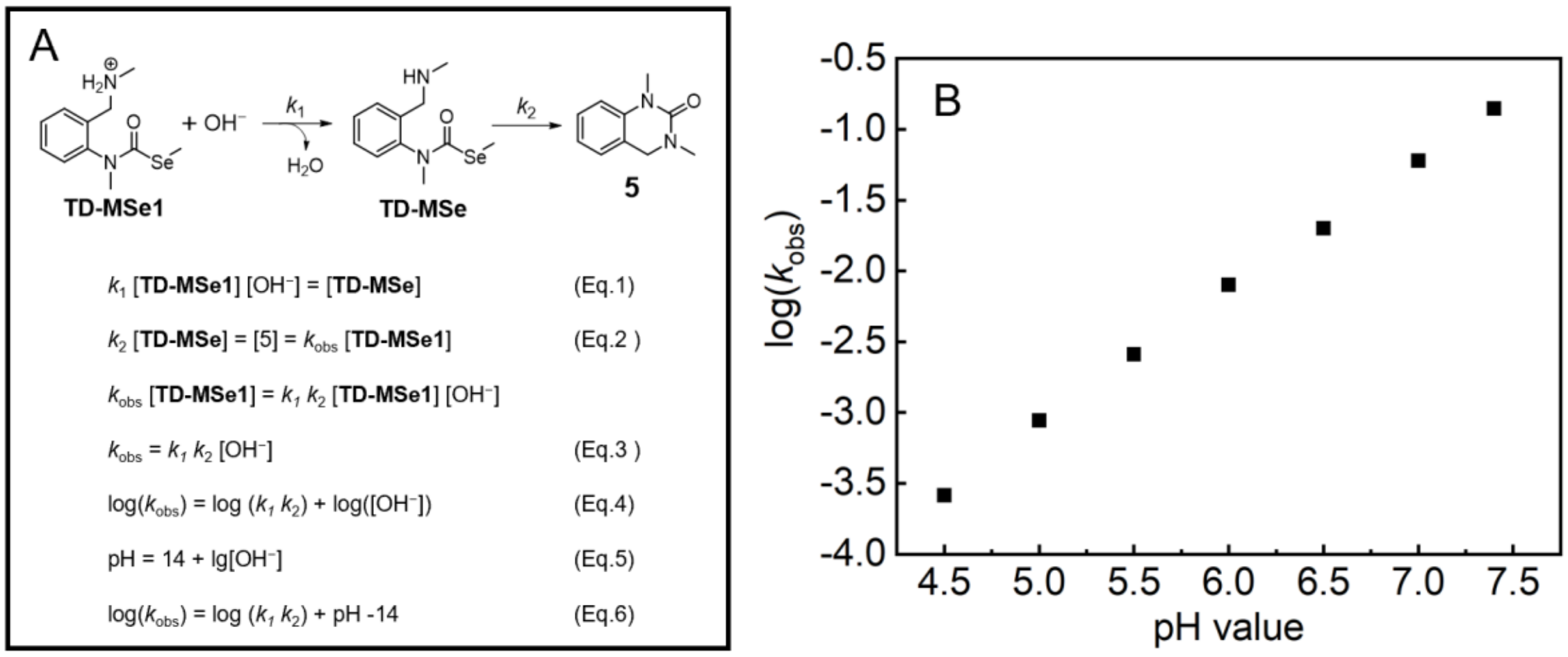

Figure S11. (A) Theoretical derivation of the linear correlation between log(k_{obs}) and pH. In Eq. 6, k_1 and k_2 are both constants, and if the amino group acts as an active nucleophile in the donor activation, the plot of log(k_{obs}) versus pH should be linearly related. (B) Plot of log(k_{obs}) versus pH, and the linear correlation implies that the deprotonated amino group is the active nucleophile.

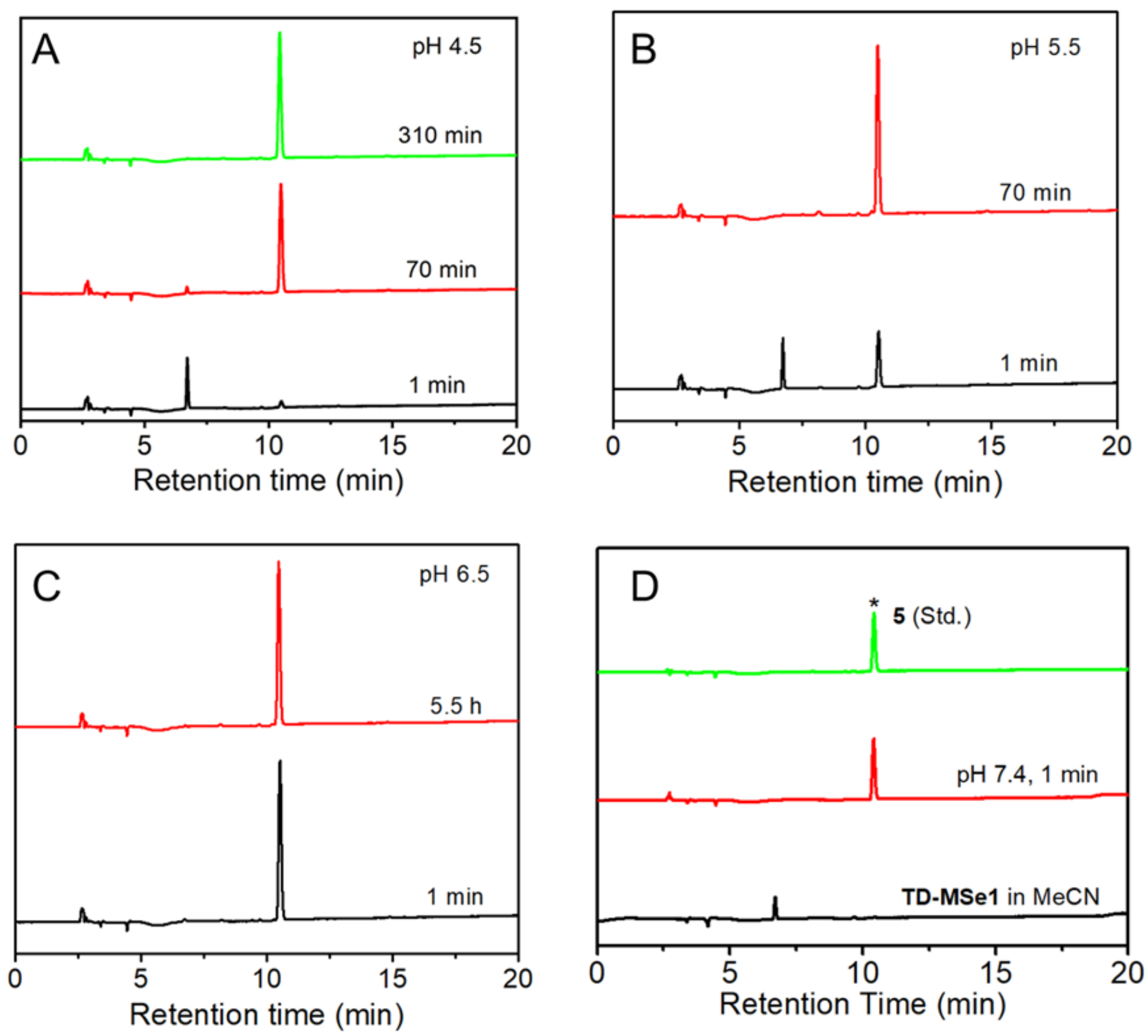

Figure S12. Time-dependent HPLC traces of **TD-MSe1** (0.5 mM) in different PBS buffers (pH 4.5 (A), pH 5.5 (B), pH 6.5 (C) and pH 7.4 (D)). Detection wavelength: 254 nm. The results imply that all reactions at these pH values generated **5**, and the reaction could complete within 1 min at pH 7.4.

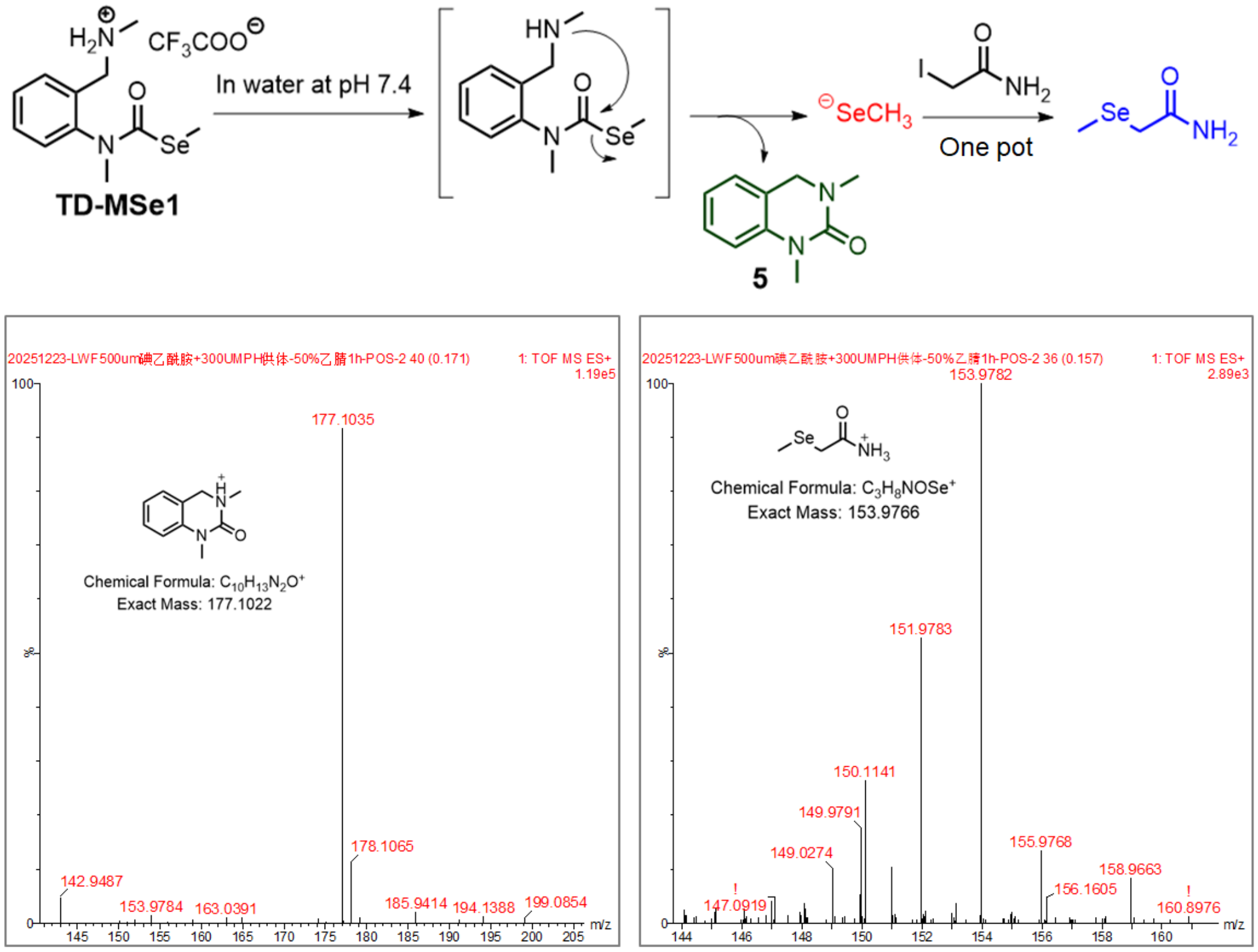

Figure S13. HRMS analysis for the one-pot reaction of **TD-MSe1** (0.3 mM) and iodoacetamide (0.5 mM) in PBS buffer (50 mM, pH 7.4, containing 50% CH_3CN) for 1 h. The possible reaction pathway for the trapped CH_3SeH product is given**.**

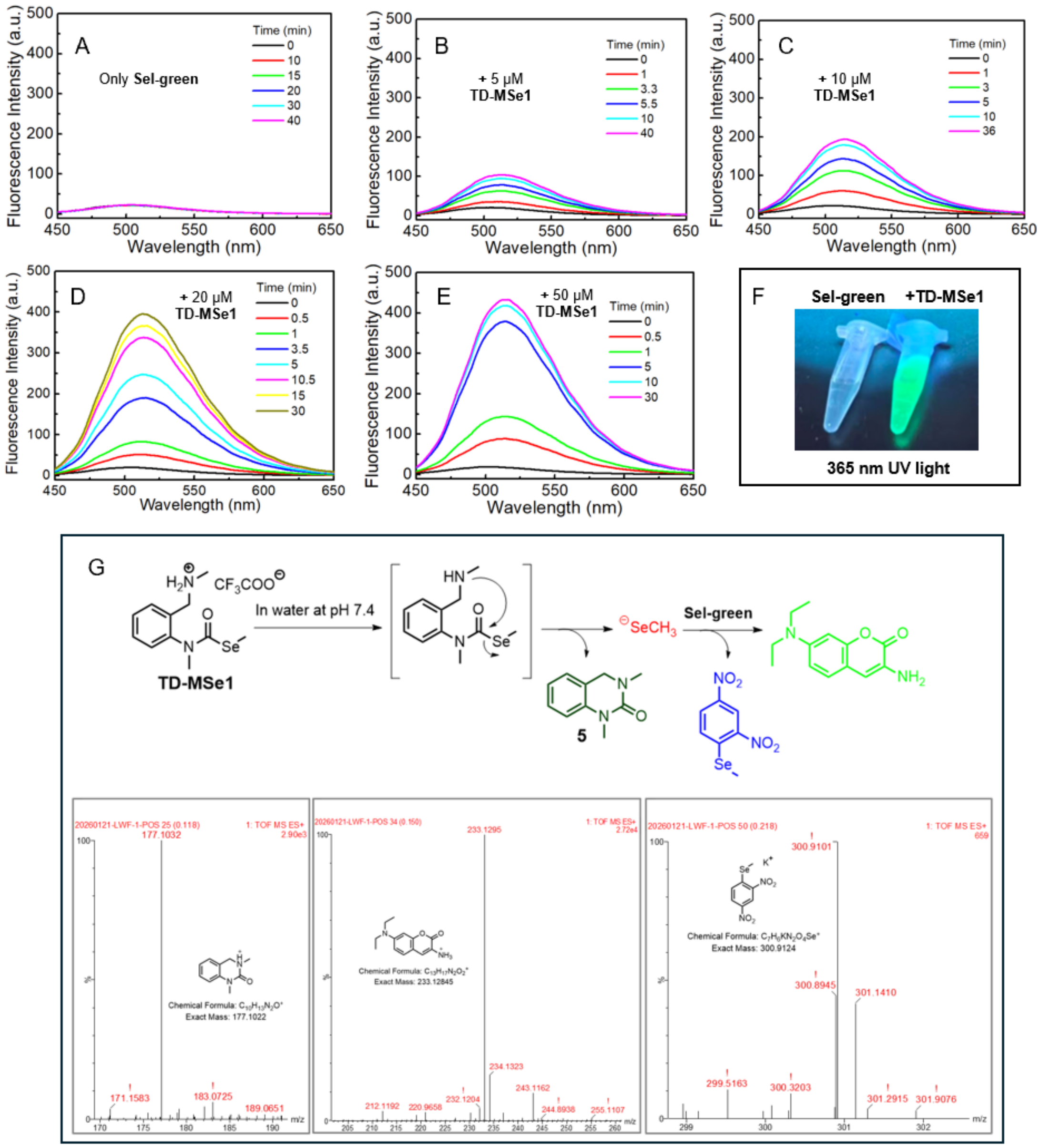

Figure S14. (A–E) Time-dependent fluorescence spectra of **Sel-green** (10 µM) without (A) or with different concentrations of **TD-MSe1** (5 µM (B), 10 µM (C), 20 µM (D) and 50 µM (E)) in PBS (pH 7.4, containing 10% CH_3CN) at 25 °C. Excitation, 370 nm. (F) Photo under 365 nm UV lamp for the solutions of **Sel-green** (10 µM) without or with **TD-MSe1** (100 µM) in PBS (pH 7.4, containing 10% CH_3CN) after 15 min of incubation. (G) HRMS analysis for the one-pot reaction of **TD-MSe1** (0.5 mM) and **Sel-green** (0.5 mM) in PBS buffer (50 mM, pH 7.4, containing 50% CH_3CN) for 0.5 h. The possible reaction pathway for these expected products is given**.**

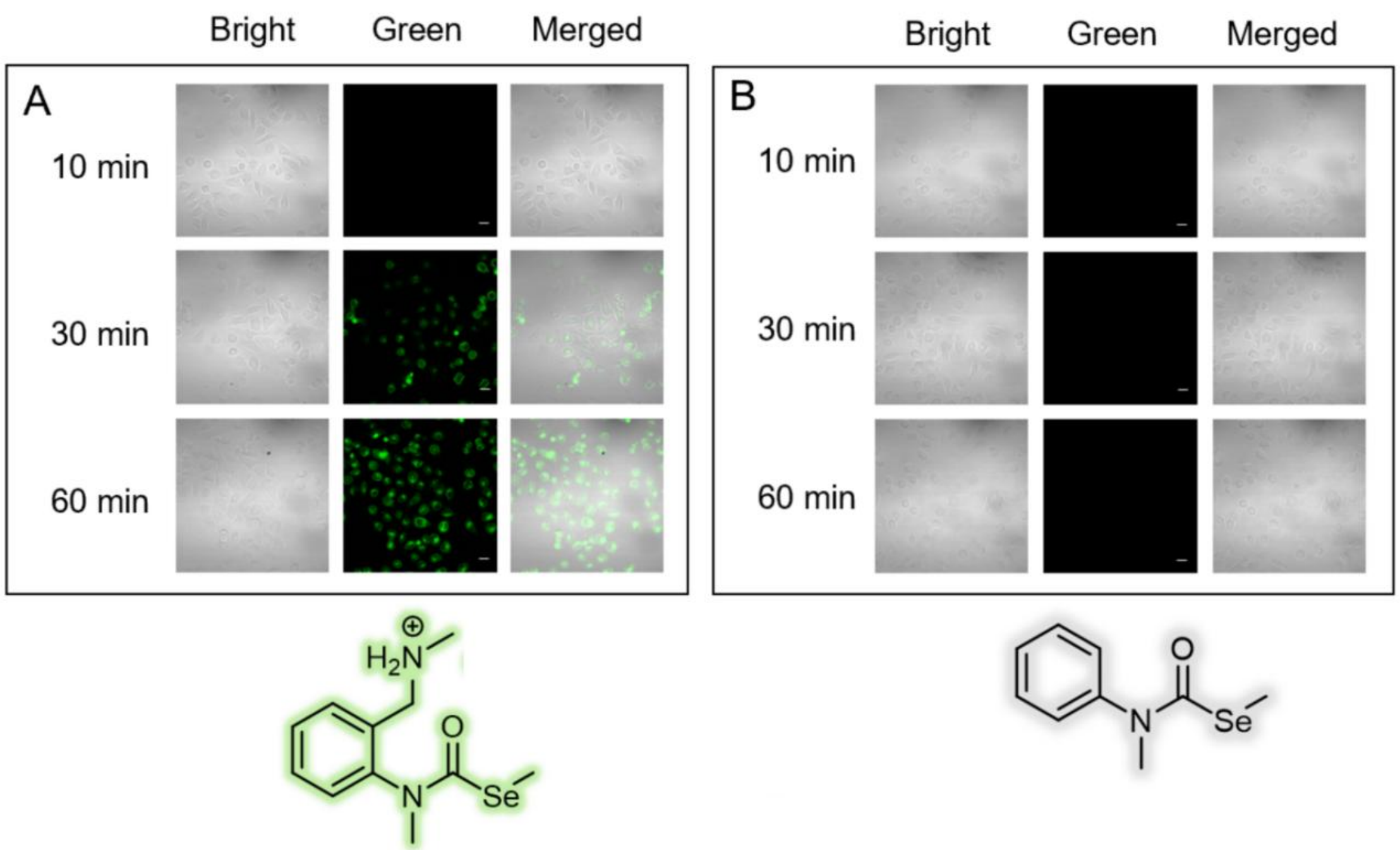

Figure S15. Confocal fluorescence images of HeLa cells stained with **Sel-green** (10 μM) for 20 min, and then incubated with 10 μM **TD-MSe1** (A) or 20 μM **3** (B) for various time periods (10–60 min). Cells were fixed at the indicated time points and then imaged. Scale bar: 20 μm.

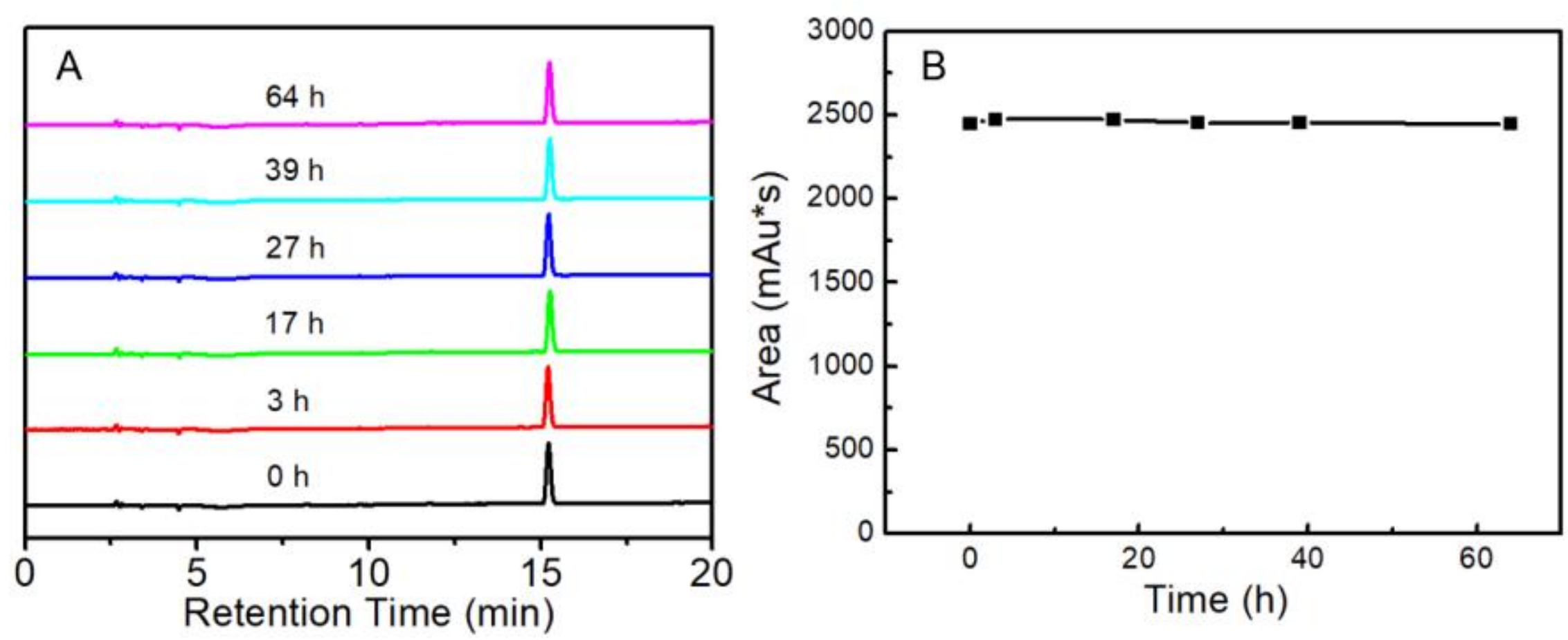

Figure S16. (A) Time-dependent HPLC traces of **TD-MSe2** (0.5 mM) in PBS buffer (pH 7.4, containing 30% CH_3CN). (B) Time-dependent peak areas of **TD-MSe2** from (A). Detection wavelength: 254 nm. The results imply that donor **TD-MSe2** is stable in PBS (pH 7.4) after 64 h of incubation.

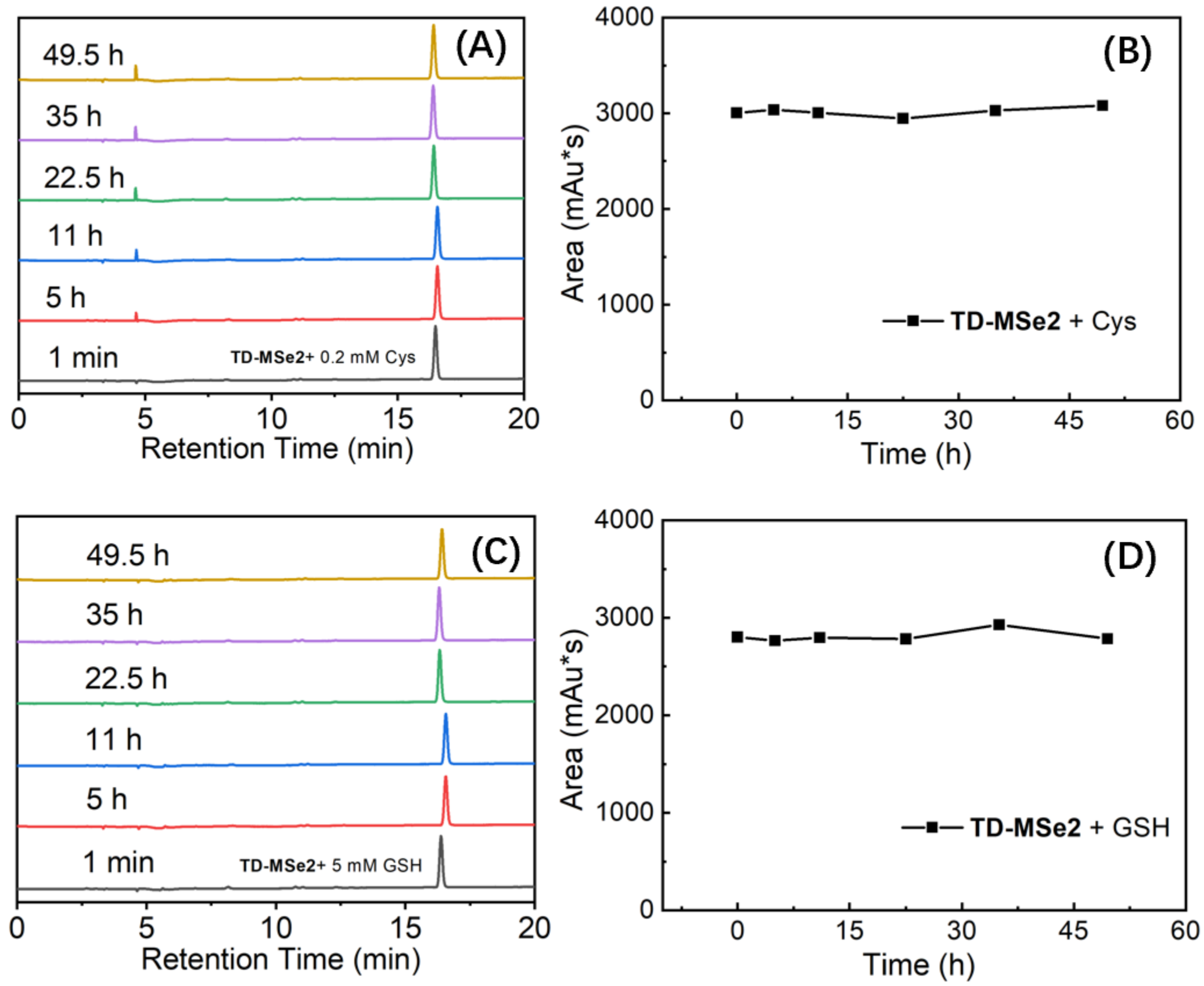

Figure S17. (A) Time-dependent HPLC traces of **TD-MSe2** (0.5 mM) in PBS buffer (pH 7.4, containing 30% CH_3CN) with 0.2 mM Cys at room temperature. (B) Time-dependent peak areas of **TD-MSe2** from (A). (C) Time-dependent HPLC traces of **TD-MSe2** (0.5 mM) in PBS buffer (pH 7.4, containing 30% CH_3CN) with 5 mM GSH at room temperature. (D) Time-dependent peak areas of **TD-MSe2** from (C). Detection wavelength: 254 nm. The results imply that donor **TD-MSe2** is stable in the presence of Cys and GSH after 2 days of incubation.

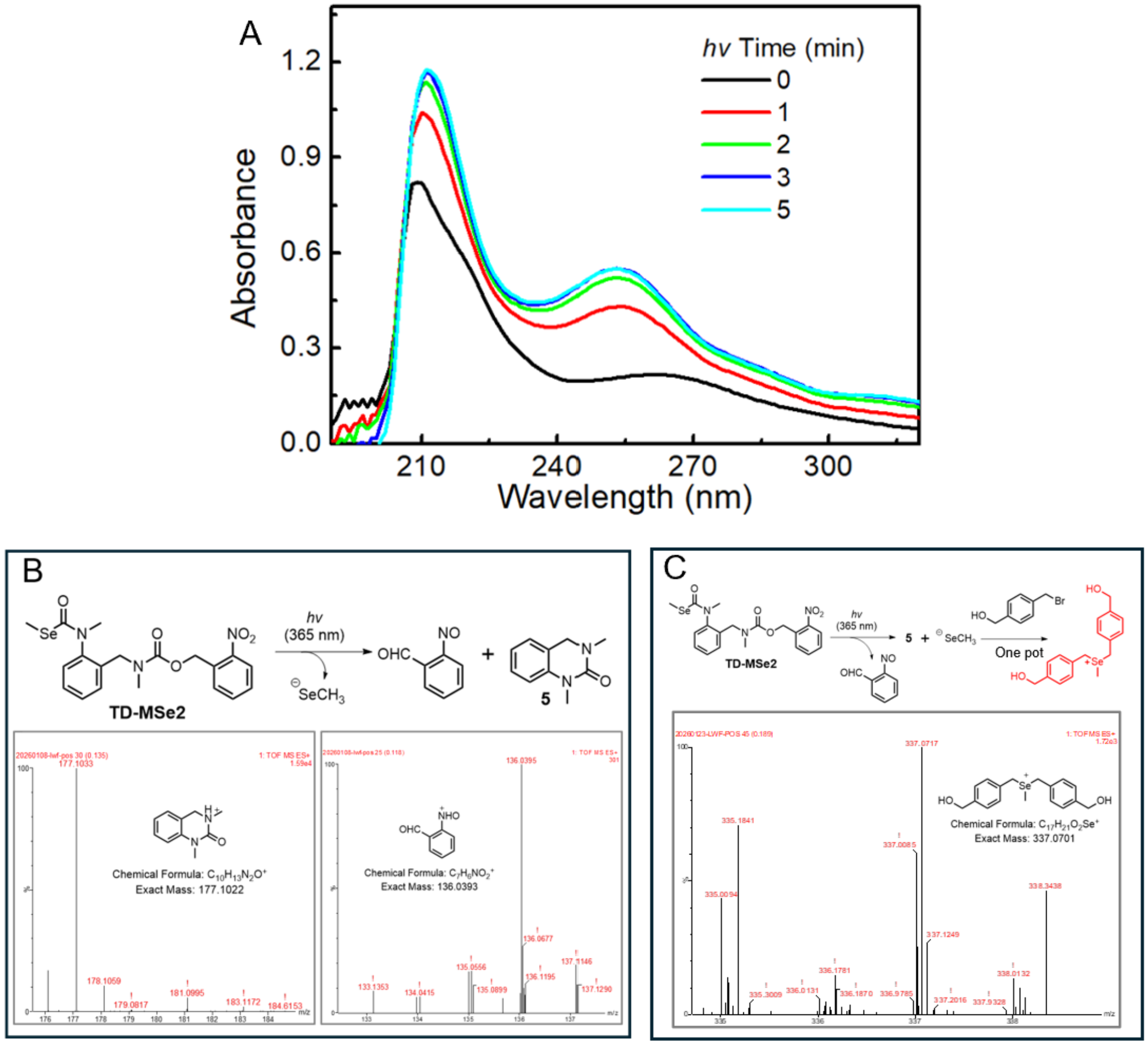

Figure S18. (A) Absorbance spectra of **TD-MSe2** (50 μM) in PBS buffer (pH 7.4, containing 10% CH_3CN) before and after different irradiation times under 365 nm light. The absorbance spectrum after 5 min irradiation is consistent with that of **5**. (B) HRMS analysis for **TD-MSe2** (0.5 mM) in PBS buffer (50 mM, pH 7.4, containing 50% CH_3CN) after 365 nm light activation for 30 min. (C) HRMS analysis for the one-pot reaction of **TD-MSe2** (1 mM) and (4-(bromomethyl)phenyl)methanol (10 mM) in PBS buffer (50 mM, pH 7.4, containing 50% CH_3CN) under 365 nm light irradiation for 30 min. The possible reaction pathways in (B, C) are given.

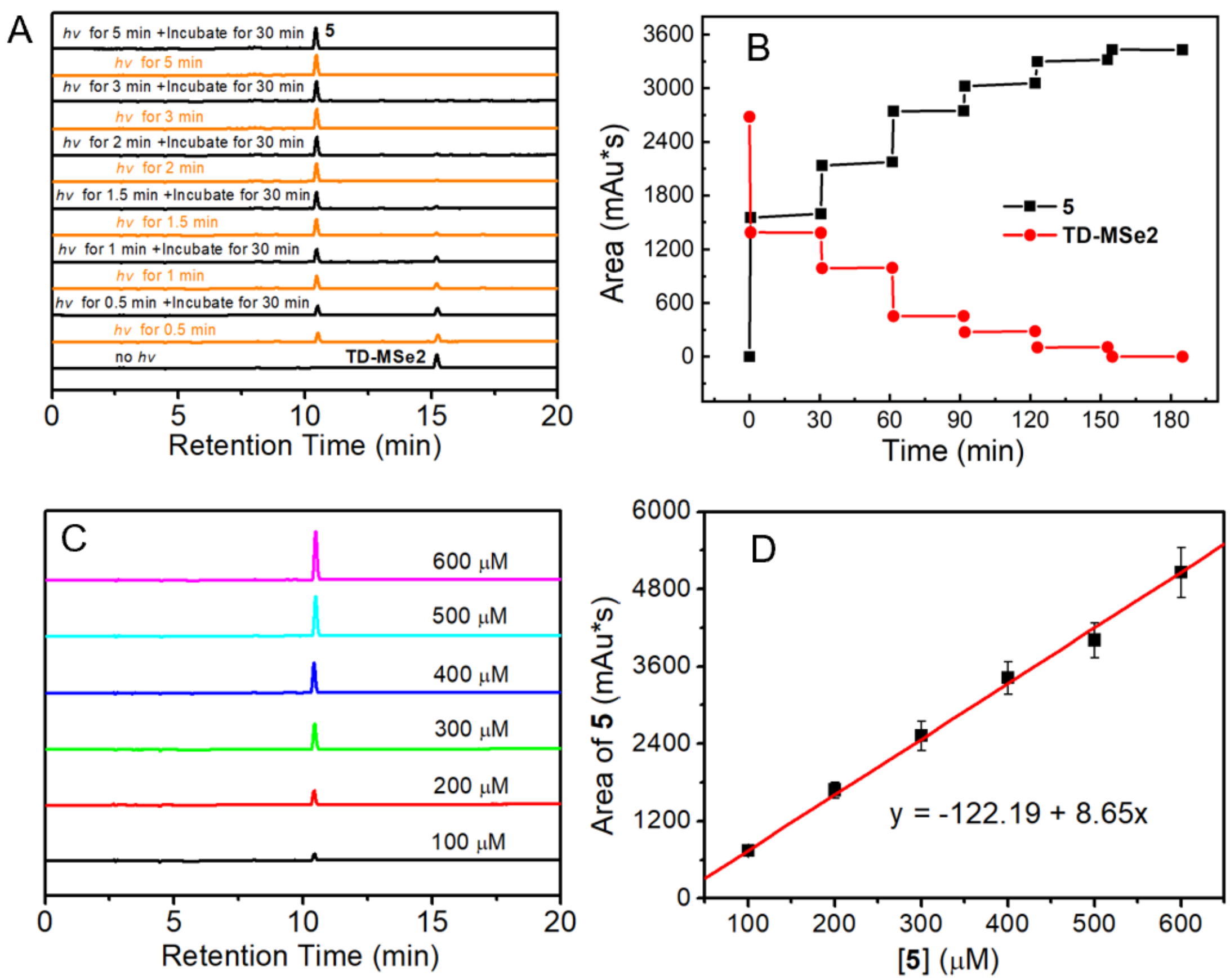

Figure S19. (A) HPLC traces of **TD-MSe2** (0.5 mM) under "light" and "dark" conditions in PBS buffer (pH 7.4, containing 30% CH_3CN). Detection wavelength: 254 nm. (B) Time-dependent peak areas of **TD-MSe2** and **5** from the HPLC traces in (A). (C) Representative HPLC traces of different concentrations of **5** (100−600 μM) in PBS buffer (pH 7.4, containing 30% CH_3CN). Detection wavelength: 254 nm. (D) HPLC calibration curve of the peak areas of **5** versus the concentrations. The results are expressed as mean ± SD (n = 3).

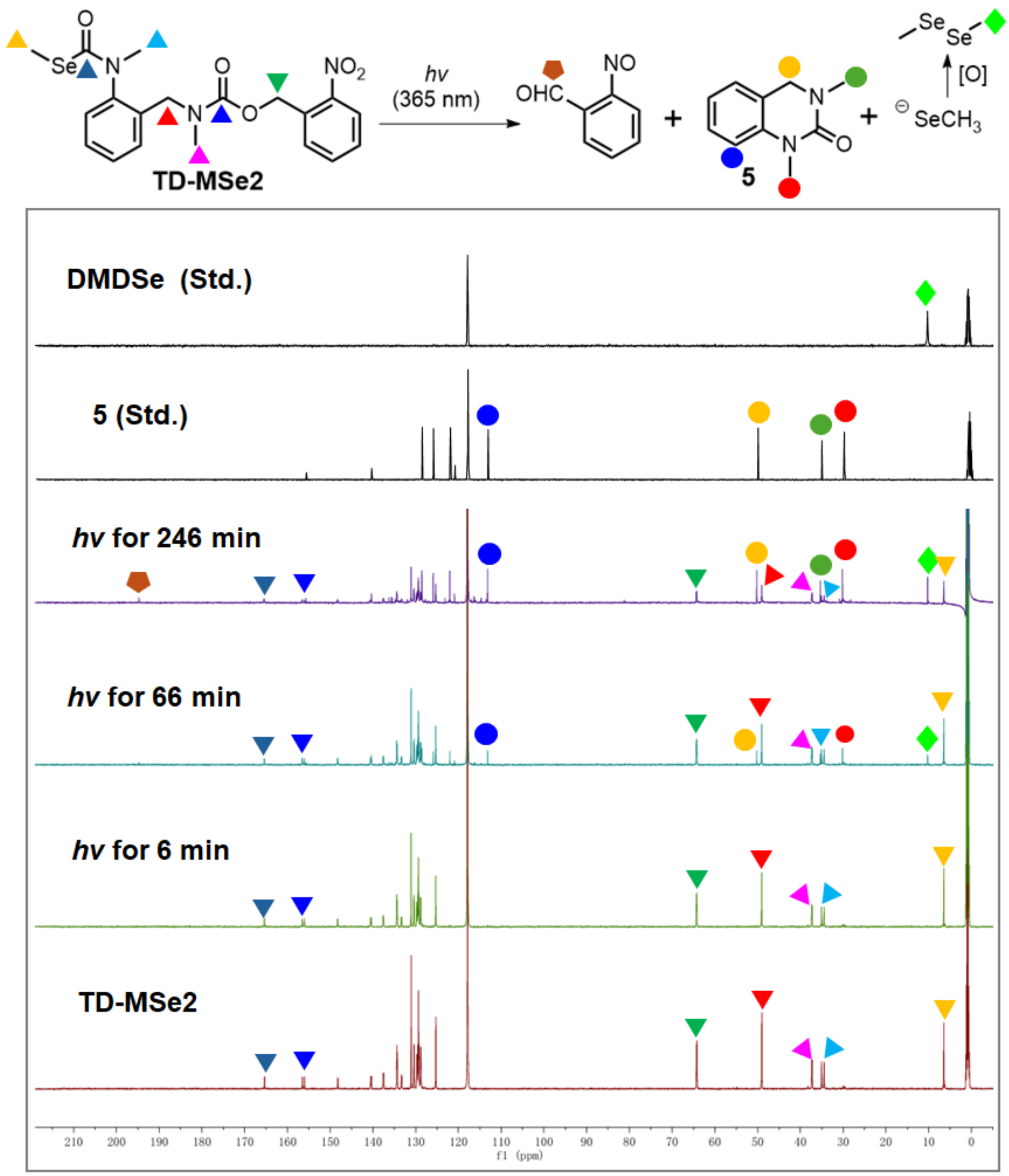

Figure S20. Time-dependent $^{13}C\{^1H\}$ NMR for the light-activated reaction of **TD-MSe2** (160 mM) in CD_3CN. Control NMR spectra of pure **5** and **DMDSe** are also shown. Several key peaks are marked by colored symbols for the expected groups.

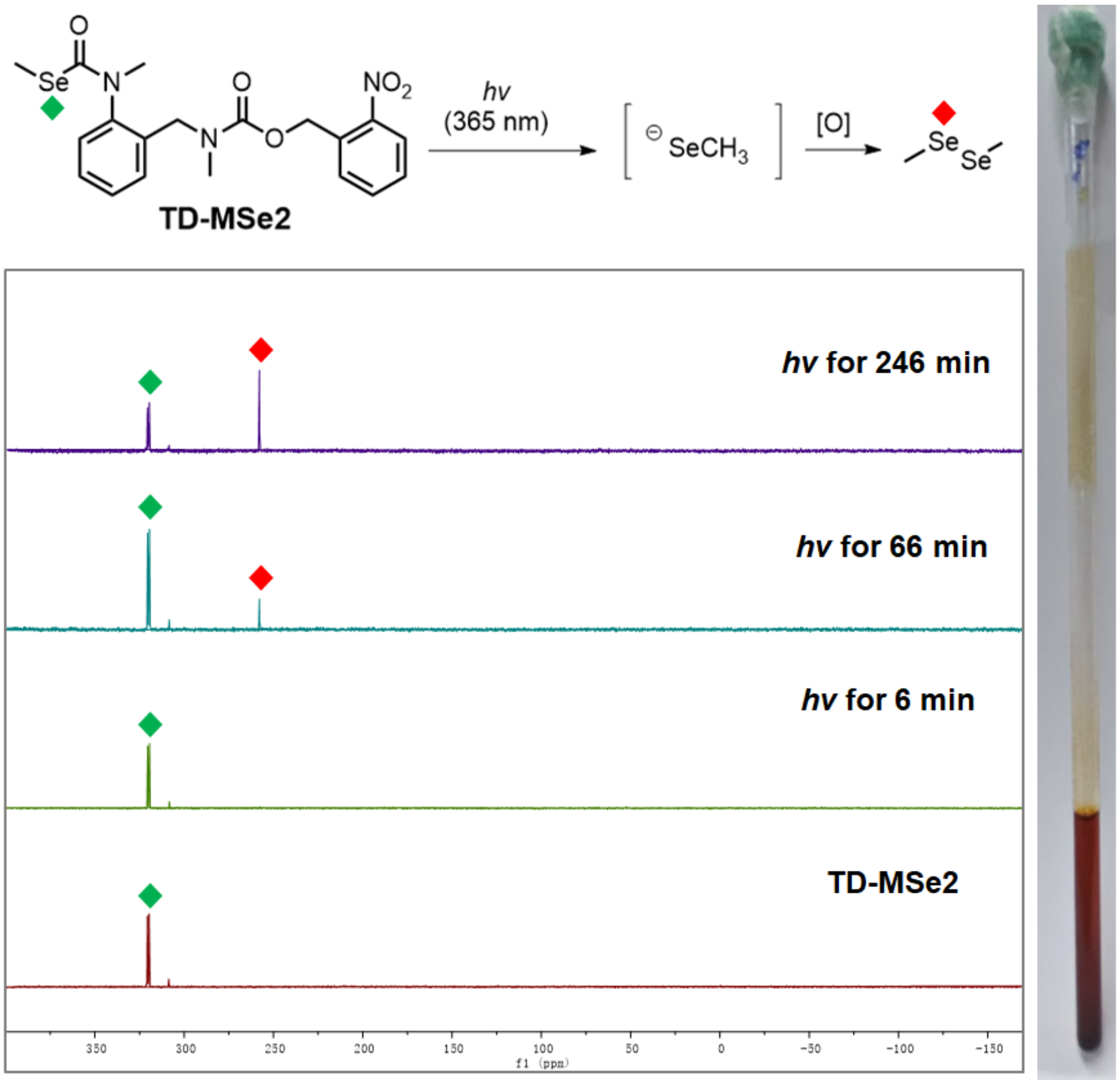

Figure S21. Time-dependent $^{77}Se\{^{1}H\}$ NMR for the light-activated reaction of **TD-MSe2** (160 mM) in CD_3CN. The chemical shift of **DMDSe** was assigned according to a literature.[1] Several key peaks are marked by colored symbols for the expected groups. Photo of the tested NMR tube after irradiation is shown in right part, and the solution color is consistent with that of **DMDSe**.

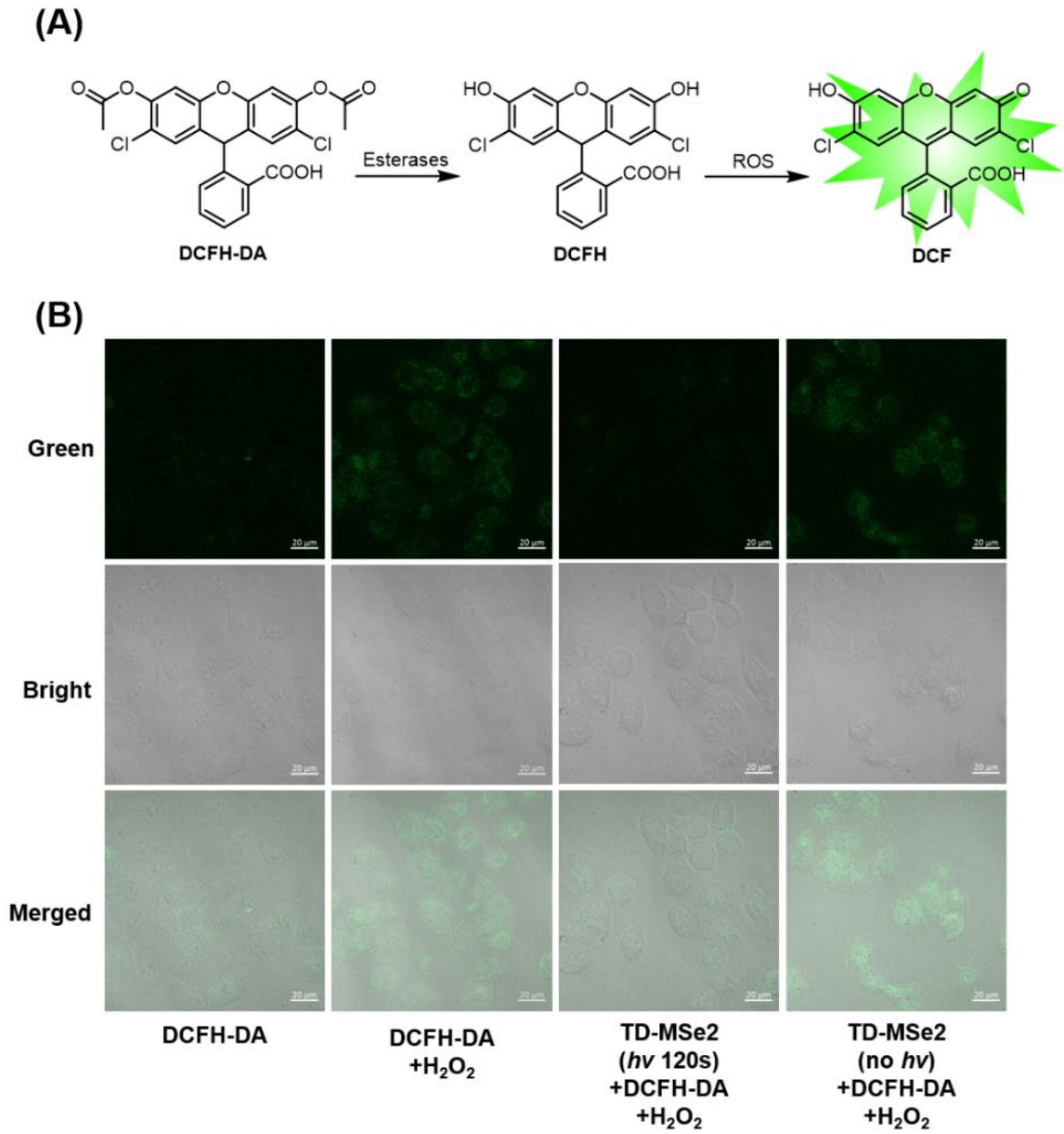

Figure S22. (A) Detection mechanism of a commercially available probe **DCFH-DA** for sensing ROS in live cells. (B) Fluorescence images of HeLa cells under the listed treatment conditions on bright and green channels. Scale bar: 20 μm.

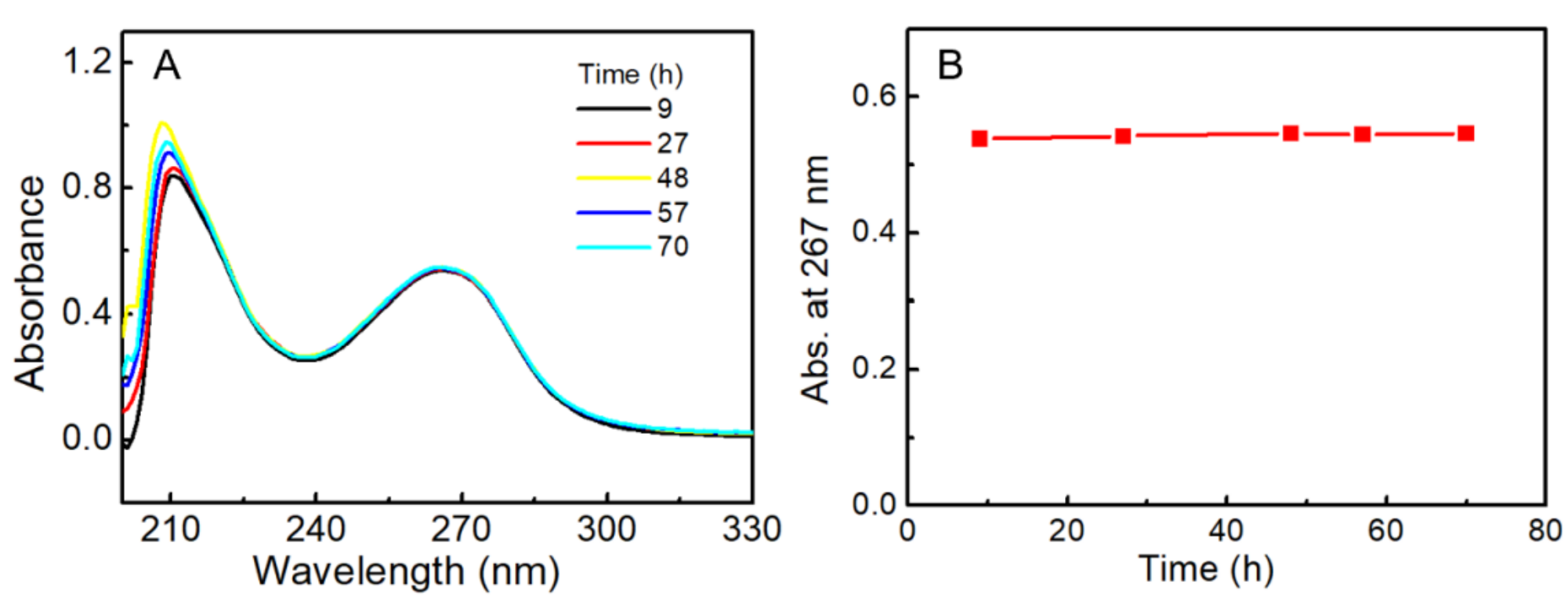

Figure S23. (A) Time-dependent UV–vis spectra of **TD-MSe3** (50 μM) in PBS buffer (pH 7.4, containing 10% CH_3CN). (B) The time-dependent intensities at 267 nm from (A). The results imply that the donor is stable in PBS over 70 h of incubation.

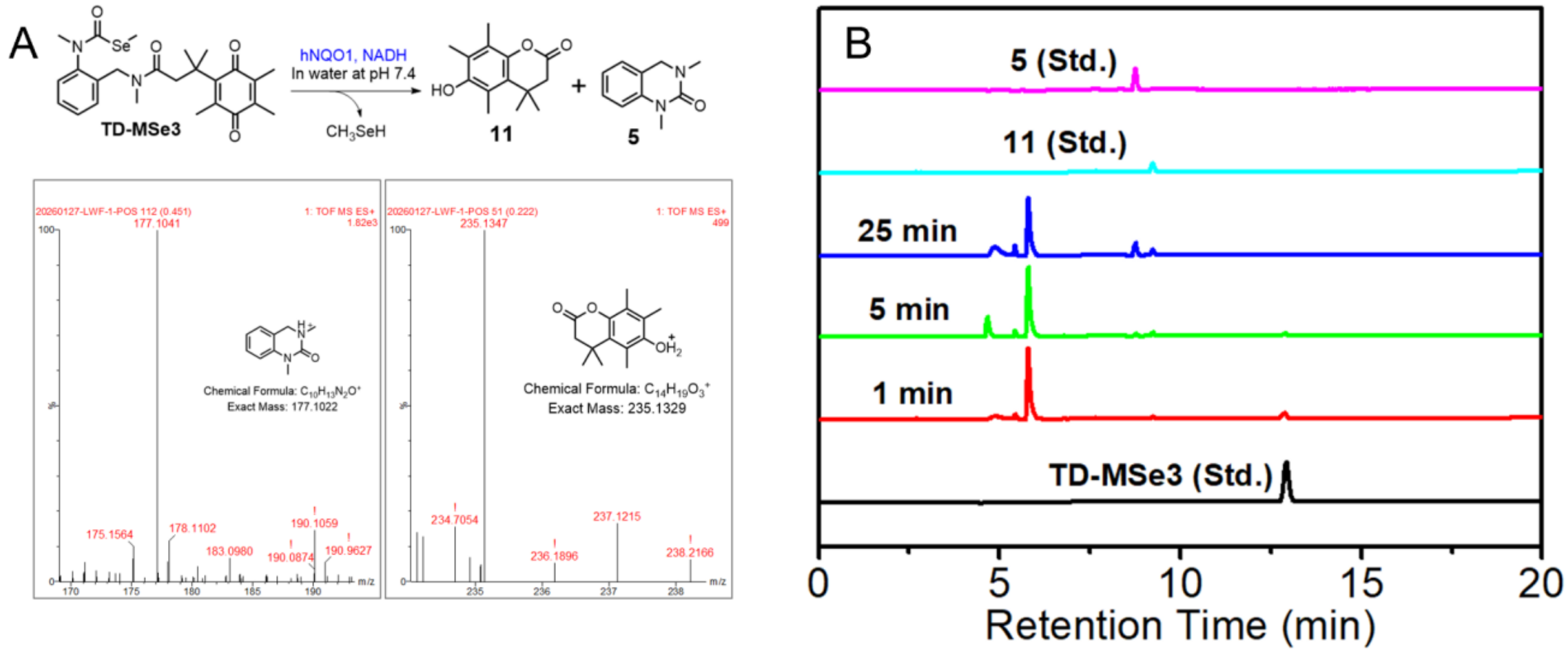

Figure S24. (A) HRMS analysis for the reaction of **TD-MSe3** (0.25 mM) in PBS buffer (50 mM, pH 7.4, containing 1 mM NADH, 1 mg/mL BSA, 10 µg/mL hNQO1 and 10% CH_3CN) for 1 h. (B) Time-dependent full HPLC traces of the enzymatic reaction of **TD-MSe3** (150 µM) in PBS buffer (pH 7.4, containing 0.5 mM NADH, 30 µg/mL BSA, 10 µg/mL hNQO1 and 10% CH_3CN) at 25 °C. The control traces of pure **TD-MSe3** in PBS buffer (pH 7.4, containing 30% CH_3CN), **5** or **11** in PBS buffer (pH 7.4, containing 10% CH_3CN) are also shown. Detection wavelength: 254 nm.

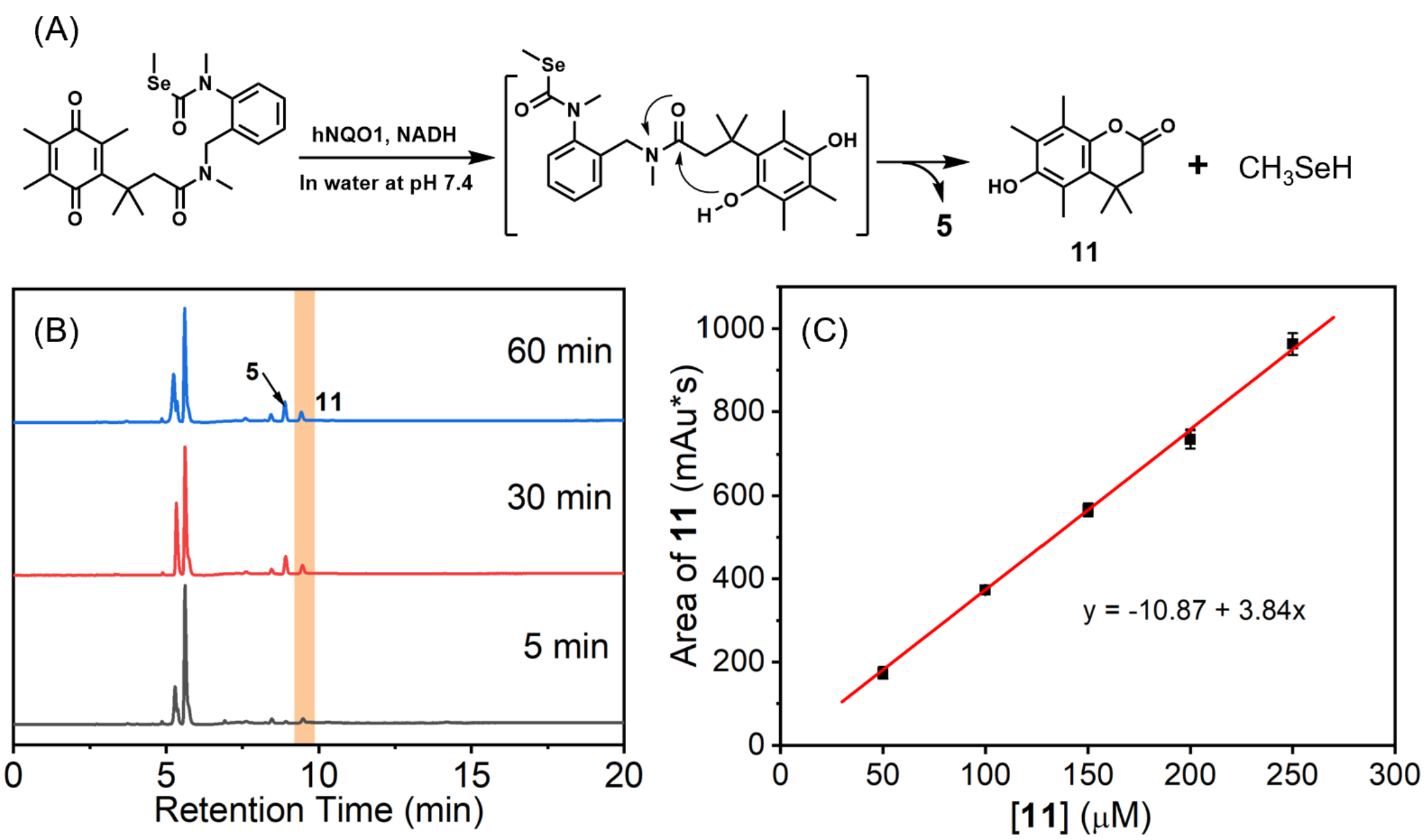

Figure S25. (A) Reaction pathways of **TD-MSe3** with hNQO1. (B) Time-dependent full HPLC traces of the enzymatic reaction of **TD-MSe3** (110 µM) in PBS buffer (pH 7.4, containing 1 mM NADH, 30 µg/mL BSA, 10 µg/mL hNQO1 and 10% CH_3CN) at 25 °C. (C) HPLC calibration curve of the peak areas of **11** versus the concentrations. The results are expressed as mean ± SD (n = 3). Detection wavelength: 254 nm.

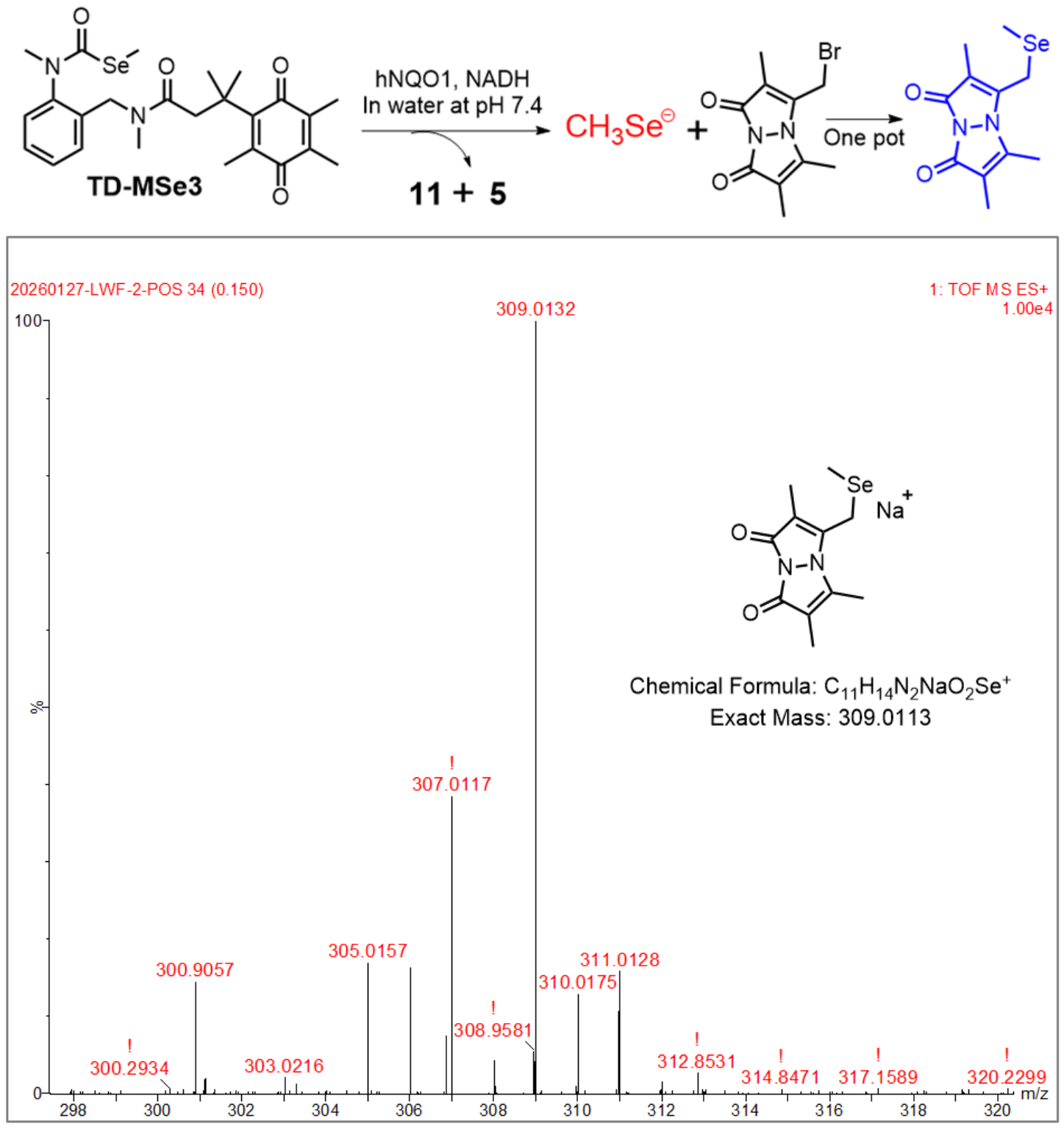

Figure S26. HRMS analysis for one-pot reaction of **TD-MSe3** (0.25 mM) and mBB (0.5 mM) in PBS (pH 7.4, containing 1 mM NADH, 1 mg/mL BSA, 10 µg/mL hNQO1 and 10% CH_3CN). The possible reaction pathway for the trapped CH_3SeH product is given.

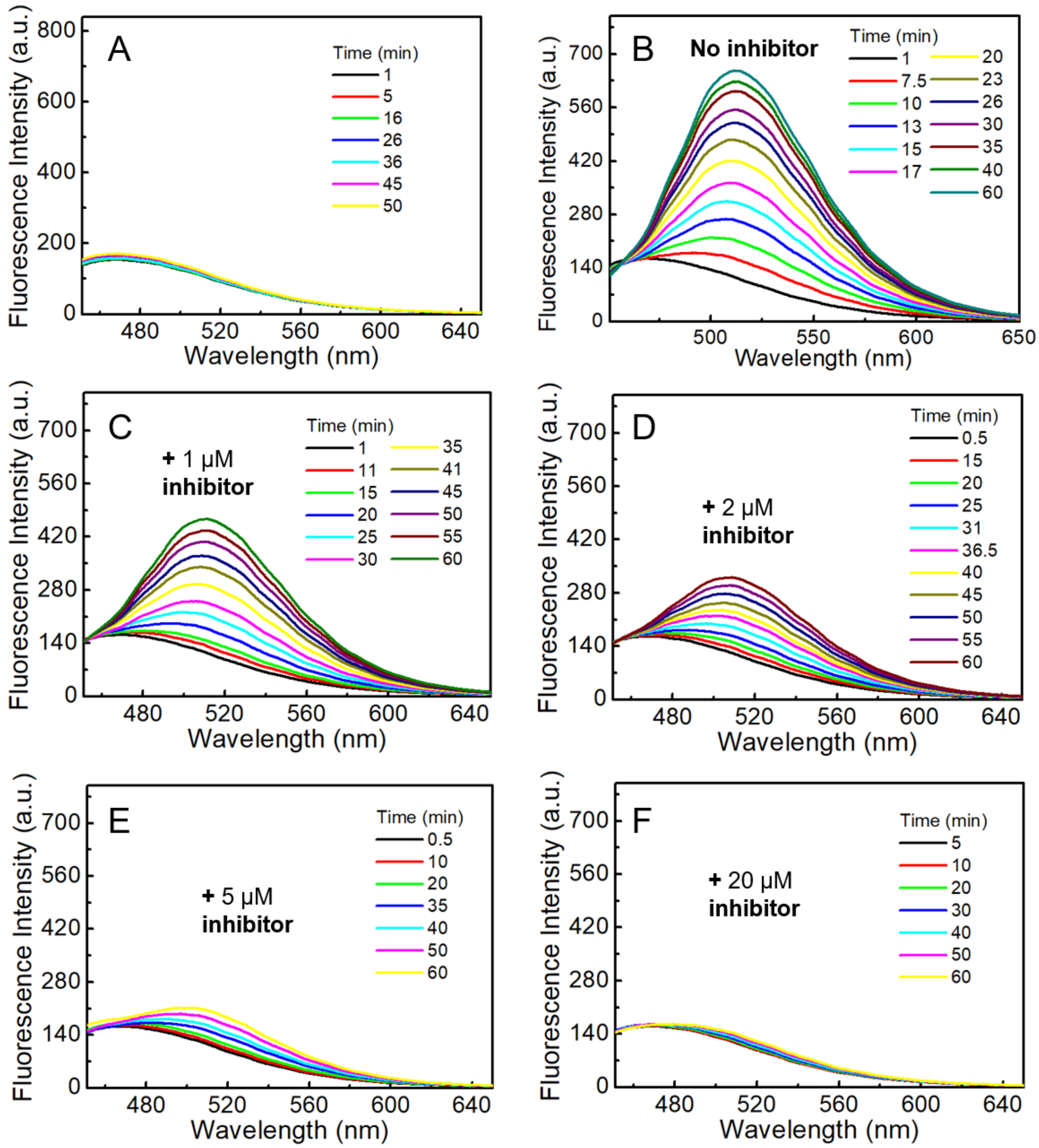

Figure S27. (A) Time-dependent fluorescence spectra of **Sel-green** (10 μM) in PBS (pH 7.4, containing containing 0.1 mM NADH, 15 μg/mL BSA, 5 μg/mL hNQO1 and 10% CH_3CN) at 25 °C. (B–F) Time-dependent fluorescence spectra of **Sel-green** (10 μM) without (B) or with different concentrations of the inhibitor dicoumarol (1 μM (C), 2 μM (D), 5 μM (E) and 20 μM (F)) in PBS (pH 7.4, containing 50 μM **TD-MSe3**, 0.1 mM NADH, 15 μg/mL BSA, 5 μg/mL hNQO1 and 10% CH_3CN) at 25 °C. Excitation, 370 nm.

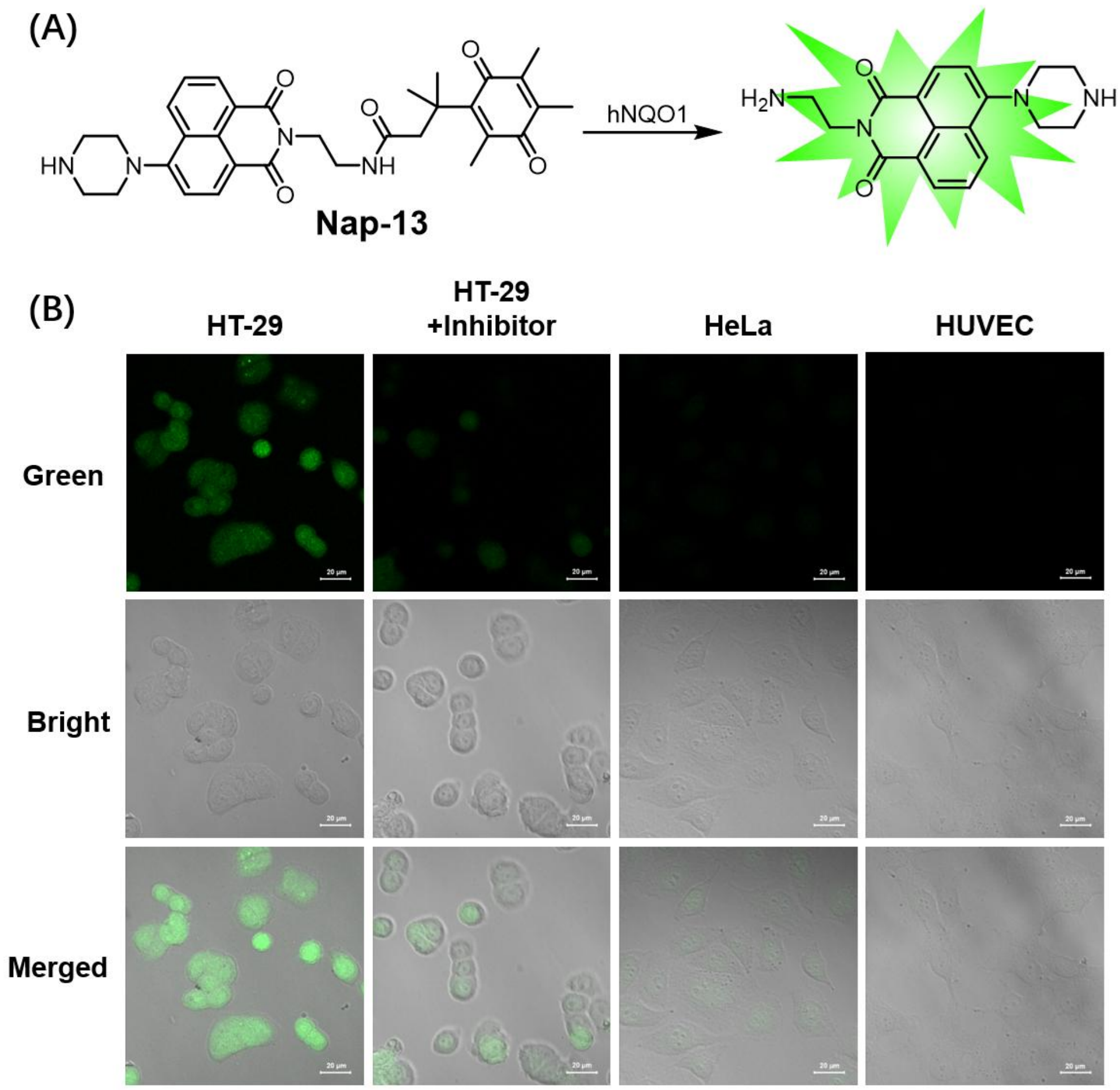

Figure S28. (A) Detection mechanism of the probe **Nap-13** for sensing hNQO1.[3] (B) Confocal fluorescence images of HT-29 cells, HeLa, and HUVEC cells incubated with **Nap-13** (10 μM) alone for 1 h. For inhibition control, HT-29 cells were pre-incubated with dicoumarol (100 μM) for 0.5 h prior to **Nap-13** (10 μM) incubation for 1 h. Scale bar: 20 μm.

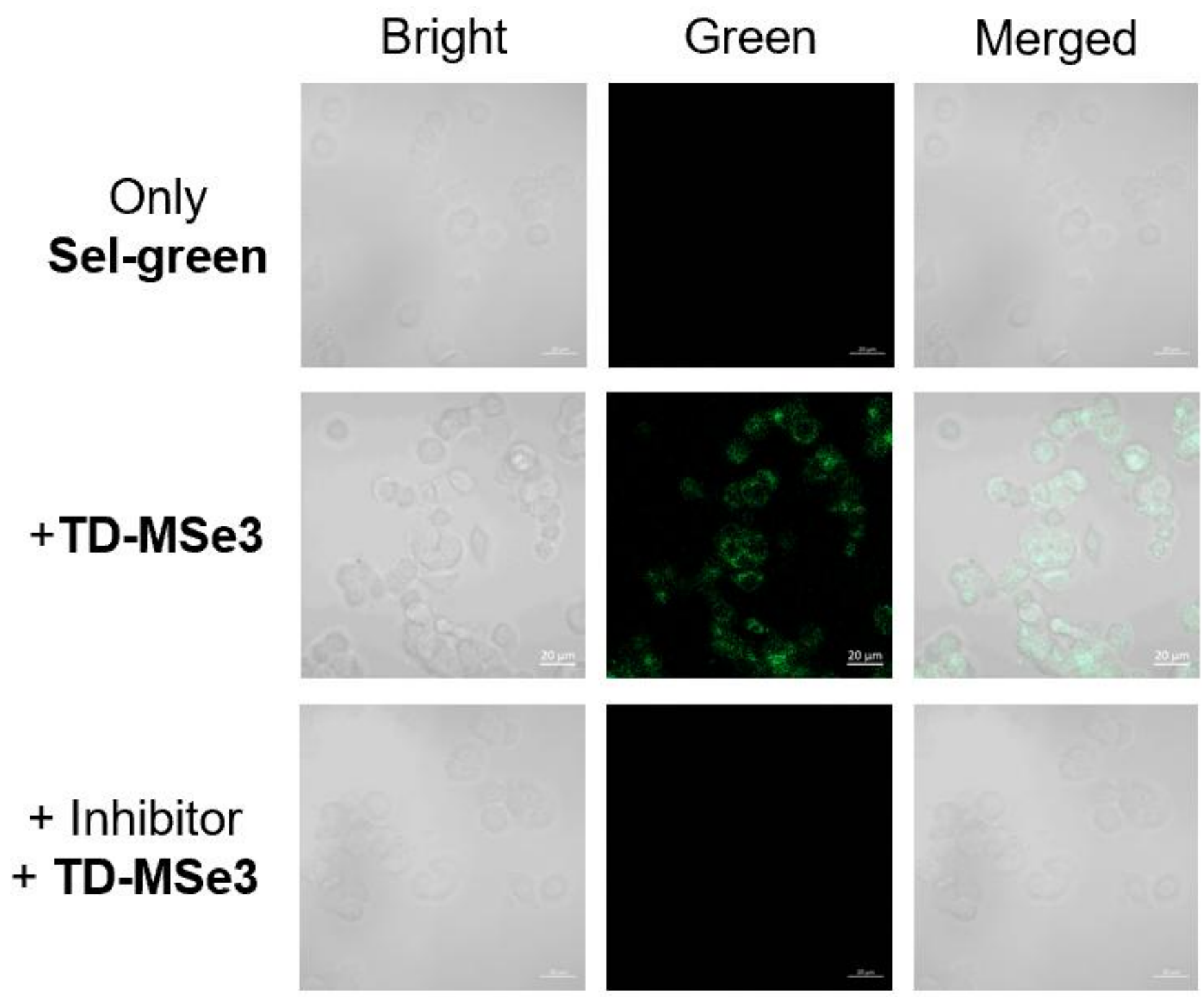

Figure S29. Confocal fluorescence images of HT-29 cells stained with **Sel-green** and treated with **TD-MSe3**. For inhibition control, cells were pre-incubated with dicoumarol and then treated with **Sel-green** and **TD-MSe3**. Scale bar: 20 μm.

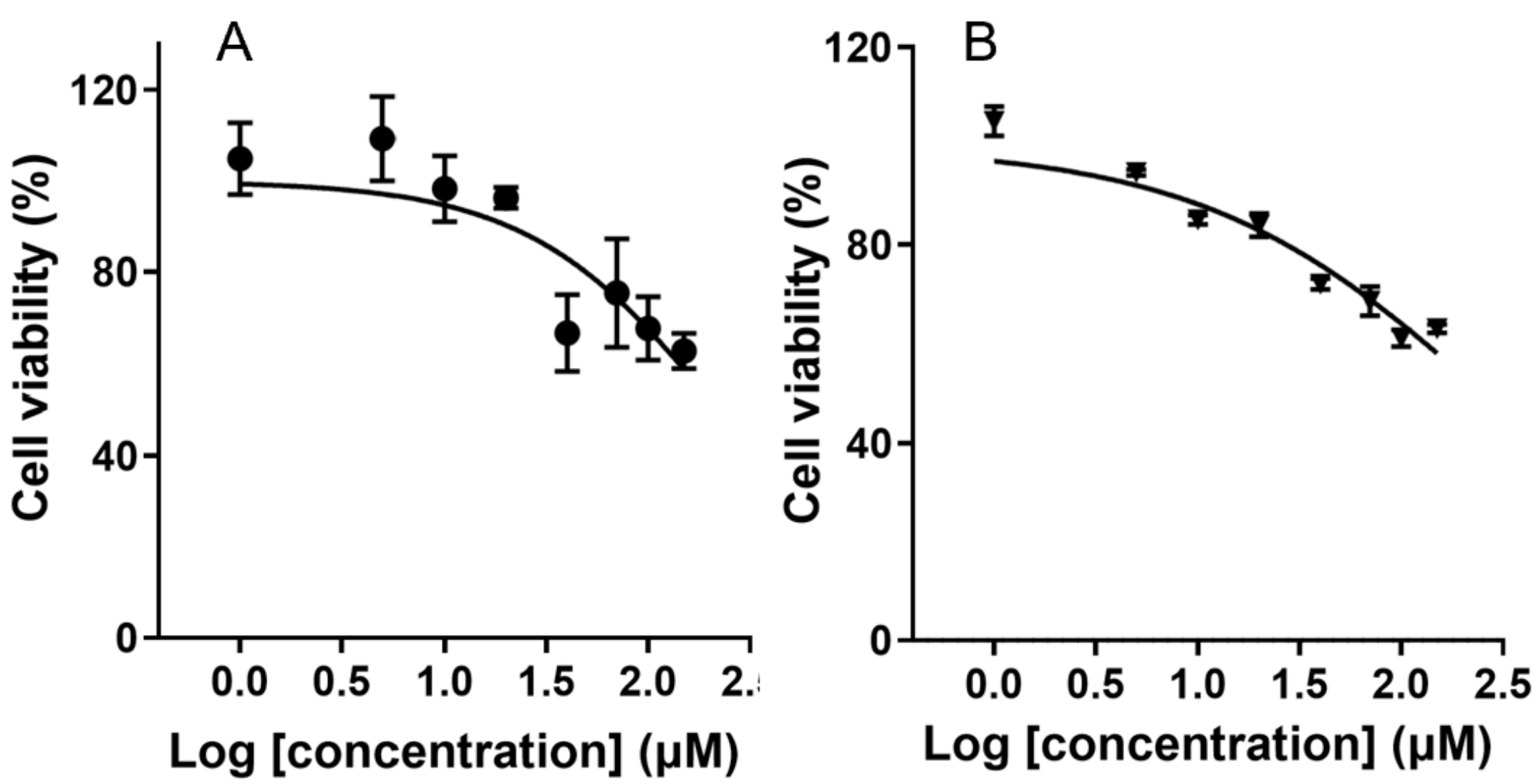

Figure S30. Cell viability of HeLa cells (A) or HUVEC cells (B) after co-incubation with different concentrations of **TD-MSe3** (0, 1, 5, 10, 20, 40, 70, 100, 150 μM) for 72 h. Data were expressed as mean ± SD (n = 3). Each solid line represents nonlinear regression analysis for IC_{50} determination.

Table S1. Comparison of properties for donors **TD-MSe** and **FITA-E1**.

Donor	Release species	Trigger type (Triggering conditions)	Stability	Release kinetics	Application	Ref.
**TD-MSe1**	CH_3SeH	Non-triggered (Hydrolysis in aqueous solution)	Stable in solid state	$t_{1/2}$ = 5 s	Intracellular CH_3SeH delivery	
**TD-MSe2**	CH_3SeH	Trigger-type (light)	Stable in PBS buffer (pH 7.4)	light activation is the rate-determining step	ROS scavenging / cytoprotection	This work
**TD-MSe3**	CH_3SeH	Trigger-type (enzyme)	Stable in PBS buffer (pH = 7.4)	hNQO1 reduction is the rate-determining step	hNQO1-dependent selective toxicity	
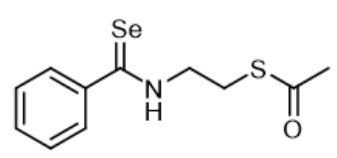 FITA-E1	H_2Se	Trigger-type (enzyme)	Stable in PBS buffer (pH 7.4)	**FITA** process: $t_{1/2}$ = 50 s esterase activation is the rate-limited step.	H_2Se delivery	[2]

2. General Chemicals and Instruments

All chemicals and solvents used for syntheses or tests in this research were purchased from commercial suppliers and applied directly in experiments without further purification. The progress of reactions was monitored by TLC on pre-coated silica plates (Merck 60F–254, 250 µm in thickness), and spots were visualized by basic $KMnO_4$, UV light and/or iodine. Merck silica gel 60 (70–200 mesh) was used for general column chromatography purification. ^{1}H, $^{13}C\{^{1}H\}$ and $^{19}F\{^{1}H\}$ NMR spectra were recorded on a Bruker 400 (400 MHz for ^{1}H NMR, 101 MHz for ^{13}C NMR and 376 MHz for ^{19}F NMR) at room temperature. $^{77}Se\{^{1}H\}$ NMR spectra were recorded on a Bruker 600 (114 MHz for ^{77}Se NMR) spectrometer at room temperature. Chemical shifts are reported in parts per million relative to internal standard tetramethylsilane ($Si(CH_3)_4$ = 0.00 ppm) or residual solvent peaks (chloroform-*d* = 7.26 ppm; water-d_2 = 4.79 ppm; methanol-d_4 = 3.31 ppm; acetonitrile-d_3 = 1.94 ppm). High-resolution mass spectra (HRMS) were recorded on a XEVO-G2QTOF (ESI) (Waters, USA). Fourier–transform infrared (FT–IR) spectra were recorded on a Bruker VERTEX 70 spectrometer. Activation of the light-triggered CH_3SeH donor was performed using a UV-lamp (Zhongshan Yanxizao Electronic Factory, AC90-240V, 18 W-365 nm). The UV-visible spectra were recorded on a UV-6000 UV-VIS-NIR spectrophotometer (METASH, China). Fluorescence studies were performed using a F-280 spectrophotometer (Tianjin Gangdong Sci & Tech., Development. Co., Ltd). ANGELA TECHNOLOGIES HPLC LC-10F with C18 column (4.6 mm x 250 mm) was employed for HPLC analysis with a detection wavelength at 254 nm. The cellular bioimaging was carried out on a confocal microscope (Olympus FV1000 or Axio-Imager_LSM-800).

3. Experimental Methods

UV–vis analyses and reaction kinetics.

Reaction kinetics of **1**. All spectroscopic experiments were performed in sealed, degassed PBS solutions (200 mM, pH 7.4, containing 30% CH_3CN) in a 3 mL sealed cuvette with a 2 mL degassed solution. **1** was freshly dissolved in CH_3CN to prepare a stock solution (10 mM). Tris-(2-carboxyethyl)-phosphine (TCEP) was freshly dissolved in sealed, degassed PBS (200 mM, pH 7.4) to prepare a stock solution (100 mM). For the UV–vis studies, **1** (50 μM) was co-incubated with TCEP (1, 4, 5, 6, 7 or 8 mM) in PBS at 25 °C, and time-dependent absorbance spectra were recorded. The absorbance profiles were recorded at different time points for kinetic analysis. The apparent rate, k_{obs} was determined by fitting the time-dependent intensities data (at 257 nm) with a single exponential function.

UV–vis analysis of **2** or **3**. **2** or **3** was dissolved in CH_3CN to prepare a stock solution (10 mM). The stability of **2** or **3** (50 μM) was assessed in PBS (pH 7.4, containing 10% CH_3CN) at 25 °C, and the time-dependent absorbance spectra were recorded. The hydrolysis rate k_{obs} was determined by fitting the time-dependent intensities data (at 255 nm) with a single exponential function.

Reaction kinetics of **TD-MSe1** at different pH values. All spectroscopic experiments were performed in sealed, degassed PBS (200 mM, containing 10% CH_3CN) in a 3 mL sealed cuvette with a 2 mL degassed solution. **TD-MSe1** was freshly dissolved in CH_3CN to prepare a stock solution (10 mM). In addition, PBS buffers were prepared with final pH values of 4.5, 5.0, 5.5, 6.0, 6.5, 7.0, 7.4, and 8.5 using HCl or NaOH solutions. For the UV−vis experiments, **TD-MSe1** (30 μM) was incubated in PBS (pH 4.5, 5.0, 5.5, 6.0, 6.5, 7.0 or 7.4) at 25 °C, and the time-dependent absorbance spectra or intensities at 255 nm were recorded for kinetic analysis. The k_{obs} was determined by fitting the time-dependent intensities data (at 255 nm) with a single exponential function.

UV–vis spectra of **5** at different pH. Compound **5** was dissolved in CH_3CN to prepare a stock solution (50 mM). The absorption spectra of **5** (50 μM) in degassed PBS (200 mM, pH 4.5, 5.5, 6.5, 7.4, or 8.5, containing 10% CH_3CN) were recorded.

UV–vis analysis of the light-triggered donor. **TD-MSe2** was dissolved in CH_3CN to prepare a stock solution (10 mM). **TD-MSe2** (50 μM) in degassed and sealed PBS (200 mM, pH 7.4, containing 10% CH_3CN) was activated by 365 nm UV-irradiation for a certain time, and then the absorption spectra were recorded.

UV–vis analysis of **TD-MSe3**. The stability was assessed by incubation of **TD-MSe3** (50 μM) in PBS (200 mM, pH 7.4, containing 10% CH_3CN) at 25 °C, and the time-dependent absorbance spectra were recorded.

Fluorescence spectroscopy analyses.

Fluorescence spectroscopy analysis of the CH_3SeH release from **2** or **3**. Probe **Sel-green** was dissolved in DMSO to make a 4 mM stock solution. **2** or **3** (20 μM) was incubated with **Sel-green** (10 μM) in degassed, sealed PBS (200 mM, pH 7.4, containing 10% CH_3CN) at 25 °C, and the time-dependent fluorescence spectra were recorded with excitation at 370 nm.

Fluorescent detection for the CH_3SeH release from **TD-MSe1**. Probe **Sel-green** (10 μM) was co-incubated without or with donor **TD-MSe1** (5, 10, 20, or 50 μM) in sealed, degassed PBS (200 mM, pH 7.4, containing 10% CH_3CN) at 25 °C, and immediately, the time-dependent fluorescent spectra were recorded with excitation at 370 nm. The donor solutions before and after reaction with **Sel-green** could be visualized under 365 nm UV lamp.

Fluorescent detection for the CH_3SeH release from **TD-MSe3**. Nicotinamide adenine dinucleotide (NADH) was dissolved in PBS (50 mM, pH 7.4) to prepare a stock solution (50 mM). Human NAD(P)H:quinine oxidoreductase 1 (hNQO1, EC1.6.99.2) was dissolved in 1 mL PBS (50 mM, pH 7.4, containing 3 mg bovine serum albumin (BSA)) to prepare a stock solution (1 mg/mL). Probe **Sel-green** (10 μM) was co-incubated with hNQO1 (5 μg/mL) and NADH (0.1 mM) at 25 °C in sealed, degassed PBS (50 mM, pH 7.4, containing 15 μg/mL BSA and 10% CH_3CN) in the presence or absence of **TD-MSe3** (50 μM), and immediately, the time-dependent fluorescent spectra were recorded with excitation at 370 nm.

A fluorescent hNQO1 inhibition assay. Different concentrations of the inhibitor dicoumarol[3] (1–20 μM) and hNQO1 (5 μg/mL) were pre-incubated for 5 min in PBS (50 mM, pH 7.4, containing 10% CH_3CN), and then **Sel-green** (10 μM), NADH (0.1 mM), BSA (15 μg/mL) and **TD-MSe3** (50 μM) were added to start the reactions. Immediately, time-dependent fluorescent spectra were monitored, and intensities at 515 nm were used to compare the inhibition effects.

HPLC tests.

HPLC analysis of the stability of **2** or **3**. The water-stability of **2** or **3** (0.5 mM) in degassed, sealed PBS buffer (200 mM, pH 7.4, containing 30% CH_3CN) was assessed by time-dependent HPLC tests. Conditions: detection wavelength: 254 nm; flow 1.0 mL/min; buffer A: 0.1% (v/v) trifluoroacetic acid (TFA) in water; buffer B: methanol; elution condition: 0–3 min, buffer B: 5–80%; 3–18 min, buffer B: 80–95%; 18–20 min, buffer B: 95–5%.

HPLC analysis of the stability of **3** in the presence of biological nucleophiles. The stability of **3** (0.5 mM) in PBS buffer (200 mM, pH 7.4, containing 30% CH_3CN) in the presence of physiologically relevant concentrations of cysteine (Cys, 0.2 mM), reduced

glutathione (GSH, 5 mM), glycine (Gly, 1 mM), or lysine (Lys, 1 mM) was monitored by time-dependent HPLC analysis. HPLC conditions were the same as the description above.

HPLC calibration curve of **3**. Different concentrations of **3** (300–700 μM) were dissolved in degassed PBS buffer (pH 8.5, containing 50% CH_3CN), and aliquots of the solutions were directly analyzed by HPLC to obtain the peak areas of **3**. HPLC conditions are the same as the description for the stability test of **3**. All HPLC measurements were repeated three times. These mean values of the peak areas were plotted against the used amounts of **3**, and the calibration curve was obtained as $y = 36.77 + 2.51*x$.

Quantitative determination of CH_3SeH release from **TD-MSe1**. Methyl(phenyl)carbamoyl chloride was dissolved in CH_3CN to prepare a stock solution (100 mM). **TD-MSe1** (0.33 mM) was co-incubated with methyl(phenyl)carbamoyl chloride (2 mM) in PBS (pH 8.5, containing 50% CH_3CN and 1 mM dithiothreitol) for 5 h. Then, aliquots of the reaction solutions were subjected to HPLC tests to determine the peak area of **3**. HPLC conditions are the same as the description for the stability test of **3**. All measurements were repeated for three times to obtain the mean peak area of **3**. The corresponding **3** concentrations (equivalent to the released CH_3SeH) were calculated from the above calibration curve.

HPLC analysis of the decomposition of **TD-MSe1** at different pH. **TD-MSe1** (0.5 mM) was incubated in different PBS buffers (200 mM, pH 4.5, 5.5, 6.5 or pH 7.4, containing 30% CH_3CN) for a certain time, and then aliquots of the reaction solutions were subjected to HPLC tests. Control compound **5** was also checked by HPLC for comparison. Conditions: detection wavelength: 254 nm; flow 1.0 mL/min; buffer A: 0.1% (v/v) TFA in water; buffer B: methanol; elution condition: 0–2 min, buffer B: 5–60%; 2–18 min, buffer B: 60–95%; 18–20 min, buffer B: 95–5%.

HPLC analysis of the stability of **TD-MSe2**. The water stability of **TD-MSe2** (0.5 mM) in degassed, sealed PBS buffer (200 mM, pH 7.4, containing 30% CH_3CN) was checked by time-dependent HPLC tests. The stability of **TD-MSe2** (0.5 mM) was further monitored by time-dependent HPLC analysis in the presence of physiologically relevant concentrations of Cys (0.2 mM) or GSH (5 mM). Conditions: detection wavelength: 254 nm; flow 1.0 mL/min; buffer A: 0.1% (v/v) TFA in water; buffer B: methanol; elution condition: 0–2 min, buffer B: 5–60%; 2–18 min, buffer B: 60–95%; 18–20 min, buffer B: 95–5%.

HPLC analysis of the light-triggered **TD-MSe2**. **TD-MSe2** (0.5 mM) in degassed and sealed PBS buffer (200 mM, pH 7.4, containing 30% CH_3CN) was activated by 365 nm UV-irradiation for a certain time, and then the reaction solution was subjected to HPLC analysis. HPLC conditions are the same as the description for the stability tests.

HPLC calibration curve of **5.** Different concentrations of **5** (100–600 μM) were dissolved in degassed PBS buffer (200 mM, containing 30% CH_3CN), and aliquots of the solutions were directly analyzed by HPLC to obtain the peak areas of **5**. HPLC conditions: detection wavelength: 254 nm. HPLC conditions are the same as description for the

stability tests of **TD-MSe2**. All HPLC measurements were repeated three times. These mean values of the peak areas were plotted against the used amounts of **5**, and the calibration curve was calculated by linear regression to provide y = -122.19 + 8.65*x.

Light 'on-off' experiments. A 0.5 mM solution of **TD-MSe2** was prepared in degassed and sealed PBS buffer (200 mM, pH 7.4, containing 30% CH_3CN) in a 1.5 mL eppendorf tube. For the light 'on' condition, the solution was placed under a 365 nm UV lamp and irradiated for 0.5, 1, 1.5, 2, 3, and 5 min. For 'dark' condition, the sample was wrapped with aluminum foil and kept inside a dark cabinet for 30 min. The sample was analyzed by HPLC before and after each incubation cycle of lights 'on' or lights 'off' to determine the peak areas of **5** and **TD-MSe2**. HPLC conditions are the same as the above description for the stability tests of **TD-MSe2**. The corresponding **5** concentration was calculated from the calibration curve. Then it was used to quantify the released efficiency of **5** from the light-triggered **TD-MSe2**.

Time-dependent HPLC analysis of the hNQO1-triggered **TD-MSe3.** The enzymatic reaction of **TD-MSe3** (0.15 mM) and hNQO1 (10 μg/mL) in sealed, degassed PBS buffer (50 mM, pH 7.4, containing 0.5 mM NADH, 30 μg/mL BSA and 10% CH_3CN) at 25 °C was analyzed by HPLC at different reaction time. Control compounds **5**, **11** and **TD-MSe3** were also checked for comparison. HPLC conditions are the same as the description for the stability test of **2**.

HPLC calibration curve of **11.** Different concentrations of **11** (50–250 μM) were dissolved in degassed PBS buffer (50 mM, containing 10% CH_3CN), and aliquots of the solutions were directly analyzed by HPLC to obtain the peak areas of **11**. HPLC conditions are the same as description for the stability tests of **2**. All HPLC measurements were repeated three times. These mean values of the peak areas were plotted against the used amounts of **11**, and the calibration curve was obtained as y = -10.87 + 3.84*x.

Quantitative determination of **11** releasing from **TD-MSe3**. **TD-MSe3** (0.11 mM) was co-incubated with hNQO1 (10 μg/mL) in sealed, degassed PBS buffer (50 mM, pH 7.4, containing 0.5 mM NADH, 30 μg/mL BSA and 10% CH_3CN) for 1 h. Then, aliquots of the reaction solutions were subjected to HPLC tests to determine the peak area of **11**. HPLC conditions are the same as the description for the stability test of **2**. All measurements were repeated three times to obtain the mean peak area of **11**. The corresponding **11** concentrations were calculated from the above calibration curve.

HRMS tests.

HRMS analysis of **1** triggered by TCEP. Compound **1** (1 mM) was co-incubated with TCEP (10 mM) in PBS buffer (50 mM, pH 7.4, containing 30% CH_3CN) overnight at room temperature, and the resultant solution was directly used for HRMS tests.

HRMS analysis of the self-decomposition of **2**. Compound **2** (5 mM) in PBS buffer (50 mM, pH 7.4, containing 50% CH_3CN) was incubated at room temperature for 1.5 h, and the resultant solution was directly subjected to HRMS tests.

HRMS analysis of the self-decomposition of **TD-MSe1**. Compound **TD-MSe1** (5 mM) in PBS buffer (50 mM, pH 7.4, containing 50% CH_3CN) was incubated at room temperature for 1 h, and the resultant solution was directly subjected to HRMS test. In addition, **TD-MSe1** (0.5 mM) was co-incubated with **Sel-green** (0.5 mM) in PBS buffer (50 mM, pH 7.4, containing 50% CH_3CN) for 0.5 h, and the resultant solution was directly subjected to HRMS tests. Considering selenol can be trapped by iodoacetamide,[4] we incubated **TD-MSe1** (0.3 mM) with iodoacetamide (0.5 mM) in PBS buffer (50 mM, pH 7.4, containing 50% CH_3CN) for 1 h, and the resultant solution was directly subjected to HRMS tests.

HRMS analysis of the light-triggered **TD-MSe2**. Compound **TD-MSe2** (0.5 mM) in PBS buffer (50 mM, pH 7.4, containing 50% CH_3CN) was activated by 365 nm UV light for 30 min before HRMS test. In addition, **TD-MSe2** (1 mM) and (4-(bromomethyl)phenyl)methanol (10 mM) in PBS buffer (50 mM, pH 7.4, containing 50% CH_3CN) were activated by 365 nm UV light for 30 min, and the resultant solution was directly subjected to HRMS test.

HRMS analysis of the bromobimane-trapped CH_3SeH from light-triggered **TD-MSe2**. A device was connected by a gas flow meter, a round-bottom flask, and a three-neck flask. The **TD-MSe2** (30 mg) in 30 mL PBS buffer (50 mM, pH 7.4, containing 30% CH_3CN and 5 mM 1,4-dithiothreitol) and bromobimane (1 mM) in 5 mL PBS buffer (50 mM, pH 9.5, containing 30% CH_3CN) were placed in the three-necked flask and the round-bottom flask, respectively. Subsequently, the donor was activated under 365 nm UV light for 2 h, and continuous nitrogen (N_2) flow was introduced into the flask to purge CH_3SeH into the bromobimane solution. The resultant bromobimane solution was used for HRMS test.

HRMS analysis of the hNQO1-triggered **TD-MSe3**. A mixture of the donor **TD-MSe3** (0.25 mM), hNQO1 (10 μg/mL), NADH (1 mM) and BSA (1 mg/mL) was incubated in PBS (50 mM, pH 7.4, containing 10% CH_3CN) at room temperature for 1 h. The reaction mixture was filtrated, and the filtrate was used for HRMS test.

HRMS analysis of bromobimane-trapped CH_3SeH from hNQO1-triggered **TD-MSe3**. A mixture of the donor (0.25 mM), hNQO1 (10 μg/mL), NADH (1 mM), BSA (1 mg/mL), and bromobimane (0.5 mM) was incubated in PBS buffer (50 mM, pH 7.4, containing 10% CH_3CN) at room temperature for 1 h. The reaction mixture was filtrated, and the filtrate was used for HRMS test.

Time-dependent NMR tests.

For the ^{1}H NMR test, donor **1** (5 mM) in a mixture of 150 μL CD_3CN and 350 μL D_2O-based PBS (100 mM, pH 7.4) in a sealed NMR tube was monitored by ^{1}H NMR spectroscopy on a Bruker 400 spectrometer at room temperature. Then, TCEP (final 25 mM) was added to the donor solution, and the resultant reaction was monitored by time-dependent ^{1}H NMR spectroscopy.

For the $^{77}Se\{^{1}H\}$ NMR test, the donor **1** (50 mM) in a mixture of 300 μL CD_3CN and 300 μL D_2O-based PBS (100 mM, pH 7.4) in a sealed NMR tube was monitored by $^{77}Se\{^{1}H\}$ spectroscopy on a Bruker 600 spectrometer at room temperature. Then, TCEP (final 100 mM) was added to the donor solution, and the resultant reaction was further monitored by time-dependent $^{77}Se\{^{1}H\}$ NMR spectroscopy.

The donor **TD-MSe2** (160 mM) in 0.60 mL CD_3CN in a sealed NMR tube (Wilmad Quartz NMR tube) was exposed to 365 nm UV light for a certain irradiation time. The reaction was then monitored by $^{1}H/^{13}C\{^{1}H\}/^{77}Se\{^{1}H\}$ NMR spectroscopy on a Bruker 600 spectrometer at room temperature. The reaction time means the total time of light irradiation.

Cell culture and confocal fluorescence imaging.

The human cervical cancer cell line (HeLa, RRID: CVCL_0030) and human umbilical vein endothelial cell line (HUVEC, RRID: CVCL_9Q53) were cultured in DMEM medium, while human colorectal adenocarcinoma cell line (HT-29, RRID: CVCL_0320) was cultured in McCoy's 5A medium. All media were supplemented with 10% fetal bovine serum (FBS), penicillin (100 U/mL) and streptomycin (100 μg/mL). All cell lines were maintained at 37 °C in a humidified incubator with 5% CO_2.

Fluorescent imaging of CH_3SeH delivery from **TD-MSe1**. HeLa cells were used to evaluate donor **TD-MSe1** by cell imaging. Briefly, the cover-slips were added to a 24-well plate, and then the cells were transferred to the 24-well plate and cultured for 24 h to a density of 1×10^5 cells per well before further experiments. After incubation with probe **Sel-green** (10 μM) for 20 min, the cells were washed with PBS buffer. The probe-treated cells were then incubated with **TD-MSe1** (0, 5, 20 μM) for another 30 min. After washing with PBS, the cells were fixed with 4% paraformaldehyde for 15 min, and mounted onto slides using an anti-fade mounting medium. The fluorescence images were captured with a confocal microscope (Olympus FV1000) with a 40 × objective using the green fluorescence channel.

To investigate the time-dependent CH_3SeH release via cell imaging, HeLa cells were first pre-incubated with 10 μM **Sel-green** for 20 min, and then washed with PBS to remove the excess probe. Subsequently, the cells were divided into three groups and incubated with fresh culture medium (control), 10 μM **TD-MSe1**, or 20 μM compound **3**, respectively. At predetermined time points (10, 30, and 60 min), the cells were washed with PBS, fixed with 4% paraformaldehyde, and mounted onto glass slides. Fluorescence

images were acquired using a confocal microscope with identical acquisition settings as described above.

ROS assay with **DCFH-DA**. The cell-trappable reactive oxygen species (ROS) probe 2′,7′-dichlorofluorescin diacetate (**DCFH-DA**) was dissolved in DMSO to prepare a 5 mM stock solution.[5] Four sets of experiments were performed as follows: (1) HeLa cells were incubated with **DCFH-DA** (5 μM) for 30 min, and then washed with PBS to remove the excess probe. (2) HeLa cells were first pre-incubated with 5 μM **DCFH-DA** for 30 min, and then washed with PBS. Subsequently, the cells were treated with H_2O_2 (500 μM) for 30 min. (3) HeLa cells were pretreated with **TD-MSe2** (100 μM) for 2 h and then irradiated with UV light (365 nm, 120 s). After irradiation, cells were incubated with **DCFH-DA** (5 μM) for 30 min, washed with PBS to remove excess probe, and subsequently incubated with H_2O_2 (500 μM) for 30 min. (4) The cells were treated under the same conditions as group (3), except that the UV irradiation was omitted. After washing with PBS, the cells were fixed with 4% paraformaldehyde and mounted onto glass slides using an anti-fade mounting medium. The fluorescence images were acquired using a confocal microscope (Axio-Imager_LSM-800) with a 40 × objective.

Relative expression levels of hNQO1 in different cell lines via **Nap-13**. The hNQO1 probe **Nap-13** (from our previous work [3]) was dissolved in DMSO to prepare a 5 mM stock solution. For the endogenous hNQO1 imaging, HT-29, HUVEC and HeLa cells were incubated with probe **Nap-13** (10 μM) at 37 °C for 1 h, and then washed with PBS. For inhibitor-treated bioimaging, HT-29 cells were pretreated with dicoumarol (100 μM) for 30 min and then washed with PBS. The cells were subsequently incubated with probe **Nap-13** (10 μM) for 1 h, and washed with PBS again. All the cells were subsequently fixed with 4% paraformaldehyde and mounted onto glass slides using an anti-fade mounting medium. Fluorescence images were acquired using a confocal microscope (Axio-Imager_LSM-800) with a 40 × objective.

Fluorescent imaging for intracellular CH_3SeH delivery from **TD-MSe3**. HT-29 cells[3] were used to evaluate the endogenous enzyme-triggered donor (**TD-MSe3**) by cell imaging. Briefly, the cells were transferred to a 12-well plate and cultured for 24 h to a density of 2×10^5 cells per well before the experiments. After that, the cellular imaging experiments were performed under the following three conditions: (1) Cells were incubated with **Sel-green** (10 μM) for 1 h. (2) Cells were co-incubated with **Sel-green** (10 μM) and **TD-MSe3** (40 μM) for 1 h. (3) Cells were pre-treated with the inhibitor dicoumarol (200 μM) for 0.5 h, followed by co-incubation with **Sel-green** and **TD-MSe3** for an additional 1 h. After washing with PBS, the cells were fixed with 4% paraformaldehyde and mounted onto glass slides using an anti-fade mounting medium. The fluorescence images were acquired using a confocal microscope (Axio-Imager_LSM-800) with a 40 × objective.

To verify that the intracellular release of CH_3SeH from **TD-MSe3** is hNQO1-dependent, both HT-29 cells (high hNQO1 expression) and HeLa cells (low hNQO1 expression)[3] were treated with **TD-MSe3** (20 μM) in the presence of the CH_3SeH-responsive probe

Sel-green (10 μM) for 2 h. The fluorescence images were subsequently acquired as described above.

Cytotoxicity assay

The cytotoxicity of donor **TD-MSe2** was assessed in HeLa cells using the Cell Counting Kit-8 (CCK-8) assay. Briefly, cells were seeded in 96-well plates at a density of 1.2×10^4 cells per well and allowed to adhere overnight. Three sets of experiments were performed as follows:

(1) Intrinsic cytotoxicity assessment. Cells were subjected to the following conditions: (a) treatment with 150 μM **TD-MSe2** for 2 h (control 1); (b) pretreatment with 150 μM **TD-MSe2** for 2 h followed by UV (365 nm) irradiation for 120 s (control 2); (c) treatment with 400 μM H_2O_2 for 2 h (control 3).

(2) Concentration-dependent cytoprotective activity. Cells were pretreated with **TD-MSe2** (30 or 100 μM) for 2 h and washed with PBS. For each concentration, cells were divided into three groups: a control group without light exposure, and two experimental groups subjected to UV light (365 nm) for 60 s or 120 s, respectively. After irradiation, H_2O_2 (400 μM) was added, and the cells were incubated for an additional 2 h.

(3) Irradiation-time-dependent antioxidant activity. Cells were pretreated with **TD-MSe2** (150 μM) for 2 h and washed with PBS. In the control group, cells were directly incubated with H_2O_2 (400 μM) for 2 h without UV exposure. In the experimental groups, cells were exposed to UV light (365 nm) for 5, 15, 30, 60, and 120 s, prior to the addition of H_2O_2 (400 μM), followed by incubation for 2 h. After all treatments, CCK-8 solution was added to each well, and the absorbance was measured at 450 nm according to the manufacturer's instructions by the Thermo Scientific Multiskan GO microplate spectrophotometer. All experiments were independently repeated three times (n = 3).

The cytotoxicity of the donor **TD-MSe3** and control molecule **3** were evaluated in HT-29 cells using the CCK-8 assay. Additionally, the toxicity of the donor was assessed in HeLa cells and HUVEC cells as controls. Cells were seeded in 96-well plates at a density of 1.2×10^4 cells per well and allowed to adhere overnight. Subsequently, the culture medium was replaced with fresh medium containing increasing concentrations of the tested compounds (0, 1, 5, 10, 20, 40, 70, 100, and 150 μM). After 72 h of incubation, cell viability was determined using the CCK-8 assay. All experiments were performed in triplicate. The half-maximal inhibitory concentration (IC_{50}) values were determined by non-linear regression analysis.

Colony formation assay.

HT-29 cells were seeded in six-well plates at a density of 3×10^3 cells per well and incubated overnight for adhesion. Cells were then incubated with culture medium containing either 0.1% DMSO or **TD-MSe3** at different concentrations (0, 2, 10, or 40

μM), and the medium was refreshed every three days. After 19 days of culture, colonies were fixed with 4% paraformaldehyde (Leagene Biotechnology) at 4 °C for 15 min and then stained with 0.1% crystal violet (Beyotime Institute of Biotechnology) for 5 min at room temperature.[6] The plates were then imaged with a camera, and colony numbers were quantified using ImageJ software (NIH). All experiments were independently repeated three times (n = 3).

4. Detailed Synthetic Procedures and Characterization Data for All Compounds

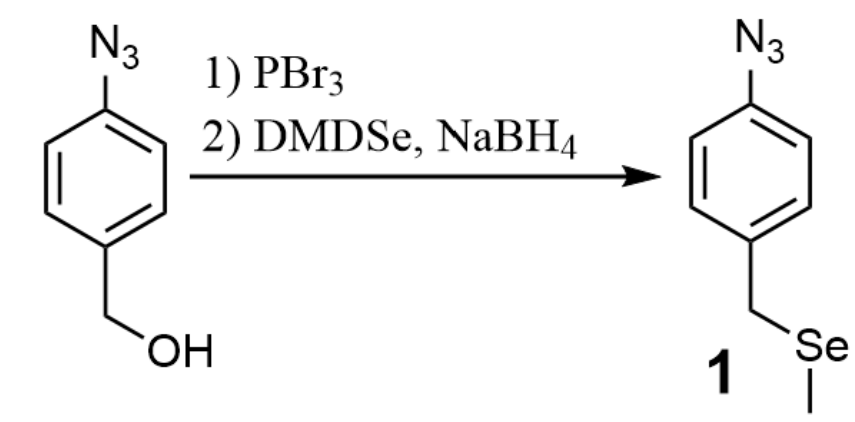

Synthesis of 1. (4-Azidophenyl)methanol (316 mg, 2.1 mmol) was dissolved in CH_2Cl_2 (10 mL) and cooled to 0 ºC, and then phosphorus tribromide (PBr_3, 1.5 equiv) was added. The reaction was warmed to room temperature and stirred for 5 h. The reaction was quenched with water and extracted twice with ethyl acetate (EtOAc). The organic layers were dried over Na_2SO_4 and concentrated under reduced pressure, and the resultant residue was dissolved in tetrahydrofuran (THF, 10 mL). On the other hand, dimethyldiselenide (DMDSe, 226 mg, 1.2 mmol) was dissolved in ethanol (EtOH, 10 mL), and then sodium borohydride ($NaBH_4$, 91 mg, 2.4 mmol) was added in portions. After stirring at room temperature under an argon atmosphere for 20 min, the above THF solution was added dropwise. The reaction mixture was stirred at room temperature until the reaction is complete. Then, the solution was diluted with water, and the organic product was extracted by EtOAc three times. The combined organic layer was washed by water and brine, dried over anhydrous Na_2SO_4, and concentrated under reduced pressure. The crude residue was purified by silica gel column chromatography to give **1** as a colorless oil (230 mg, yield: 48% over 2 steps). ^{1}H NMR (400 MHz, acetonitrile-d_3) δ 7.11–7.05 (m, 2H), 6.81–6.75 (m, 2H), 3.53 (s, 2H), 1.68 (s, 3H). ^{13}C{^{1}H} NMR (101 MHz, acetonitrile-d_3) δ 139.2, 138.0, 131.2, 120.0, 29.9, 4.3. ^{77}Se{^{1}H} NMR (114 MHz, water-d_2) δ (169.3). IR (KBr) 2108 cm^{-1} (N_3). HRMS (ESI): m/z $[M+H]^+$ calculated for $C_8H_{12}NSe^+$: 202.0129; found: 202.0142.

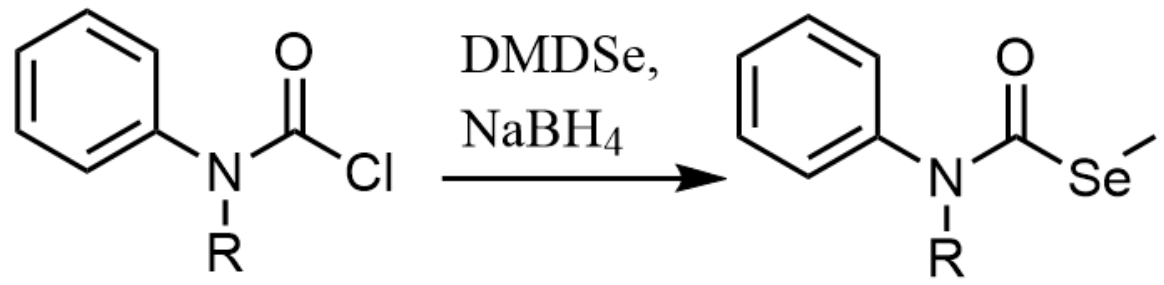

2, R=H
3, R=CH_3

Synthesis of 2. Donor **2** was prepared according to a previous literature.[7] ^{1}H NMR (400 MHz, chloroform-*d*) *δ* 7.43–7.40 (m, 3H), 7.33–7.30 (m, 2H), 7.15–7.11 (m, 1H), 2.34 (s, 3H). ^{13}C{^{1}H} NMR (101 MHz, chloroform-*d*) *δ* 137.7, 129.3, 124.9, 120.1, 6.5. These NMR data of **2** were consistent with a previous literature.[8] HRMS (ESI): m/z [M+H]$^+$ calculated for $C_8H_{10}NOSe^+$: 215.9922; found: 215.9934.

Synthesis of 3. DMDSe (200 mg, 1.06 mmol) was dissolved in ethanol (EtOH, 5 mL), and then $NaBH_4$ (81 mg, 2.1 mmol) was added in portions. After stirring at room temperature under an argon atmosphere for 20 min, a solution of *N*-methyl-*N*-phenylcarbamoyl chloride (188 mg, 1.1 mmol) in THF (5 mL) was added slowly to the reaction mixture. The resultant reaction solution was stirred for another 2 h, and then the organic solution was mixed with brine, and the product was exacted by EtOAc. The combined organic layers were dried over anhydrous Na_2SO_4 and concentrated under reduced pressure. The crude product was purified by column chromatography with petroleum ether/EtOAc (20/1) to give **3** as a light yellow oil (210 mg, yield: 84%). ^{1}H NMR (400 MHz, chloroform-*d*) *δ* 7.45–7.37 (m, 3H), 7.32–7.27 (m, 2H), 3.33 (s, 3H), 2.15 (s, 3H). ^{13}C{^{1}H} NMR (101 MHz, chloroform-*d*) *δ* 165.9, 142.3, 129.6, 128.9, 128.7, 38.3, 7.1. ^{77}Se{^{1}H} NMR (114 MHz, chloroform-*d*) *δ* 324.1. HRMS (ESI): m/z [M+H]$^+$ calculated for $C_9H_{12}NOSe^+$: 230.0079; found: 230.0084.

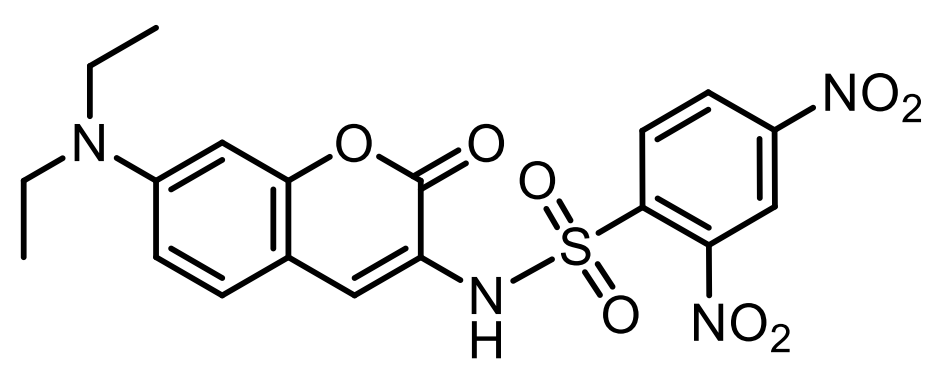

Sel-green

Synthesis of probe Sel-green. Probe **Sel-green** was prepared according to a previous literature.[9] ^{1}H NMR (400 MHz, acetonitrile-*d*$_3$) *δ* 8.62 (d, *J* = 2.2 Hz, 1H), 8.43 (dd, *J* = 8.7, 2.2 Hz, 1H), 8.21 (d, *J* = 8.7 Hz, 1H), 8.00 (bs, 1H), 7.81 (s, 1H), 7.39 (d, *J* = 8.9 Hz, 1H), 6.70 (dd, *J* = 8.9, 2.5 Hz, 1H), 6.46 (d, *J* = 2.3 Hz, 1H), 3.41 (q, *J* = 7.1 Hz, 4H), 1.14 (q, *J* = 7.1 Hz, 6H). ^{13}C{^{1}H} NMR (101 MHz, acetonitrile-*d*$_3$) *δ* 160.5, 156.1, 152.1, 151.4, 148.9, 138.4, 138.1, 133.7, 130.5, 128.1, 121.5, 115.4, 110.6, 108.3, 97.6, 45.4, 12.6. HRMS (ESI): m/z [M+H]$^+$ calculated for $C_{19}H_{19}N_4O_8S^+$: 463.0918; found: 463.0921.

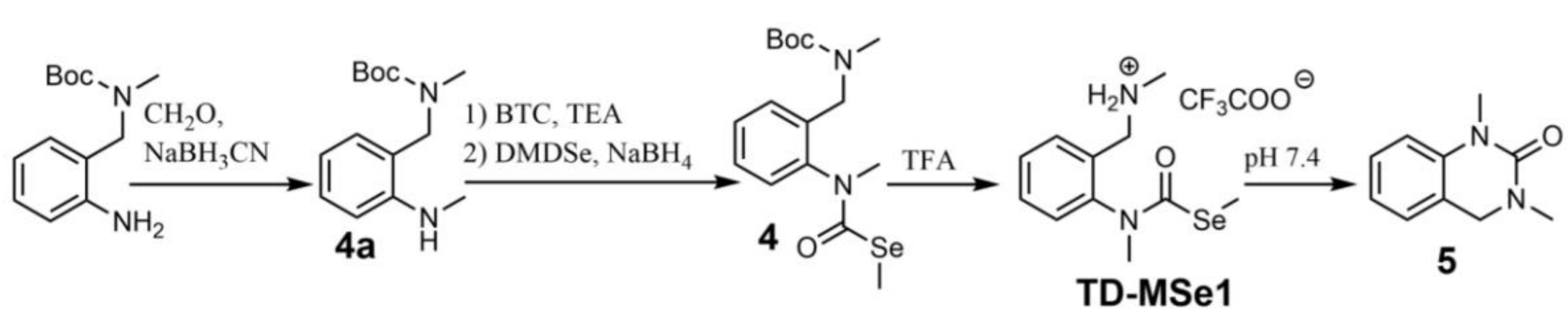

Synthesis of 4a. To a solution of *tert*-butyl (2-(methylamino)benzyl)carbamate (372 mg, 1.58 mmol) in methanol (10 mL) at 0 ºC, TFA was added to adjust pH to about 5.0. Then, $NaBH_3CN$ (148.7 mg, 2.36 mmol) and HCHO (1.9 mmol) were added, and the reductive

amination reaction was stirred at room temperature overnight.[10] After removing the solvent under reduced pressure, the residue was dissolved in EtOAc, and the resultant solution was washed with water and brine, dried over Na_2SO_4, and concentrated under reduced pressure. The crude residue was purified by silica gel column chromatography with petroleum ether/EtOAc (100/3) to give **4a** as a light-yellow oil (196 mg, yield: 50%). ^{1}H NMR (400 MHz, chloroform-*d*) δ 7.29–7.25 (m, 1H), 7.05 (d, J = 7.1 Hz, 1H), 6.67–6.63 (m, 2H), 5.53 (bs, 1H), 4.37 (s, 2H), 2.89 (s, 3H), 2.78 (s, 3H), 1.55 (s, 9H). ^{13}C{^{1}H} NMR (101 MHz, chloroform-*d*) δ 156.5, 148.2, 131.3, 129.5, 120.2, 115.1, 109.3, 80.1, 50.0, 32.9, 30.2, 28.4. HRMS (ESI): m/z $[M+H]^+$ calcd. for $C_{14}H_{23}N_2O_2^+$: 251.1754; found: 251.1766.

Synthesis of 4. Compound **4a** (80 mg, 0.32 mmol) was dissolved in CH_2Cl_2 (10 mL) and cooled to 0 ºC, and then triethylamine (TEA, 2.0 equiv) was added followed by triphosgene (BTC, 0.5 equiv). The reaction was warmed to room temperature and stirred until completion as indicated by TLC. The reaction was quenched with water and extracted twice with CH_2Cl_2. The organic layers were dried over Na_2SO_4 and concentrated under reduced pressure, and the resultant residue was dissolved in THF (3 mL). On the other hand, DMDSe (60 mg, 0.32 mmol) was dissolved in EtOH (5 mL), and then $NaBH_4$ (24 mg, 0.64 mmol) was added in portions. After stirring at room temperature under an argon atmosphere for 15 min, the above THF solution was added dropwise. The resultant mixture was stirred at room temperature for 2 h. Then, the solution was diluted with water, and the organic product was extracted by EtOAc three times. The combined organic layers were washed by water and brine, dried over anhydrous Na_2SO_4, and concentrated under reduced pressure. The crude residue was purified by silica gel column chromatography with petroleum ether/EtOAc (10/1) to give **4** as a light yellow oil (110 mg, yield: 92% over 2 steps). ^{1}H NMR (400 MHz, chloroform-*d*) δ 7.43–7.39 (m, 1H), 7.32 (s, 1H), 7.24–7.20 (m, 2H), 4.62–4.30 (m, 2H), 3.23 (s, 3H), 2.83 (s, 3H), 2.12 (s, 3H), (1.49, 1.42) (s, 9H). ^{13}C{^{1}H} NMR (101 MHz, chloroform-*d*) δ 166.0, (156.4, 155.9), 139.6, (137.5, 137.4), (130.3, 129.9), (128.6, 128.4), 128.0, 80.0, (48.9, 47.9), 37.4, (34.7, 34.5), 28.5, 6.9. ^{77}Se{^{1}H} NMR (114 MHz, chloroform-*d*) δ 326.3. HRMS (ESI): m/z $[M+Na]^+$ calculated for $C_{16}H_{24}N_2NaO_3Se^+$: 395.0844; found: 395.0846.

Synthesis of TD-MSe1. To a solution of **4** (82 mg, 0.22 mmol) in CH_2Cl_2 (6 mL), triethylsilane (TES, 0.5 mL) and TFA (2 mL) were added at room temperature. The resultant solution was stirred at room temperature for 0.5 h, and then the solvent was removed under reduced pressure to obtain **TD-MSe1** as a light-yellow oil (80 mg, yield: 94%). ^{1}H NMR (400 MHz, chloroform-*d*) δ 8.33 (bs, 1H), 8.19–7.93 (m, 1H), 7.55–7.35 (m, 3H), 4.24–3.97 (m, 2H), (3.39, 3.30) (s, 3H), 2.79 (s, 3H), (2.30, 2.17) (s, 3H). ^{13}C{^{1}H} NMR (101 MHz, chloroform-*d*) δ 161.5, 143.0, 132.4, (131.3, 131.1), 130.8, 129.3, 126.8, 115.5 (q, $^{1}J_{C\text{-}F}$ = 278.4 Hz), (49.9, 48.6), (39.4, 38.6), (33.7, 33.3), (7.7, 7.4). ^{19}F{^{1}H} NMR (376 MHz, chloroform-*d*) δ −75.9. ^{77}Se{^{1}H} NMR (114 MHz, chloroform-*d*) δ (331.5, 329.7). HRMS (ESI): m/z $[M+H]^+$ calculated for $C_{11}H_{17}N_2OSe^+$: 273.0501; found: 273.0504.

Synthesis of 5 from self-decomposition of TD-MSe1 in water at pH 7.4. TD-MSe1 (55 mg, 0.14 mmol) was dissolved in 30 mL PBS solution (200 mM, pH 7.4, containing 10% CH_3CN) and stirred for 1 h at room temperature. Then, CH_3CN was removed under reduced pressure, and the residue was diluted with water. The organic product was extracted by EtOAc three times, and the combined organic layers were washed by water and brine, dried over anhydrous Na_2SO_4, and concentrated under reduced pressure. A colorless oily substance **5** (26 mg, 0.14 mmol, quantitative yield) was obtained. ^1H NMR (400 MHz, chloroform-*d*) δ 7.29–7.19 (m, 1H), 7.04 (d, J = 7.2 Hz, 1H), 7.00–6.92 (m, 1H), 6.82 (d, J = 8.2 Hz, 1H), 4.34 (s, 2H), 3.30 (s, 3H), 3.02 (s, 3H). $^{13}C\{^1H\}$ NMR (101 MHz, chloroform-*d*) δ 155.2, 139.6, 128.2, 125.3, 121.6, 119.8, 112.6, 50.3, 35.6, 30.2. HRMS (ESI): m/z $[M+H]^+$ calculated for $C_{10}H_{13}N_2O^+$: 177.1022; found: 177.1049.

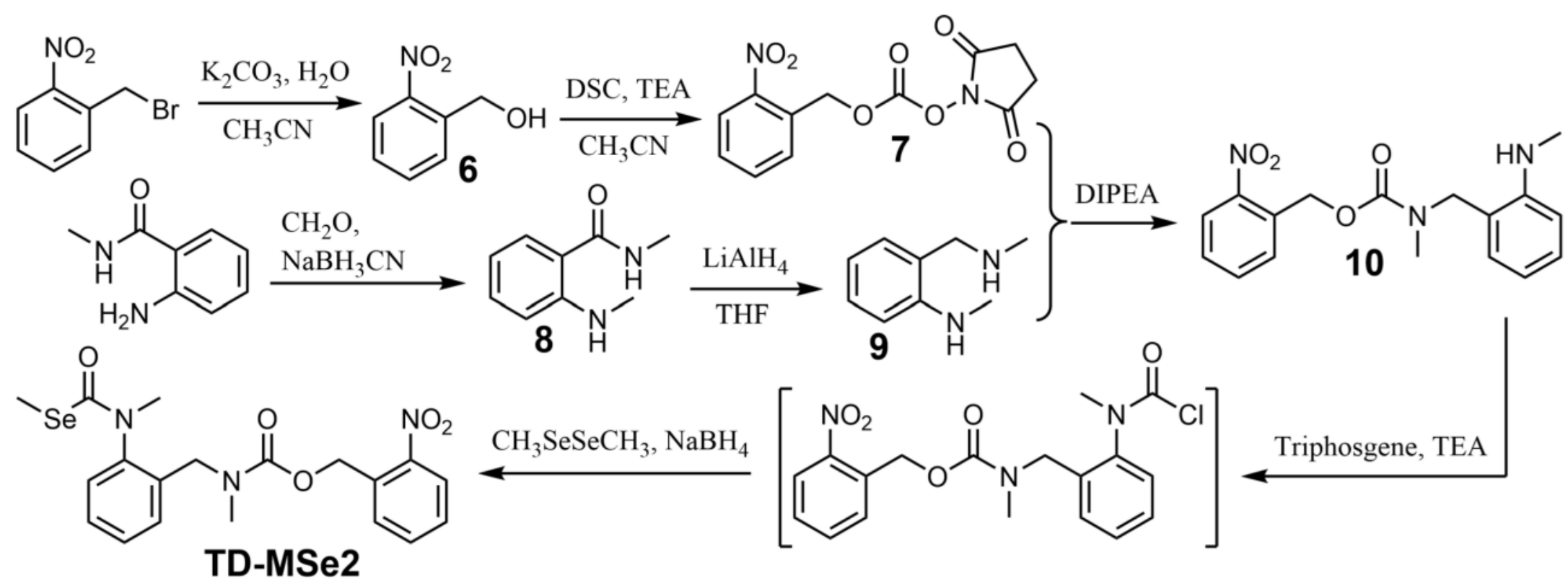

Synthesis of 6. 2-Nitrobenzyl bromide (3.86 g, 17.9 mmol) and K_2CO_3 (12.4 g, 89.8 mmol) in a mixture of water and acetonitrile (V/V = 1:1) were stirred at 90 ºC for 4 h. Then, the reaction solution was mixed with water, and the product was extracted by EtOAc three times. The combined organic layer was dried over anhydrous Na_2SO_4 and concentrated under reduced pressure. The resultant residue was purified by column chromatography with MeOH/CH_2Cl_2 (1/100) to give **6** as a white solid (2.35 g, yield: 86%). ^1H NMR (400 MHz, methanol-d_4) δ 8.05 (d, J = 9.6 Hz, 1H), 7.86 (d, J = 7.8 Hz, 1H), 7.76–7.69 (m, 1H), 7.53–7.47 (m, 1H), 4.96 (s, 2H). HRMS (ESI): m/z $[M–H]^–$ calcd. for $C_7H_6NO_3^–$: 152.0353; found: 152.0348.

Synthesis of 7. Compound **6** (1.53 g, 10.0 mmol) was dissolved in CH_3CN (50 mL), and then *N,N'*-disuccinimidyl carbonate (DSC, 5.12 g, 20.0 mmol) and TEA (3.36 mL, 24.0 mmol) were slowly added to the reaction mixture. The resultant mixture was stirred under N_2 atmosphere at room temperature overnight, and then the organic solvent was removed under reduced pressure. The resultant residue was purified by column chromatography with petroleum ether/EtOAc (2/1) to give **7** as a white solid (2.81 g, yield: 96%). ^1H NMR (400 MHz, chloroform-*d*) δ 8.19 (d, J = 8.2 Hz, 1H), 7.77–7.67 (m, 2H), 7.59–7.51 (m, 1H), 5.78 (s, 2H), 2.85 (s, 4H).

Synthesis of 8. To a solution of 2-amino-*N*-methylbenzamide (12 g, 80 mmol) in methanol (60 mL) at 0 ºC, TFA was added to adjust pH to about 5.0. Then, CH_2O (7 mL, 96 mmol) and $NaBH_3CN$ (7.5 g, 120 mmol) were added, and the reductive amination reaction was stirred at room temperature overnight.[10] After removing the solvent under

reduced pressure, the residue was dissolved in EtOAc, and the resultant solution was washed with water and brine, dried over Na_2SO_4, and concentrated under reduced pressure. The crude residue was purified by silica gel column chromatography to give **8** as a light-yellow oil (4.6 g, yield: 35%). ^{1}H NMR (400 MHz, chloroform-*d*) δ 7.31–7.28 (m, 2H), 6.65 (d, *J* = 8.7 Hz, 1H), 6.57–6.53 (m, 1H), 6.25 (bs, 1H), 2.91 (d, *J* = 4.9 Hz, 3H), 2.83 (s, 3H). ^{13}C{^{1}H} NMR (101 MHz, chloroform-*d*) δ 170.8, 150.3, 132.8, 127.3, 115.4, 114.7, 111.2, 29.8, 26.6. HRMS (ESI): m/z $[M+H]^+$ calcd. for $C_9H_{13}N_2O^+$: 165.1022; found: 165.1031.

Synthesis of 9. To a solution of **8** (4.6 g, 28 mmol) in anhydrous THF (30 mL) at 0 ºC, $LiAlH_4$ (44 mL, 2.5 M in THF) was added dropwise.[11] The reaction mixture was refluxed for 8 h. Then, the reaction was quenched with water and extracted twice with CH_2Cl_2. The combined organic layers were dried over Na_2SO_4 and concentrated under reduced pressure, and the resultant residue was purified by silica gel column chromatography to give **9** as a light-yellow oil (3.9 g, yield: 93%). ^{1}H NMR (400 MHz, chloroform-*d*) δ 7.36–7.24 (m, 1H), 7.13 (d, *J* = 9.2 Hz, 1H), 6.79–6.70 (m, 2H), 3.83 (s, 2H), 2.94 (s, 3H), 2.50 (s, 3H). ^{13}C{^{1}H} NMR (101 MHz, chloroform-*d*) δ 149.3, 129.5, 128.6, 123.4, 115.9, 109.5, 55.4, 35.9, 30.1. HRMS (ESI): m/z $[M+H]^+$ calcd. for $C_9H_{15}N_2^+$: 151.1230; found: 151.1235.

Synthesis of 10. Compound **7** (1.18 g, 4.0 mmol) and *N,N*-diisopropylethylamine (DIPEA, 1.04 mL, 6.0 mmol) were dissolved in 15 mL DMF, and then a solution of **9** (0.9 g, 6 mmol) in 5 mL DMF was added. The reaction solution was stirred at room temperature overnight. Then the solution was diluted with water, and the organic product was extracted by EtOAc three times. The resultant organic layer was washed by water and brine, dried over anhydrous Na_2SO_4, and concentrated under reduced pressure. The crude product was purified by column chromatography on silica gel (CH_2Cl_2/CH_3OH = 200:1), and **10** was obtained as a yellow solid (0.89 g, yield: 68%). ^{1}H NMR (400 MHz, chloroform-*d*) δ 8.11 (d, *J* = 8.2 Hz, 1H), 7.68–7.61 (m, 1H), 7.56 (d, *J* = 6.3 Hz, 1H), 7.52–7.45 (m, 1H), 7.29–7.22 (m, 1H), 7.05 (d, *J* = 5.7 Hz, 1H), 6.67–6.58 (m, 2H), 5.60 (s, 2H), 4.40 (s, 2H), 2.89 (s, 3H), 2.78 (s, 3H). ^{13}C{^{1}H} NMR (101 MHz, chloroform-*d*) δ 156.5, 148.1, 147.5, 133.8, 133.3, 131.5, 129.8, 128.6, 125.1, 119.4, 115.4, 109.6, 64.3, 50.6, 32.8, 30.4. HRMS (ESI): m/z $[M+H]^+$ calcd. for $C_{17}H_{20}N_3O_4^+$: 330.1448; found: 330.1458.

Synthesis of TD-MSe2. Compound **10** (329 mg, 1.0 mmol) was dissolved in CH_2Cl_2 (10 mL) and cooled to 0 ºC, and then TEA (2.0 equiv) was added followed by BTC (0.5 equiv). The reaction was warmed to room temperature and stirred for 3 h. The reaction was quenched with water and extracted twice with CH_2Cl_2. The organic layers were dried over Na_2SO_4 and concentrated under reduced pressure, and the resultant residue was dissolved in THF (10 mL). On the other hand, DMDSe (188 mg, 1.0 mmol) was dissolved in EtOH (10 mL), and then $NaBH_4$ (76 mg, 2.0 mmol) was added in portions. After stirring at room temperature under an argon atmosphere for 20 min, the above THF solution was added dropwise. The reaction mixture was stirred at room temperature for 3 h. Then, the solution was diluted with water, and the organic product was extracted by EtOAc three times. The combined organic layer was washed by water and brine, dried

over anhydrous Na_2SO_4, and concentrated under reduced pressure. The crude residue was purified by silica gel column chromatography with petroleum ether/EtOAc (10/3) to give **TD-MSe2** as a light-yellow oil (320 mg, yield: 71% over 2 steps). ^{1}H NMR (400 MHz, chloroform-*d*) δ 8.09–7.96 (m, 1H), 7.62–7.14 (m, 8H), 5.60–5.46 (m, 2H), 4.69–4.34 (m, 2H), 3.27–3.18 (m, 3H), 2.96 (s, 3H), 2.15–2.04 (m, 3H). ^{13}C{^{1}H} NMR (101 MHz, chloroform-*d*) δ 165.9, 156.3, 155.7, 147.6, (139.7, 139.5), (136.7, 136.5), 133.6, 132.8, (130.4, 130.3), 130.0, 129.1, (128.9, 128.7), (128.0, 127.7), 124.9, (64.3, 64.1), 48.6, (37.4, 37.3), (35.3, 34.5), 6.9. ^{77}Se{^{1}H} NMR (114 MHz, chloroform-*d*) δ (327.4, 326.9). HRMS (ESI): m/z $[M+Na]^+$ calcd. for $C_{19}H_{21}N_3NaO_5Se^+$: 474.0539; found: 474.0543.

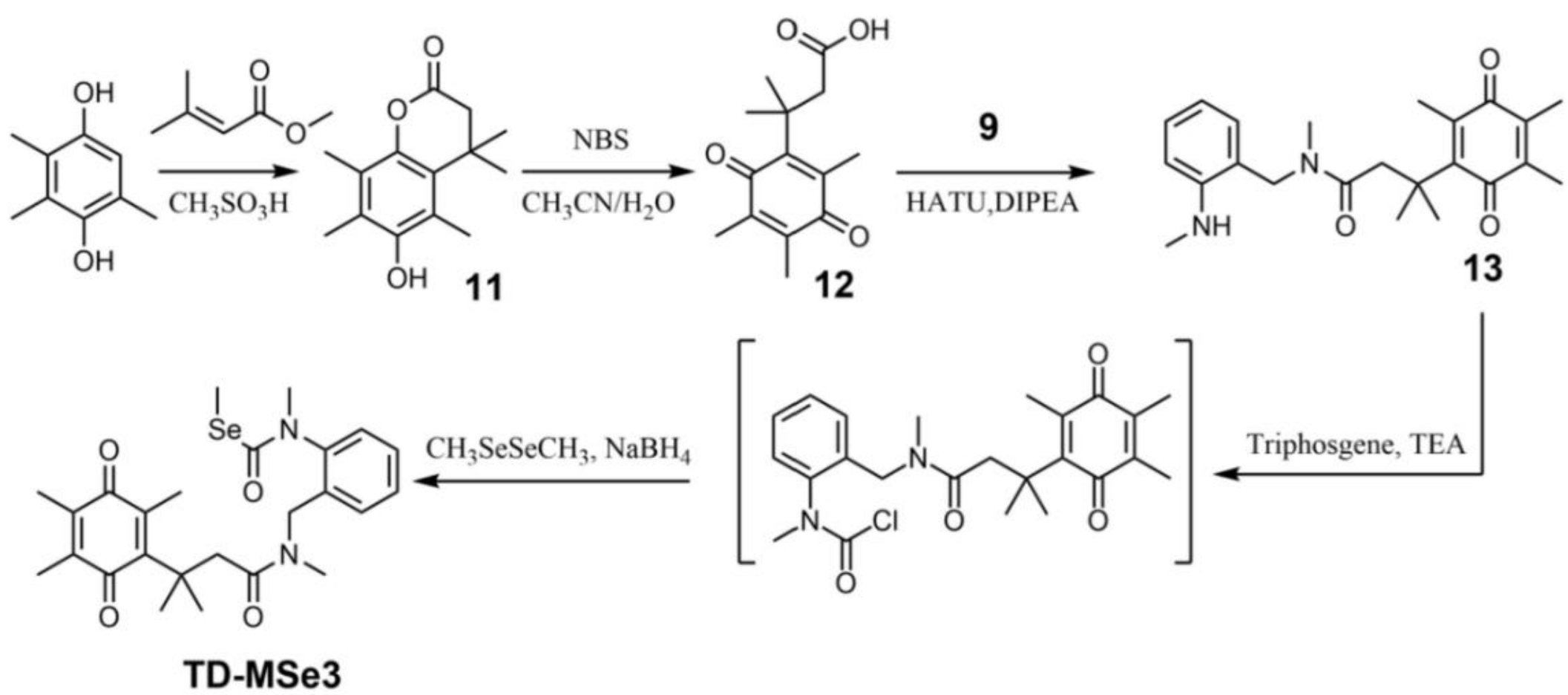

Synthesis of 11. Compound **11** was prepared according to a previous literature.[12] ^{1}H NMR (400 MHz, chloroform-*d*) δ 4.98 (s, 1H), 2.54 (s, 2H), 2.36 (s, 3H), 2.20 (s, 3H), 2.18 (s, 3H), 1.44 (s, 6H). ^{13}C{^{1}H} NMR (101 MHz, chloroform-*d*) δ 169.2, 149.0, 143.5, 128.3, 123.5, 122.2, 119.2, 77.4, 46.2, 35.5, 27.8, 14.6, 12.6, 12.4. HRMS (ESI): m/z $[M+H]^+$ calcd. for $C_{14}H_{19}O_3^+$: 235.1329; found: 235. 1342.

Synthesis of 12. Compound **12** was prepared according to a previous literature.[12] ^{1}H NMR (400 MHz, methanol-*d*$_4$) δ 2.92 (s, 2H), 2.12 (s, 3H), 1.93 (s, 6H), 1.43 (s, 6H). ^{13}C{^{1}H} NMR (101 MHz, methanol-*d*$_4$) δ 192.1, 188.6, 176.1, 154.6, 144.5, 139.4, 139.1, 48.5, 39.0, 29.1, 14.4, 12.6, 12.0. HRMS (ESI): m/z $[M-H]^-$ calcd. for $C_{14}H_{17}O_4^-$: 249.1132; found: 249.1113.

Synthesis of 13. A mixture of **12** (625 mg, 2.50 mmol), 2-(7-azabenzotriazol-1-yl)-*N,N,N′,N′*-tetramethyluronium hexafluorophosphate (HATU, 1.43 g, 1.5 equiv) and DIPEA (1.3 mL, 3 equiv) in DMF was treated with **9** (1.13 g, 3 equiv), and the reaction solution was stirred at room temperature for 4 h. After the coupling reaction, the solvent was removed under reduced pressure. The resultant solid was dissolved in CH_2Cl_2, and the organic layer was washed with water and brine, and dried over anhydrous Na_2SO_4. After removing the organic solvent, the residue was purified by column chromatography with petroleum ether/EtOAc (10/1) to give **13** as a yellow solid (907 mg, yield: 95%). ^{1}H NMR (400 MHz, chloroform-*d*) δ 7.24–7.18 (m, 1H), 7.01 (d, J = 7.4 Hz, 1H), 6.63–6.57 (m, 1H), 6.54 (d, J = 8.2 Hz, 1H), 4.38 (s, 2H), 3.01 (s, 2H), 2.87 (s, 3H), 2.71 (s, 3H),

2.16 (s, 3H), 1.97 (s, 3H), 1.93 (s, 3H), 1.44 (s, 6H). $^{13}C\{^1H\}$ NMR (101 MHz, chloroform-*d*) δ 191.3, 187.3, 172.6, 154.3, 147.9, 143.3, 138.3, 137.0, 131.6, 129.7, 119.6, 115.4, 109.4, 77.4, 48.5, 47.3, 38.2, 34.4, 30.3, 29.0, 14.4, 12.8, 12.3. HRMS (ESI): m/z $[M+H]^+$ calcd. for $C_{23}H_{31}N_2O_3^+$: 383.2329; found: 383.2340.

Synthesis of TD-MSe3. Compound **13** (306 mg, 0.80 mmol) was dissolved in CH_2Cl_2 (10 mL) and cooled to 0 ºC, and then TEA (2.0 equiv) was added followed by BTC (0.5 equiv). The reaction was warmed to room temperature and stirred for 3 h. The reaction was quenched with water and extracted twice with CH_2Cl_2. The organic layers were dried over Na_2SO_4 and concentrated under reduced pressure, and the resultant residue was dissolved in THF (10 mL). On the other hand, DMDSe (150 mg, 0.8 mmol) was dissolved in EtOH (3 mL), and then $NaBH_4$ (15 mg, 0.4 mmol) was added in portions. After stirring at room temperature under an argon atmosphere for 20 min, the solution was added to the above THF solution dropwise. The reaction mixture was stirred at room temperature for 2 h. Then, the solution was diluted with water, and the organic product was extracted by EtOAc three times. The combined organic layer was washed by water and brine, dried over anhydrous Na_2SO_4, and concentrated under reduced pressure. The crude residue was purified by silica gel column chromatography with petroleum ether/EtOAc (100/13) to give **TD-MSe3** as yellow oil (108 mg, yield: 27% over 2 steps). ^1H NMR (400 MHz, chloroform-*d*) δ 7.51–7.06 (m, 4H), 4.84–4.19 (m, 2H), 3.30–2.86 (m, 8H), 2.16–2.11 (m, 6H), 1.95–1.83 (m, 6H), 1.47–1.36 (m, 6H). $^{13}C\{^1H\}$ NMR (101 MHz, chloroform-*d*) δ 191.4, 187.8, 172.9, 166.0, (154.7, 154.6), (143.4, 143.2), (139.7, 139.4), (138.2, 138.1), (136.5, 136.2), (136.5, 136.1), (130.7, 130.3), (130.4, 130.1), (129.2, 128.5), (127.6, 127.0), 77.4, (49.7, 47.9), (47.1, 46.6), (37.8, 37.5, 37.4, 37.3), (35.7, 34.2, 34.0), (29.0, 25.7), (28.6, 25.0), (14.4, 14.2), 12.7, 12.2, 7.1. $^{77}Se\{^1H\}$ NMR (114 MHz, chloroform-*d*) δ (327.6, 326.7). HRMS (ESI): m/z $[M+H]^+$ calcd. for $C_{25}H_{33}N_2O_4Se^+$: 505.1600; found: 505.1610.

5. Supporting NMR, IR and HRMS Spectra

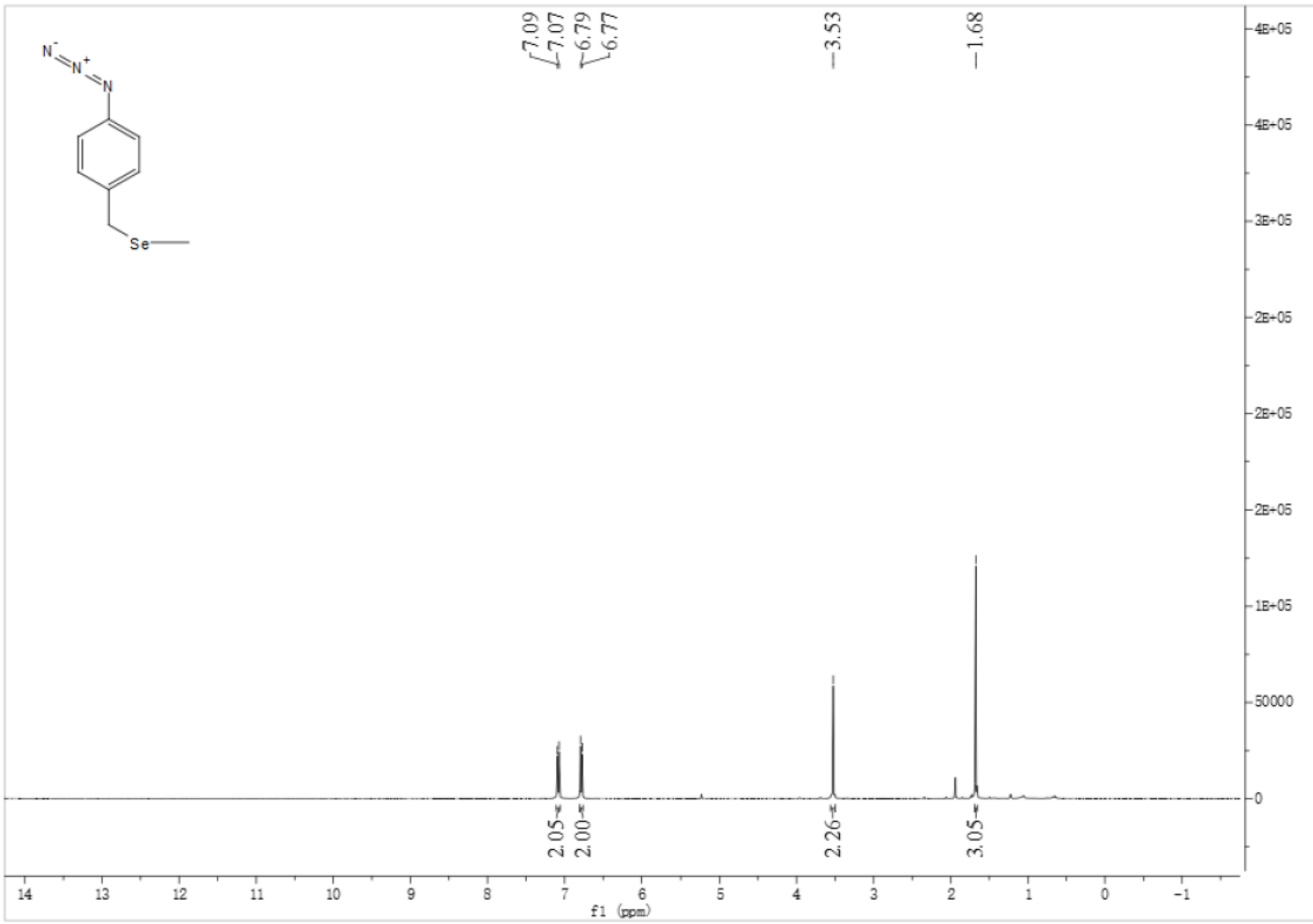

^{1}H NMR (400 MHz, acetonitrile-d_3) spectrum of compound **1**.

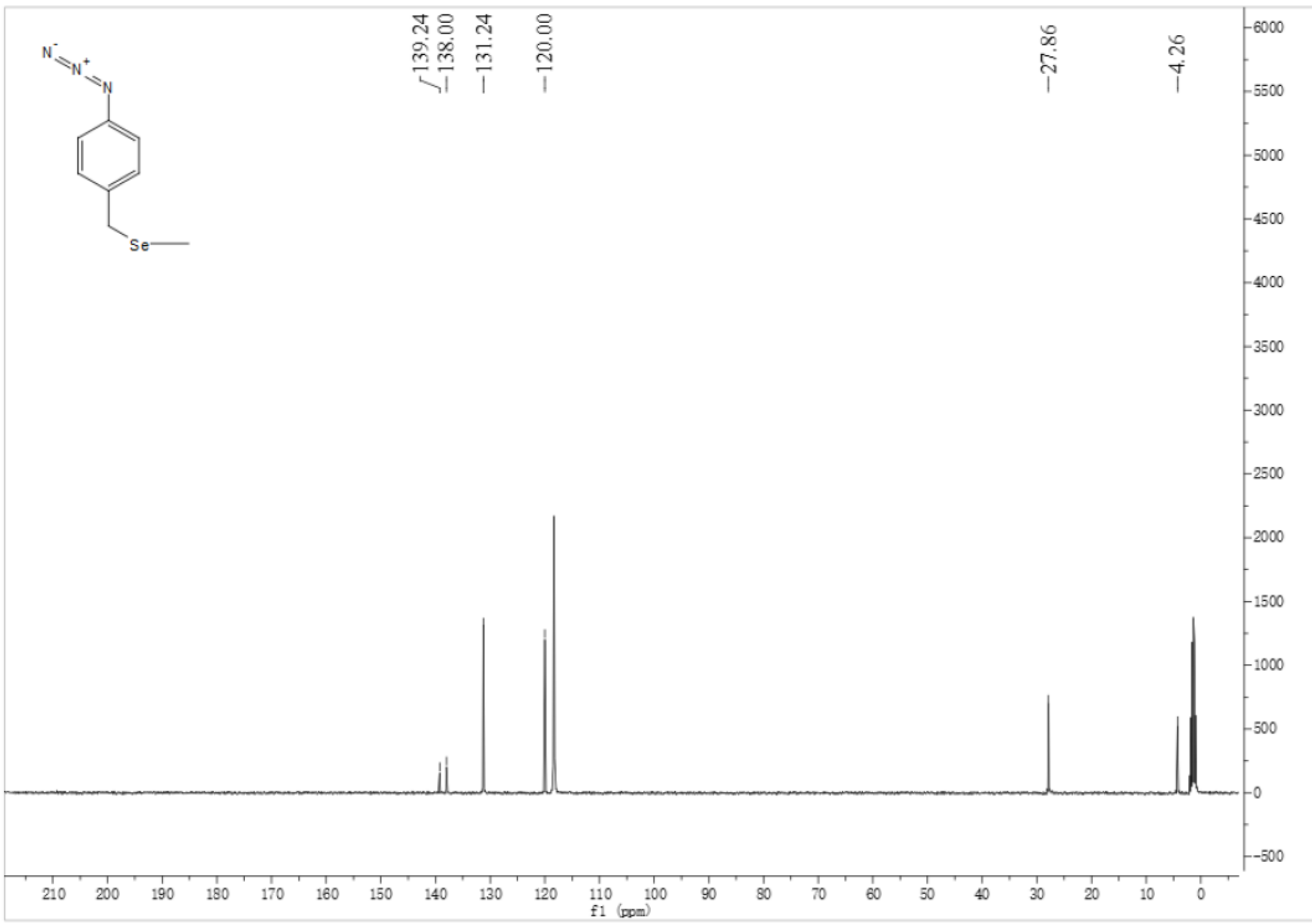

^{13}C{^{1}H} NMR (101 MHz, acetonitrile-d_3) spectrum of compound **1**.

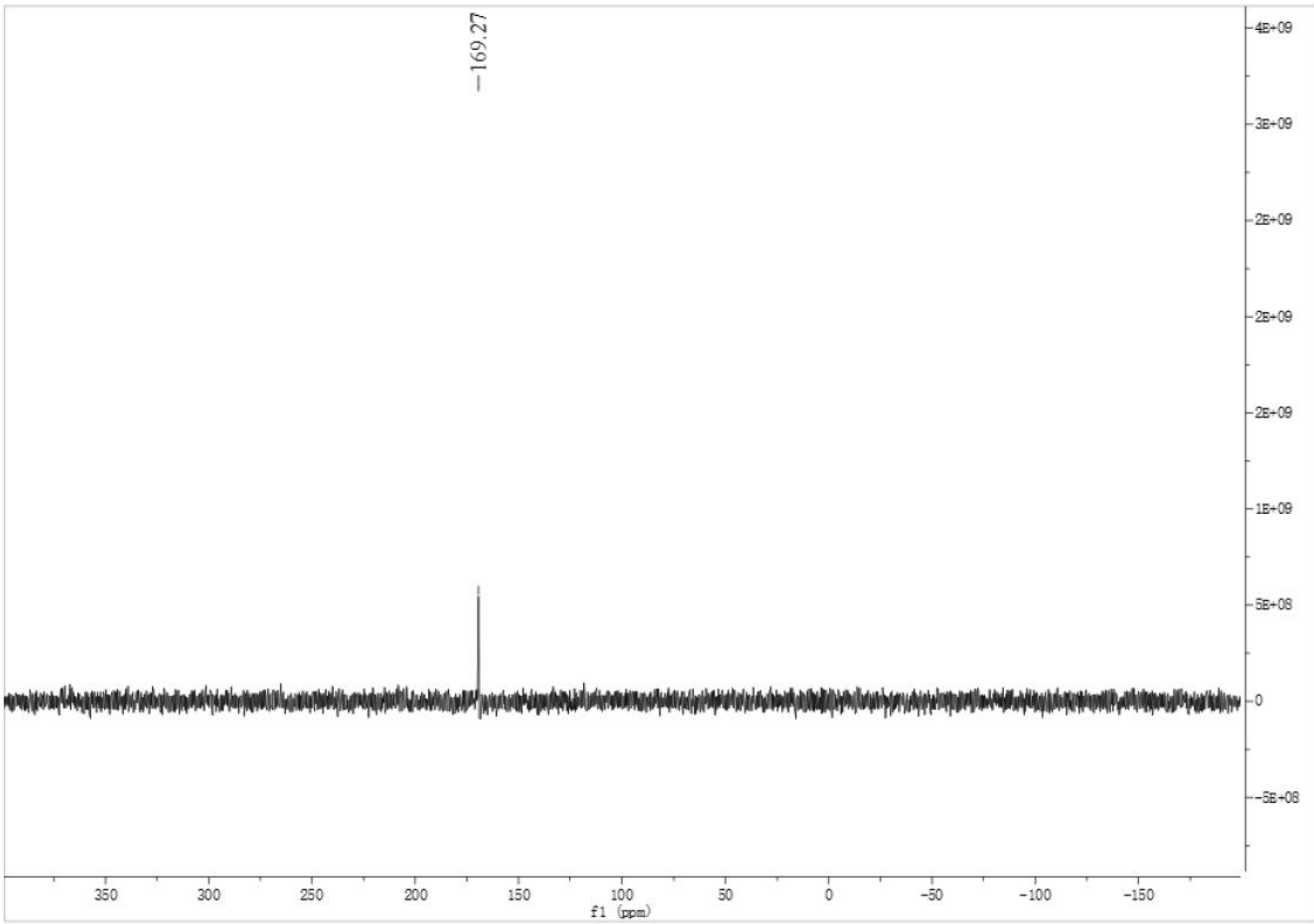

$^{77}Se\{^{1}H\}$ NMR (114 MHz, water-d_2) spectrum of compound **1**

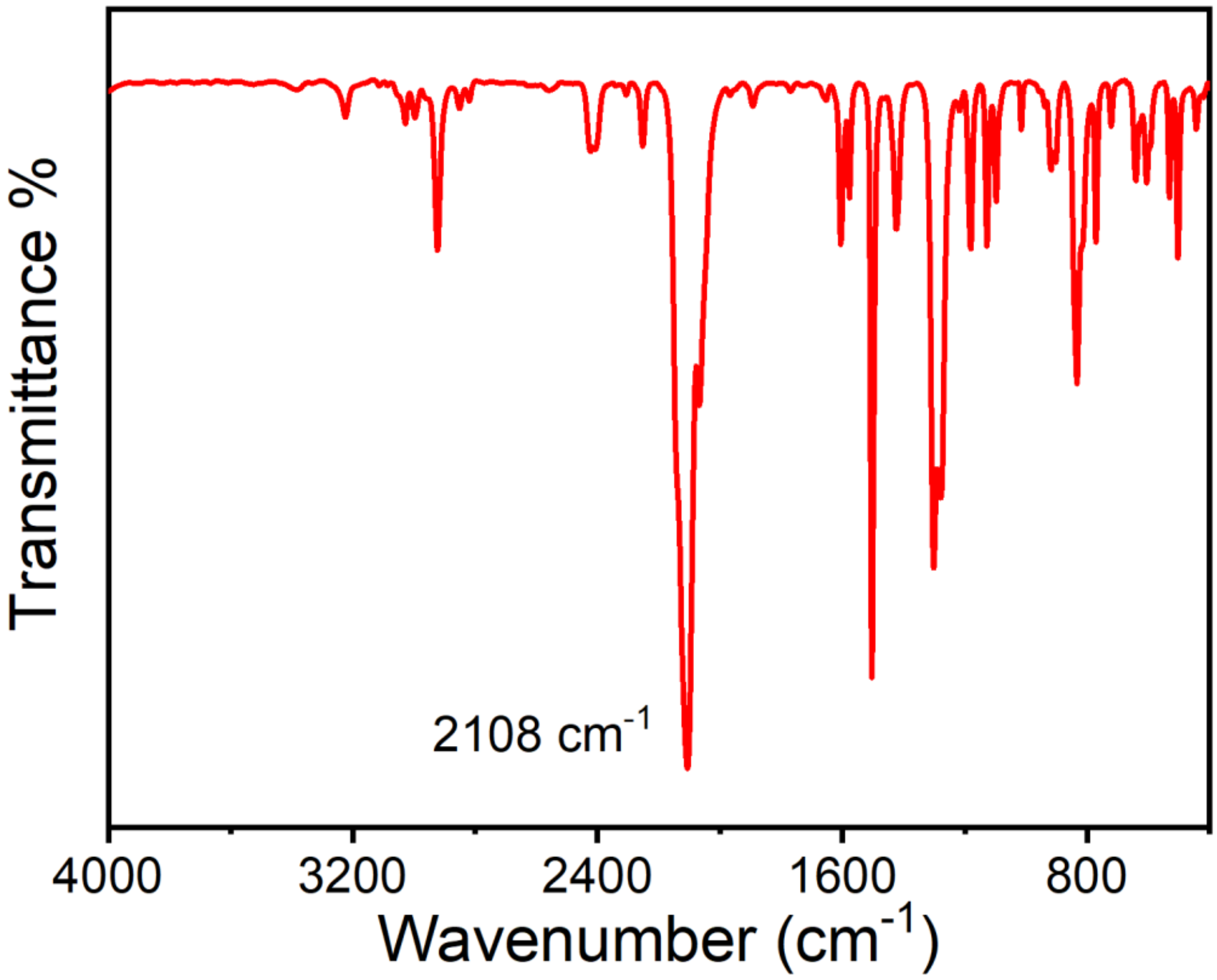

FT-IR spectrum of compound **1**.

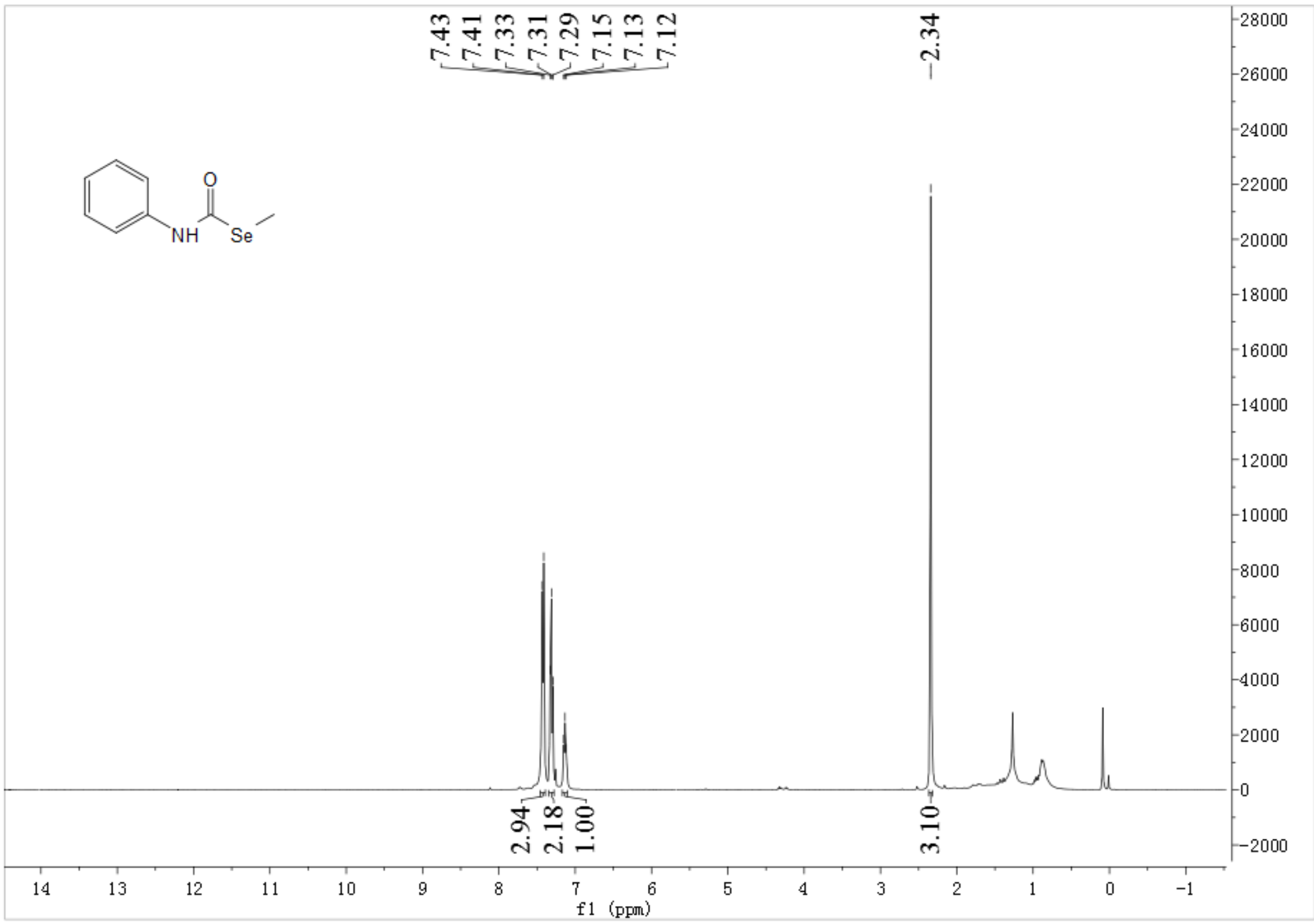

^{1}H NMR (400 MHz, chloroform-*d*) spectrum of compound **2**.

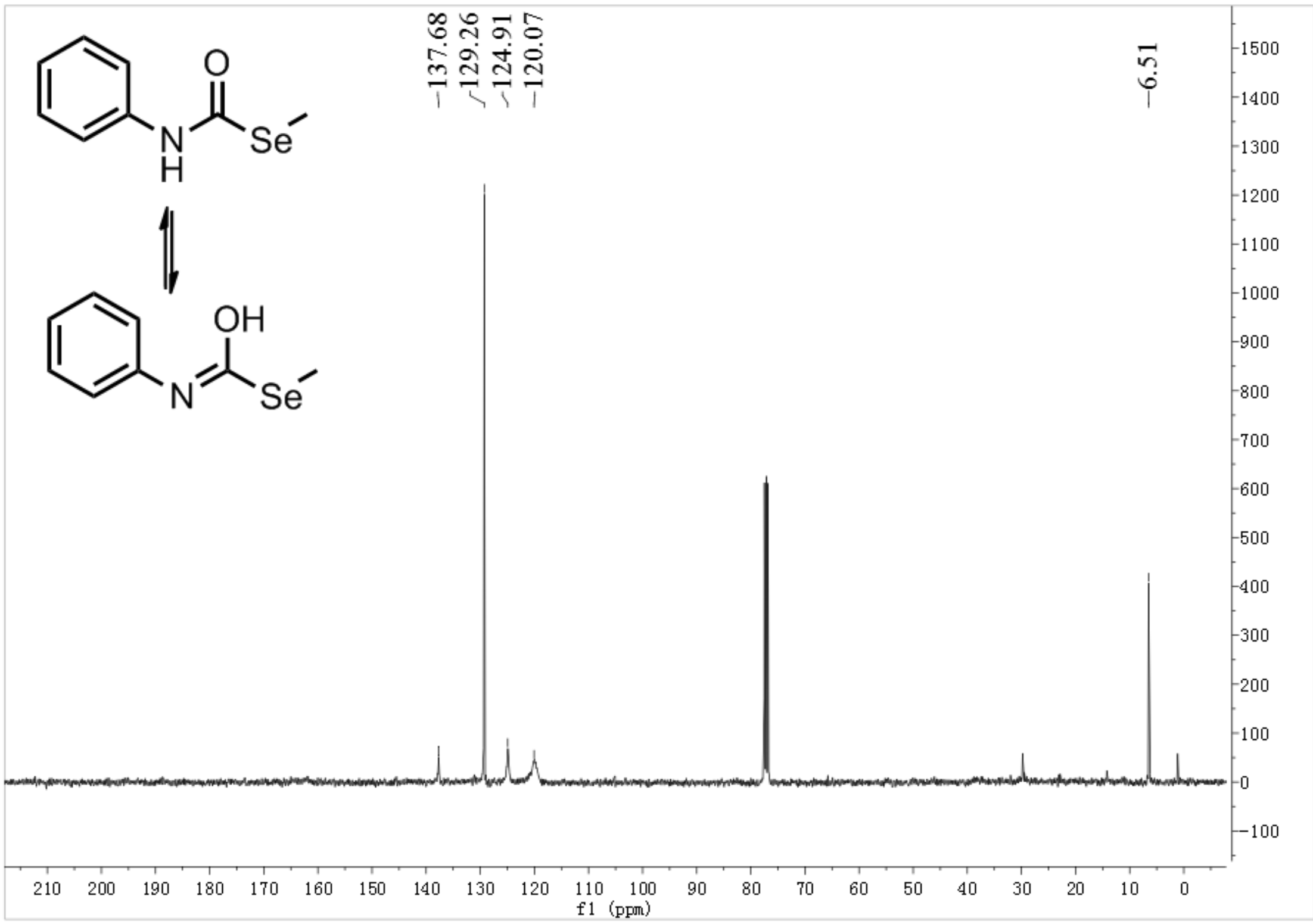

^{13}C{^{1}H} NMR (101 MHz, chloroform-*d*) spectrum of compound **2**.

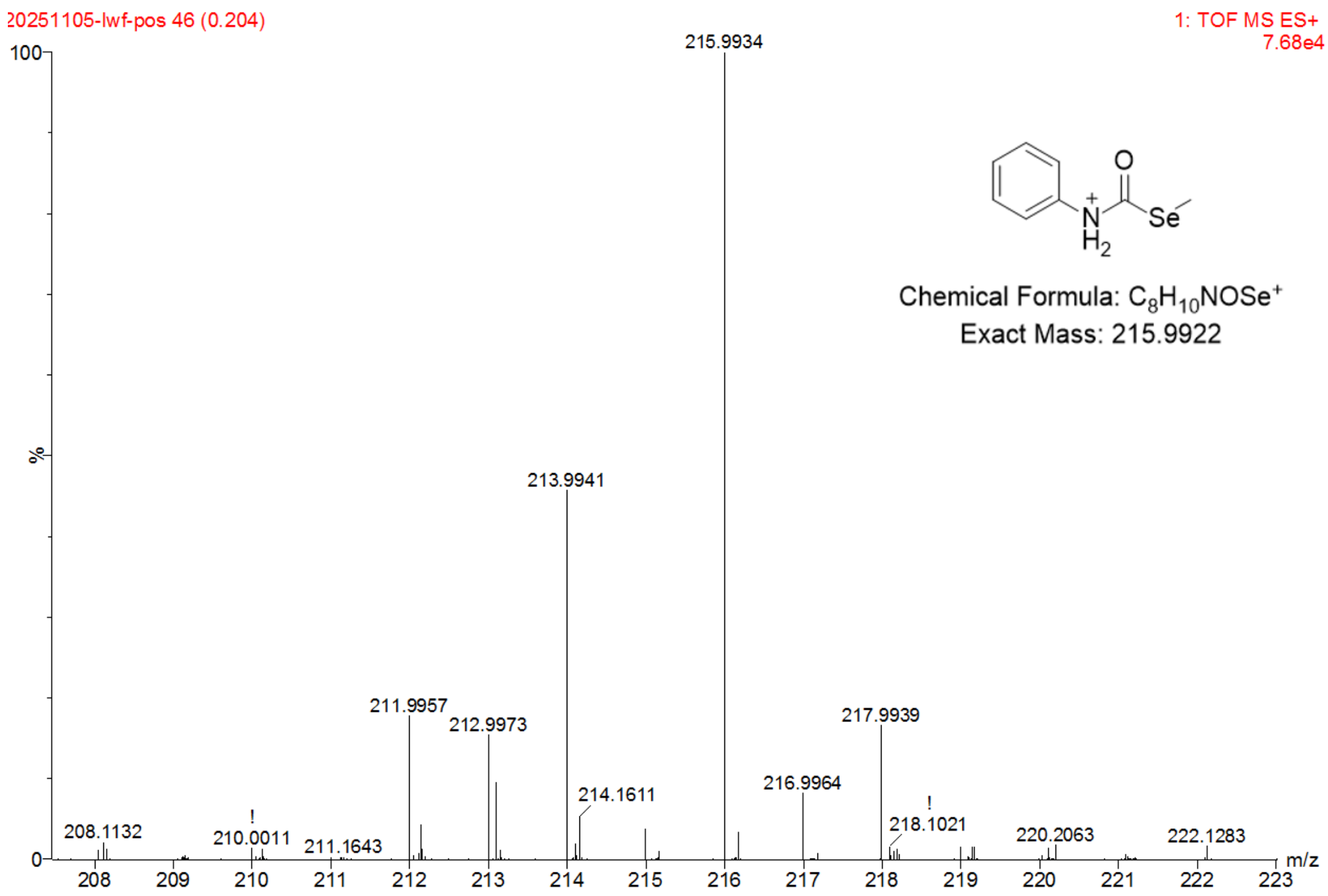

HRMS (ESI) of compound **2**.

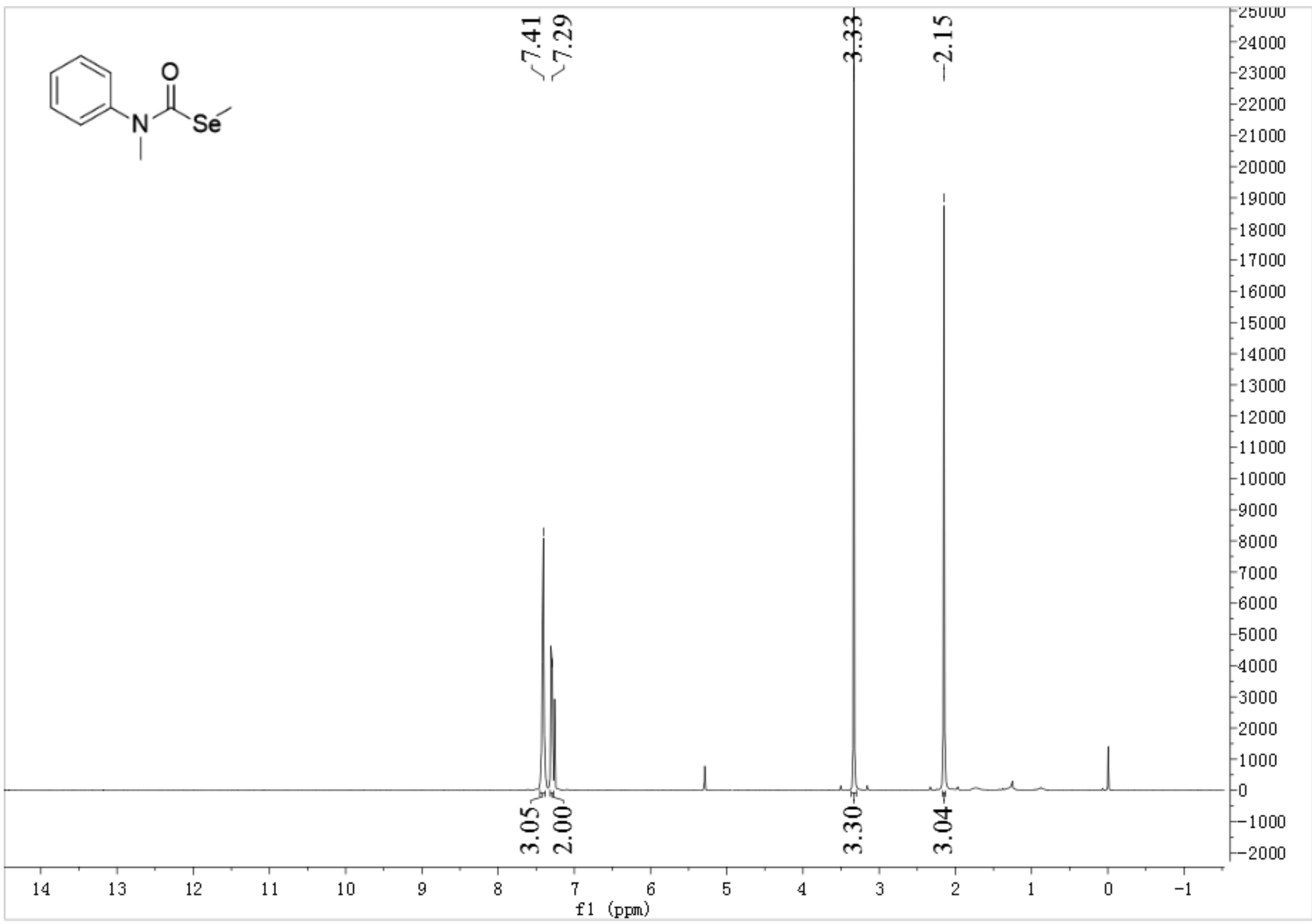

^{1}H NMR (400 MHz, chloroform-*d*) spectrum of compound **3**.

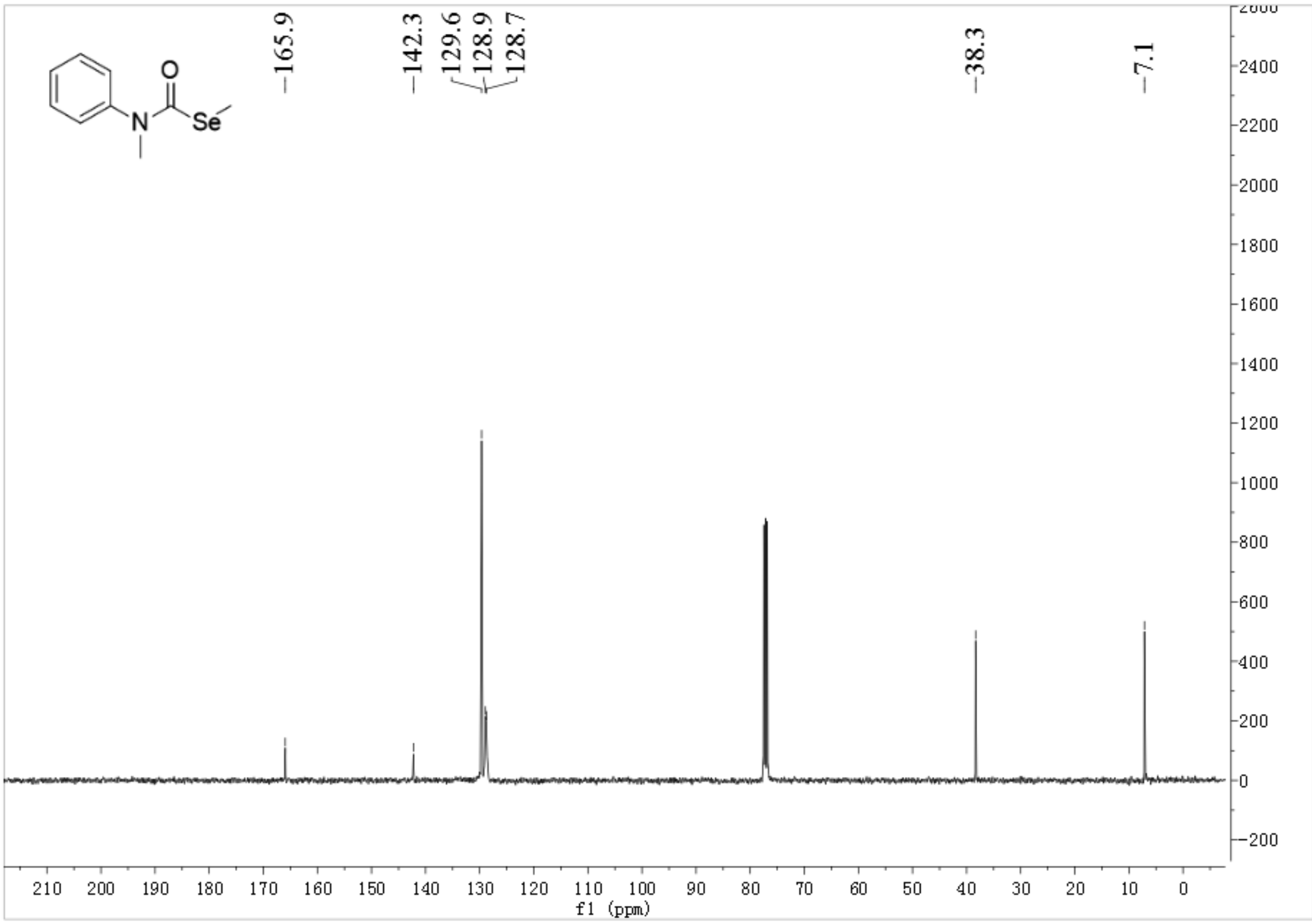

$^{13}C\{^1H\}$ NMR (101 MHz, chloroform-*d*) spectrum of compound **3**.

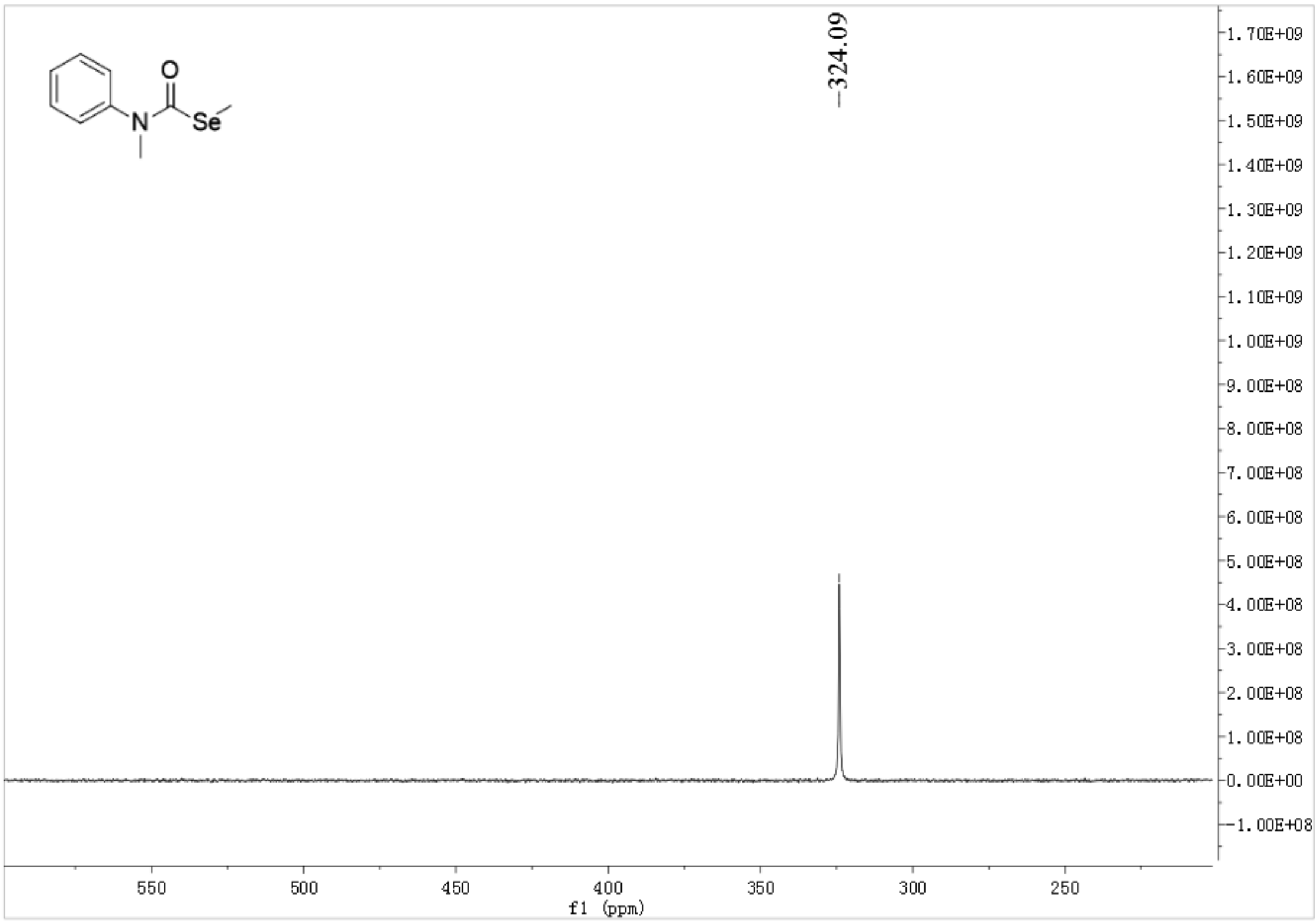

$^{77}Se\{^1H\}$ NMR (114 MHz, chloroform-*d*) spectrum of compound **3**.

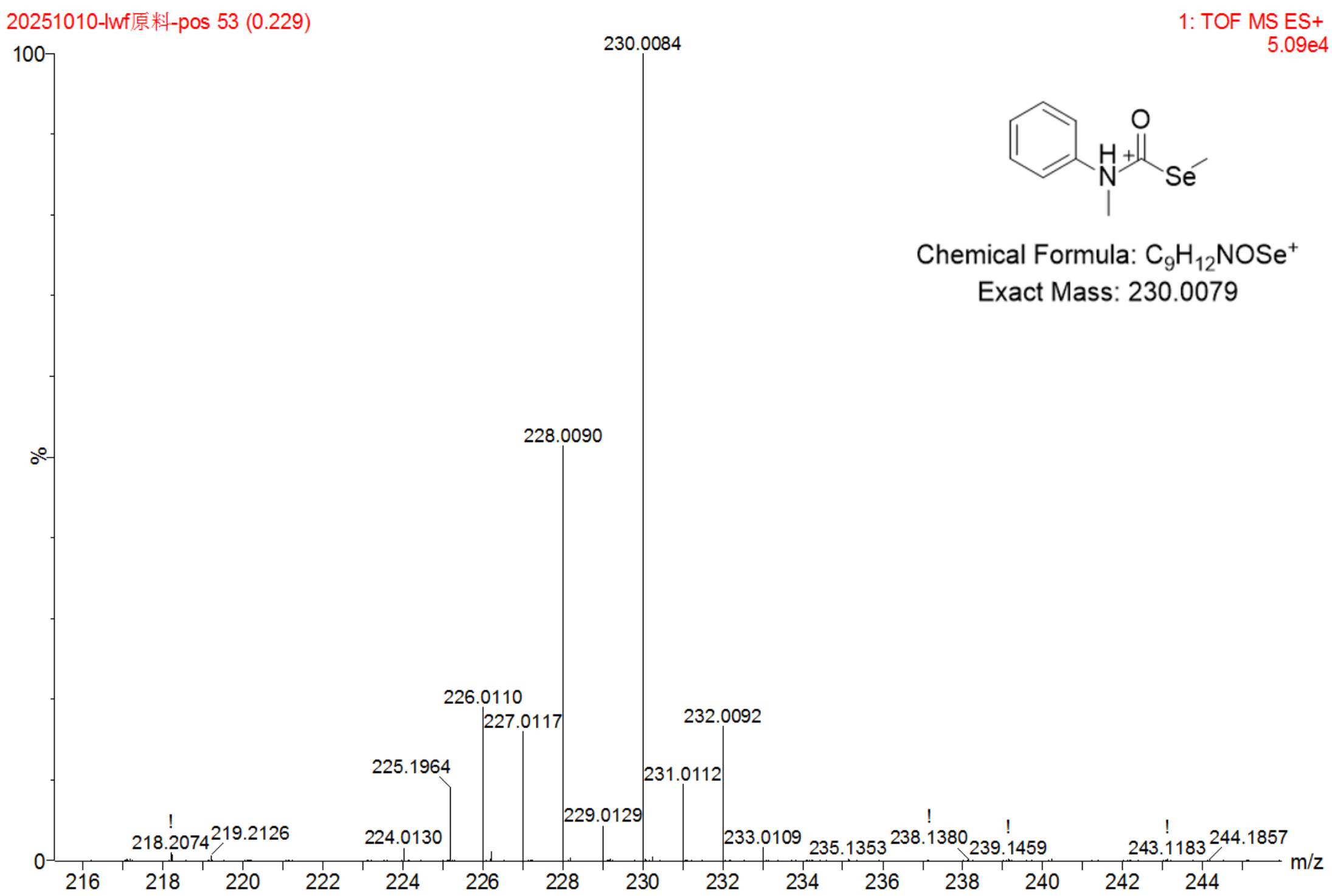

HRMS (ESI) of compound **3**.

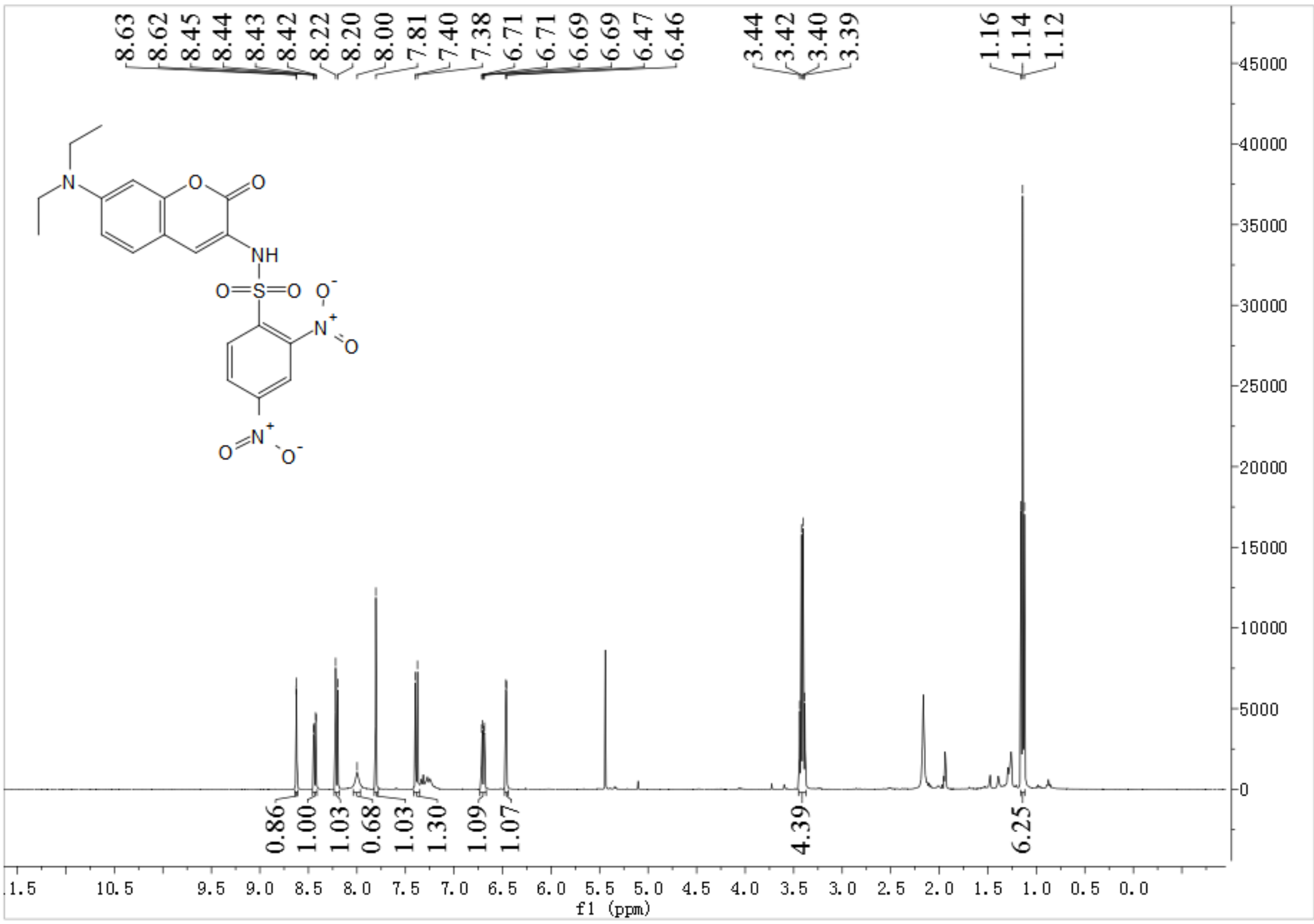

^{1}H NMR (400 MHz, acetonitrile-d_3) spectrum of probe **Sel-green**.

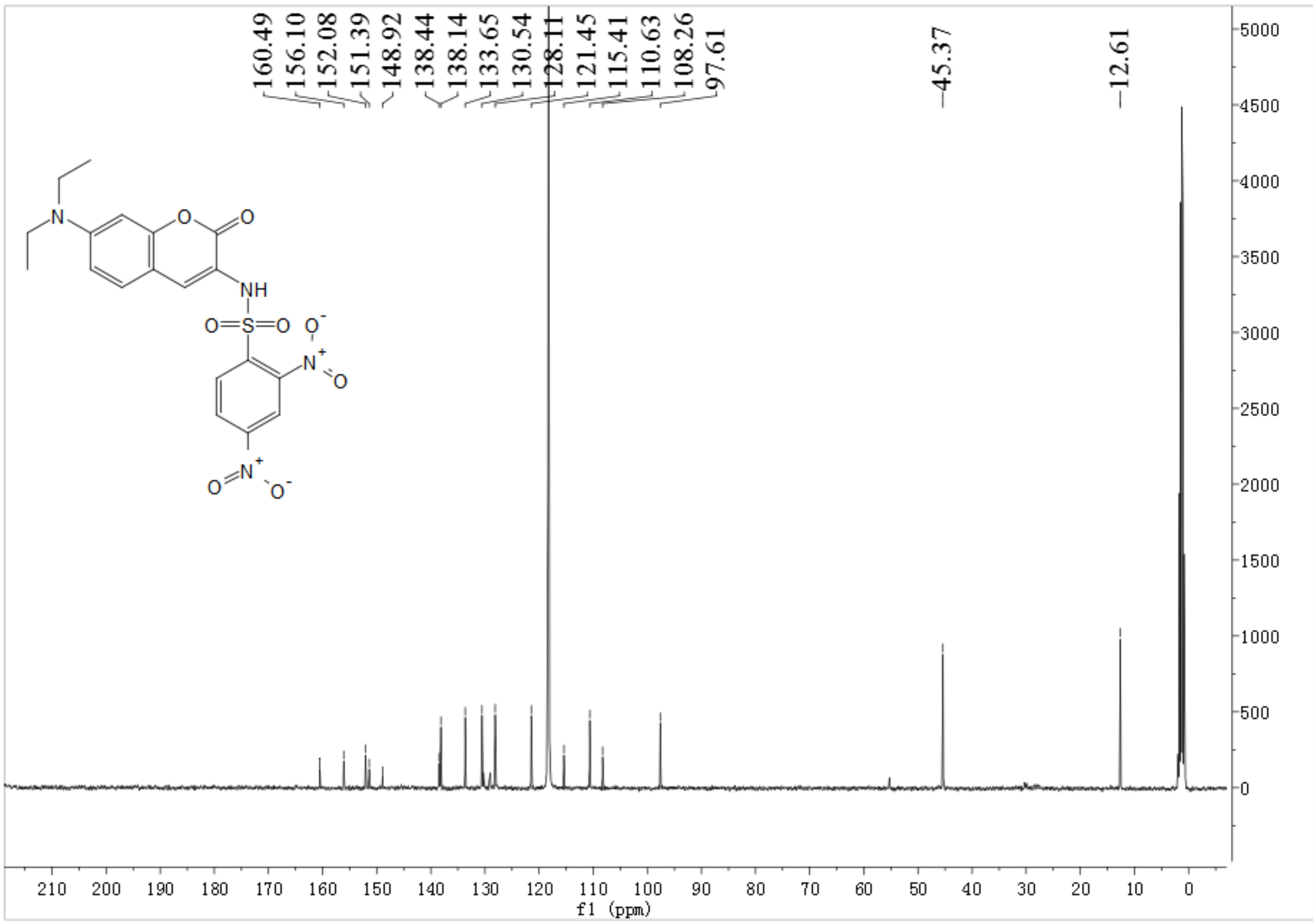

$^{13}C\{^{1}H\}$ NMR (101 MHz, acetonitrile-d_3) spectrum of probe **Sel-green**.

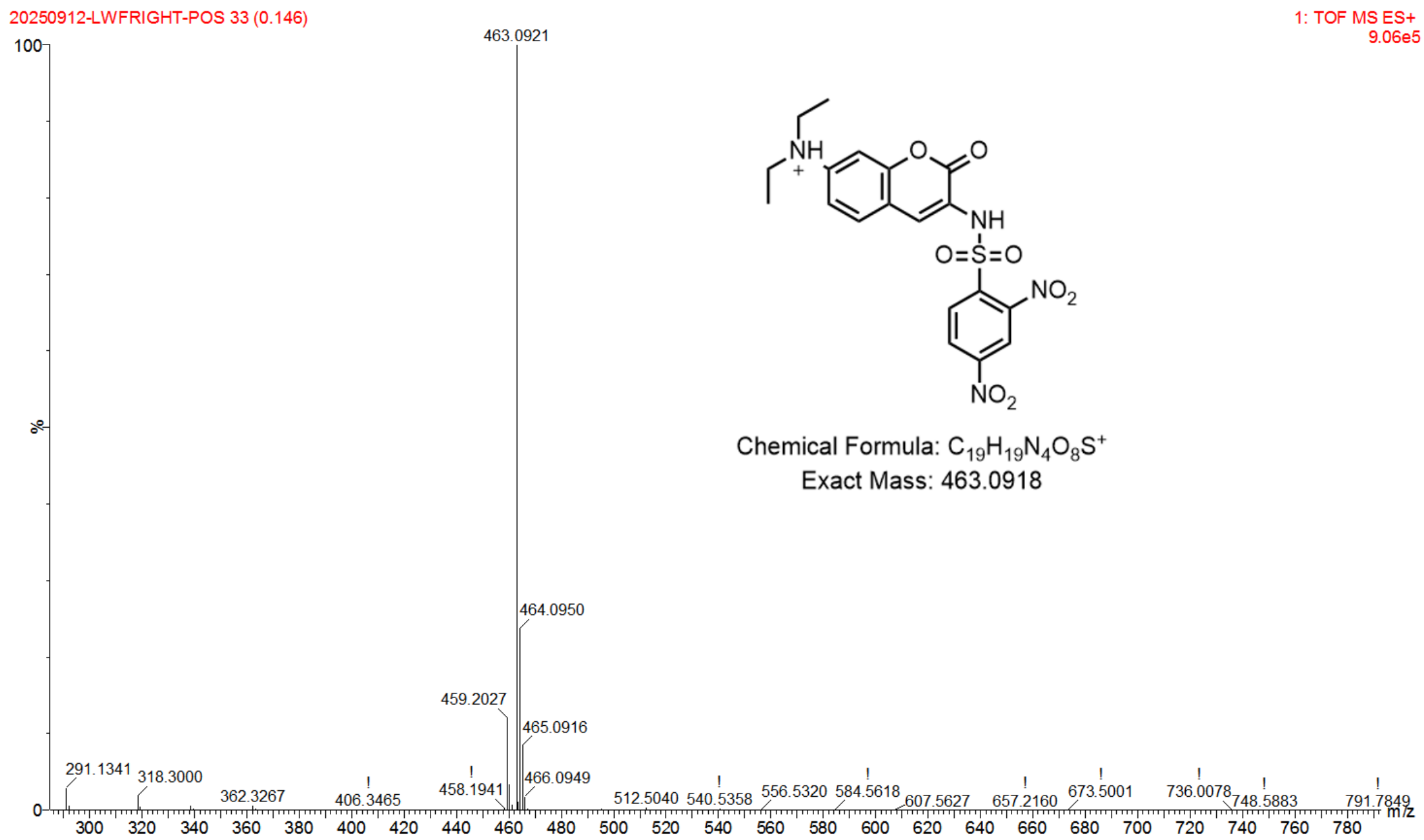

HRMS (ESI) of probe **Sel-green**.

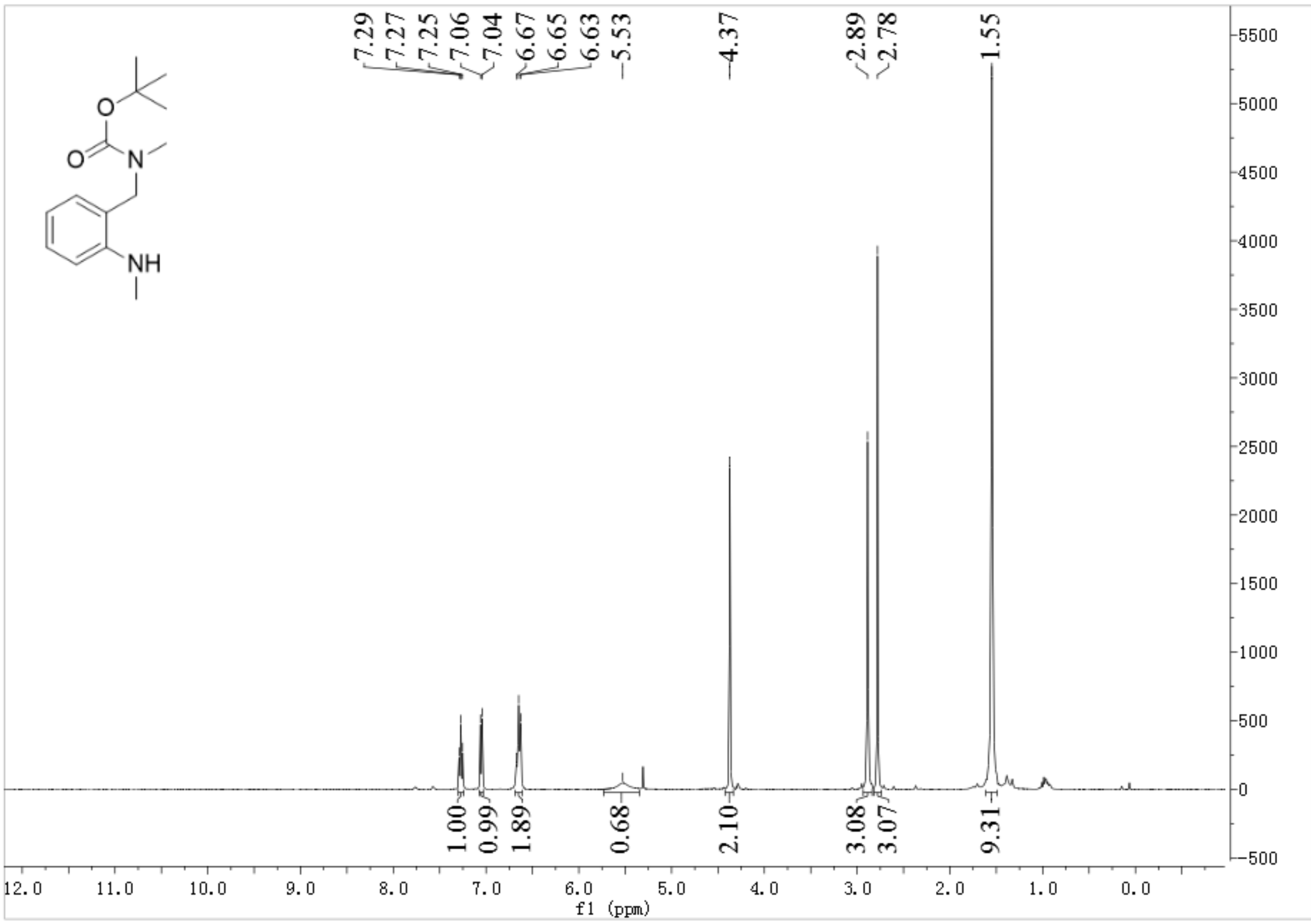

^{1}H NMR (400 MHz, chloroform-*d*) spectrum of compound **4a**.

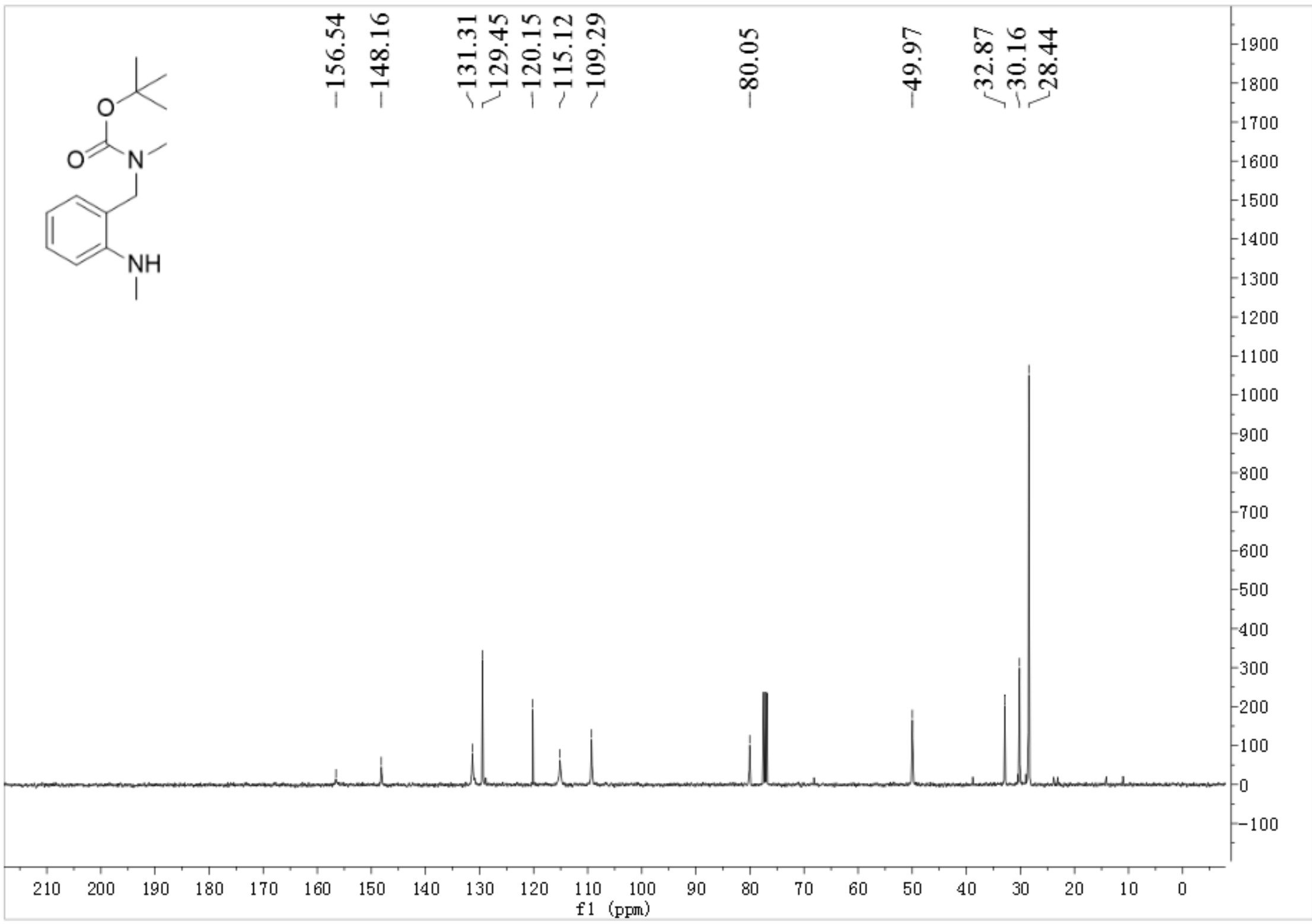

$^{13}C\{^{1}H\}$ NMR (101 MHz, chloroform-*d*) spectrum of compound **4a**.

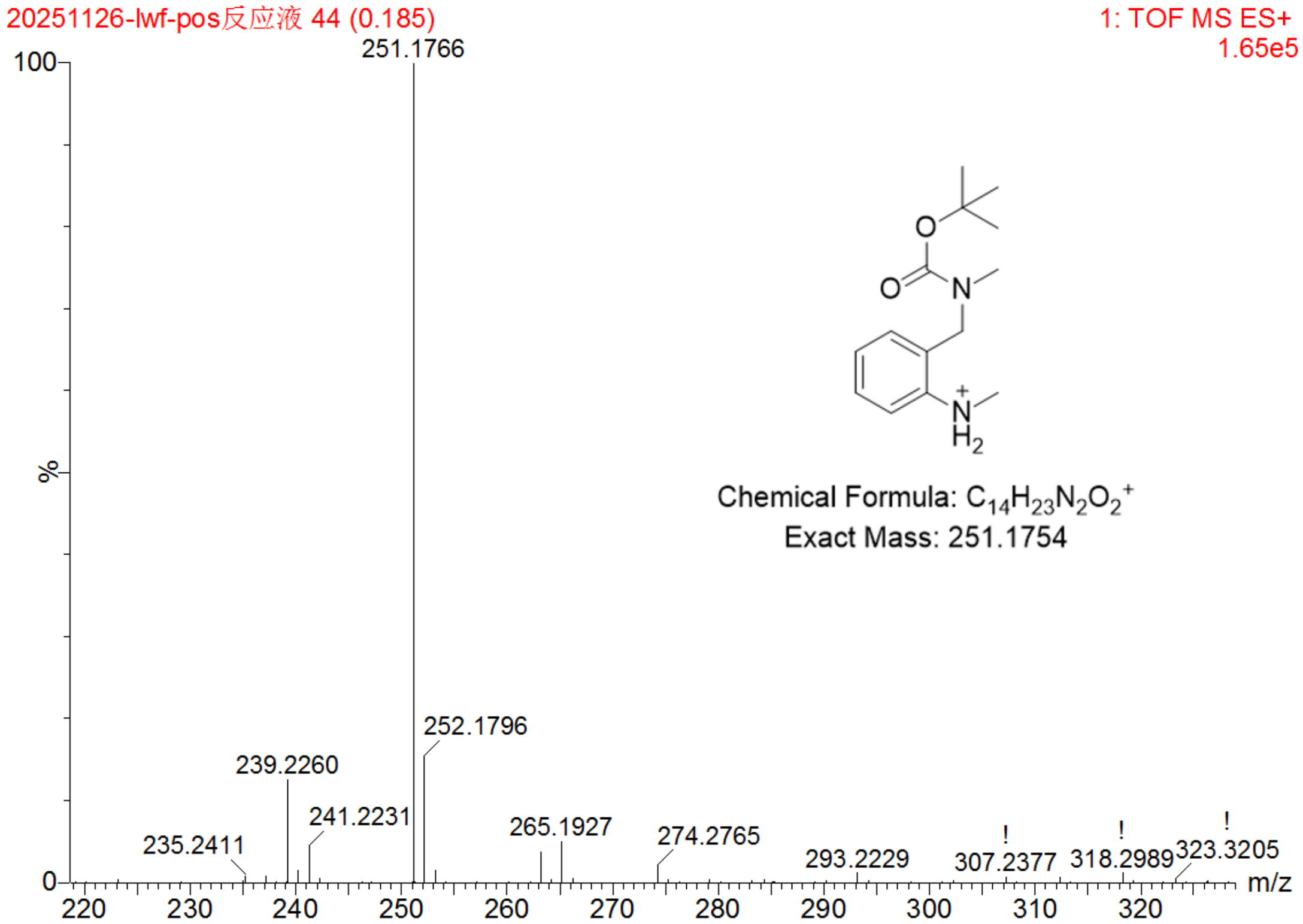

HRMS (ESI) of compound **4a**.

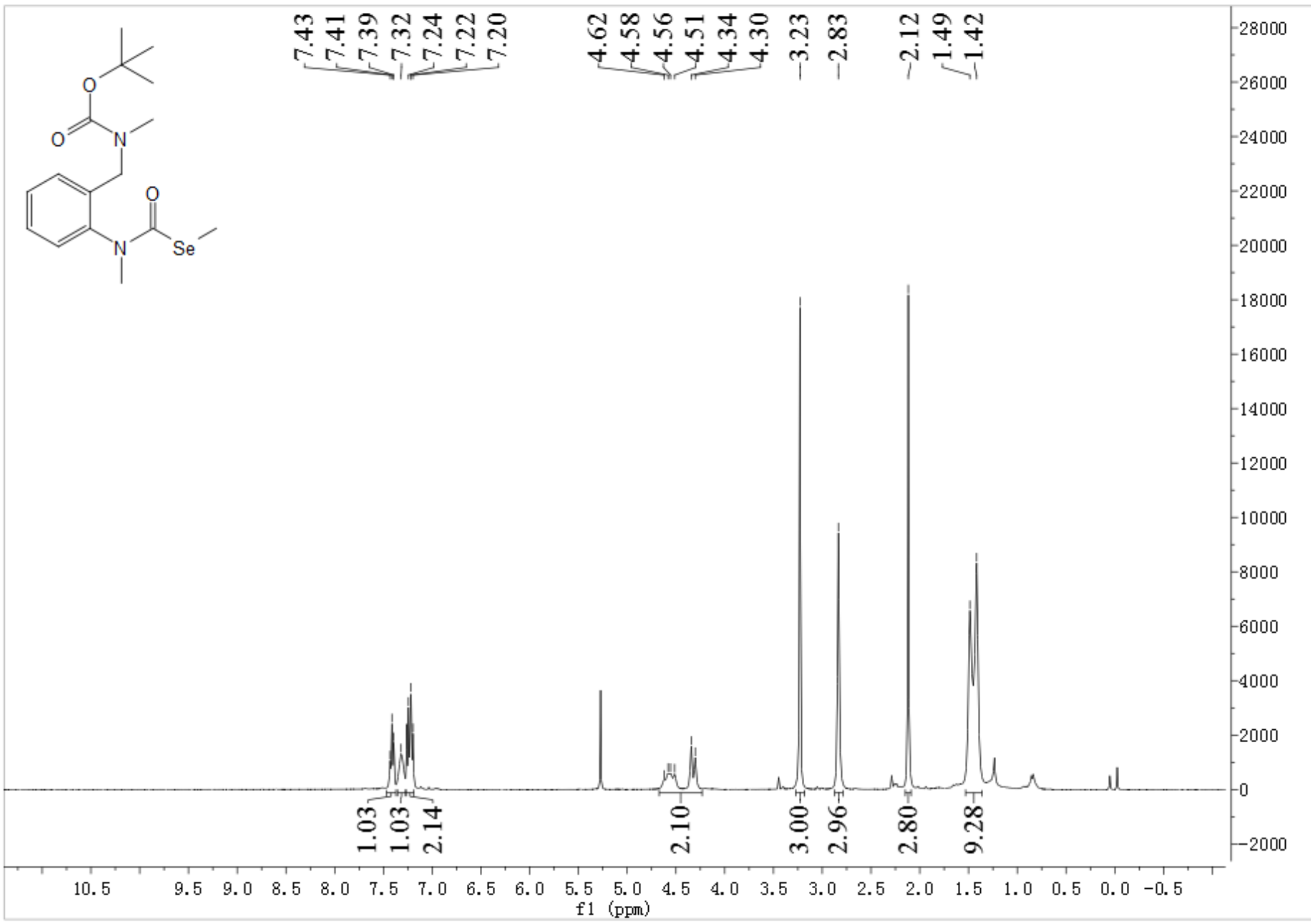

^{1}H NMR (400 MHz, chloroform-*d*) spectrum of compound **4**.

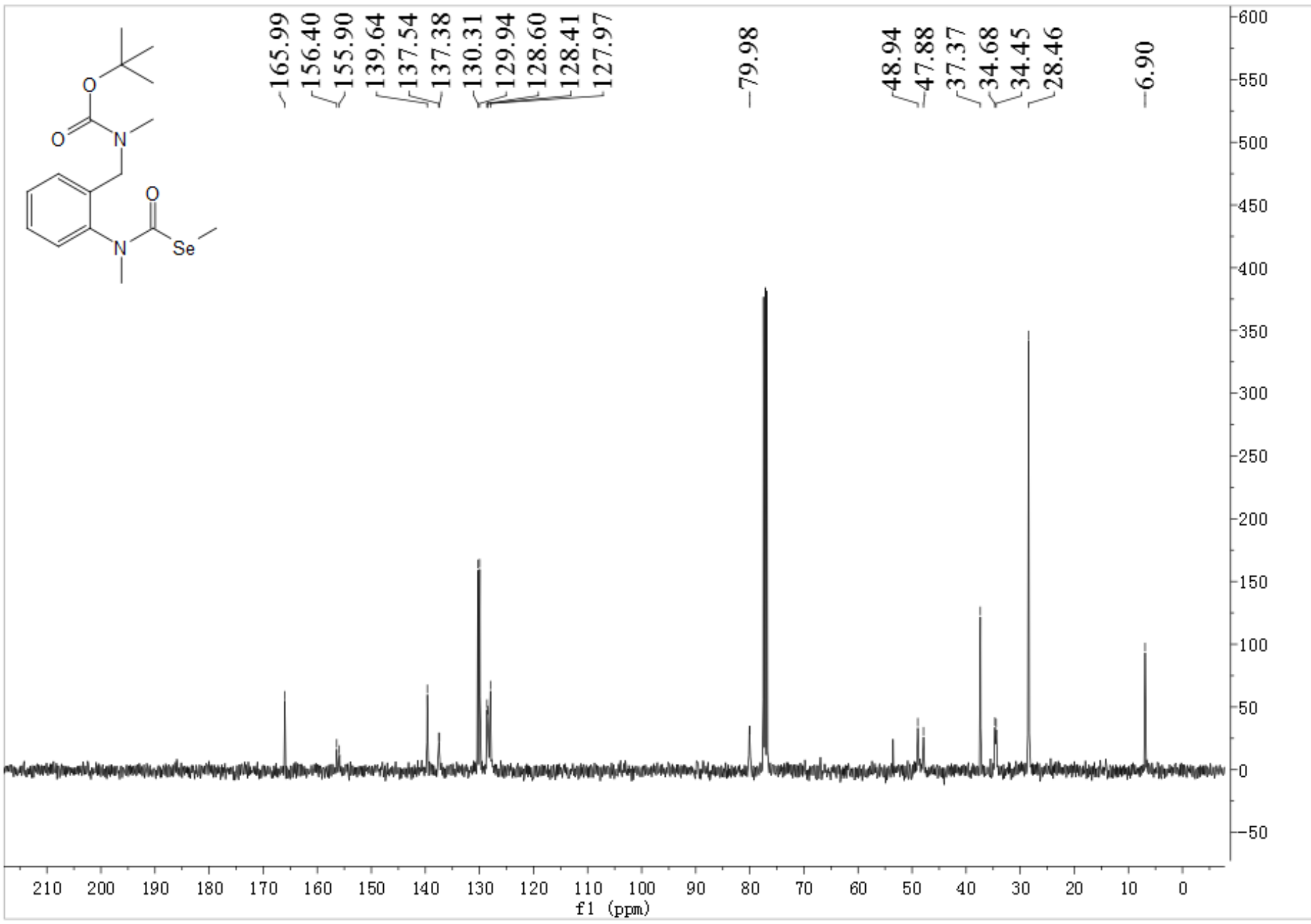

$^{13}C\{^1H\}$ NMR (101 MHz, chloroform-*d*) spectrum of compound **4**.

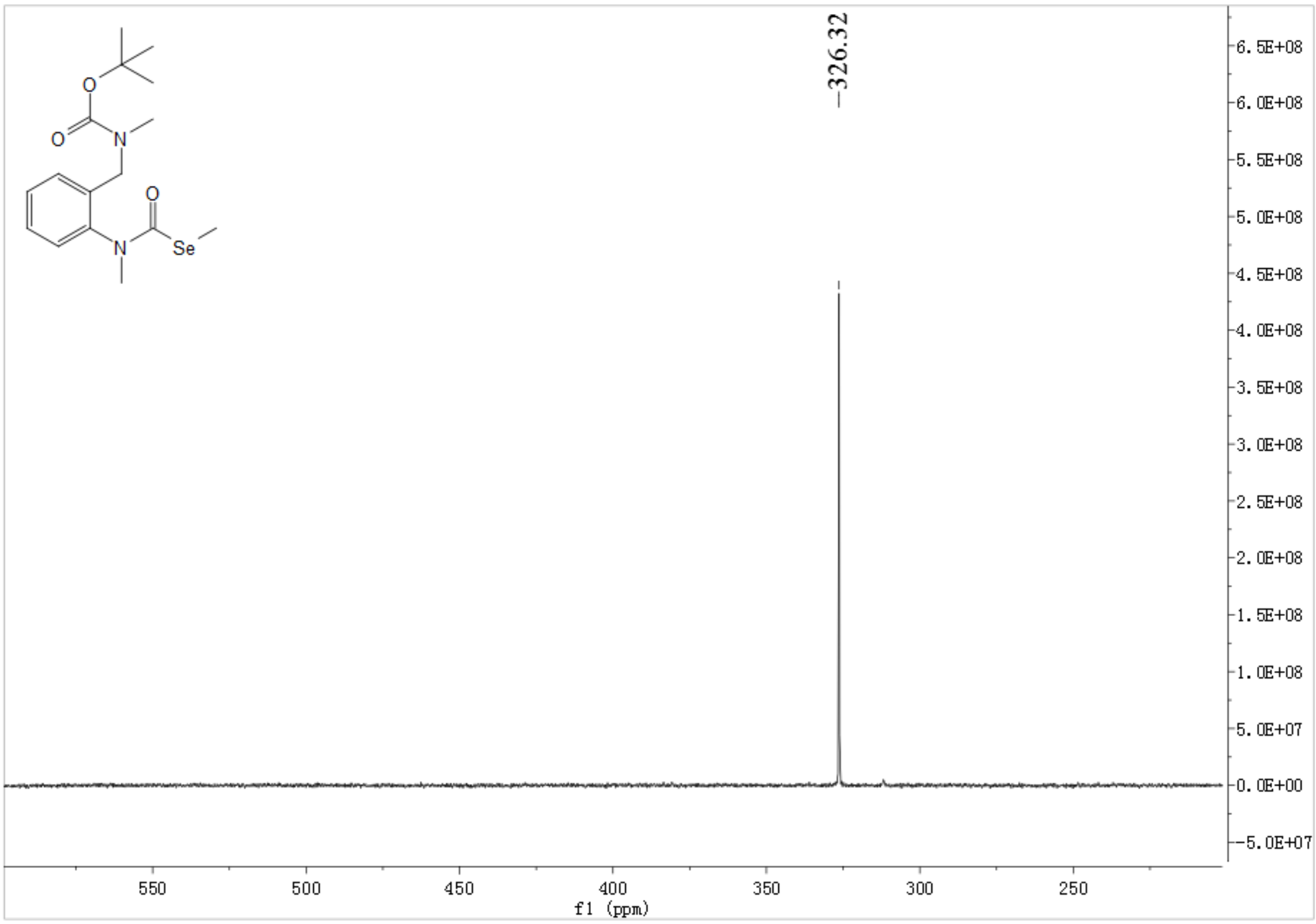

$^{77}Se\{^1H\}$ NMR (114 MHz, chloroform-*d*) spectrum of compound **4**.

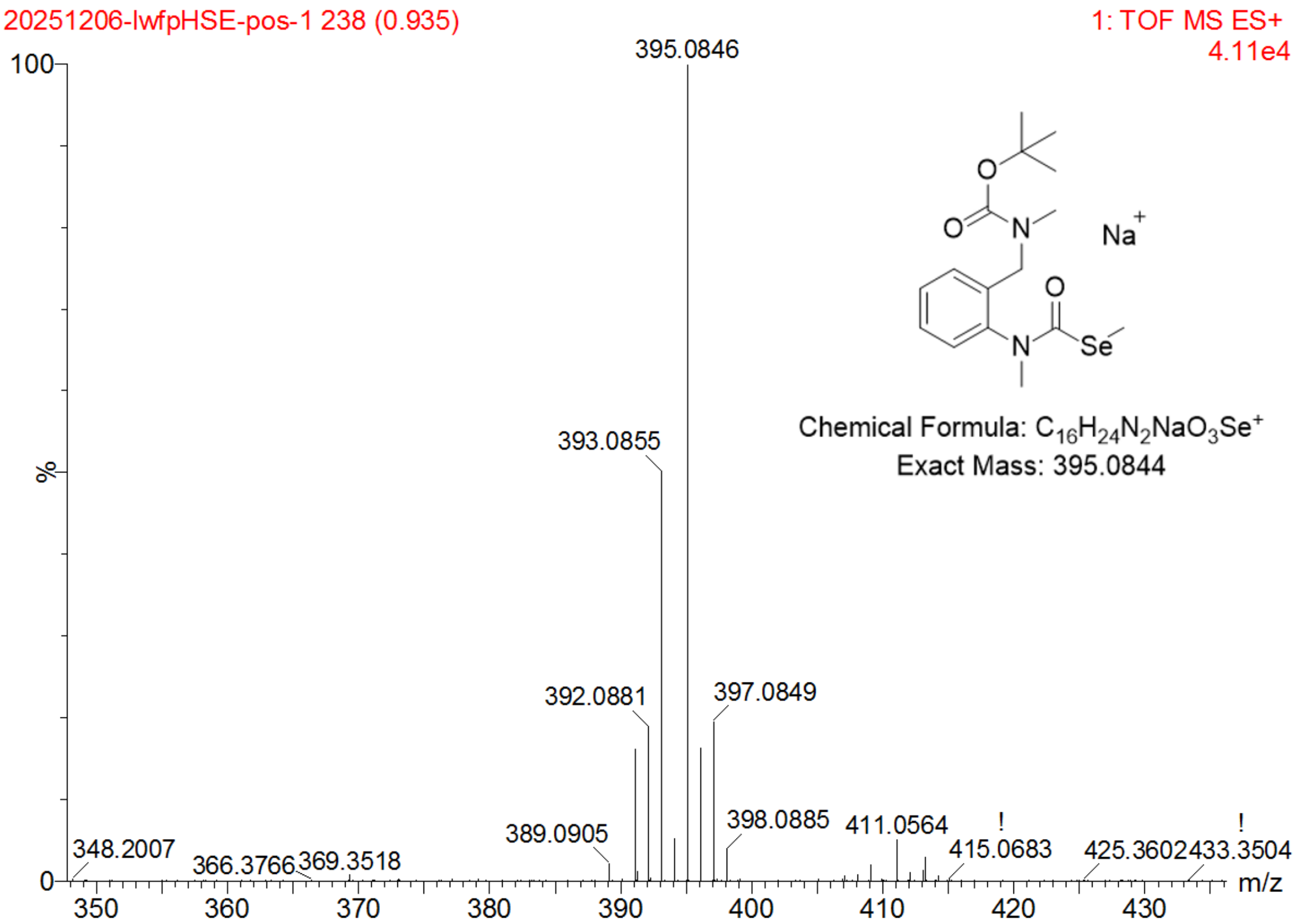

HRMS (ESI) of compound **4**.

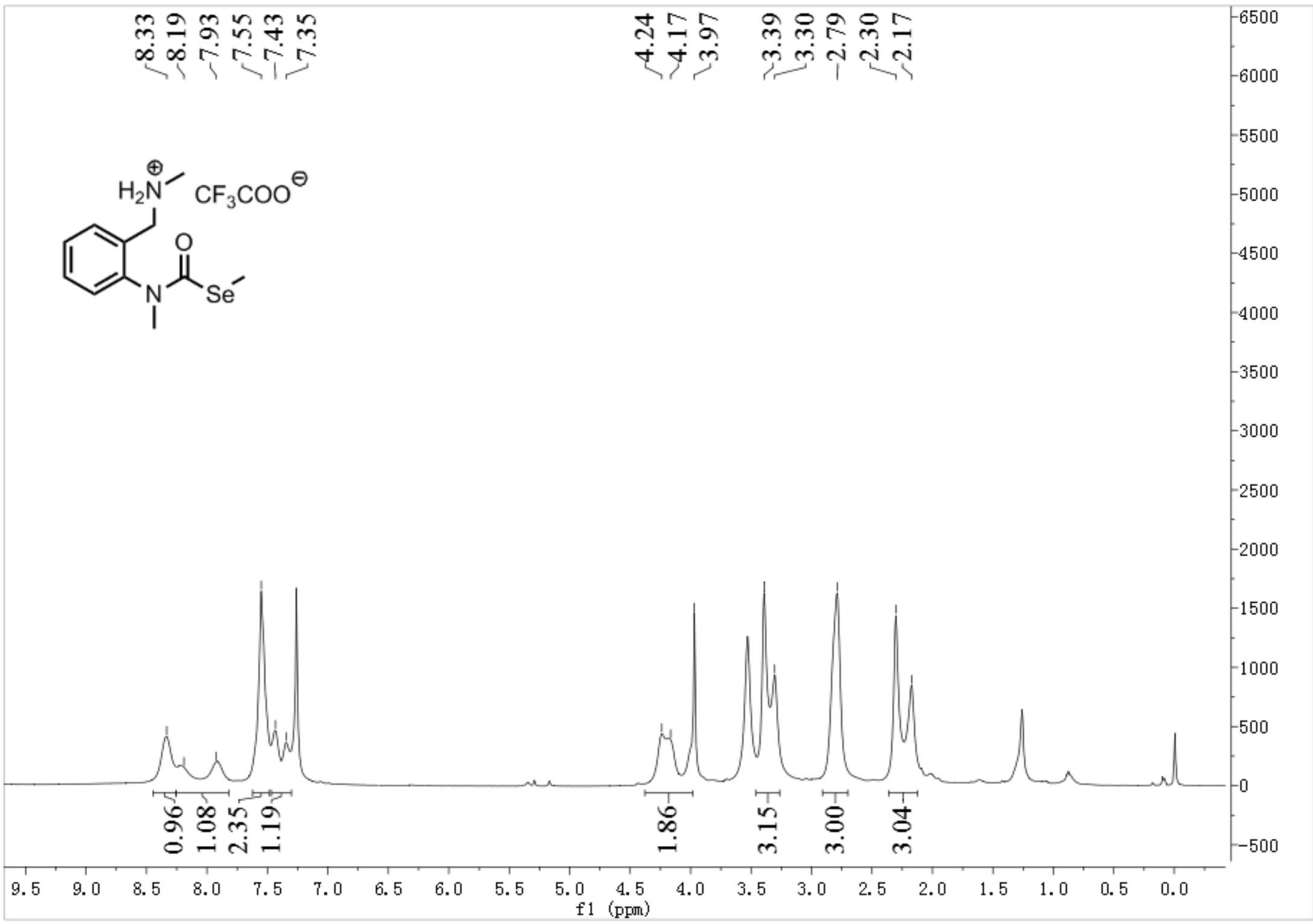

^{1}H NMR (400 MHz, chloroform-*d*) spectrum of compound **TD-MSe1**.

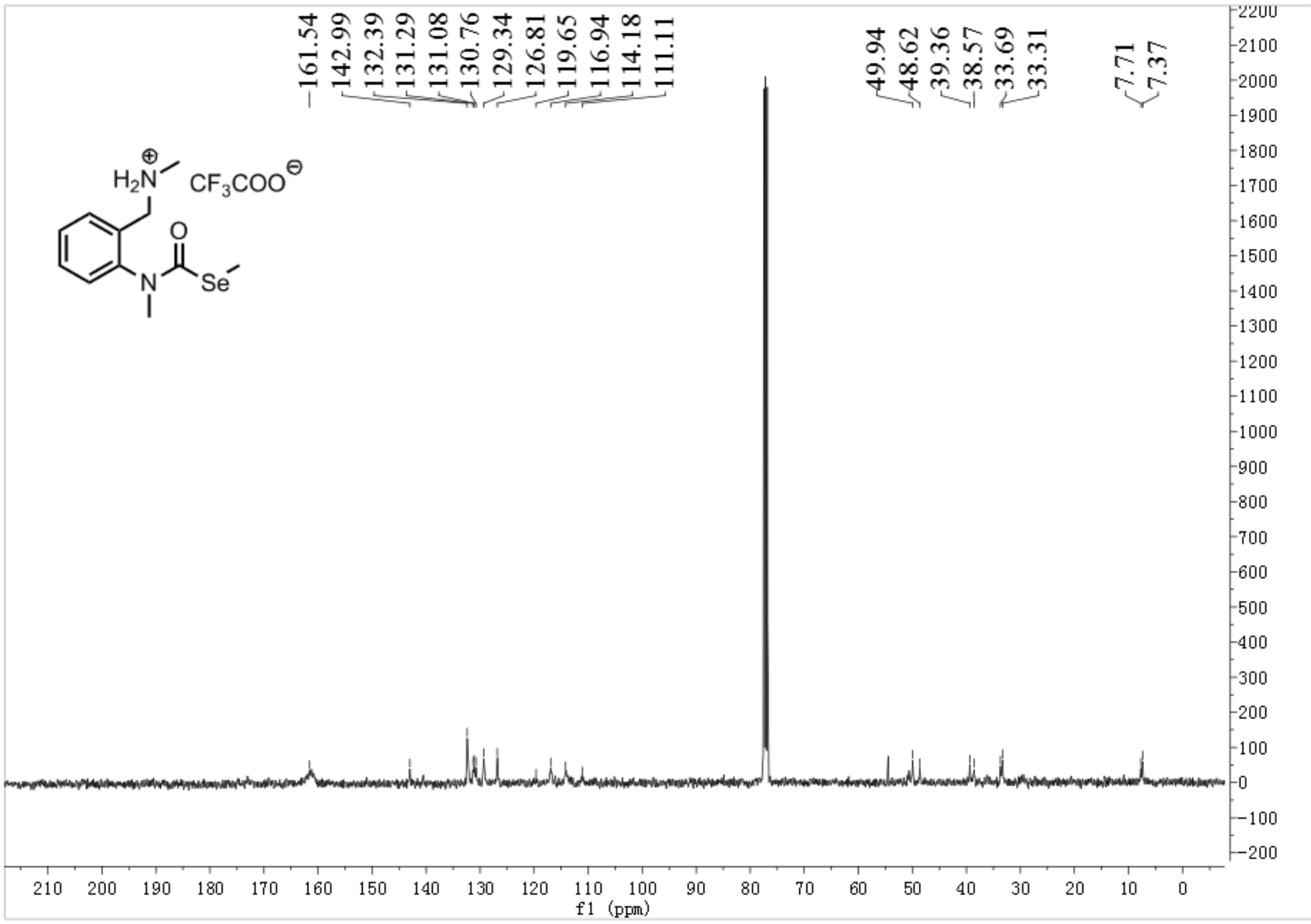

$^{13}C\{^1H\}$ NMR (101 MHz, chloroform-*d*) spectrum of compound **TD-MSe1**.

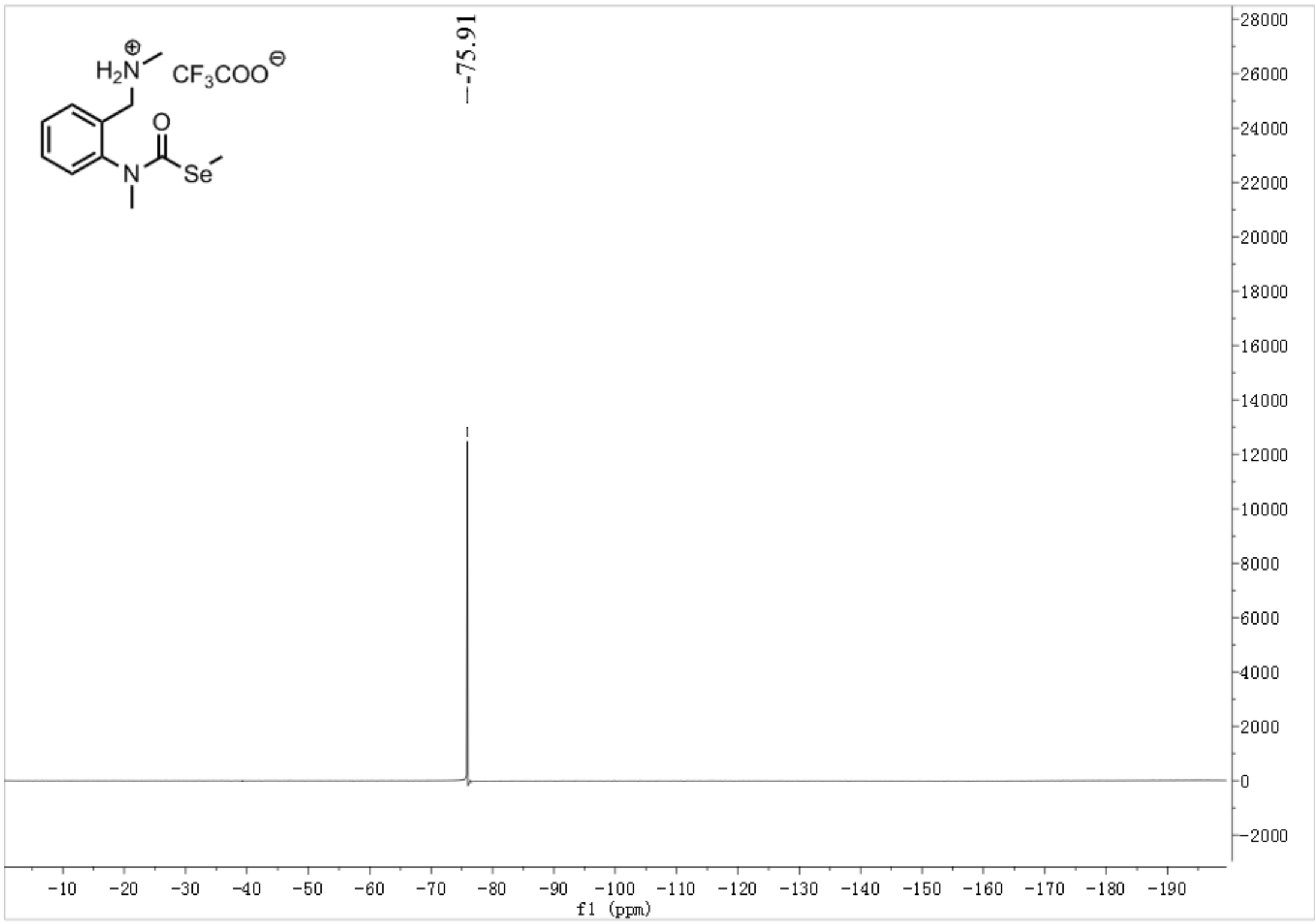

$^{19}F\{^1H\}$ NMR (376 MHz, chloroform-*d*) spectrum of compound **TD-MSe1**.

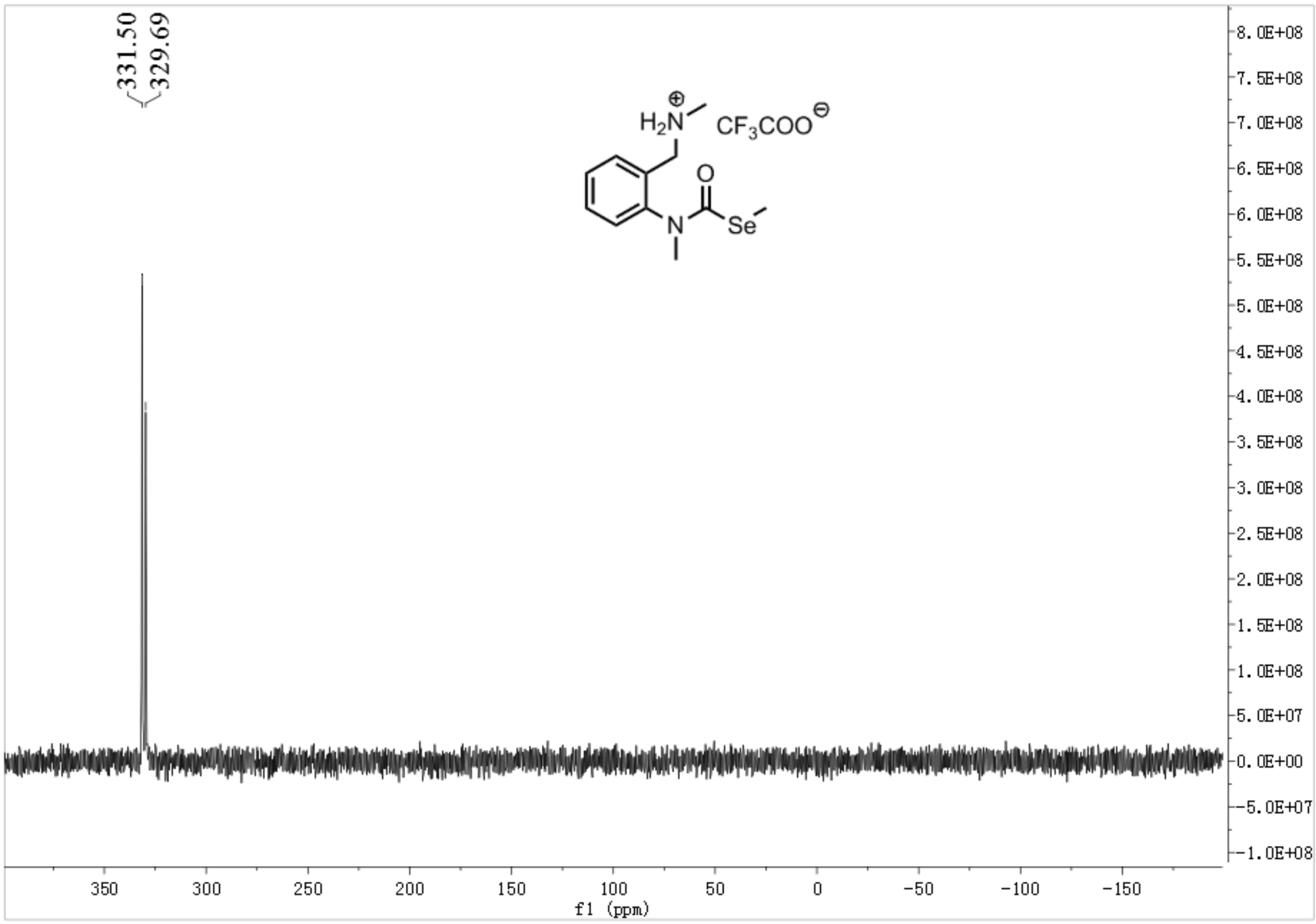

$^{77}Se\{^{1}H\}$ NMR (114 MHz, chloroform-*d*) spectrum of compound **TD-MSe1**.

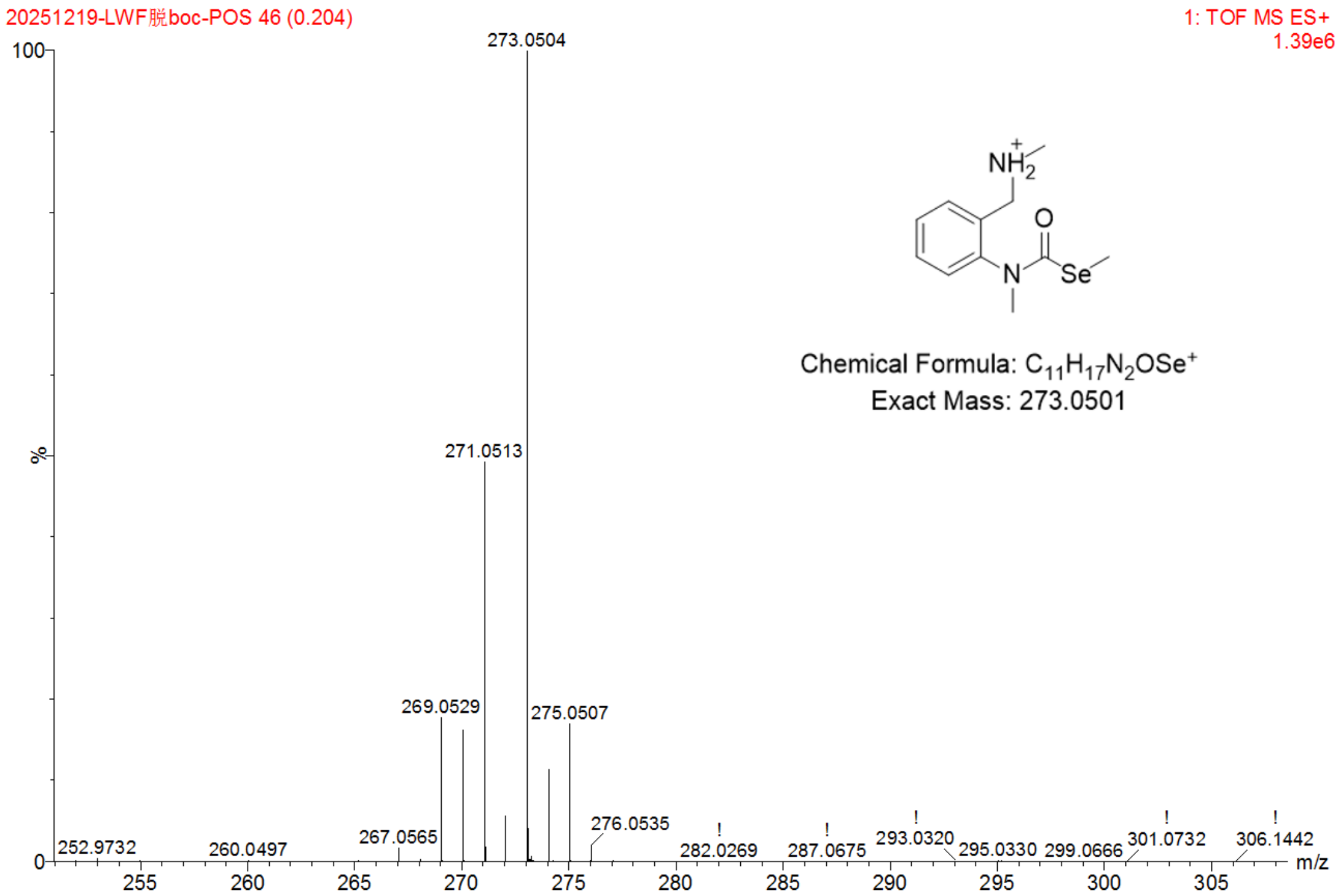

HRMS (ESI) of compound **TD-MSe1**.

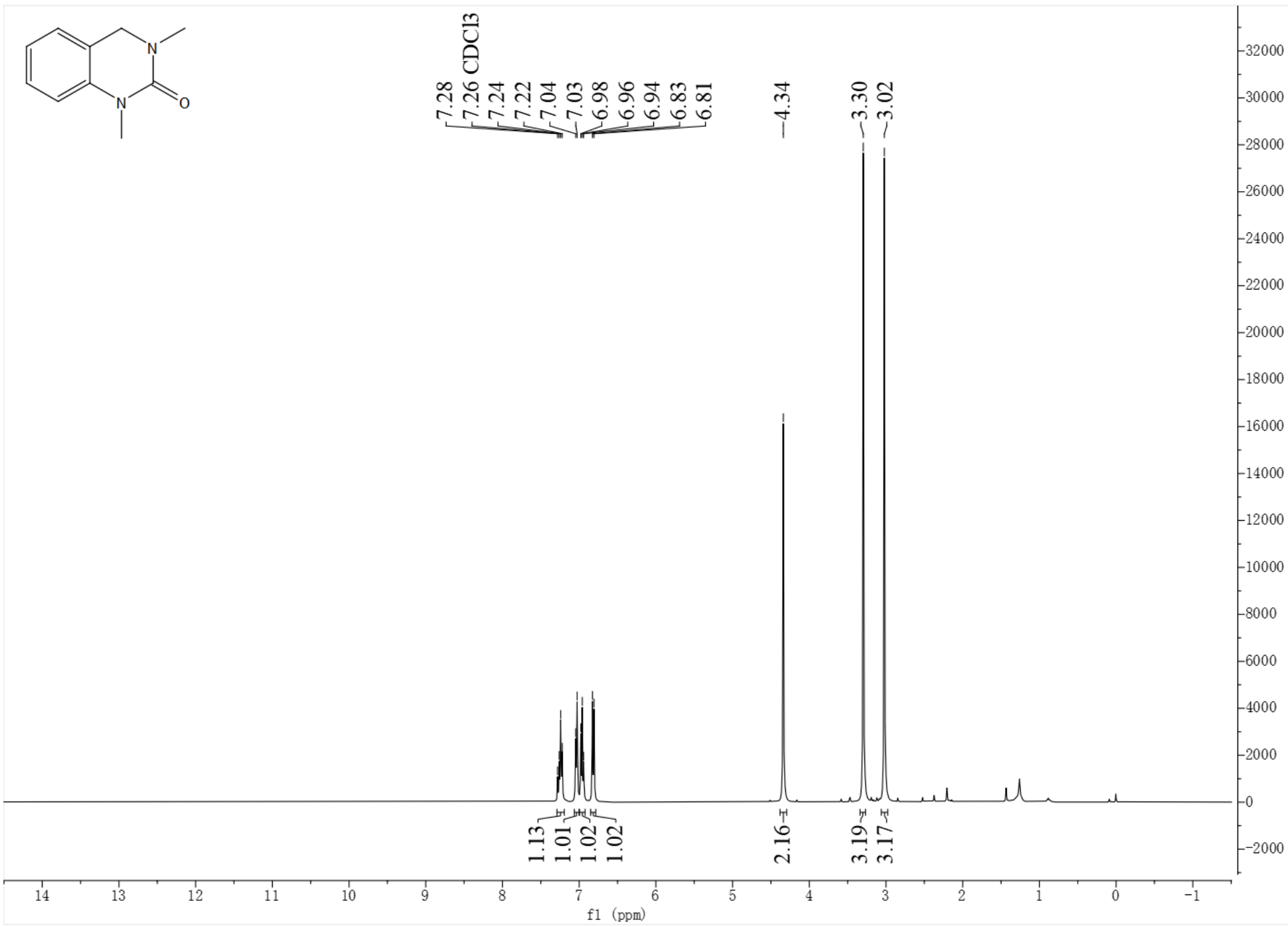

^{1}H NMR (400 MHz, chloroform-*d*) spectrum of compound **5**.

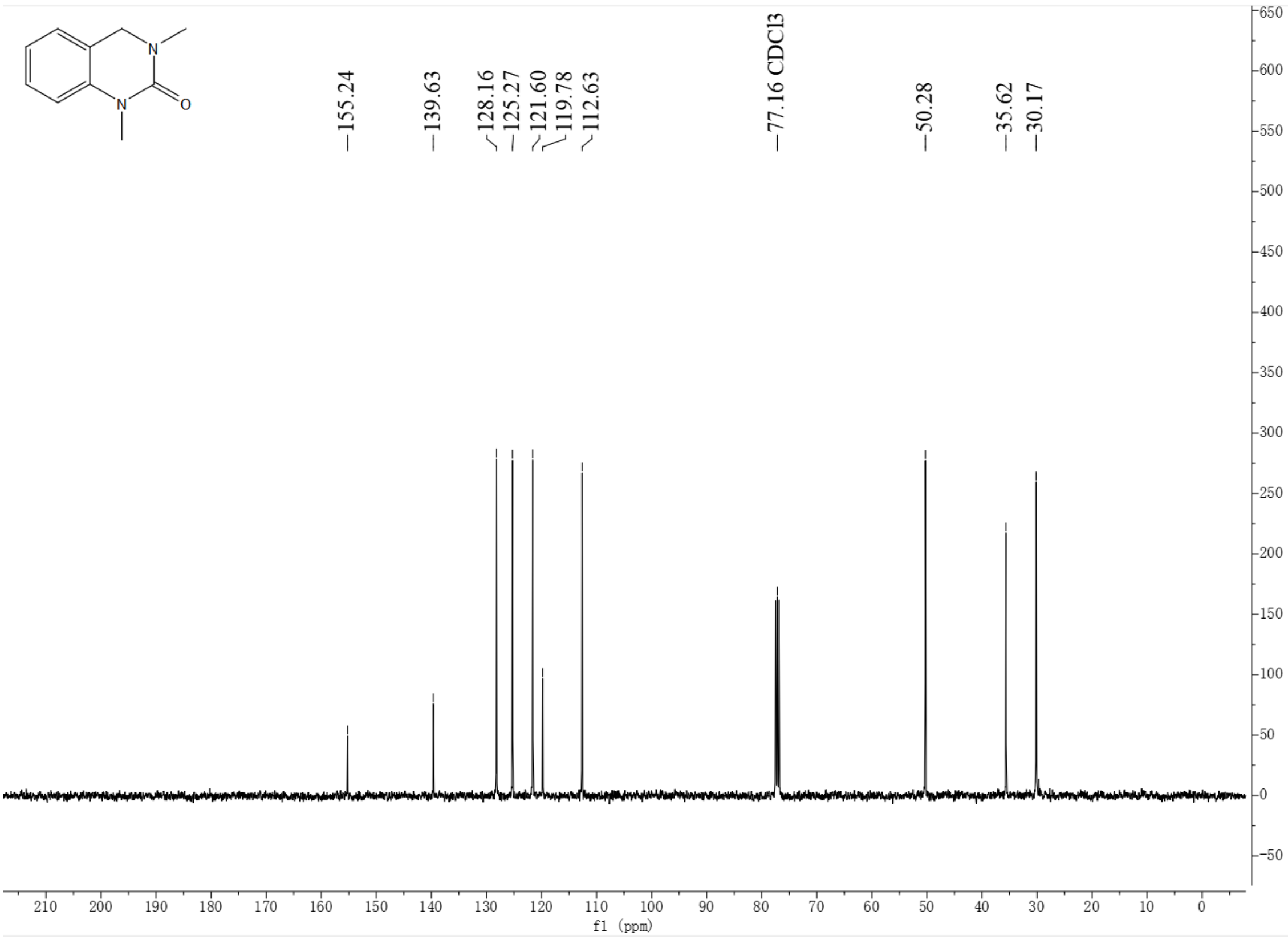

^{13}C{^{1}H} NMR (101 MHz, chloroform-*d*) spectrum of compound **5**.

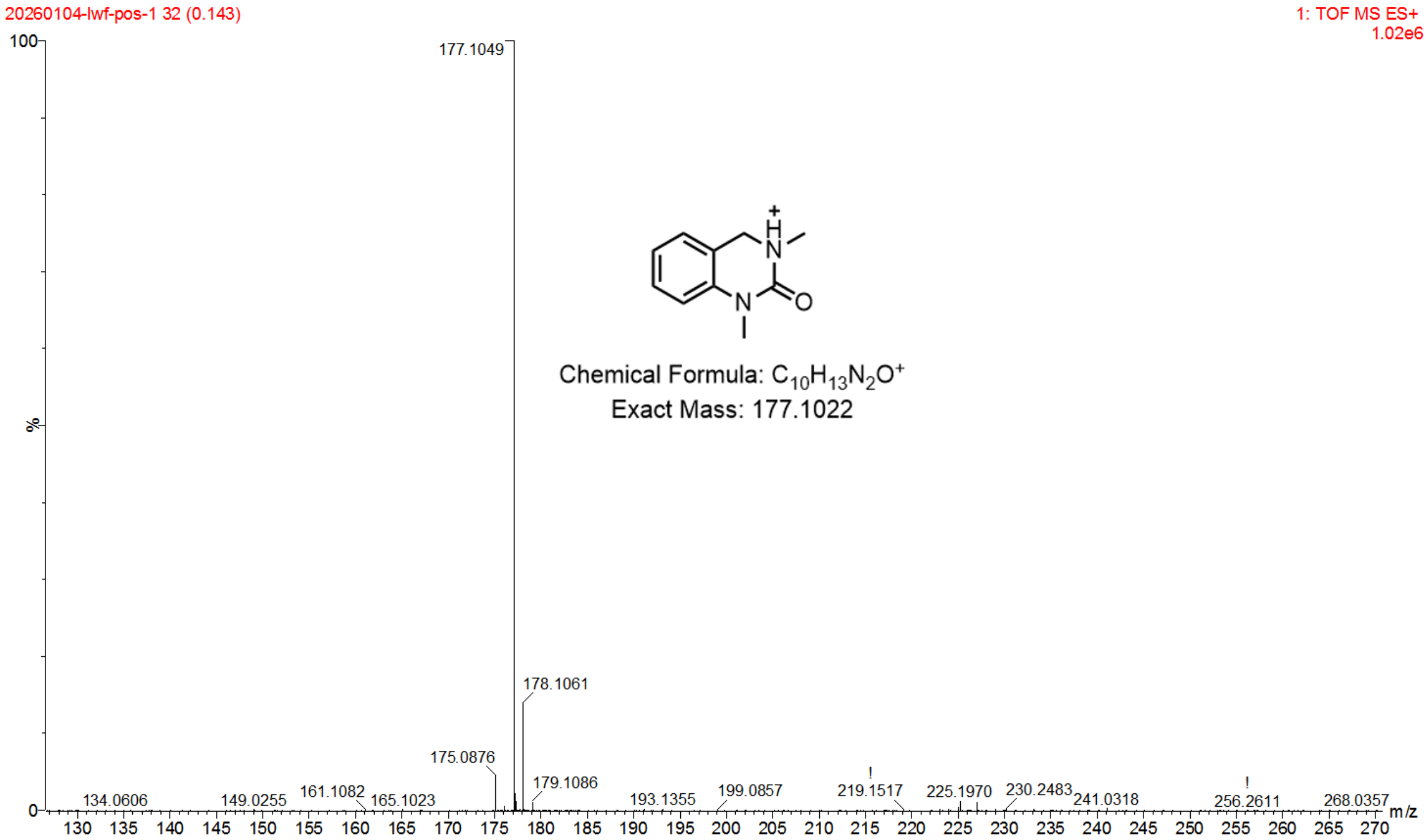

HRMS (ESI) of compound **5**.

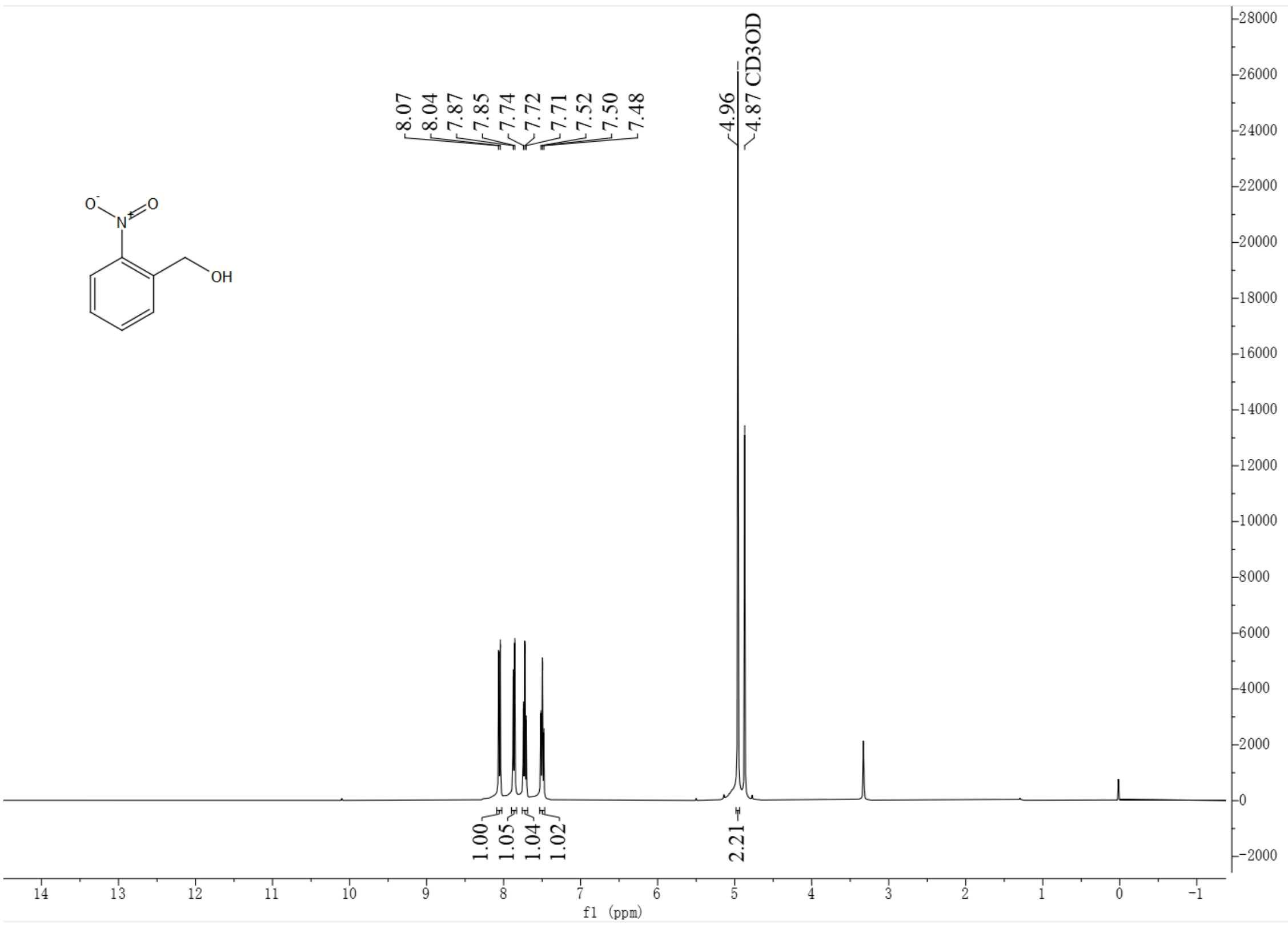

^{1}H NMR (400 MHz, methanol-*d*4) spectrum of compound **6**.

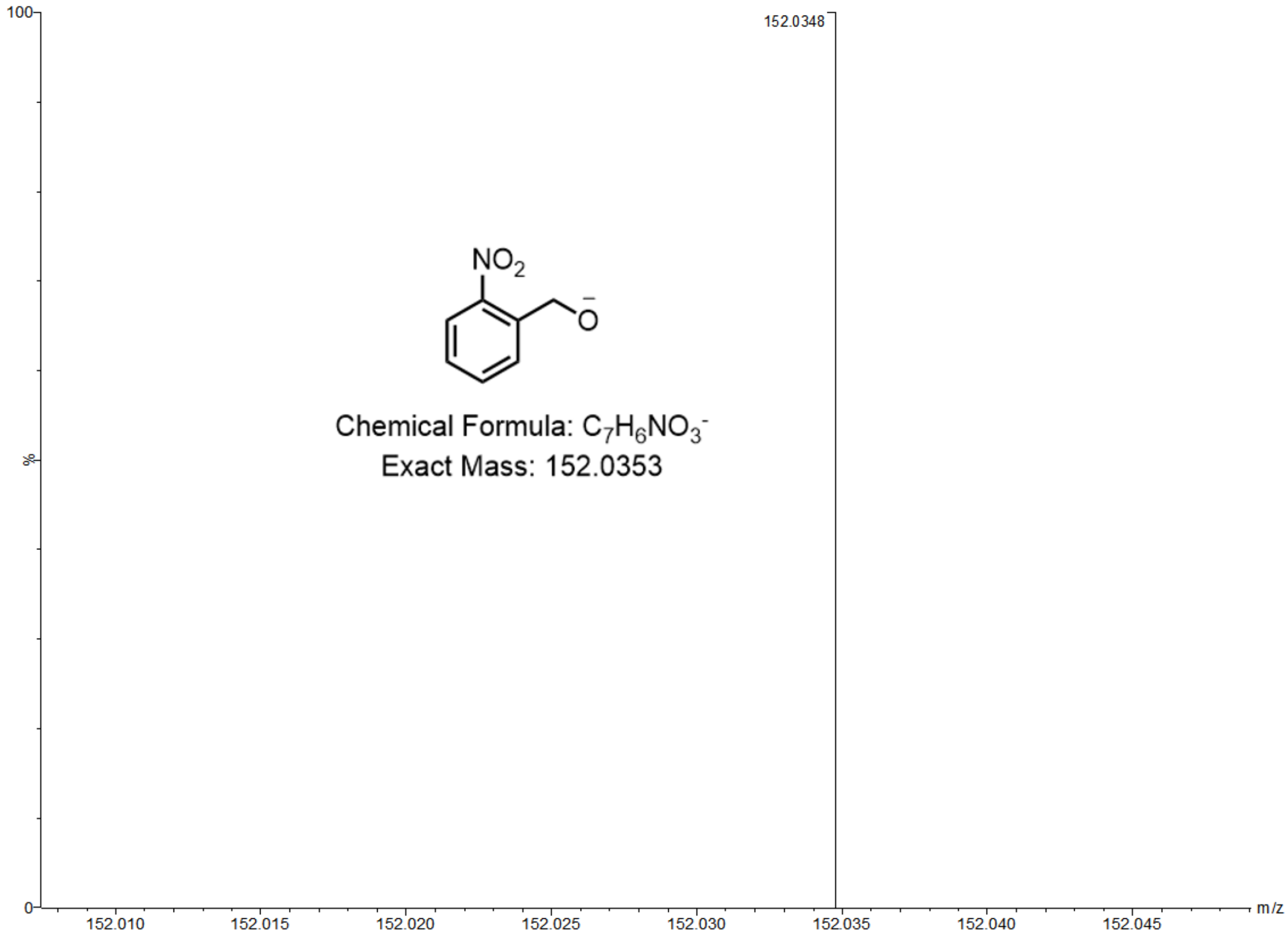

HRMS (ESI) of compound **6**.

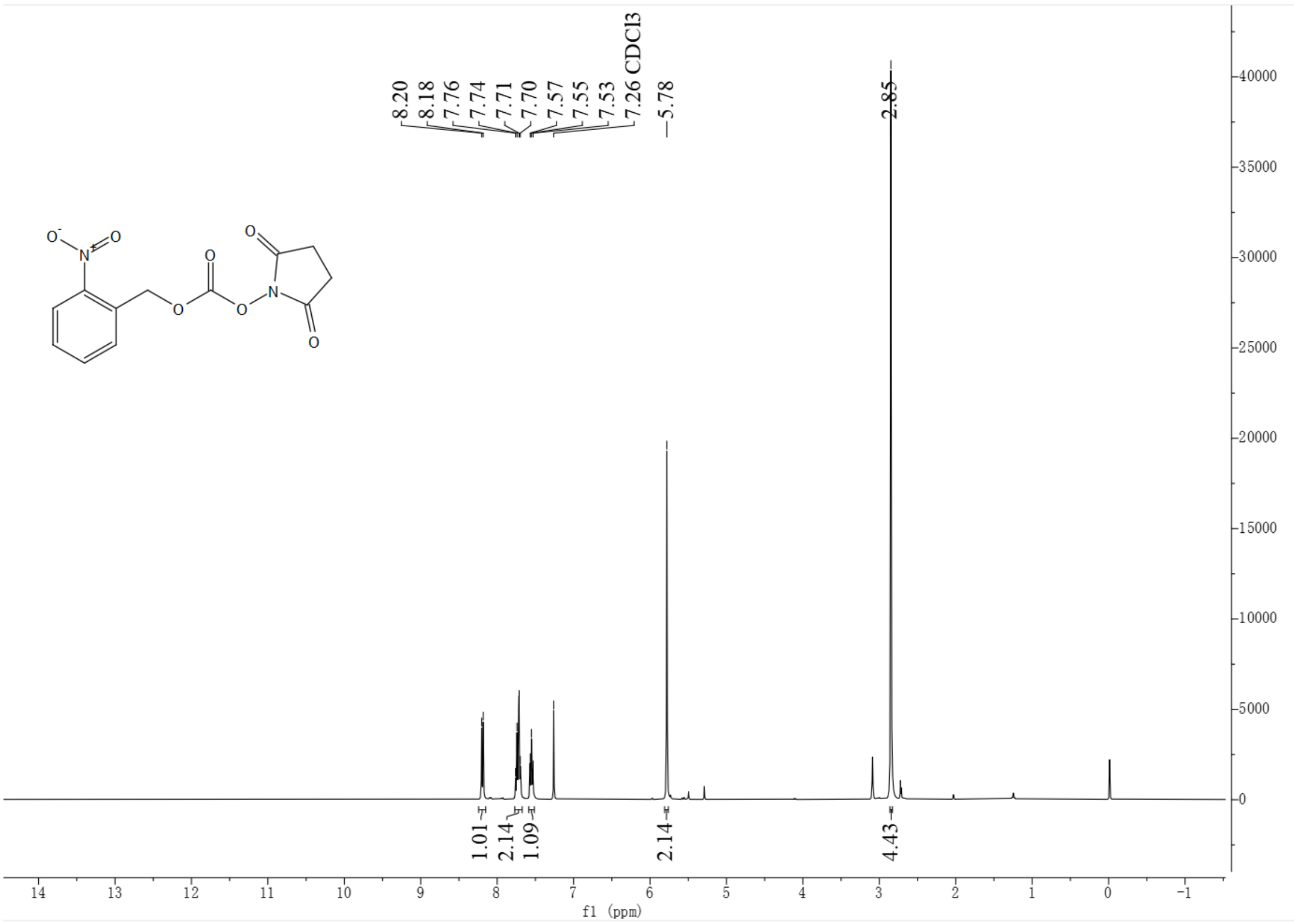

^{1}H NMR (400 MHz, chloroform-*d*) spectrum of compound **7**.

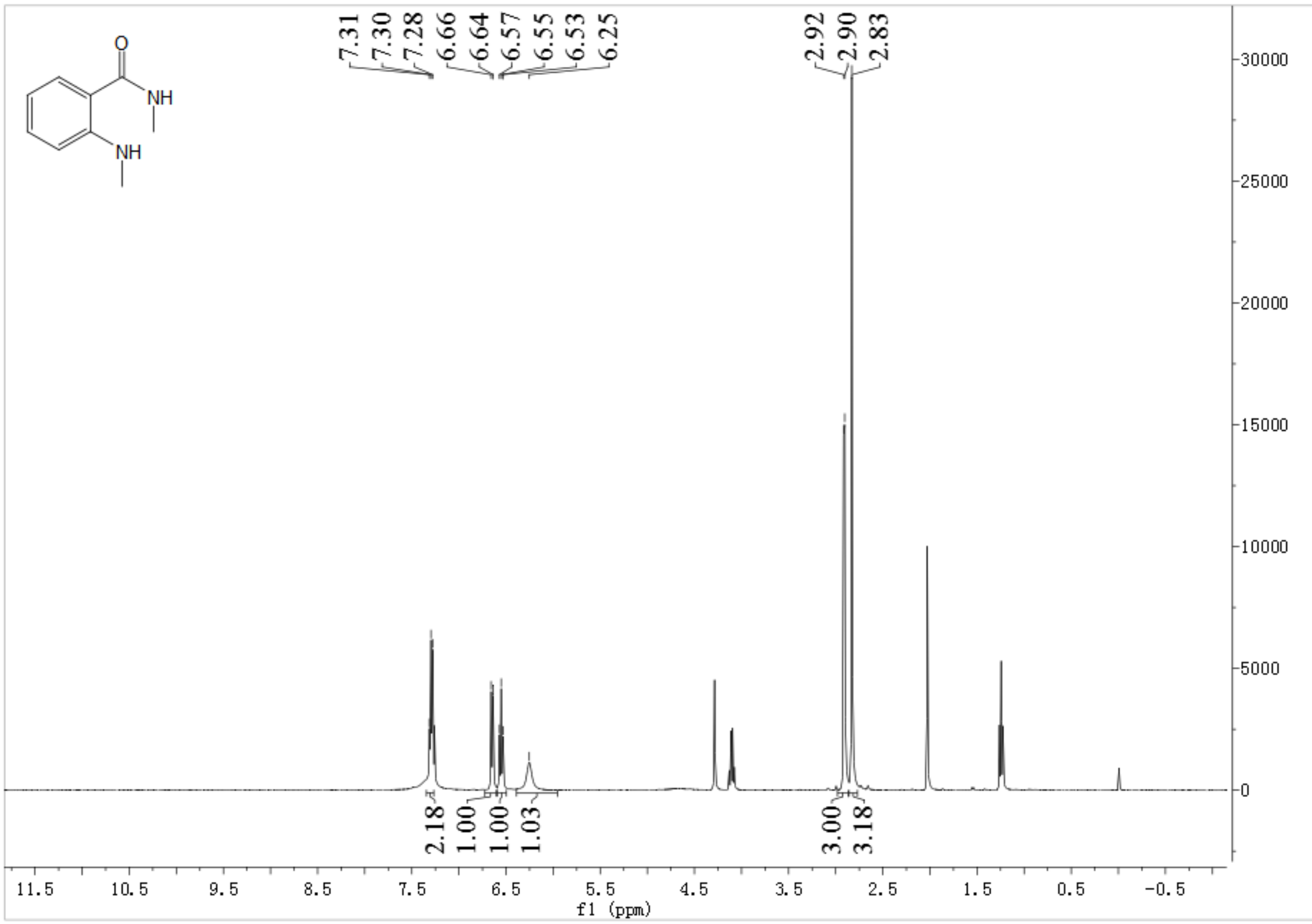

^{1}H NMR (400 MHz, chloroform-*d*) spectrum of compound **8**.

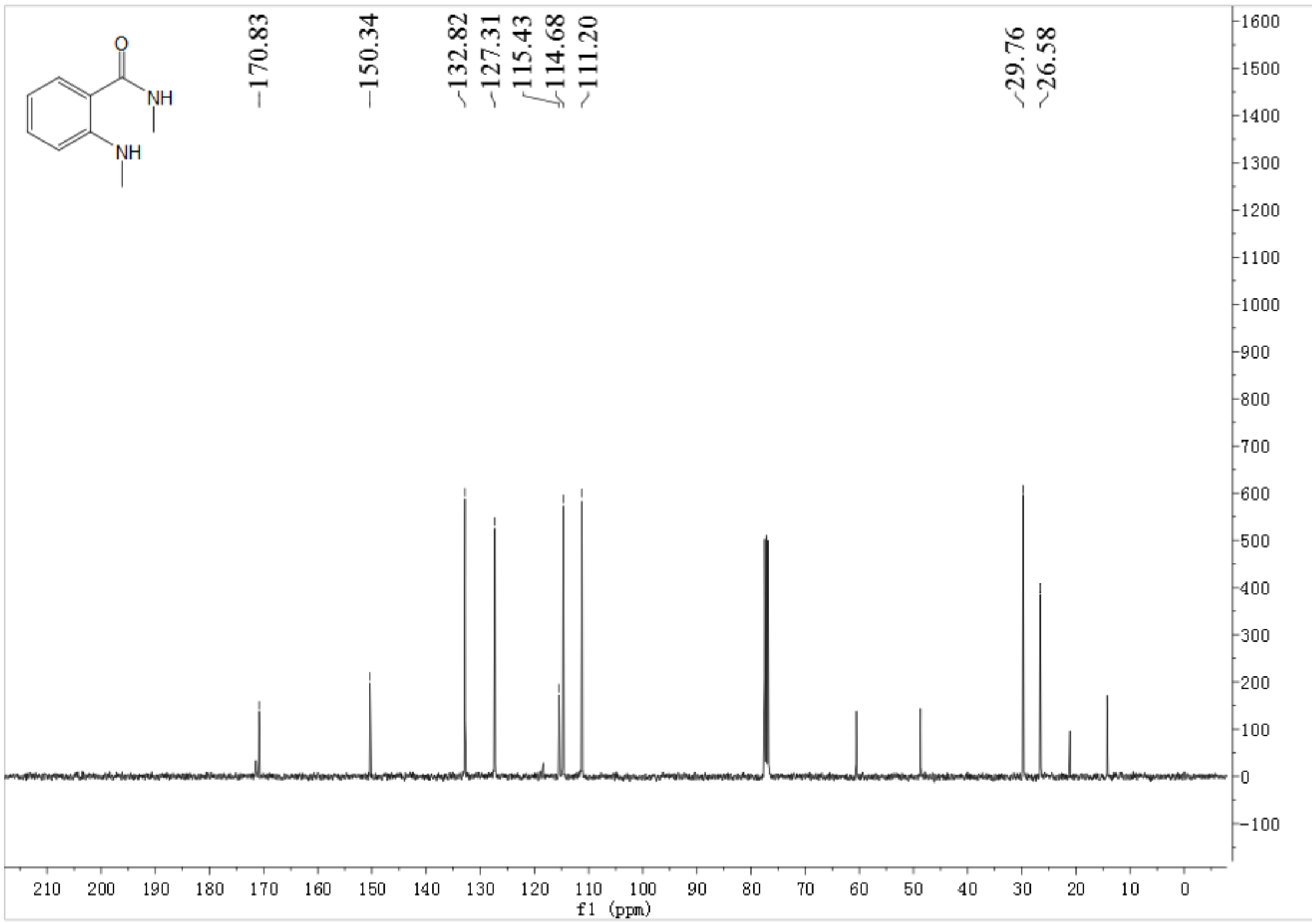

^{13}C{^{1}H} NMR (101 MHz, chloroform-*d*) spectrum of compound **8**.

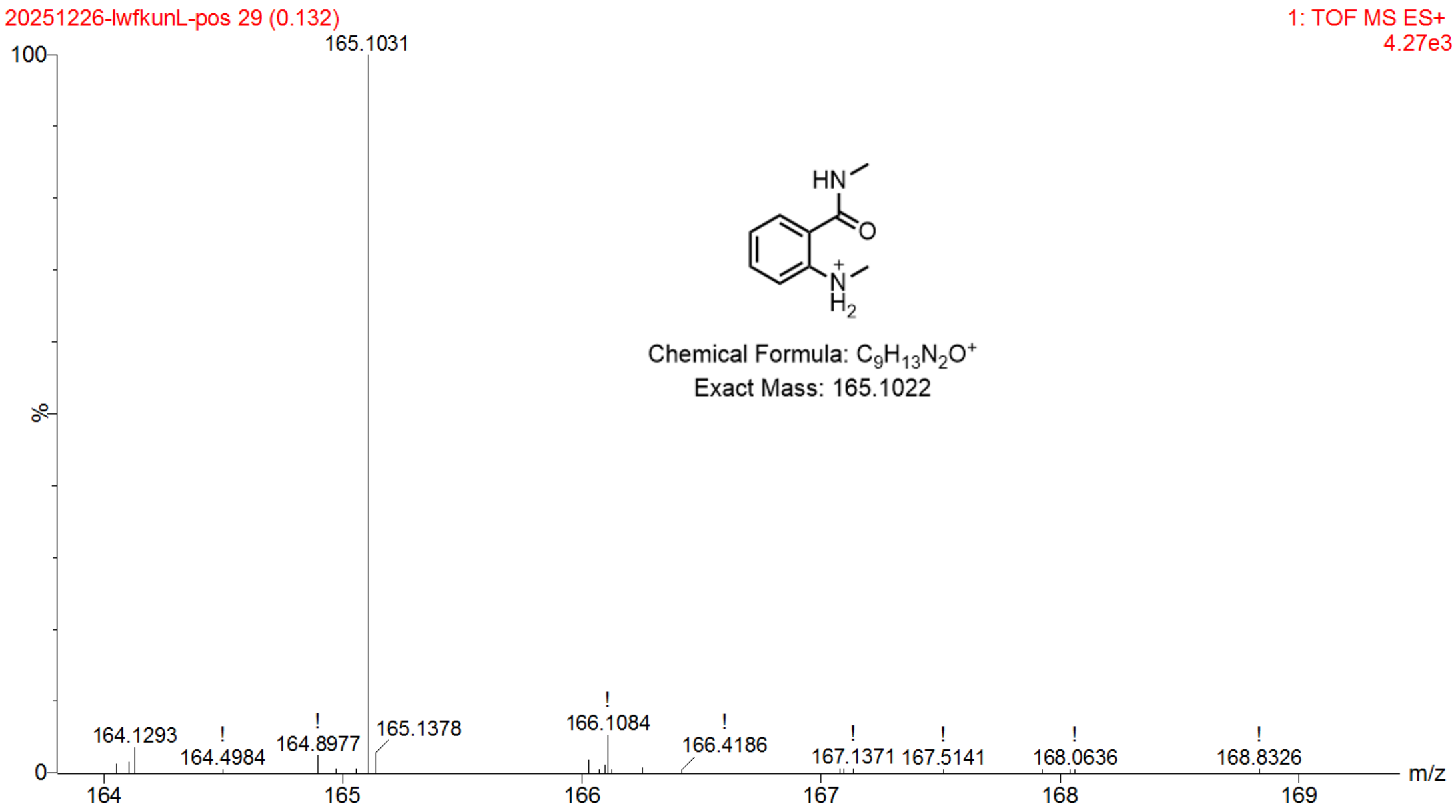

HRMS (ESI) of compound **8**.

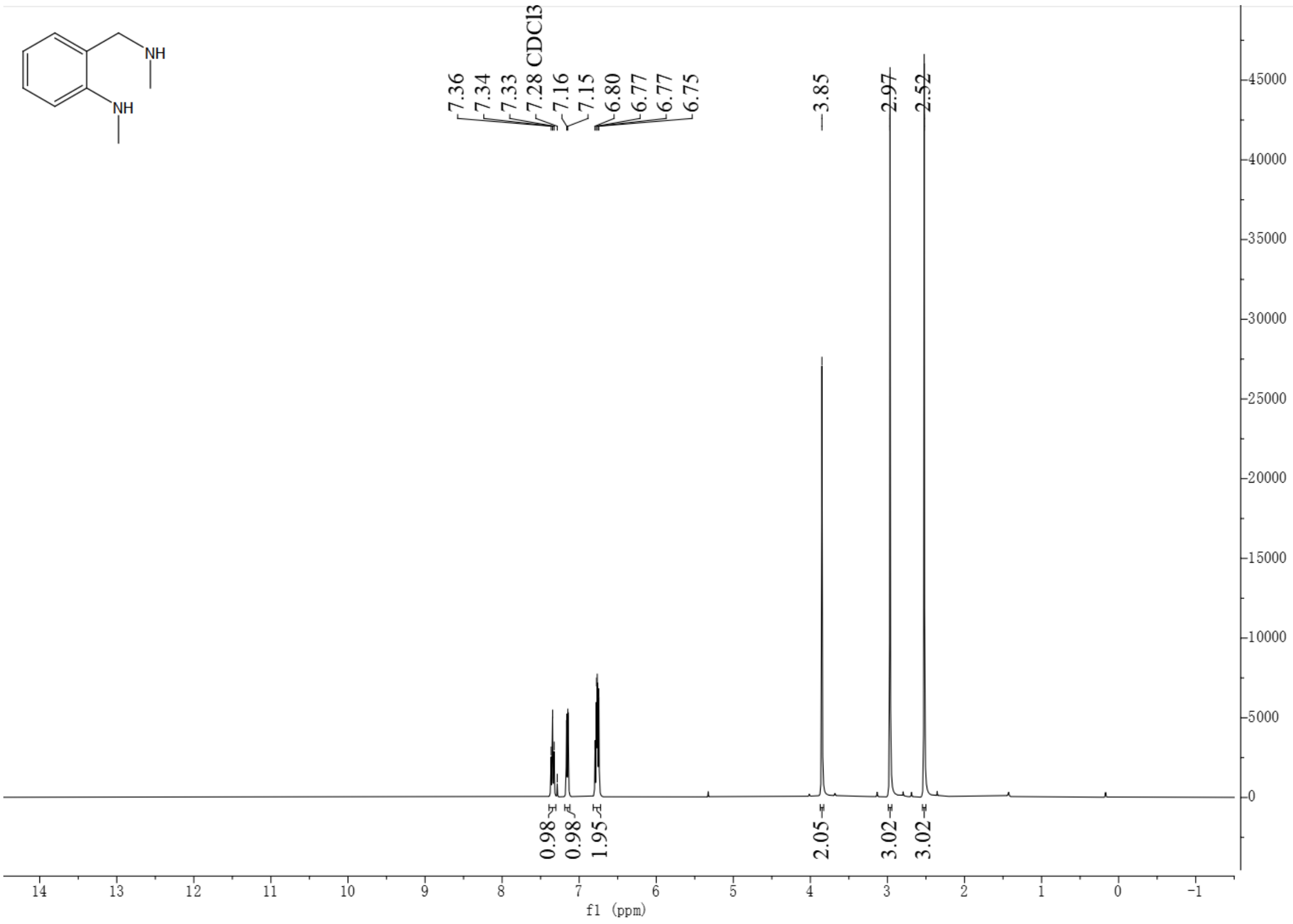

^{1}H NMR (400 MHz, chloroform-*d*) spectrum of compound **9**.

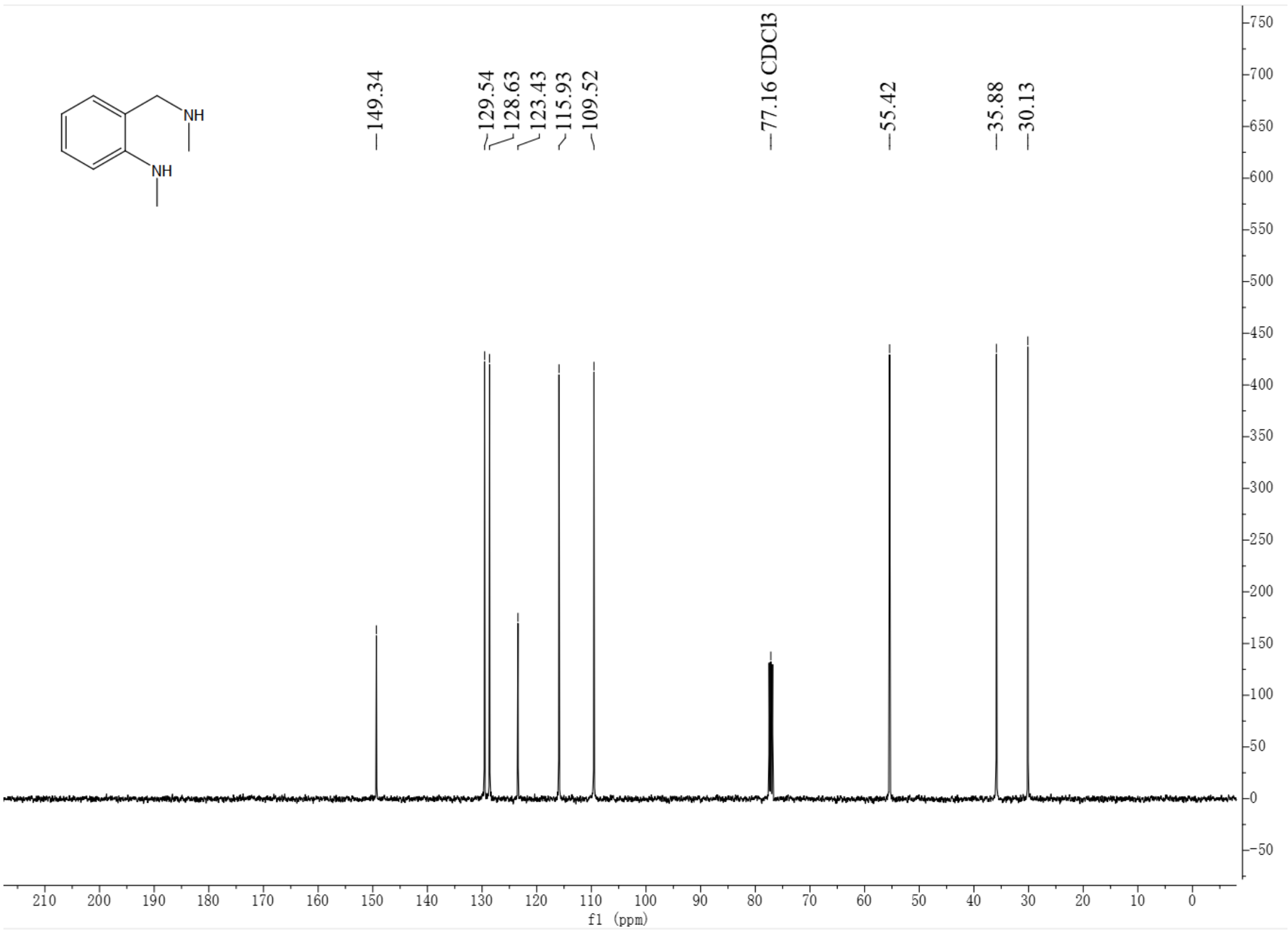

$^{13}C\{^{1}H\}$ NMR (101 MHz, chloroform-*d*) spectrum of compound **9**.

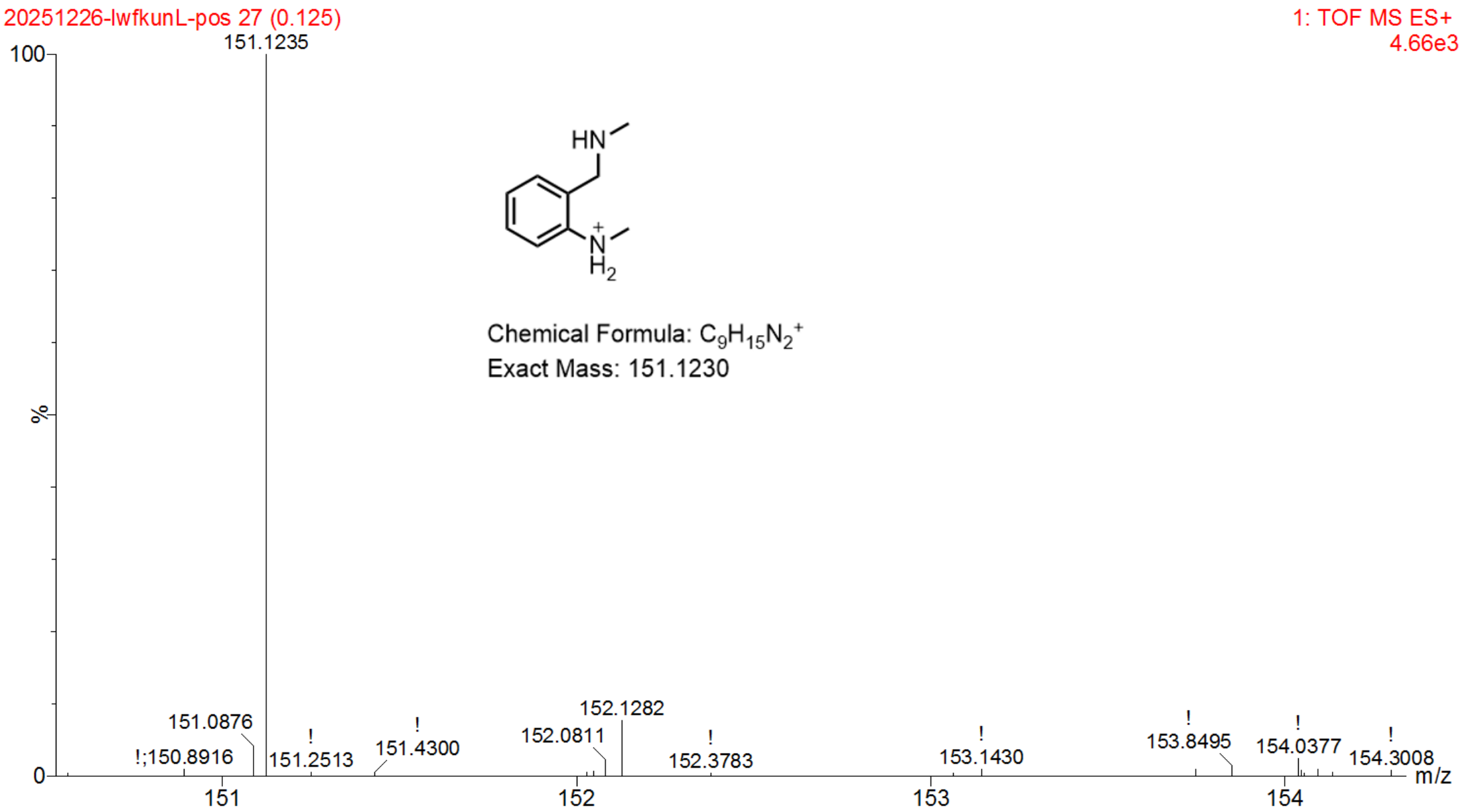

HRMS (ESI) of compound **9**.

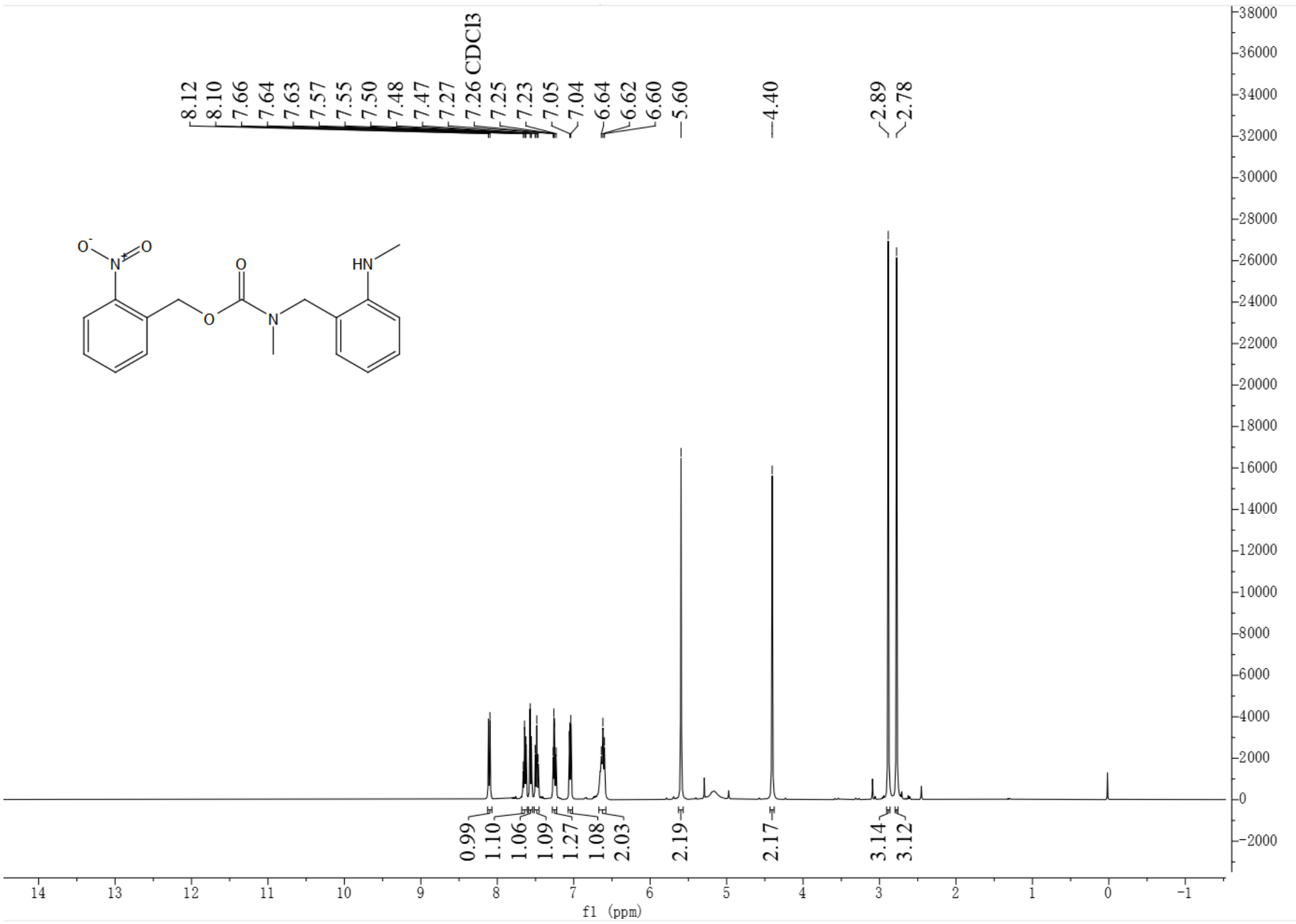

^{1}H NMR (400 MHz, chloroform-*d*) spectrum of compound **10**.

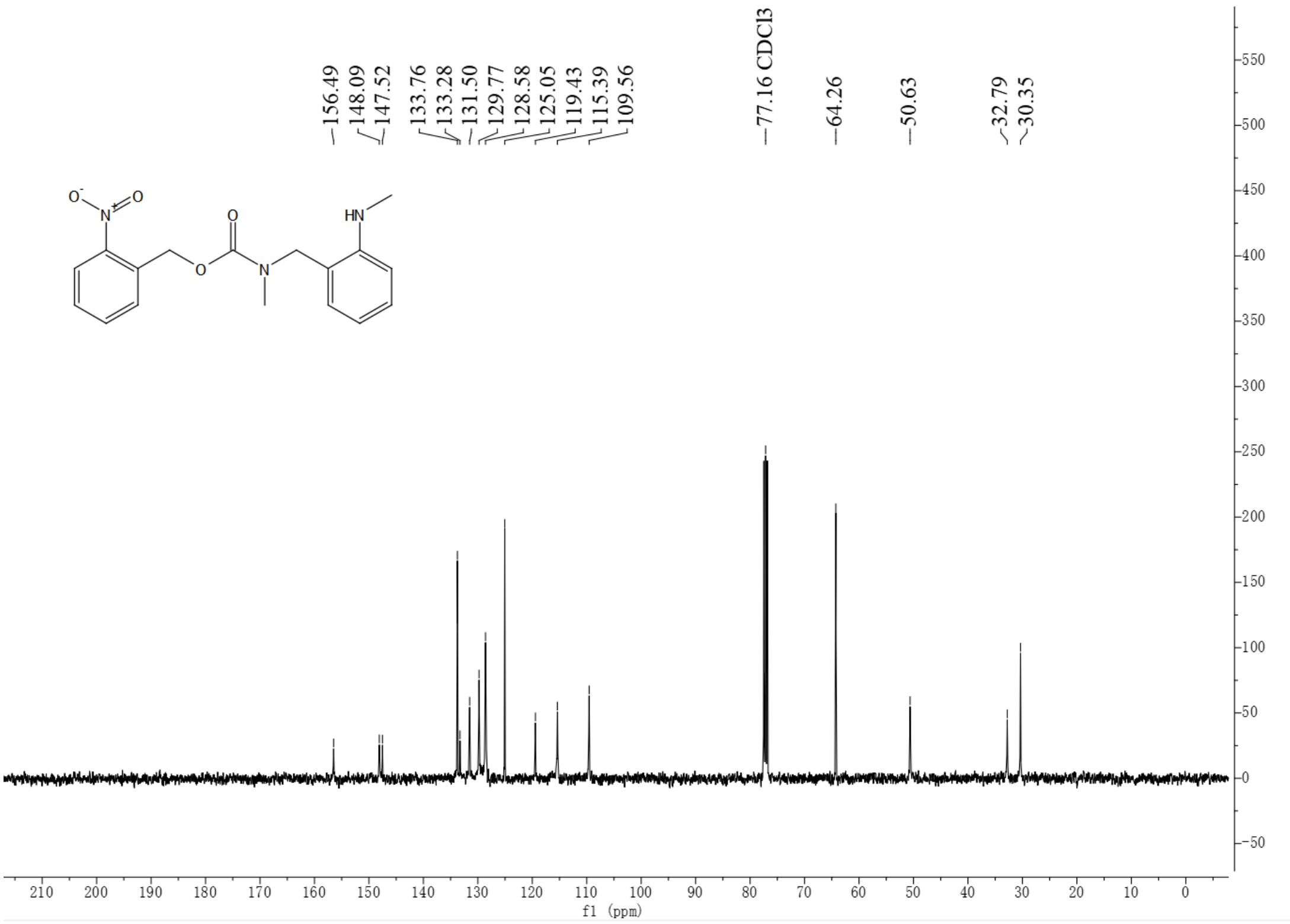

^{13}C{^{1}H} NMR (101 MHz, chloroform-*d*) spectrum of compound **10**.

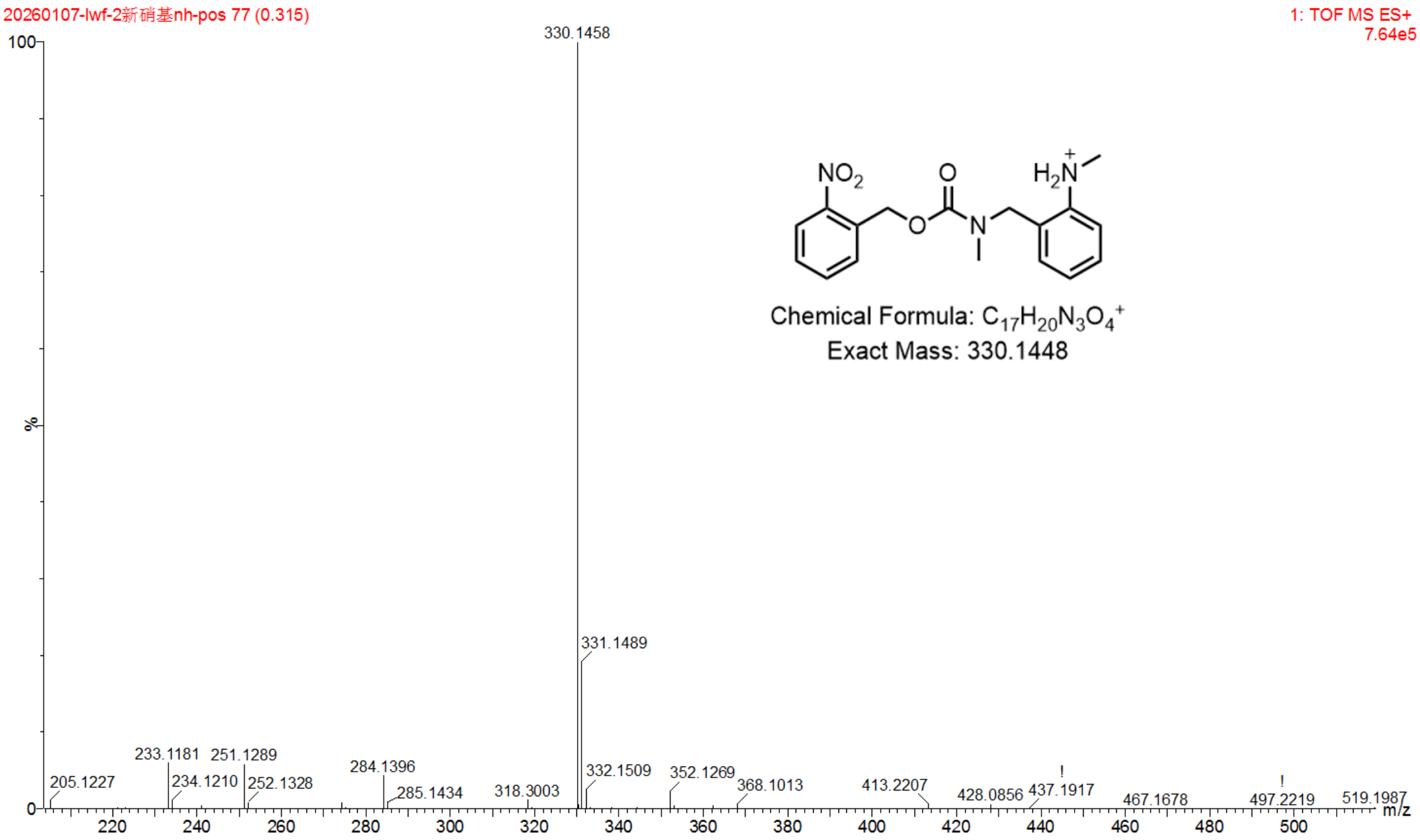

HRMS (ESI) of compound **10**.

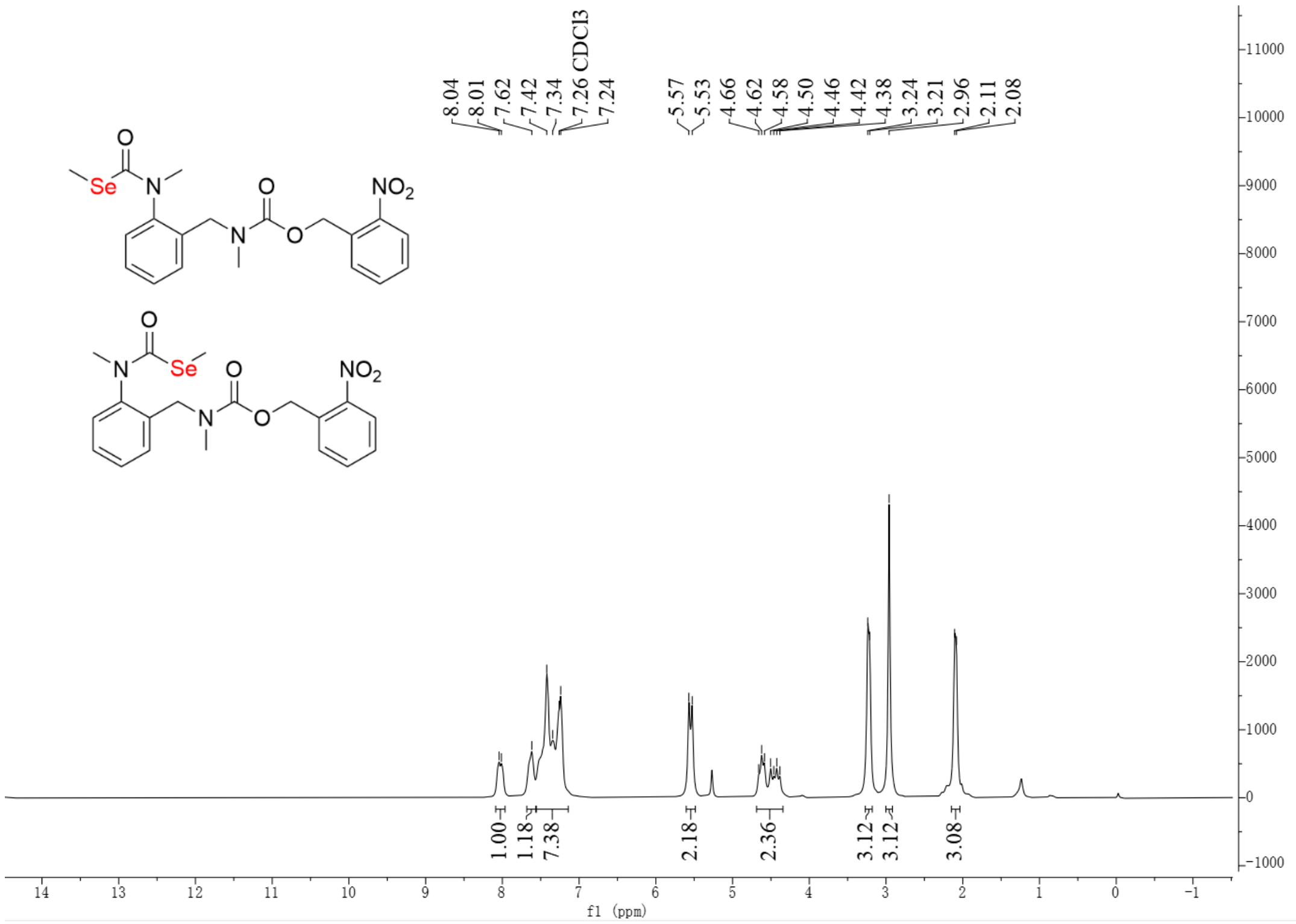

^{1}H NMR (400 MHz, chloroform-*d*) spectrum of compound **TD-MSe2**. With regard to the conformational behaviors, it should be noted that an intramolecular interaction between Se and O atoms may exist via the chalcogen bonding (ChB).[13] Based on the NMR data, we propose two conformations for **TD-MSe2** as shown inset.

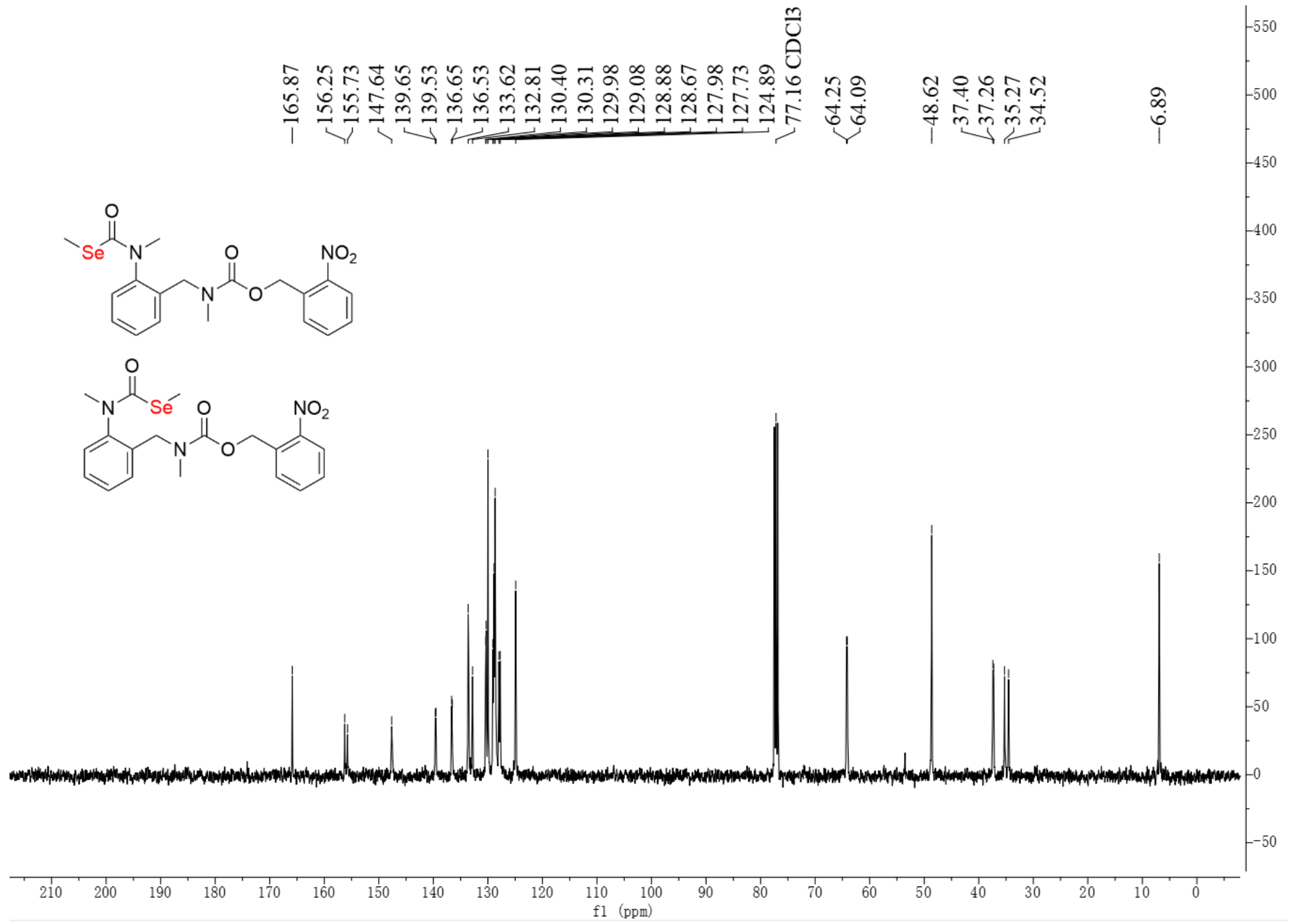

$^{13}C\{^{1}H\}$ NMR (101 MHz, chloroform-*d*) spectrum of compound **TD-MSe2**.

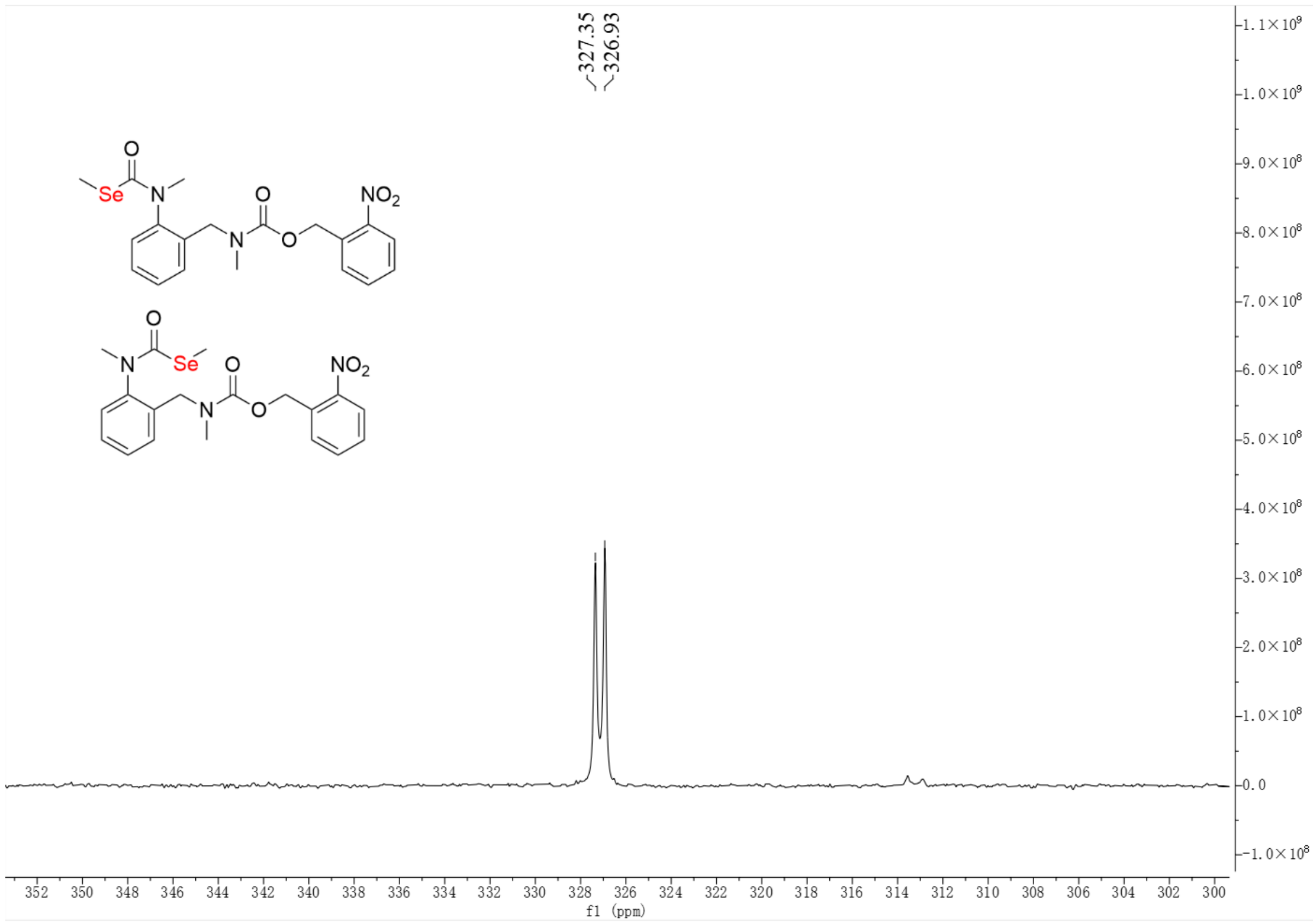

$^{77}Se\{^{1}H\}$ NMR (114 MHz, chloroform-*d*) spectrum of compound **TD-MSe2**.

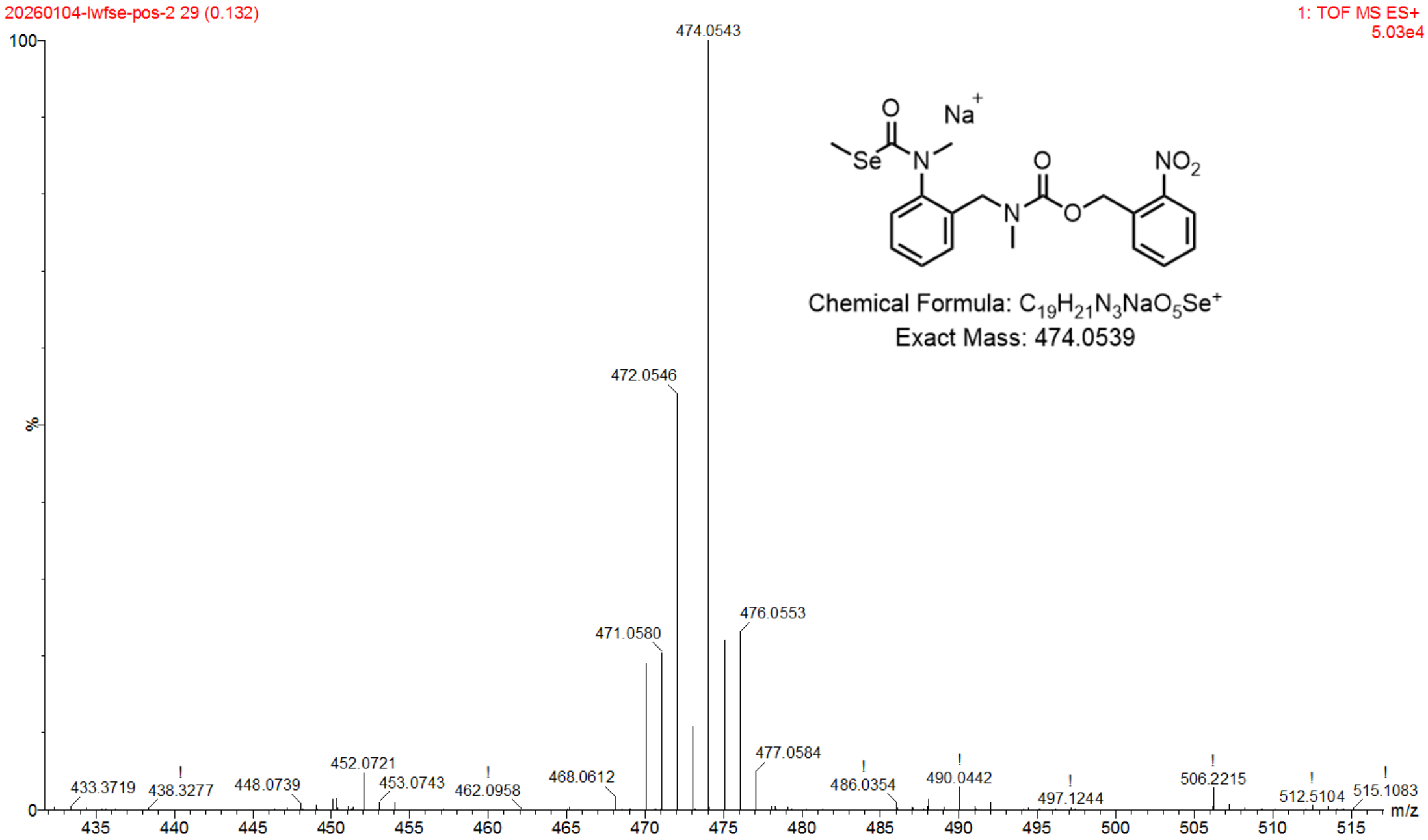

HRMS (ESI) of compound **TD-MSe2**.

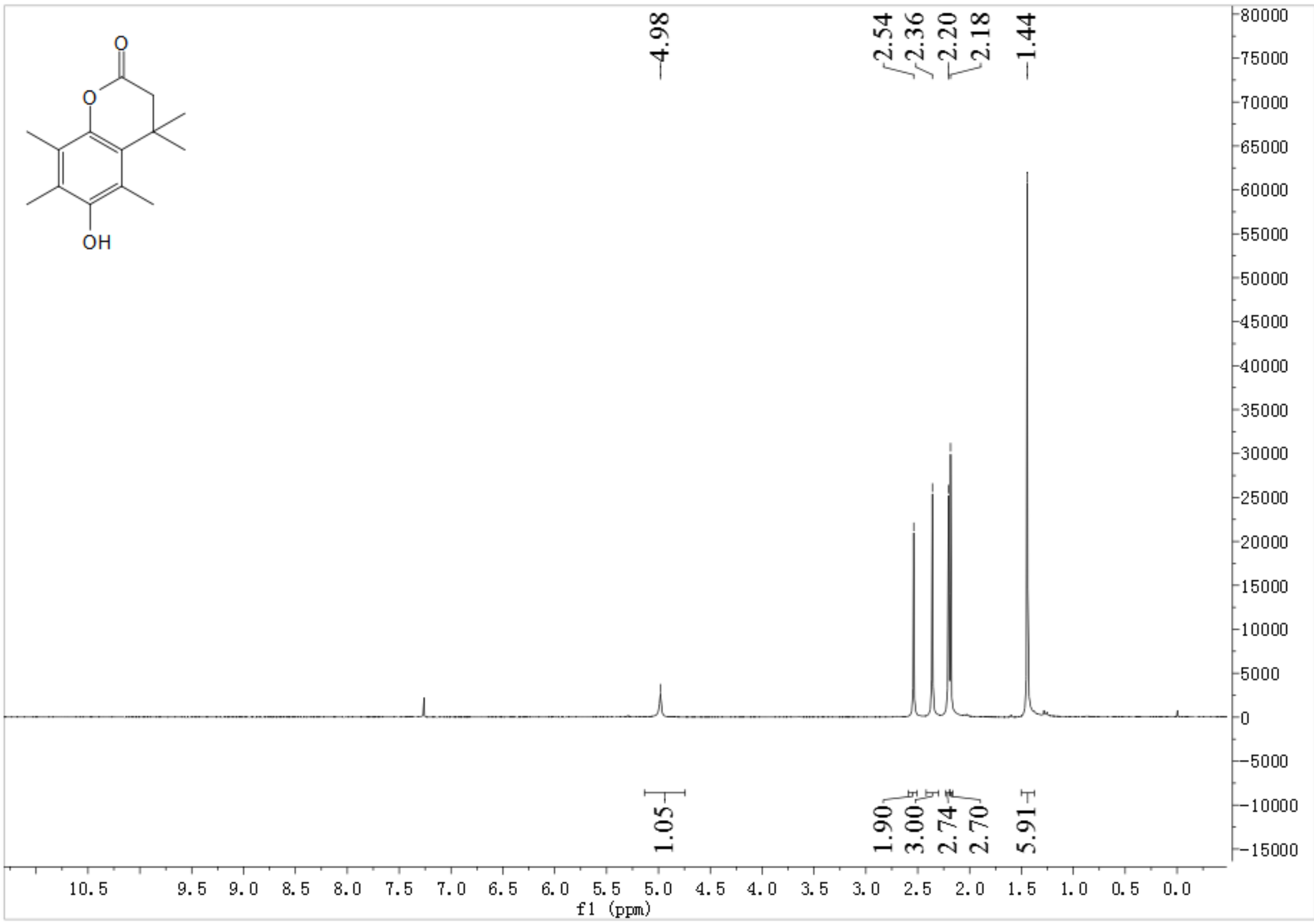

^{1}H NMR (400 MHz, chloroform-*d*) spectrum of compound **11**.

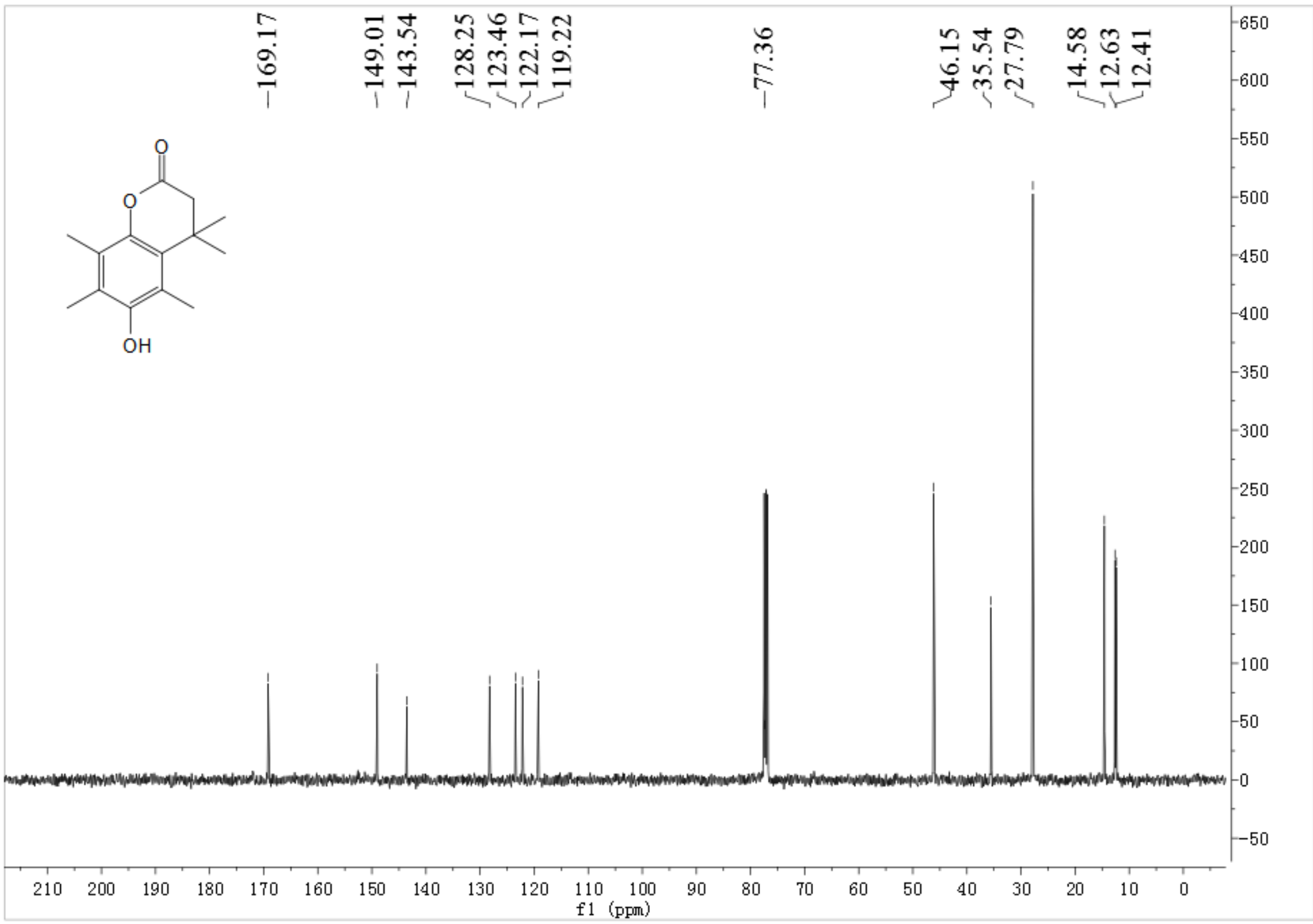

$^{13}C\{^1H\}$ NMR (101 MHz, chloroform-*d*) spectrum of compound **11**.

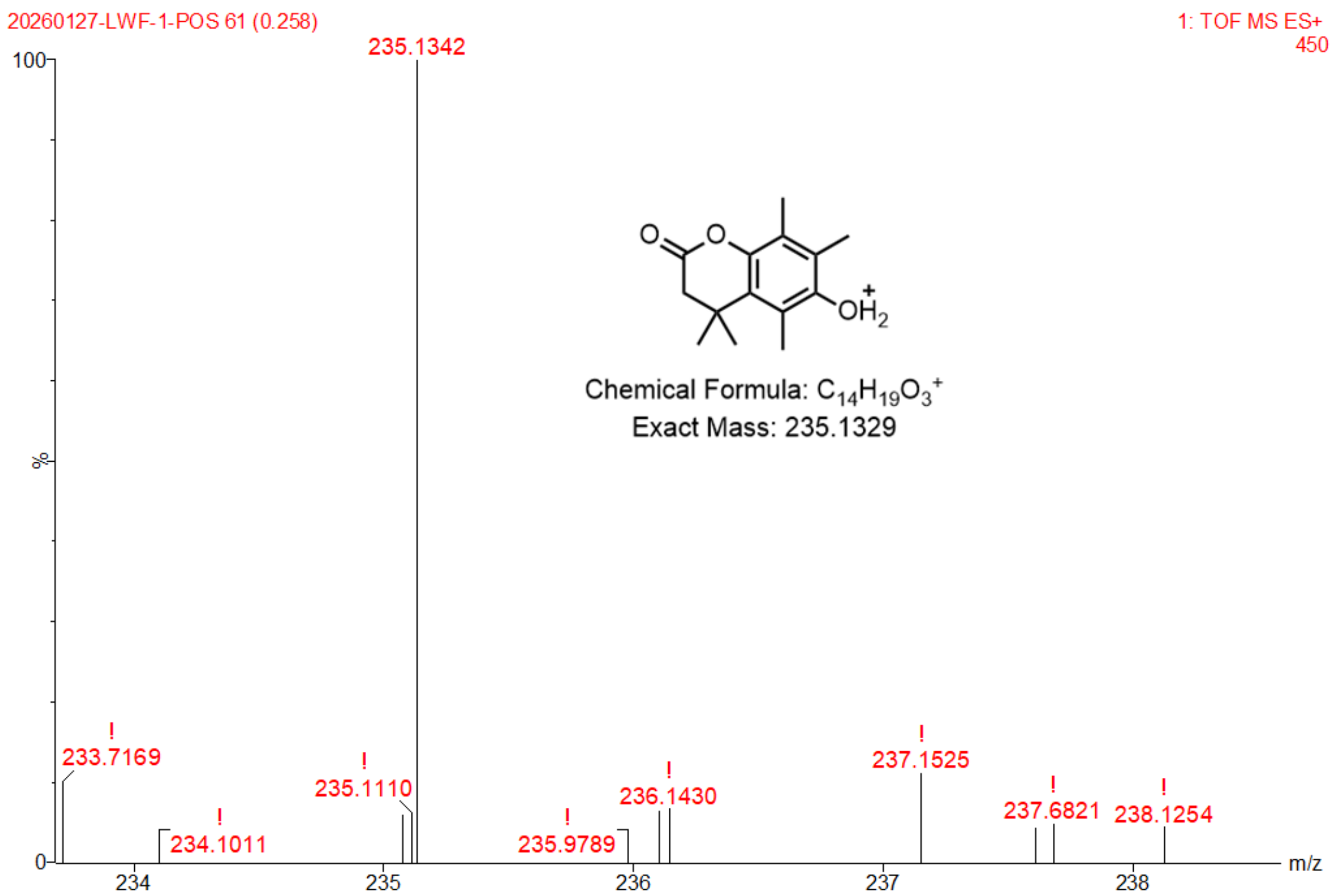

HRMS (ESI) of compound **11**.

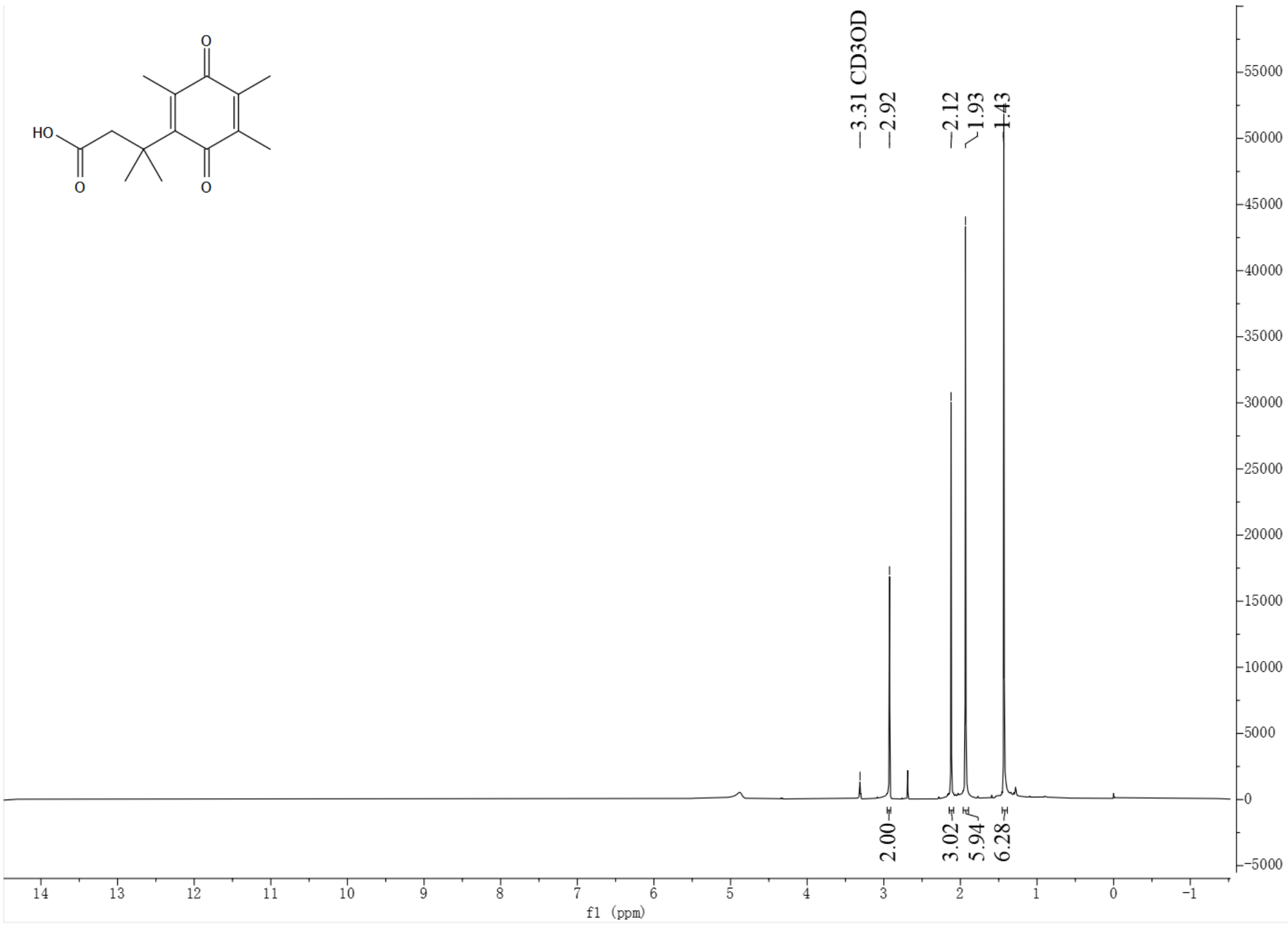

^{1}H NMR (400 MHz, methanol-d_4) spectrum of compound **12**.

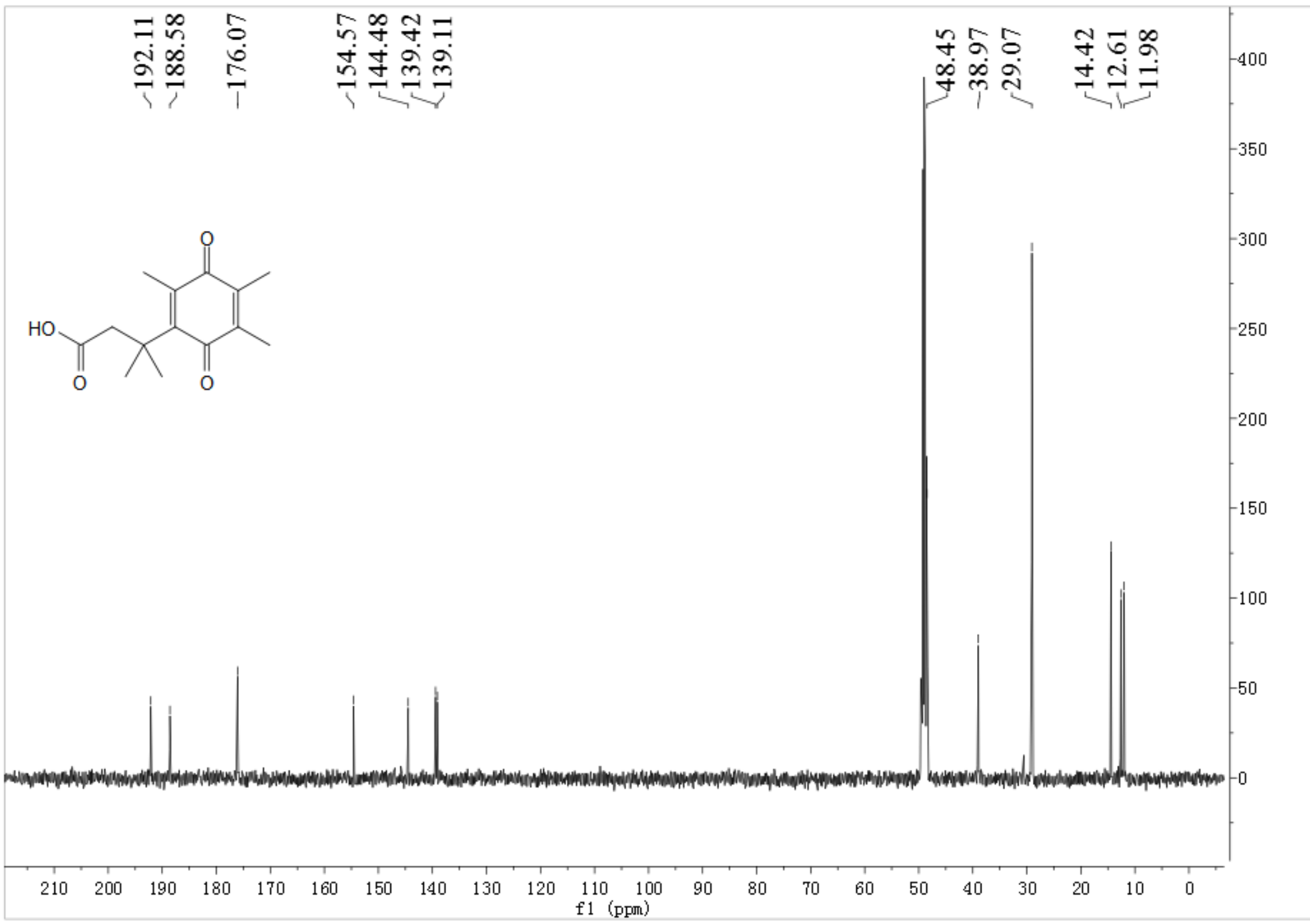

^{13}C{^{1}H} NMR (101 MHz, methanol-d_4) spectrum of compound **12**.

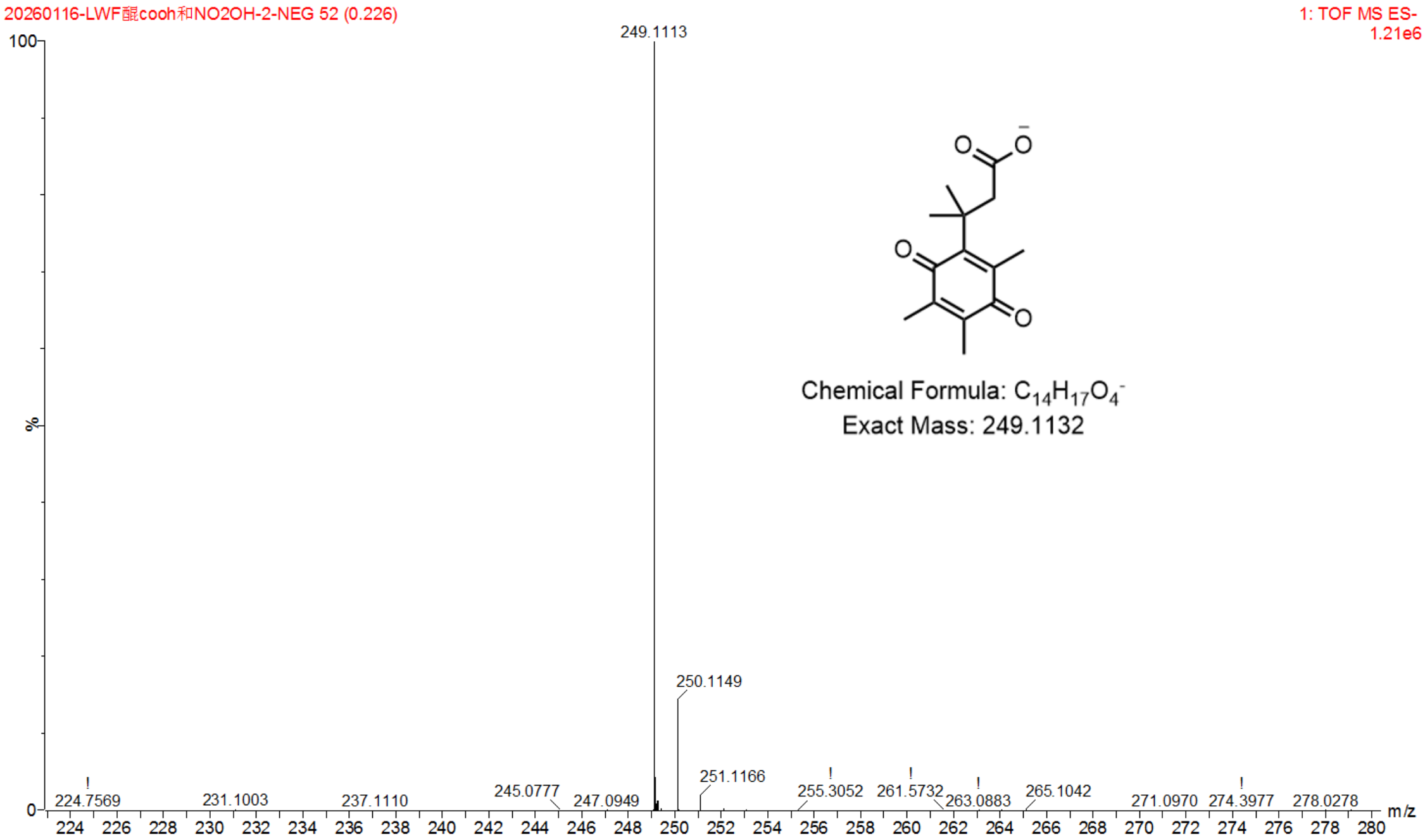

HRMS (ESI) of compound **12**.

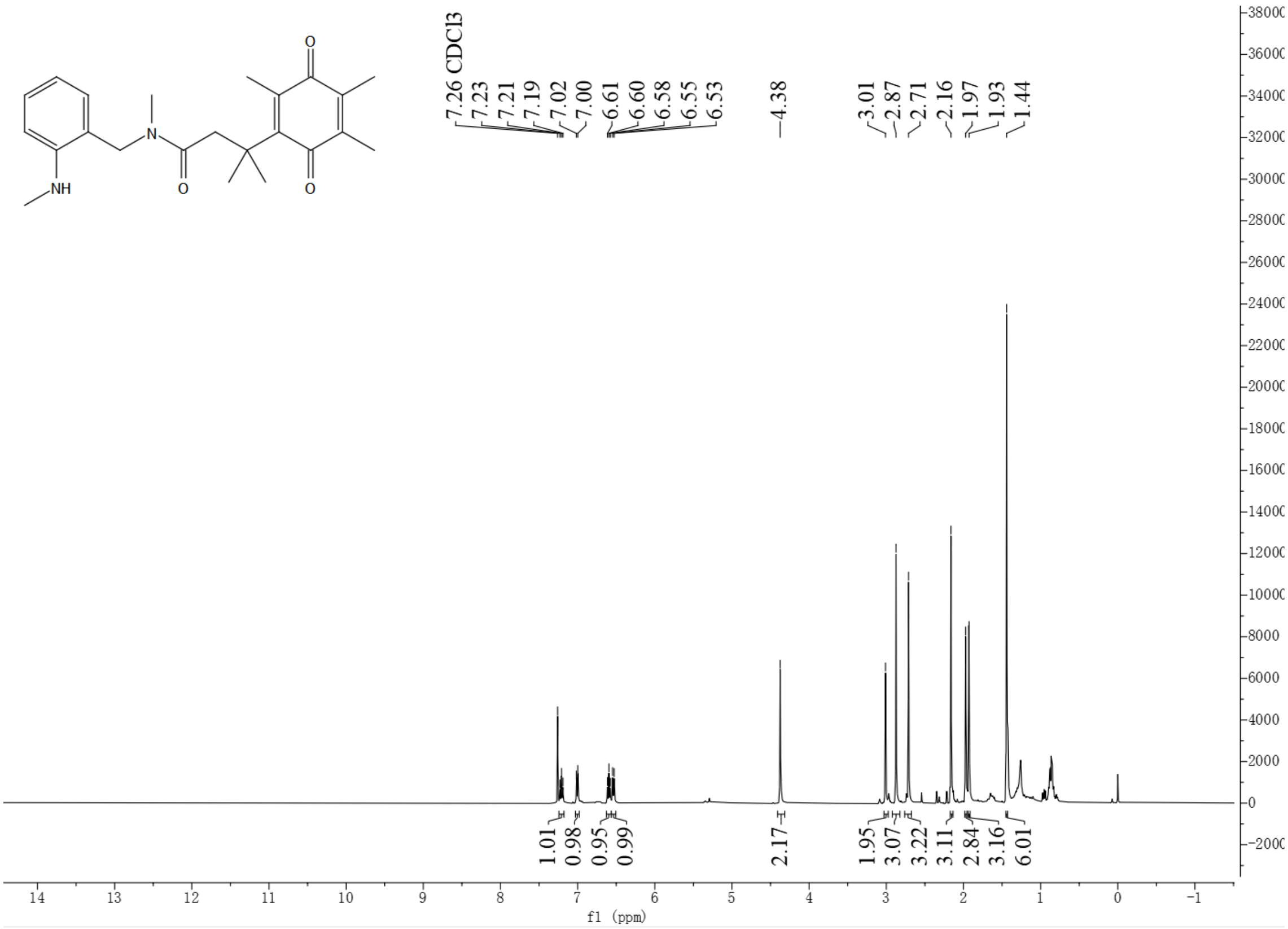

^{1}H NMR (400 MHz, chloroform-*d*) spectrum of compound **13**.

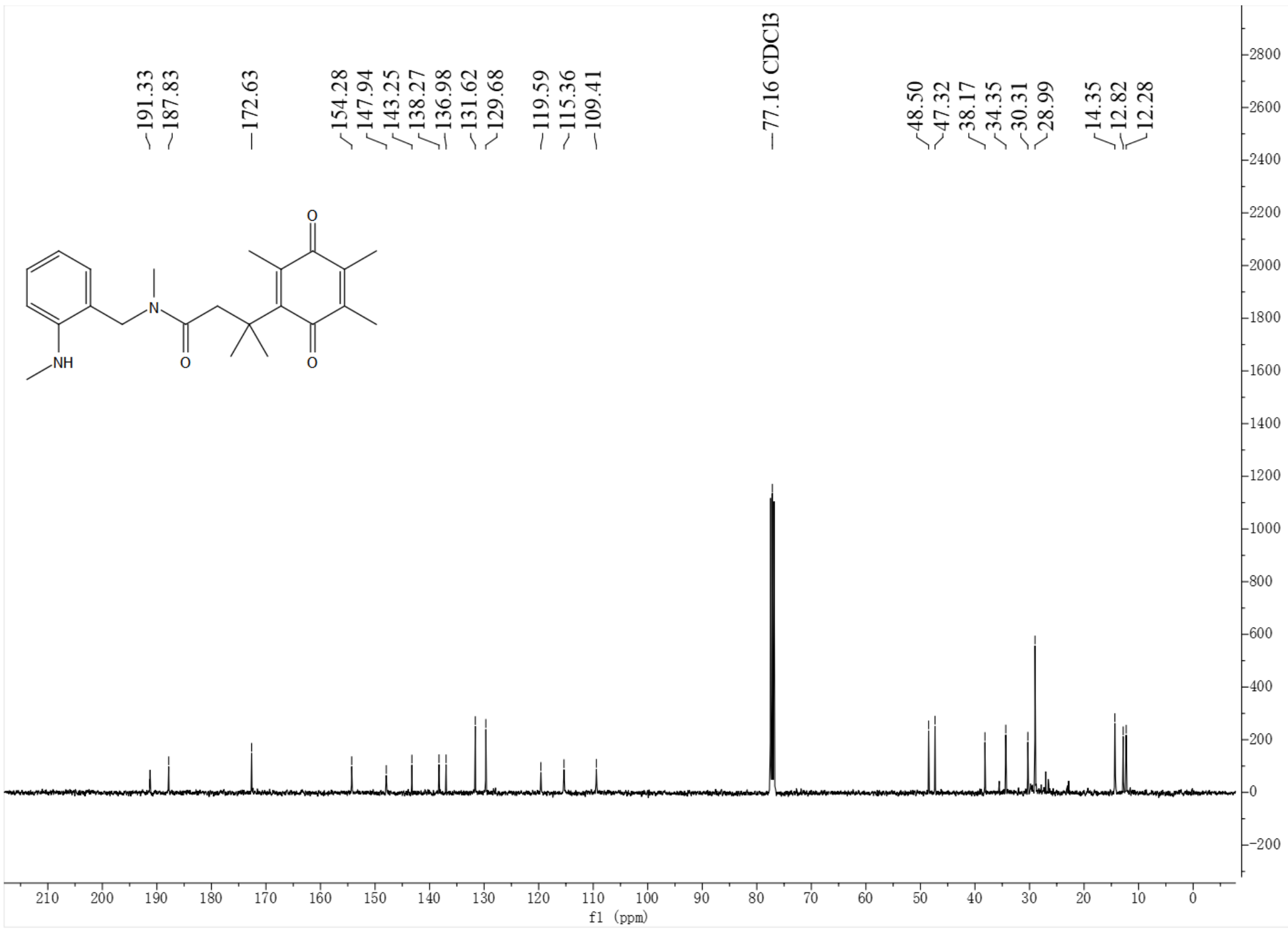

$^{13}C\{^{1}H\}$ NMR (101 MHz, chloroform-*d*) spectrum of compound **13**.

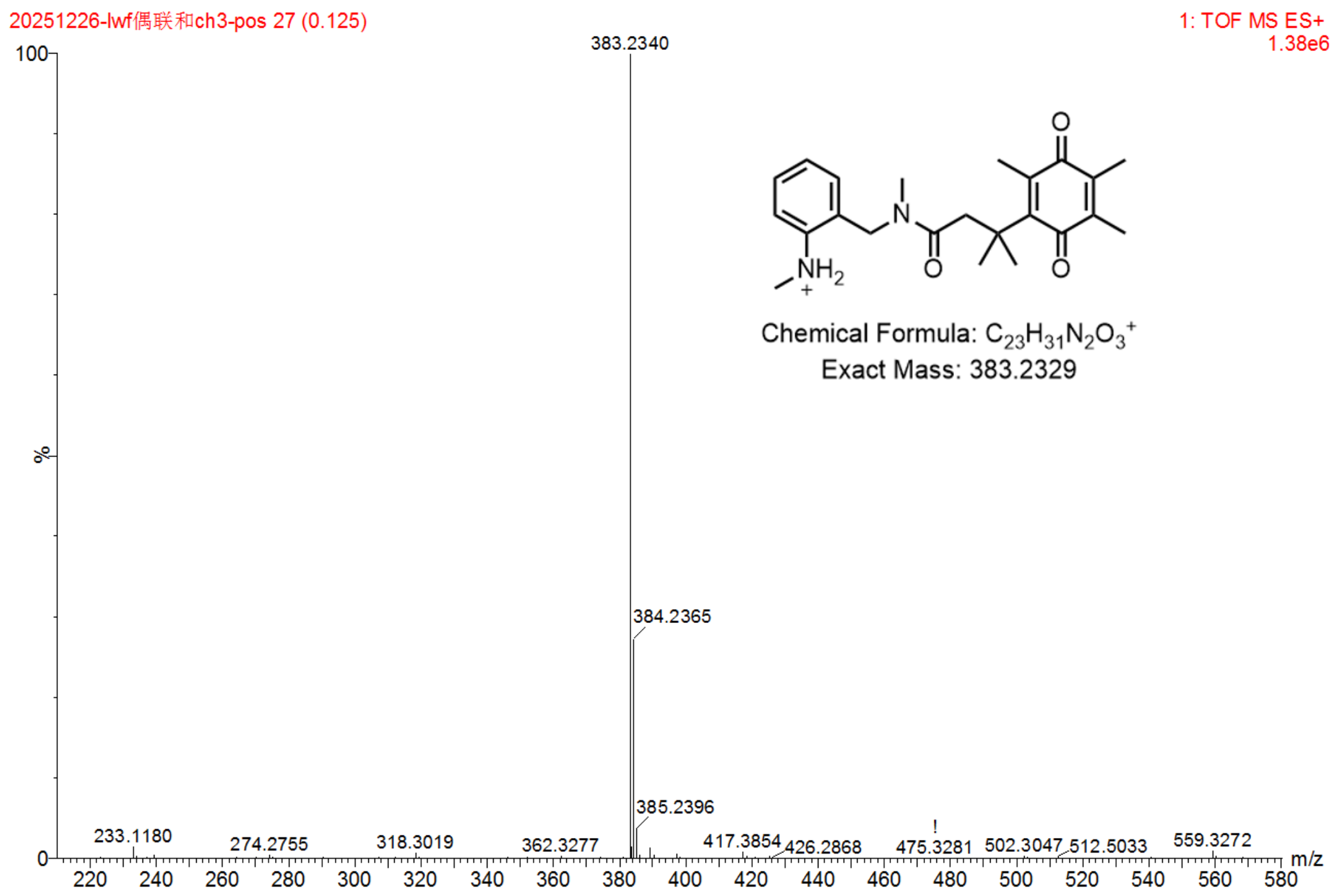

HRMS (ESI) of compound **13**.

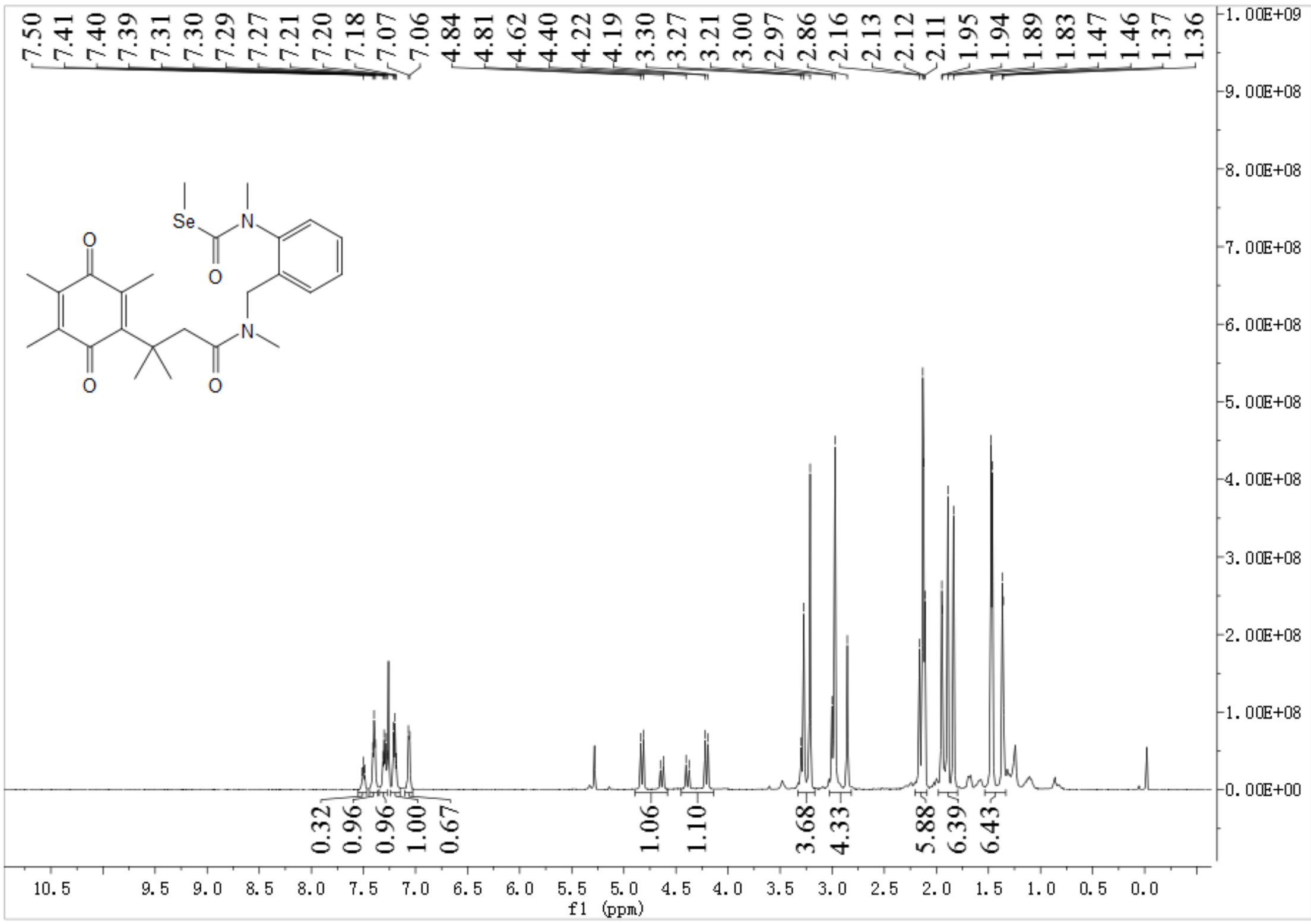

^{1}H NMR (400 MHz, chloroform-*d*) spectrum of compound **TD-MSe3**.

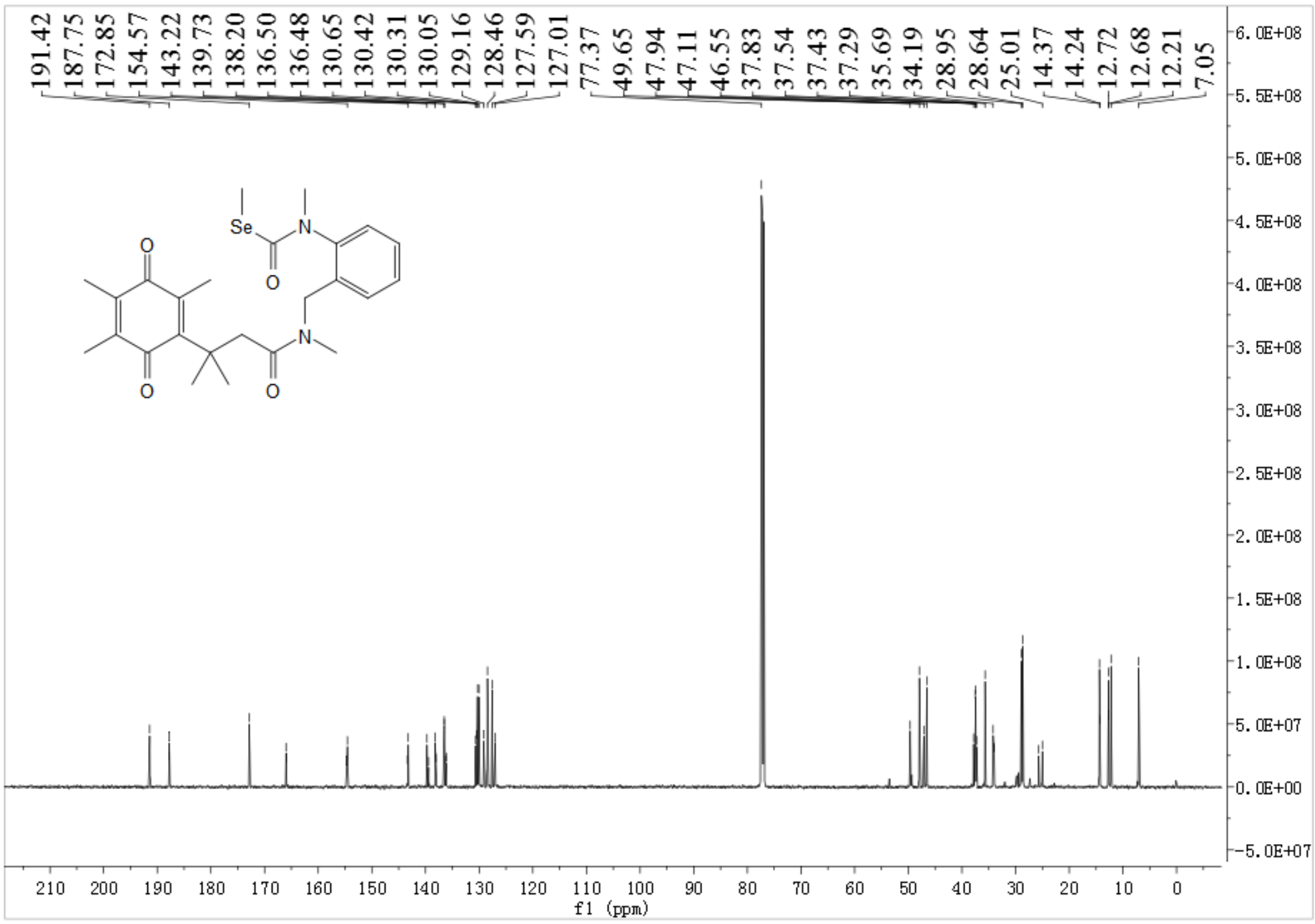

^{13}C{^{1}H} NMR (101 MHz, chloroform-*d*) spectrum of compound **TD-MSe3**.

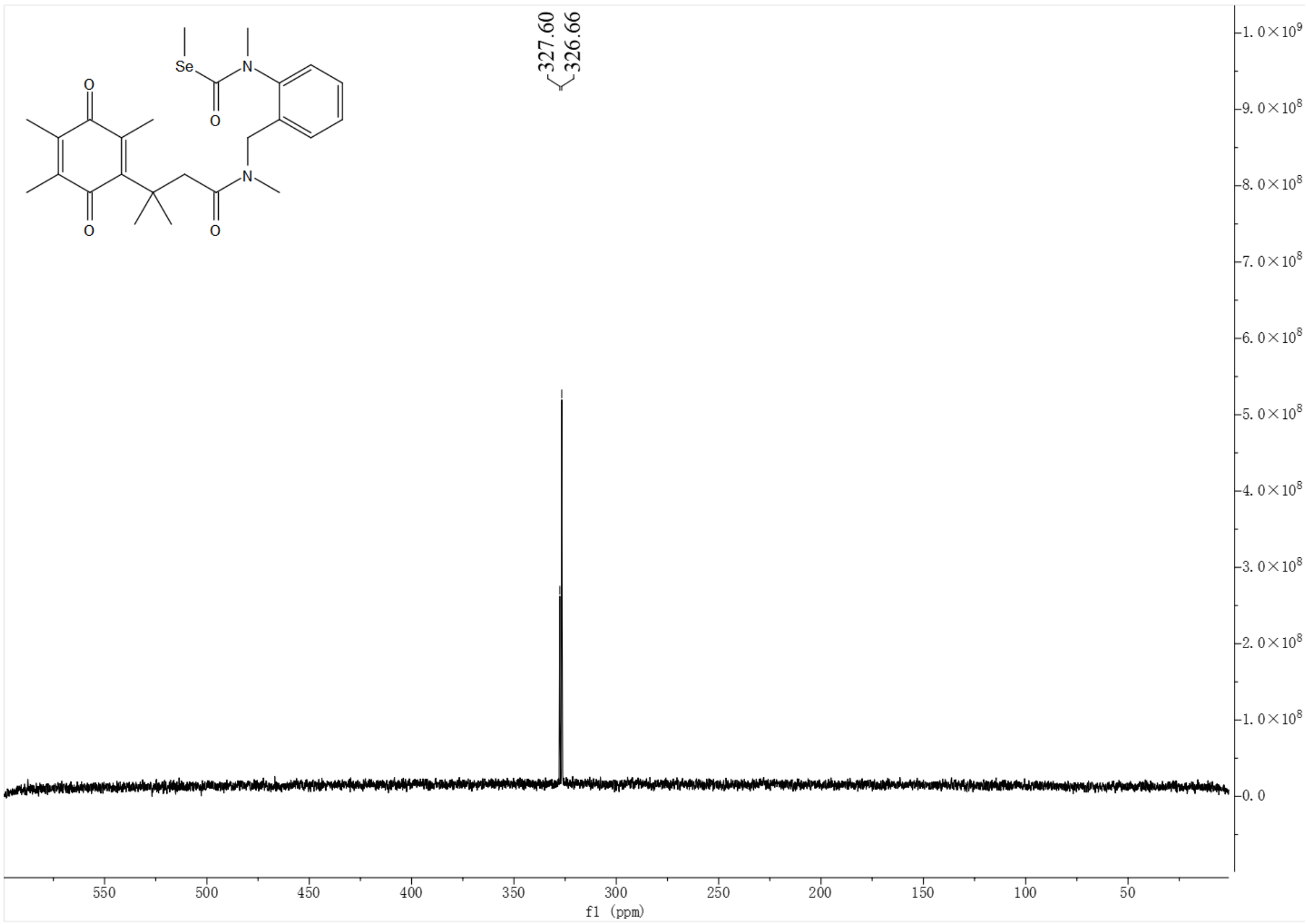

$^{77}Se\{^{1}H\}$ NMR (114 MHz, chloroform-*d*) spectrum of compound **TD-MSe3**.

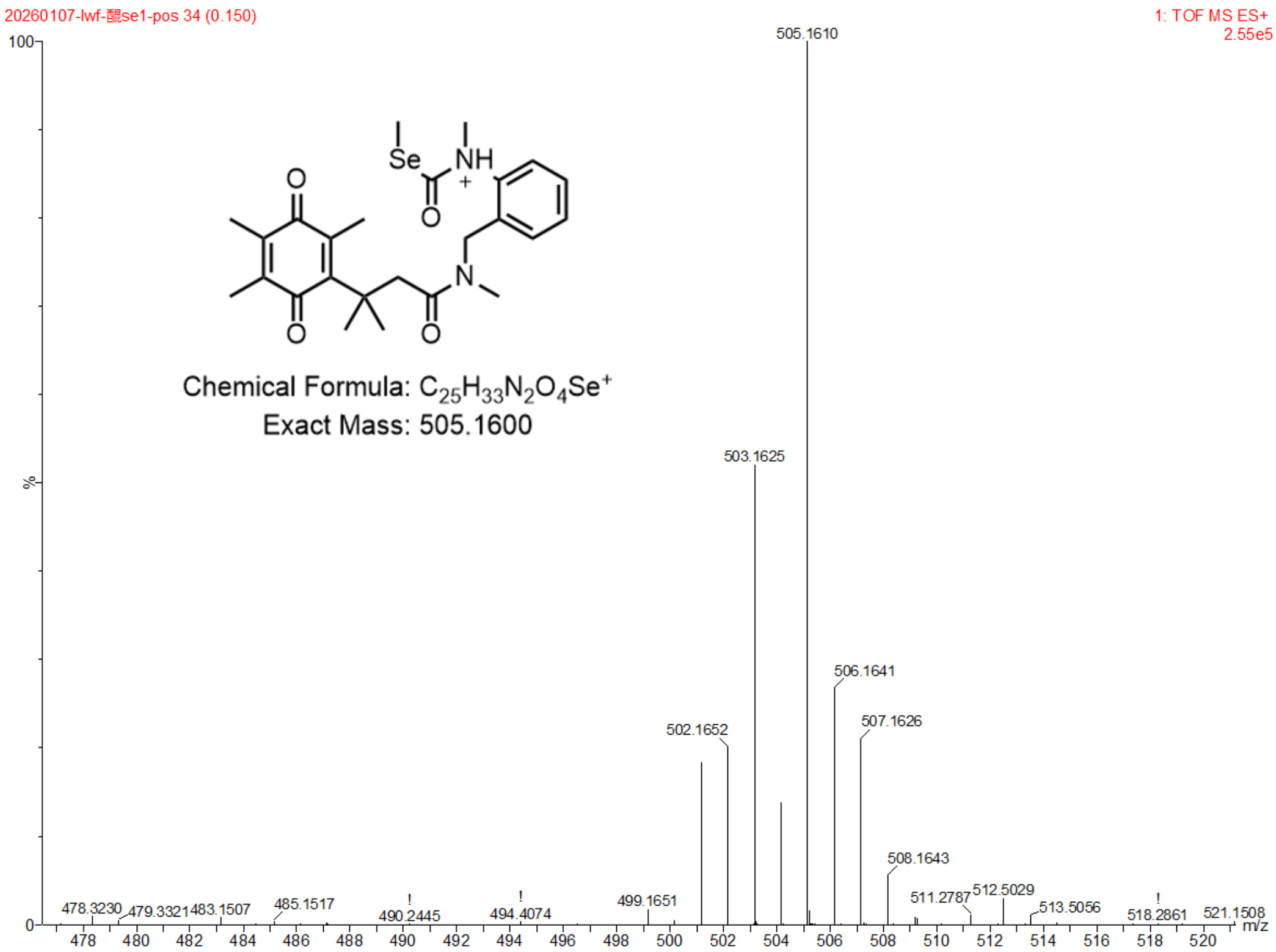

HRMS (ESI) of compound **TD-MSe3**.

6. SI References

1. C. A. Bayse, "Considerations for Reliable Calculation of ^{77}Se Chemical Shifts," *Journal of Chemical Theory and Computation* 1, no. 6 (2005): 1119–1127. https://doi.org/10.1021/ct050136t
2. Y. Dong, W. Liang and L. Yi, "Fast Intramolecular Thiol-Activated Arylselenoamides Provide Access to Triggered, Fluorogenic H_2Se Donors," *Journal of the American Chemical Society* 146, no. 36 (2024): 24776–24781. https://doi.org/10.1021/jacs.4c09215
3. C. Zhang, Q-Z. Zhang, K. Zhang, et al., "Dual-Biomarker-Triggered Fluorescence Probes for Differentiating Cancer Cells and Revealing Synergistic Antioxidant Effects under Oxidative Stress," *Chemical Science* 10, no. 7 (2019): 1945–1952. https://doi.org/10.1039/c8sc03781g
4. X. Kang, H. Huang, C. Jiang, et al., "Cysteine-Activated Small-Molecule H_2Se Donors Inspired by Synthetic H_2S Donors," *Journal of the American Chemical Society* 144, no. 9 (2022): 3957–3967. https://doi.org/10.1021/jacs.1c12006
5. T. D. Newton, S. G. Bolton, A. C. Garcia, et al., "Hydrolysis-Based Small-Molecule Hydrogen Selenide (H_2Se) Donors for Intracellular H_2Se Delivery," *Journal of the American Chemical Society* 143, no. 46 (2021): 19542–19550. https://doi.org/10.1021/jacs.1c09525
6. X. Cai, J. Zhang and H. Ye, et al., "Ligand-Directed H_2S Probe and Scavenger for Specific Tumor Imaging," *Chemistry - A European Journal* 29, no. 44 (2023): e202301105. https://doi.org/10.1002/chem.202301105
7. H. Ishihara, M. Koketsu, Y. Fukuta and F. Nada, "Reaction of Lithium Aluminum Hydride with Elemental Selenium: Its Application as a Selenating Reagent into Organic Molecules," *Journal of the American Chemical Society* 123, no. 34 (2001): 8408–8409. https://doi.org/10.1021/ja005800o
8. M. Koketsu, M. Ishida, N. Takakura and H. Ishihara, "Preparation and Characterization of N-Alkyl-Se-alkylselenocarbamates," *Journal of Organic Chemistry* 67, no. 2 (2002): 486–490. https://doi.org/10.1021/jo010763k
9. B. Zhang, C. Ge, J. Yao, Y. Liu, H. Xie and J. Fang, "Selective Selenol Fluorescent Probes: Design, Synthesis, Structural Determinants, and Biological Applications," *Journal of the American Chemical Society* 137, no. 2 (2015): 757–769. https://doi.org/10.1021/ja5099676
10. Y. Dong, H. Ye and B. Wang, et al., "Intramolecular Thiol-Promoted Decomposition of Cysteine Ester (ITPDC): A General Platform for Controllable Release of Reactive Sulfur Species," *Angewandte Chemie International Edition* 64, no. 26 (2025): e202422087. https://doi.org/10.1002/anie.202422087
11. A. Göblyös, L. Lázár and F. Fülöp, "Ring-Chain Tautomerism of 2-Aryl-Substituted-Hexahydropyrimidines and Tetrahydroquinazolines," *Tetrahedron* 58, no. 5 (2002): 1011–1016. https://doi.org/10.1016/S0040-4020(01)01196-6
12. J. Li, C. Zhang, S. Wu, et al., "Facile Synthesis of Green-Light and Large Stokes-Shift Emitting Coumarins for Bioconjugation," *Dyes and Pigments* 151 (2018): 303–309. https://doi.org/10.1016/j.dyepig.2018.01.016
13. M. P. Hoffman and S. S. Xantheas, "Engineering the Formation of Chalcogen Bonds with Water: I. Heterodimers of the Chalcogen Hydrides H_2O–H_2X (X = S, Se, Te)," *Journal of Physical Chemistry A* 129, no. 49 (2025): 11328–11337. https://doi.org/10.1021/acs.jpca.5c06655
